# Supplementary material for: Triadic communication with teenagers and young adults with cancer: a systematic literature review – ‘make me feel like I’m not the third person’
Source: BMJ Open. 2024 Feb 17;14(2):e080024. doi: 10.1136/bmjopen-2023-080024 (PMC10875529; doi:10.1136/bmjopen-2023-080024)
Supplement: Supplementary data [file bmjopen-2023-080024supp001.pdf]

Update – December 2023

Medline

Ovid MEDLINE(R) and Epub Ahead of Print, In-Process, In-Data-Review & Other Non-Indexed Citations, Daily and Versions <1946 to December 19, 2023>

1 (Parent* or guardian* or mother* or father* or partner or wife or wives or husband* or boyfriend* or girlfriend* or sibling* or friend* or carer* or "third person" or caregiver* or "care-giver*" or spouse* or supporter* or support network*).ti,ab. or parents/ or fathers/ or mothers/ or spouses/ or caregivers/ or siblings/ or friends/ or legal guardians/ 1148168

2 (TYA cancer or TYA oncology or AYA cancer or AYA oncology or (young adult adj3 (cancer or oncology or leuk?em* or lymphom* or h?ematol*)) or ("teenage and young adult" adj3 cancer) or ("teenage and young adult" adj3 oncology) or (teenage* adj3 cancer) or (teenage* adj3 oncology) or (adolescen* adj3 cancer) or (adolescen* adj3 oncology) or (young people adj3 cancer) or (young people adj3 oncology) or ("teenage and young adult" adj3 leuk?emia*) or (teenage* adj3 leuk?emia*) or (adolescen* adj3 leuk?emia*) or (young people adj3 leuk?emia*) or (young adult adj3 leuk?emia*) or ("teenage and young adult" adj3 h?ematol*) or (teenage* adj3 h?ematol*) or (adolescen* adj3 h?ematol*) or (young people adj3 h?ematol*) or (young adult adj3 h?ematol*) or ("teenage and young adult" adj3 lymphom*) or (teenage* adj3 lymphom*) or (adolescen* adj3 lymphom*) or (young people adj3 lymphom*) or (young adult adj3 lymphom*)).ti,ab. or ((exp adolescent/ or exp young adult/) and exp neoplasms/) 337284

3 (Communicat* or Disclos* or inform* or Interact* or relationship* or Conversation* or Dialogue* or triad* or Interview* or consult* or decision making).ti,ab. or exp communication/ or exp disclosure/ or exp information dissemination/ or exp physician-patient relations/ 6147070

4 (affect* or effect* or influenc* or resultant or impact* or perception* or perspective* or encounter* or preference or opinion or involvement or occurance* or feel or "go through" or experienc*).ti,ab. 13278879

5 1 and 2 and 3 and 4 3519

6 limit 5 to english language 3399

7 (202211* or 202212* or 2023* or 2024*).dp. or (202211* or 202212* or 2023* or 2024*).ez. or (202211* or 202212* or 2023* or 2024*).ed. or (202211* or 202212* or 2023* or 2024*).ep. 1958643

8 6 and 7 163

Embase

Embase <1974 to 2023 December 19>

1 (TYA cancer or TYA oncology or AYA cancer or AYA oncology or (young adult adj3 (cancer or oncology or leuk?em* or lymphom* or h?ematol*)) or ("teenage and young adult" adj3 cancer) or ("teenage and young adult" adj3 oncology) or (teenage* adj3 cancer) or (teenage* adj3 oncology) or (adolescen* adj3 cancer) or (adolescen* adj3 oncology) or (young people adj3 cancer) or (young people adj3 oncology) or ("teenage and young adult" adj3 leuk?emia*) or (teenage* adj3 leuk?emia*) or (adolescen* adj3 leuk?emia*) or (young people adj3 leuk?emia*) or (young adult adj3 leuk?emia*) or ("teenage and young adult" adj3 h?ematol*) or (teenage* adj3 h?ematol*) or (adolescen* adj3 h?ematol*) or (young people adj3 h?ematol*) or (young adult adj3 h?ematol*) or ("teenage and young adult" adj3 lymphom*) or (teenage* adj3 lymphom*) or (adolescen* adj3 lymphom*) or (young people adj3 lymphom*) or (young adult adj3 lymphom*)).ti,ab. or ((exp *adolescent/ or exp *young adult/) and exp *neoplasm/) 10359
2 (Communicat* or Disclos* or inform* or Interact* or relationship* or Conversation* or Dialogue* or triad* or Interview* or consult* or decision making).ti,ab. or exp *interpersonal communication/ or exp *professional-patient relationship/ or exp *information dissemination/ or exp *conversation/ 7565603
3 (Parent* or guardian* or mother* or father* or partner or wife or wives or husband* or boyfriend* or girlfriend* or sibling* or friend* or carer* or "third person" or caregiver* or "care-giver*" or spouse* or supporter* or support network*).ti,ab. or *parent/ or *father/ or *mother/ or *spouse/ or *caregiver/ or *social worker/ or *sibling/ or *friend/ or *legal guardian/ 1440315
4 (affect* or effect* or influenc* or resultant or impact* or perception* or perspective* or encounter* or preference or opinion or involvement or occurance* or feel or "go through" or experienc*).ti,ab. 16593794
5 1 and 2 and 3 and 4 1032
6 limit 5 to english language 1010
7 (202211* or 202212* or 2023* or 2024*).dc. or (202211* or 202212* or 2023* or 2024*).dd. or (202211* or 202212* or 2023* or 2024*).dp. 2647560
8 6 and 7 107

PsycINFO (via Ebsco)

#	Query	Limiters/Expanders	Last Run Via	Results
S11	S1 AND S2 AND S7 AND S8	Limiters - Publication Date: 20221101-20241231 Expanders - Apply equivalent subjects Search modes - Boolean/Phrase	Interface - EBSCOhost Research Databases Search Screen - Basic Search Database - APA PsycInfo	238
S10	S1 AND S2 AND S7 AND S8	Expanders - Apply equivalent subjects Narrow by	Interface - EBSCOhost Research	6,315

		Language: - english Search modes - Boolean/Phrase	Databases Search Screen - Basic Search Database - APA PsycInfo	
S9	S1 AND S2 AND S7 AND S8	Expanders - Apply equivalent subjects Search modes - Boolean/Phrase	Interface - EBSCOhost Research Databases Search Screen - Basic Search Database - APA PsycInfo	6,549
S8	(affect* or effect* or influenc* or resultant or impact* or perception* or perspective* or encounter* or preference or opinion or involvement or occurance* or feel or "go through" or experienc*)	Expanders - Apply equivalent subjects Search modes - Boolean/Phrase	Interface - EBSCOhost Research Databases Search Screen - Basic Search Database - APA PsycInfo	3,524,144
S7	(S5) or (S3)	Expanders - Apply equivalent subjects Search modes - Boolean/Phrase	Interface - EBSCOhost Research Databases Search Screen - Basic Search Database - APA PsycInfo	62,557
S6	S4 AND S5	Expanders - Apply equivalent subjects Search modes - Boolean/Phrase	Interface - EBSCOhost Research Databases Search Screen - Basic Search Database - APA PsycInfo	62,384

S5	((DE "neoplasms" OR DE "Benign Neoplasms" OR DE "Breast Neoplasms" OR DE "Endocrine Neoplasms" OR DE "Leukemias" OR DE "Melanoma" OR DE "Metastasis" OR DE "Nervous System Neoplasms" OR DE "Terminal Cancer"))	Expanders - Apply equivalent subjects Search modes - Boolean/Phrase	Interface - EBSCOhost Research Databases Search Screen - Basic Search Database - APA PsycInfo	62,384
S4	((DE "neoplasms" OR DE "Benign Neoplasms" OR DE "Breast Neoplasms" OR DE "Endocrine Neoplasms" OR DE "Leukemias" OR DE "Melanoma" OR DE "Metastasis" OR DE "Nervous System Neoplasms" OR DE "Terminal Cancer"))	Expanders - Apply equivalent subjects Search modes - Boolean/Phrase	Interface - EBSCOhost Research Databases Search Screen - Basic Search Database - APA PsycInfo	62,384
S3	("TYA cancer" or "TYA oncology" or "AYA cancer" or "AYA oncology" or ("young adult" n3 (cancer or oncology or leuk?em* or lymphom* or h?ematol*)) or ("teenage and young adult" n3 cancer) or ("teenage and young adult" n3 oncology) or (teenage* n3 cancer) or (teenage* n3 oncology) or (adolescen* n3 cancer) or (adolescen* n3 oncology) or ("young people" n3 cancer) or ("young people" n3 oncology) or ("teenage and young adult" n3 leuk?emia*) or (teenage* n3 leuk?emia*) or (adolescen* n3 leuk?emia*) or ("young people" n3 leuk?emia*) or ("young adult" n3 leuk?emia*) or ("teenage and young adult" n3 h?ematol*) or (teenage* n3 h?ematol*) or (adolescen* n3 h?ematol*) or ("young people" n3 h?ematol*) or ("young adult" n3 h?ematol*) or ("teenage and young adult" n3 lymphom*) or (teenage* n3 lymphom*) or (adolescen* n3 lymphom*) or ("young people" n3 lymphom*) or ("young adult" n3 lymphom*))	Expanders - Apply equivalent subjects Search modes - Boolean/Phrase	Interface - EBSCOhost Research Databases Search Screen - Basic Search Database - APA PsycInfo	2,072
S2	(Communicat* or Disclos* or inform* or Interact* or relationship* or Conversation* or Dialogue* or triad* or Interview* or consult* or "decision making") or DE "communication" OR DE "information dissemination" OR DE "conversation"	Expanders - Apply equivalent subjects Search modes - Boolean/Phrase	Interface - EBSCOhost Research Databases Search Screen - Basic Search Database - APA PsycInfo	2,545,968

S1	(Parent* or guardian* or mother* or father* or partner or wife or wives or husband* or boyfriend* or girlfriend* or sibling* or friend* or teacher* or social worker* or carer* or "third person" or caregiver* or "care-giver*" or spouse* or chaperone*) OR DE “parents” OR DE “mothers” OR DE “fathers” OR DE “spouses” OR DE “wives” OR DE “husbands” OR DE “siblings” OR DE “significant others” OR DE “social workers” OR DE “guardianship” OR DE “caregivers”	Expanders - Apply equivalent subjects Search modes - Boolean/Phrase	Interface - EBSCOhost Research Databases Search Screen - Basic Search Database - APA PsycInfo	937,154

CINAHL (via Ebsco)

Wednesday, December 20, 2023 4:07:56 PM

#	Query	Limiters/Expanders	Last Run Via	Results
S7	S1 AND S2 AND S3 AND S4	Limiters - Publication Date: 20221101-20241231 Expanders - Apply equivalent subjects Search modes - Boolean/Phrase	Interface - EBSCOhost Research Databases Search Screen - Advanced Search Database - CINAHL	99
S6	S1 AND S2 AND S3 AND S4	Expanders - Apply equivalent subjects Narrow by Language: - english Search modes - Boolean/Phrase	Interface - EBSCOhost Research Databases Search Screen - Advanced Search Database - CINAHL	2,152
S5	S1 AND S2 AND S3 AND S4	Expanders - Apply equivalent subjects Search modes - Boolean/Phrase	Interface - EBSCOhost Research Databases Search Screen - Advanced Search Database - CINAHL	2,189

S4	(affect* or effect* or influenc* or resultant or impact* or perception* or perspective* or encounter* or preference or opinion or involvement or occurance* or feel or "go through" or experienc*)	Expanders - Apply equivalent subjects Search modes - Boolean/Phrase	Interface - EBSCOhost Research Databases Search Screen - Advanced Search Database - CINAHL	3,149,468
S3	("TYA cancer" or "TYA oncology" or "AYA cancer" or "AYA oncology" or ("young adult" n3 (cancer or oncology or leuk?em* or lymphom* or h?ematol*)) or ("teenage and young adult" n3 cancer) or ("teenage and young adult" n3 oncology) or (teenage* n3 cancer) or (teenage* n3 oncology) or (adolescen* n3 cancer) or (adolescen* n3 oncology) or ("young people" n3 cancer) or ("young people" n3 oncology) or ("teenage and young adult" n3 leuk?emia*) or (teenage* n3 leuk?emia*) or (adolescen* n3 leuk?emia*) or ("young people" n3 leuk?emia*) or ("young adult" n3 leuk?emia*) or ("teenage and young adult" n3 h?ematol*) or (teenage* n3 h?ematol*) or (adolescen* n3 h?ematol*) or ("young people" n3 h?ematol*) or ("young adult" n3 h?ematol*) or ("teenage and young adult" n3 lymphom*) or (teenage* n3 lymphom*) or (adolescen* n3 lymphom*) or ("young people" n3 lymphom*) or ("young adult" n3 lymphom*)) OR ((MH “adolescence+” OR MH “young adult+”) AND (MH “neoplasms+”))	Expanders - Apply equivalent subjects Search modes - Boolean/Phrase	Interface - EBSCOhost Research Databases Search Screen - Advanced Search Database - CINAHL	61,263
S2	(Communicat* or Disclos* or inform* or Interact* or relationship* or Conversation* or Dialogue* or triad* or Interview* or consult* or "decision making") or MH “communication+” OR MH “discussion” OR MH “conversation”	Expanders - Apply equivalent subjects Search modes - Boolean/Phrase	Interface - EBSCOhost Research Databases Search Screen - Advanced	2,080,061

	OR (MH "Professional-Patient Relations+")		Search Database - CINAHL	
S1	(Parent* or guardian* or mother* or father* or partner or wife or wives or husband* or boyfriend* or girlfriend* or sibling* or friend* or teacher* or social worker* or carer* or "third person" or caregiver* or "care-giver*" or spouse* or chaperone*) OR MH “parents” OR MH “mothers” OR MH “fathers” OR MH “spouses” OR MH “siblings” OR MH “teachers” OR MH “social workers” OR MH “caregivers”	Expanders - Apply equivalent subjects Search modes - Boolean/Phrase	Interface - EBSCOhost Research Databases Search Screen - Advanced Search Database - CINAHL	567,768

Web of Science Core Collection

Web of Science Search Strategy (v0.1)

Database: Web of Science Core Collection

Entitlements:

- WOS.IC: 1993 to 2023
- WOS.CCR: 1985 to 2023
- WOS.SCI: 1900 to 2023
- WOS.AHCI: 1975 to 2023
- WOS.BHCI: 2008 to 2023
- WOS.BSCI: 2008 to 2023
- WOS.ESCI: 2018 to 2023
- WOS.ISTP: 1990 to 2023
- WOS.SSCI: 1956 to 2023
- WOS.ISSHP: 1990 to 2023

Searches:

1: TS=(Parent* or guardian* or mother* or father* or partner or wife or wives or husband* or boyfriend* or girlfriend* or sibling* or friend* or teacher* or social worker* or carer* or "third person" or caregiver* or "care-giver*" or spouse* or chaperone*)

Date Run: Wed Dec 20 2023 16:03:59 GMT+0000 (Greenwich Mean Time)

Results: 2283955

2: TS=("TYA cancer" or "TYA oncology" or "AYA cancer" or "AYA oncology" or ("young adult" near/3 (cancer or oncology or leuk?em* or lymphom* or h?ematol*)) or ("teenage and young adult" near/3 cancer) or ("teenage and young adult" near/3 oncology) or (teenage*

near/3 cancer) or (teenage* near/3 oncology) or (adolescen* near/3 cancer) or (adolescen* near/3 oncology) or ("young people" near/3 cancer) or ("young people" near/3 oncology) or ("teenage and young adult" near/3 leuk?emia*) or (teenage* near/3 leuk?emia*) or (adolescen* near/3 leuk?emia*) or ("young people" near/3 leuk?emia*) or ("young adult" near/3 leuk?emia*) or ("teenage and young adult" near/3 h?ematol*) or (teenage* near/3 h?ematol*) or (adolescen* near/3 h?ematol*) or ("young people" near/3 h?ematol*) or ("young adult" near/3 h?ematol*) or ("teenage and young adult" near/3 lymphom*) or (teenage* near/3 lymphom*) or (adolescen* near/3 lymphom*) or ("young people" near/3 lymphom*) or ("young adult" near/3 lymphom*)) Date Run: Wed Dec 20 2023 16:04:06 GMT+0000 (Greenwich Mean Time) Results: 8540

3: TS=(Communicat* or Disclos* or inform* or Interact* or relationship* or Conversation* or Dialogue* or triad* or Interview* or consult* or "decision making") Date Run: Wed Dec 20 2023 16:04:13 GMT+0000 (Greenwich Mean Time) Results: 12748181

4: TS= (affect* or effect* or influenc* or resultant or impact* or perception* or perspective* or encounter* or preference or opinion or involvement or occurance* or feel or "go through" or experienc*) Date Run: Wed Dec 20 2023 16:04:19 GMT+0000 (Greenwich Mean Time) Results: 26006930

5: #4 AND #3 AND #2 AND #1 Date Run: Wed Dec 20 2023 16:04:26 GMT+0000 (Greenwich Mean Time) Results: 764

6: #4 AND #3 AND #2 AND #1 and English (Languages) Date Run: Wed Dec 20 2023 16:04:35 GMT+0000 (Greenwich Mean Time) Results: 737

7: #4 AND #3 AND #2 AND #1 and English (Languages) and 2022 or 2023 (Publication Years) Date Run: Wed Dec 20 2023 16:04:39 GMT+0000 (Greenwich Mean Time) Results: 132

AMED via Ovid

AMED (Allied and Complementary Medicine) <1985 to October 2023>

1 (Parent* or guardian* or mother* or father* or partner or wife or wives or husband* or boyfriend* or girlfriend* or sibling* or friend* or carer* or "third person" or caregiver* or "care-giver*" or spouse* or supporter* or support network*).ti,ab. 14291

2 (TYA cancer or TYA oncology or AYA cancer or AYA oncology or (young adult adj3 (cancer or oncology or leuk?em* or lymphom* or h?ematol*)) or ("teenage and young adult" adj3 cancer) or ("teenage and young adult" adj3 oncology) or (teenage* adj3 cancer) or (teenage* adj3 oncology) or (adolescen* adj3 cancer) or (adolescen* adj3 oncology) or (young people adj3 cancer) or (young people adj3 oncology) or ("teenage and young adult" adj3 leuk?emia*) or (teenage* adj3 leuk?emia*) or (adolescen* adj3 leuk?emia*) or (young people adj3 leuk?emia*) or (young adult adj3 leuk?emia*) or ("teenage and young adult" adj3 h?ematol*) or (teenage* adj3 h?ematol*) or (adolescen* adj3 h?ematol*) or (young people adj3 h?ematol*) or (young adult adj3 h?ematol*) or ("teenage and young adult" adj3

lymphom*) or (teenage* adj3 lymphom*) or (adolescen* adj3 lymphom*) or (young people adj3 lymphom*) or (young adult adj3 lymphom*)).ti,ab. 120
3 (Communicat* or Disclos* or inform* or Interact* or relationship* or Conversation* or Dialogue* or triad* or Interview* or consult* or decision making).ti,ab. 60609
4 (affect* or effect* or influenc* or resultant or impact* or perception* or perspective* or encounter* or preference or opinion or involvement or occurance* or feel or "go through" or experienc*).ti,ab. 143225
5 1 and 2 and 3 and 4 19
6 limit 5 to yr="2005 -Current" 14

First run – November 2022

Medline (via Ovid)

Ovid MEDLINE(R) and Epub Ahead of Print, In-Process, In-Data-Review & Other Non-Indexed Citations, Daily and Versions <1946 to November 23, 2022>

1 (Parent* or guardian* or mother* or father* or partner or wife or wives or husband* or boyfriend* or girlfriend* or sibling* or friend* or carer* or "third person" or caregiver* or "care-giver*" or spouse* or supporter* or support network*).ti,ab. or parents/ or fathers/ or mothers/ or spouses/ or caregivers/ or siblings/ or friends/ or legal guardians/ 1074121

2 (TYA cancer or TYA oncology or AYA cancer or AYA oncology or (young adult adj3 (cancer or oncology or leuk?em* or lymphom* or h?ematol*)) or ("teenage and young adult" adj3 cancer) or ("teenage and young adult" adj3 oncology) or (teenage* adj3 cancer) or (teenage* adj3 oncology) or (adolescen* adj3 cancer) or (adolescen* adj3 oncology) or (young people adj3 cancer) or (young people adj3 oncology) or ("teenage and young adult" adj3 leuk?emia*) or (teenage* adj3 leuk?emia*) or (adolescen* adj3 leuk?emia*) or (young people adj3 leuk?emia*) or (young adult adj3 leuk?emia*) or ("teenage and young adult" adj3 h?ematol*) or (teenage* adj3 h?ematol*) or (adolescen* adj3 h?ematol*) or (young people adj3 h?ematol*) or (young adult adj3 h?ematol*) or ("teenage and young adult" adj3 lymphom*) or (teenage* adj3 lymphom*) or (adolescen* adj3 lymphom*) or (young people adj3 lymphom*) or (young adult adj3 lymphom*)).ti,ab. or ((exp adolescent/ or exp young adult/) and exp neoplasms/) 333070

3 (Communicat* or Disclos* or inform* or Interact* or relationship* or Conversation* or Dialogue* or triad* or Interview* or consult* or decision making).ti,ab. or exp communication/ or exp disclosure/ or exp information dissemination/ or exp physician-patient relations/ 5715959

4 (affect* or effect* or influenc* or resultant or impact* or perception* or perspective* or encounter* or preference or opinion or involvement or occurance* or feel or "go through" or experienc*).ti,ab. 12406352

5 1 and 2 and 3 and 4 3380

6 limit 5 to (english language and yr="2005 -Current") 2715

Embase (via Ovid)

Embase <1974 to 2022 November 23>

1 (TYA cancer or TYA oncology or AYA cancer or AYA oncology or (young adult adj3 (cancer or oncology or leuk?em* or lymphom* or h?ematol*)) or ("teenage and young adult" adj3 cancer) or ("teenage and young adult" adj3 oncology) or (teenage* adj3 cancer) or (teenage* adj3 oncology) or (adolescen* adj3 cancer) or (adolescen* adj3 oncology) or (young people adj3 cancer) or (young people adj3 oncology) or ("teenage and young adult" adj3 leuk?emia*) or (teenage* adj3 leuk?emia*) or (adolescen* adj3 leuk?emia*) or (young people adj3 leuk?emia*) or (young adult adj3 leuk?emia*) or ("teenage and young adult" adj3 h?ematol*) or (teenage* adj3 h?ematol*) or (adolescen* adj3 h?ematol*) or (young people adj3 h?ematol*) or (young adult adj3 h?ematol*) or ("teenage and young adult" adj3 lymphom*) or (teenage* adj3 lymphom*) or (adolescen* adj3 lymphom*) or (young people adj3 lymphom*) or (young adult adj3 lymphom*)).ti,ab. or ((exp *adolescent/ or exp *young adult/) and exp *neoplasm/) 9638

2 (Communicat* or Disclos* or inform* or Interact* or relationship* or Conversation* or Dialogue* or triad* or Interview* or consult* or decision making).ti,ab. or exp *interpersonal communication/ or exp *professional-patient relationship/ or exp *information dissemination/ or exp *conversation/ 6997005

3 (Parent* or guardian* or mother* or father* or partner or wife or wives or husband* or boyfriend* or girlfriend* or sibling* or friend* or carer* or "third person" or caregiver* or "care-giver*" or spouse* or supporter* or support network*).ti,ab. or *parent/ or *father/ or *mother/ or *spouse/ or *caregiver/ or *social worker/ or *sibling/ or *friend/ or *legal guardian/ 1339977

4 (affect* or effect* or influenc* or resultant or impact* or perception* or perspective* or encounter* or preference or opinion or involvement or occurance* or feel or "go through" or experienc*).ti,ab. 15453173

5 1 and 2 and 3 and 4 939

6 limit 5 to (english language and yr="2005 -Current") 873

PsycInfo (via Ebscohost)

#	Query	Limiters/Expanders	Last Run Via	Results
S11	S1 AND S2 AND S7 AND S8	Limiters - Publication Year: 2005-2022<br>Expanders - Apply equivalent subjects<br>Narrow by Language: - english<br>Search modes - Boolean/Phrase	Interface - EBSCOhost Research Databases<br>Search Screen - Basic Search<br>Database - APA PsycInfo	1,683
S10	S1 AND S2 AND S7 AND S8	Expanders - Apply equivalent subjects<br>Narrow by Language: - english	Interface - EBSCOhost Research Databases<br>Search Screen - Basic Search	1,981

		Search modes - Boolean/Phrase	Database - APA PsycInfo	
S9	S1 AND S2 AND S7 AND S8	Expanders - Apply equivalent subjects<br>Search modes - Boolean/Phrase	Interface - EBSCOhost Research Databases<br>Search Screen - Basic Search<br>Database - APA PsycInfo	2,017
S8	(affect* or effect* or influenc* or resultant or impact* or perception* or perspective* or encounter* or preference or opinion or involvement or occurance* or feel or "go through" or experienc*)	Expanders - Apply equivalent subjects<br>Search modes - Boolean/Phrase	Interface - EBSCOhost Research Databases<br>Search Screen - Basic Search<br>Database - APA PsycInfo	3,366,619
S7	(S5) or (S3)	Expanders - Apply equivalent subjects<br>Search modes - Boolean/Phrase	Interface - EBSCOhost Research Databases<br>Search Screen - Basic Search<br>Database - APA PsycInfo	13,719
S6	S4 AND S5	Expanders - Apply equivalent subjects<br>Search modes - Boolean/Phrase	Interface - EBSCOhost Research Databases<br>Search Screen - Basic Search<br>Database - APA PsycInfo	13,275
S5	((DE “neoplasms” OR DE "Benign Neoplasms" OR DE "Breast Neoplasms" OR DE "Endocrine Neoplasms" OR DE "Leukemias" OR DE "Melanoma" OR DE "Metastasis" OR DE "Nervous System Neoplasms" OR DE "Terminal Cancer"))	Expanders - Apply equivalent subjects<br>Narrow by SubjectAge: - adolescence (13-17 yrs)<br>Narrow by SubjectAge: - young adulthood (18-29 yrs)<br>Search modes - Boolean/Phrase	Interface - EBSCOhost Research Databases<br>Search Screen - Basic Search<br>Database - APA PsycInfo	13,275
S4	((DE “neoplasms” OR DE "Benign Neoplasms" OR DE "Breast Neoplasms" OR DE "Endocrine Neoplasms" OR DE "Leukemias" OR DE "Melanoma" OR DE "Metastasis" OR DE "Nervous System Neoplasms" OR DE "Terminal Cancer"))	Expanders - Apply equivalent subjects<br>Search modes - Boolean/Phrase	Interface - EBSCOhost Research Databases<br>Search Screen - Basic Search<br>Database - APA PsycInfo	58,767
S3	("TYA cancer" or "TYA oncology" or "AYA cancer" or "AYA oncology" or ("young adult" n3 (cancer or oncology or leuk?em* or lymphom* or h?ematol*)) or ("teenage and young adult" n3 cancer) or ("teenage and young adult" n3 oncology) or (teenage* n3 cancer) or (teenage* n3 oncology) or (adolescen* n3 cancer) or	Expanders - Apply equivalent subjects<br>Search modes - Boolean/Phrase	Interface - EBSCOhost Research Databases<br>Search Screen - Basic Search<br>Database - APA PsycInfo	1,864

	(adolescen* n3 oncology) or ("young people" n3 cancer) or ("young people" n3 oncology) or ("teenage and young adult" n3 leuk?emia*) or (teenage* n3 leuk?emia*) or (adolescen* n3 leuk?emia*) or ("young people" n3 leuk?emia*) or ("young adult" n3 leuk?emia*) or ("teenage and young adult" n3 h?ematol*) or (teenage* n3 h?ematol*) or (adolescen* n3 h?ematol*) or ("young people" n3 h?ematol*) or ("young adult" n3 h?ematol*) or ("teenage and young adult" n3 lymphom*) or (teenage* n3 lymphom*) or (adolescen* n3 lymphom*) or ("young people" n3 lymphom*) or ("young adult" n3 lymphom*))			
S2	(Communicat* or Disclos* or inform* or Interact* or relationship* or Conversation* or Dialogue* or triad* or Interview* or consult* or "decision making") or DE “communication” OR DE “information dissemination” OR DE “conversation”	Expanders - Apply equivalent subjects Search modes - Boolean/Phrase	Interface - EBSCOhost Research Databases Search Screen - Basic Search Database - APA PsycInfo	2,423,980
S1	(Parent* or guardian* or mother* or father* or partner or wife or wives or husband* or boyfriend* or girlfriend* or sibling* or friend* or teacher* or social worker* or carer* or "third person" or caregiver* or "care-giver*" or spouse* or chaperone*) OR DE “parents” OR DE “mothers” OR DE “fathers” OR DE “spouses” OR DE “wives” OR DE “husbands” OR DE “siblings” OR DE “significant others” OR DE “social workers” OR DE “guardianship” OR DE “caregivers”	Expanders - Apply equivalent subjects Search modes - Boolean/Phrase	Interface - EBSCOhost Research Databases Search Screen - Basic Search Database - APA PsycInfo	894,375

CINAHL (via Ebscohost)

Accessibility Information and Tips

Print Search History

Thursday, November 24, 2022 6:21:27 PM

#	Query	Limiters/Expanders	Last Run Via	Results
S7	S1 AND S2 AND S3 AND S4	Limiters - Published Date: 20050101-20221231<br>Expanders - Apply equivalent subjects<br>Narrow by Language: - english<br>Search modes - Boolean/Phrase	Interface - EBSCOhost Research Databases Search Screen - Advanced Search Database - CINAHL	1,837

S6	S1 AND S2 AND S3 AND S4	Limiters - Published Date: 20050101-20221231<br>Expanders - Apply equivalent subjects<br>Search modes - Boolean/Phrase	Interface - EBSCOhost Research Databases<br>Search Screen - Advanced Search<br>Database - CINAHL	1,866
S5	S1 AND S2 AND S3 AND S4	Expanders - Apply equivalent subjects<br>Search modes - Boolean/Phrase	Interface - EBSCOhost Research Databases<br>Search Screen - Advanced Search<br>Database - CINAHL	2,106
S4	(affect* or effect* or influenc* or resultant or impact* or perception* or perspective* or encounter* or preference or opinion or involvement or occurance* or feel or "go through" or experienc*)	Expanders - Apply equivalent subjects<br>Search modes - Boolean/Phrase	Interface - EBSCOhost Research Databases<br>Search Screen - Advanced Search<br>Database - CINAHL	3,016,184
S3	("TYA cancer" or "TYA oncology" or "AYA cancer" or "AYA oncology" or ("young adult" n3 (cancer or oncology or leuk?em* or lymphom* or h?ematol*)) or ("teenage and young adult" n3 cancer) or ("teenage and young adult" n3 oncology) or (teenage* n3 cancer) or (teenage* n3 oncology) or (adolescen* n3 cancer) or (adolescen* n3 oncology) or ("young people" n3 cancer) or ("young people" n3 oncology) or ("teenage and young adult" n3 leuk?emia*) or (teenage* n3 leuk?emia*) or (adolescen* n3 leuk?emia*) or ("young people" n3	Expanders - Apply equivalent subjects<br>Search modes - Boolean/Phrase	Interface - EBSCOhost Research Databases<br>Search Screen - Advanced Search<br>Database - CINAHL	59,927

	leuk?emia*) or ("young adult" n3 leuk?emia*) or ("teenage and young adult" n3 h?ematol*) or (teenage* n3 h?ematol*) or (adolescen* n3 h?ematol*) or ("young people" n3 h?ematol*) or ("young adult" n3 h?ematol*) or ("teenage and young adult" n3 lymphom*) or (teenage* n3 lymphom*) or (adolescen* n3 lymphom*) or ("young people" n3 lymphom*) or ("young adult" n3 lymphom*)) OR ((MH “adolescence+” OR MH “young adult+”) AND (MH “neoplasms+”))			
S2	(Communicat* or Disclos* or inform* or Interact* or relationship* or Conversation* or Dialogue* or triad* or Interview* or consult* or "decision making") or MH “communication+” OR MH “discussion” OR MH “conversation” OR (MH "Professional-Patient Relations+")	Expanders - Apply equivalent subjects Search modes - Boolean/Phrase	Interface - EBSCOhost Research Databases Search Screen - Advanced Search Database - CINAHL	2,016,086
S1	(Parent* or guardian* or mother* or father* or partner or wife or wives or husband* or boyfriend* or girlfriend* or sibling* or friend* or teacher* or social worker* or carer* or "third person" or caregiver* or "care-giver*" or spouse* or chaperone*) OR MH “parents” OR MH “mothers” OR MH “fathers” OR MH “spouses” OR MH “siblings” OR MH “teachers” OR MH “social workers” OR MH “caregivers”	Expanders - Apply equivalent subjects Search modes - Boolean/Phrase	Interface - EBSCOhost Research Databases Search Screen - Advanced Search Database - CINAHL	544,991

Web of Science Core Collection

Web of Science Search Strategy (v0.1)

Database: Web of Science Core Collection

Entitlements:

- WOS.IC: 1993 to 2022
- WOS.CCR: 1985 to 2022

- WOS.SCI: 1900 to 2022
- WOS.AHCI: 1975 to 2022
- WOS.BHCI: 2008 to 2022
- WOS.BSCI: 2008 to 2022
- WOS.ESCI: 2017 to 2022
- WOS.ISTP: 1990 to 2022
- WOS.SSCI: 1956 to 2022
- WOS.ISSHP: 1990 to 2022

Searches:

1: TS=(Parent* or guardian* or mother* or father* or partner or wife or wives or husband* or boyfriend* or girlfriend* or sibling* or friend* or teacher* or social worker* or carer* or "third person" or caregiver* or "care-giver*" or spouse* or chaperone*)
Results: 2129759

2: TS=("TYA cancer" or "TYA oncology" or "AYA cancer" or "AYA oncology" or ("young adult" near/3 (cancer or oncology or leuk?em* or lymphom* or h?ematol*)) or ("teenage and young adult" near/3 cancer) or ("teenage and young adult" near/3 oncology) or (teenage* near/3 cancer) or (teenage* near/3 oncology) or (adolescen* near/3 cancer) or (adolescen* near/3 oncology) or ("young people" near/3 cancer) or ("young people" near/3 oncology) or ("teenage and young adult" near/3 leuk?emia*) or (teenage* near/3 leuk?emia*) or (adolescen* near/3 leuk?emia*) or ("young people" near/3 leuk?emia*) or ("young adult" near/3 leuk?emia*) or ("teenage and young adult" near/3 h?ematol*) or (teenage* near/3 h?ematol*) or (adolescen* near/3 h?ematol*) or ("young people" near/3 h?ematol*) or ("young adult" near/3 h?ematol*) or ("teenage and young adult" near/3 lymphom*) or (teenage* near/3 lymphom*) or (adolescen* near/3 lymphom*) or ("young people" near/3 lymphom*) or ("young adult" near/3 lymphom*)) Results: 7793

3: TS=(Communicat* or Disclos* or inform* or Interact* or relationship* or Conversation* or Dialogue* or triad* or Interview* or consult* or "decision making")
Results: 11889093

4: TS= (affect* or effect* or influenc* or resultant or impact* or perception* or perspective* or encounter* or preference or opinion or involvement or occurance* or feel or "go through" or experienc*) Results: 24306121

5: #4 AND #3 AND #2 AND #1 Results: 684

6: #4 AND #3 AND #2 AND #1 Results: 684

7: #4 AND #3 AND #2 AND #1 and 2005 or 2006 or 2007 or 2008 or 2009 or 2010 or 2011 or 2012 or 2013 or 2014 or 2015 or 2016 or 2017 or 2018 or 2019 or 2020 or 2021 or 2022 (Publication Years) Results: 644

8: #4 AND #3 AND #2 AND #1 and 2005 or 2006 or 2007 or 2008 or 2009 or 2010 or 2011 or 2012 or 2013 or 2014 or 2015 or 2016 or 2017 or 2018 or 2019 or 2020 or 2021 or 2022 (Publication Years) and English (Languages) Results: 619
