# Supplementary material for: Triadic communication with teenagers and young adults with cancer: a systematic literature review – ‘make me feel like I’m not the third person’
Source: BMJ Open. 2024 Feb 17;14(2):e080024. doi: 10.1136/bmjopen-2023-080024 (PMC10875529; doi:10.1136/bmjopen-2023-080024)
Supplement: Supplementary data [file bmjopen-2023-080024supp002.pdf]

Table 2. Summary of Articles

First author (year)	Title	Setting	Study Type - analysis method<br>*Data collection*	Focus	Participant Characteristics	Key findings	WoE Score
Ananth (2021)	A Stakeholder-Driven Qualitative Study to Define High Quality End-Of-Life Care for Children with Cancer	USA<br>Multicentre	Qualitative - thematic analysis<br><br>*Semi-structured interviews and focus groups.*	To explore end of life care (EOLC) priorities for children with cancer and their families.	54 participants:<br>**10 AYACs (age range: 17-23 years)**<br>25 parents (including 12 bereaved parents)<br>19 healthcare professionals	Important to have direct communication with the child or young person regarding decision-making.<br>Interdisciplinary care with integrated teams is vital for high quality end of life care. Continuity of healthcare professionals was positive.<br>AYACs would prefer to die at home but family and healthcare professionals may be hesitant.	Medium
Bahrami (2017)	Information Sharing Challenges Between Adolescents with Cancer, their Parents and Health Care Providers: A Qualitative Study	Iran<br>Single centre	Qualitative descriptive-exploratory study - grounded theory analysis<br><br>*Semi-structured interviews.*	Information sharing between AYACs, parents and health professionals.	33 participants:<br>**12 AYACs (age range at interview: 15-20 years, within 1 year of diagnosis)**<br>6 supporters<br>6 healthcare professionals	AYACs feel they are excluded from information-sharing sessions between parents and healthcare professionals. This leads to disaffiliation, confusion and AYACs seek information from 'inferior' sources.<br>Parents were often the first receivers of information allowing them to act as gatekeepers controlling information to flow to AYACs. Parents may want to shield AYACs from bad news.<br>Trust and honesty are the foundations of effective communication between AYACs and healthcare professionals. AYACs reacted negatively towards dishonesty.	Medium
Barakat (2014)	A Qualitative Study of Phase III Cancer Clinical Trial Enrollment Decision Making Perspectives from AYAC, Caregivers and Providers	USA<br>Single centre	Qualitative - thematic analysis<br><br>*Semi-structured interviews.*	Clinical trial enrollment.	40 participants:<br>**13 AYACs (age range: 15-21 years)**<br>16 supporters<br>11 healthcare professionals	Four patterns of decision-making patterns identified:<br>1. AYAC abdicates to caregiver,<br>2. Caregiver based and AYAC approved,<br>3. Collaborative,<br>4. AYAC in charge of decision-making.<br>Caregivers perceived AYAC to be in charge of decision making most of the time whereas the AYACs felt that "AYAC abdicates to carer" was the most common form of decision making.<br>Distress and poor health limited AYAC involvement in the decision.<br>Developmental and emotional maturity facilitated involvement.	High

Critoph DJ, *et al. BMJ Open* 2024; 14:e080024. doi: 10.1136/bmjopen-2023-080024

Barlevy (2019)	Oncofertility decision making: findings from Israeli adolescents and parents	Israel<br>Single centre	Qualitative-thematic analysis<br><br>*semi-structured interviews*	To understand adolescent oncofertilty decision making in Israel, from perspectives of parents and adolescents	35 participants<br>**16 AYACs (age range 12-16 years)**<br>19 parents	As in other cultural contexts, Israeli adolescents and parents demonstrate multifaceted decision making with respect to oncofertility. A significant finding from this study suggests that health professional shy from discussing posthumous planning of cryopreserved materials with adolescent cancer patients and their parents. 5 out of 16 AYAs felt that the decision was not theirs and that it was instead the parents' or the pysicians' to make. Some parents felt that the decision was made by the clinician - explicit or implicit recommendations from the clinician strongly influence decision making. No decisional regret expressed by any members of the dyad.	Medium
Cicero-Oneto (2017)	Decision-making on therapeutic futility in Mexican adolescents with cancer: a qualitative study	Mexico<br>Multicentre	Qualitative - thematic analysis<br><br>*Semi-structured interviews*	Decision making on therapeutic futility	32 Participants<br>13 paediatric oncologists<br>13 parents or primary carers<br>**6 AYACs (age range 13-18 years)**	Four themes were identified<br>1. flow of information to inform decision making<br>2. disclosure of prognosis<br>3. decision maker and stakeholder involved in decision making<br>4. barriers and facilitators to decision making<br>Differing values and agendas. The parents valued messages to "life the spirits" whereas the AYACs values honesty from the healthcare professionals<br>Gatekeeping of information.<br>Theme of "deference to authority"	Medium

Darabos (2021)	Cancer Related Decision Making Among AYAC, Care Givers and Oncology Providers	USA<br>Single centre	Qualitative – content analysis with hypothesis coding<br><br>*Semi-structured interviews.*	Decision-making and triadic communication	30 Participants:<br>**11 AYACs (age range: 15-24 years)**<br>11 supporters<br>8 healthcare professionals	Four decision-making patterns were identified:<br>1. AYAC driven,<br>2. Collaborative,<br>3. Deferral to parents,<br>4. Provider-based, AYA/Caregiver-approved.<br>Collaborative decision-making and AYAC-driven decisions were most commonly described. There was recognition that some decision-making was day/context dependent.<br>AYACs were more likely to drive decisions regarding supportive care than treatment related decisions.<br>AYACs and caregivers explained how cognitive and emotional processes influenced cancer related decisions.<br>Emotional coping was more common than problem-based coping<br>Direct and honest communication contributes to a stronger relationship.<br>Individuality is key along with flexibility.<br>Time alone is important.	High
Davies (2019)	'Life then', 'life interrupted', 'life reclaimed': the fluctuation of agency in teenagers and young adults with cancer	UK<br>single centre	qualitative - thematic analysis<br><br>*case studies - multiple interviews*	Fluctuation of agency across time and between cases	22 participants<br>**5 AYACs (16-24 years)**<br>**5 parents carers** (2 fathers, 3 mothers, 1 couple),<br>5 healthcare professionals (4 nurses and 1 oncology consultant)<br>5 other supporters (1 boyfriend, 1 girlfriend, 1 aunt, 2 friends)	Agency fluctuates over time within cases and between cases. Agency can fluctuate between personal, proxy and collective perspectives. Personal agency is high prior to diagnosis, decreases after diagnosis and is reclaimed after treatment.	Medium
Ellis (2016)	Fertility concerns among child and adolescent survivors and parents: a qualitative study	Australia<br>single centre	Qualitative semi-structured telephone interviews	Fertility related themes with AYACs who are recently off treatment and with their parents	97 participants from 45 families<br>**19 AYACs (age range 7-17 at diagnosis, mean age 13.3)**<br>44 mothers and 34 fathers	Both parents and AYACs are concerned about the potential impacts on fertility of treatment. Poor doctor-patient communication was reported and conversations about fertility were frequently interrupted to discuss illness and treatment. These fertility discussions were not then continues once the AYAC was off treatment	Medium

Essig (2016)	Improving Communication in Adolescent Cancer Care: A Multiperspective Study	Germany, Austria, Switzerland<br><br>Number of centres not stated	Qualitative – <u>inductive thematic analysis</u><br><br>*Focus groups.*	Explore effective communication with AYACs for communication skills training.	<u>54 participants:</u><br>**16 AYACs (age range: 13-19 years)**<br>8 parents<br>30 healthcare professionals	Decision-making can cause conflict when adolescents are cognitively mature but legally lack the ability to make decisions.<br>AYACs feel a loss of autonomy.<br>Age-appropriate environments are important.<br>Effective communication differs depending on the type of professional (i.e., doctor vs nurse)<br>Adolescents negatively affect communication when:<br>1. They are indifferent.<br>2. There priorities conflict with treatment<br>3. They conflict with parents.<br>Healthcare professionals negatively affect communication when:<br>1. They don't treat the adolescent in an age-appropriate way.<br>2. They don't take the adolescent seriously.<br>3. They give too much information or withhold important information	Medium
Fern (2013)	The Art of Age-Appropriate Care	UK<br>Number of centres not stated	Qualitative – <u>thematic analysis</u><br><br>*Peer-to-peer interviews, field notes and spider diagrams from focus groups.*	Review a conceptual model of AYACs' cancer care experiences.	<u>11 participants:</u><br>**11 AYACs (age range: 13-25 years)**	Young people must be kept at the centre of interactions in recognition of their stated needs:<br>1. Engagement.<br>2. Individually tailored information.<br>3. Support unproxied by parents/family.<br>AYACs did not want information to be directed at parents but at them.<br>AYACs found it embarrassing when sensitive information was revealed in the presence of their parents.<br>Lack of continuity of healthcare professionals leads to AYACs dissatisfaction and irritation having to repeat their cancer story.	Medium

Critoph DJ, *et al. BMJ Open* 2024; 14:e080024. doi: 10.1136/bmjopen-2023-080024

Fourneret (2018)	Breaking bad news about cancer to adolescents and young adults: the french experience	France Multicentre	qualitative semi-structured interviews	Explore the effectiveness and implementation of the French announcement protocol in 7 french paediatric oncology centres	<u>90 participants</u><br>**27 AYACs (21 were 14-17 and 6 were 18-22)**<br>30 parents (16 mothers, 5 fathers, 9 parents together at the appointment)<br>33 healthcare professionals	Parents and AYACs have different needs - both of which need to be accounted for when breaking bad news. Awkward and premature announcements were noted<br>The announcement consultation – young patients were never alone when informed of their disease; either with parents (n=31) (parents were informed before their child 10 out of 31 times - this was motivated by a compassionate goal of preparing the parents so they can better support the child when the bad news is broken) or close family member/sibling or boyfriend or girlfriend (n=2).<br>Some parents withheld info and some AYACs preferred parents not to know their diagnosis<br>Asymmetry in the triad discussed - but the key quality needed in the triad is mutual trust<br>HCP found parental presence helpful in the study. HCPs should show empathy (no neutrality) and attention to detail	Medium
Frederick (2018)	Adolescent Patient Involvement in Discussions About Relapsed or Refractory Cancer with Oncology Clinicians.	USA Single centre	Qualitative – <u>content analysis</u><br><br>*Audiotaped conversations.*	Breaking bad news of relapsed or refractory cancer.	<u>75 participants:</u><br>**11 AYACs (age range: 12.6-17.5 years)**<br>44 supporters<br>20 healthcare professionals	Adolescent patients' involvement in conversations about relapsed or refractory cancer is limited.<br>Adolescents were accompanied by one (27%) two (64%) or more than two (18%) family members in the discussion.<br>Adolescents spoke 3.5% of words compared to 66.9% clinicians and 30% parents.<br>No conversations included instances in which the clinicians' asked adolescents for their communication preferences or desired role in decision-making.	High
Friebert (2020)	Congruence gaps between adolescents with cancer and their families regarding values, goals and beliefs about end-of-life care	USA multicentre	*Qualitative cross-sectional study*	End of life care	<u>126 participants</u>:<br>**126 parent-AYAC dyads AYACs (14-20 years, mean age 16.9)**	Young people wanted early information (86%) but only 39% families knew this. Families understanding of what was important to their adolescents when dealing with their own dying was excellent for wanting honest answers from their physician and understanding treatment choices but poor for dying a natural death and being off machines that extend life, if dying.<br>Parents do not know what AYACs want at the end of life	Low

Glackin (2023)	Experiences of Oncofertility Decision-Making and Care in a National Sample of Adolescent and Young Adult Cancer Patients and Parents	Australasia multicentre	Qualitative – cross sectional survey. Reflexive thematic analysis	Oncofertility decision making	210 participants:<br>**99 AYACs (age range 15-25 years)**<br>111 parents<br>41 AYAC parent dyads from the same family	Four themes were identified: emotional care needs; parent-AYA dynamics including autonomy and agendcy; decision-making considerations including values and practicalities; and reflections on oncofertility and follow-up.<br>Both AYAC and parents placed importanceon AYA autonomy in fertility decision-making but, but many AYAs appreciated the role of parents in providing support and guidance throughout the process.<br>Healthcare professionals are encouraged to autonomously engage AYA's around fertility decision making, while concurrently offering opportunities that promotes parental support.	Low
Hart (2020)	The Challenges of Making Informed Decisions About Treatment and Trial Participation Following Cancer: A Qualitative Study with Adolescent and Young Adults with Cancer and Care Givers	UK Multicentre	Qualitative – thematic analysis<br><br>*Semi-structured interviews.*	Shared decision-making – primary treatment and trial participation – at diagnosis.	33 participants:<br>**18 AYACs (age range: 16-24 years)**<br>15 supporters	AYACs struggled to process information around diagnosis, exacerbated by symptom burden, emotions, and the fast pace of clinical activity.<br>Some AYACs disengaged from conversation topics which were distressing.<br>There are limited options for 'real' decision-making at diagnosis. However, many preferred this when they were already overwhelmed by emotions/symptoms.<br>For trial enrollment, many AYACs allowed themselves to be steered by the recommendation of the healthcare professional who recruited them, thinking they were acting in their best interests.	Medium
Hong (2016)	Care Partnerships: toward technology to support teen's participation in their health care	US multicentre	Qualitative semi-structured interviews and observations	To investigate how technology can support the partnerships between AYACs, parents and clinicians when the AYAC is experiencing complex chronic illness	33 interviews.<br>**15 with AYACs (13 of whom had cancer. age range 13-17)**<br>15 parents (10 mothers, 1 fathers, 1 aunt and 2 fathers and mothers together)<br>8 clinician caregivers	Participants faced challenges concerning:<br>1) Teens' limited participation in their care<br>2) communicating emotionally sensitive information<br>3) managing physical and emotional responses<br>Time alone with clinicians was important. Mutual protectionism or the need to "emotionally protect eachother" was prevalent.	Medium

Ingersgaard (2018)	A qualitative study on decision-making on Phase III randomized clinical trial participation in paediatric oncology: adolescents' and parents' perspectives and preferences	Denmark	qualitative exploratory study - in-depth semi-structured interviews with thematic analysis	To explore patients' and AYACs' motivs for accepting/ declining participation in the AL2008 trial and adolescents' involvement in decision making	16 participants<br>**5 AYACs (age range 12-16)**<br>6 parents of AYACs<br>5 parents of children aged 3-10 years with cancer	Key themes<br>1) altruism - wanting to help future AYACs<br>2) trust in the clinicians<br>3) individuals perceptions of cure contra toxicity<br>4) adolescents as active participants in the decision making process<br>5) parental responsibility and authority<br>6) the difficulty of uncertainty	Low
Jacobs (2015)	Adolescent end of life preferences and congruence with their parents' preferences: results of a survey of adolescents with cancer	Norway	Qualitative<br><br>three sessions of dyadic interviews	To explore AYACs' end of life preferences and to assess the congruence of these preferences with the parents' beliefs	17 adolescent/ family dyads<br>**17 AYACs (age range 14-21, 71% under 18)**	Adolescents with cancer were comfortable discussing EOL, and the majority preferred to talk about EOL issues before they are facing EOL. There were substantive areas of agreement between adolescents and their surrogates, but important facets of adolescents' EOL wishes were not known by their families, reinforcing the importance of eliciting individual preferences and engaging dyads so parents can understand their children's wishes. 53% of AYACs had never spoken about their end of life preferences but 82% considered it important to let their loved ones know their wishes.	Low

Critoph DJ, *et al. BMJ Open* 2024; 14:e080024. doi: 10.1136/bmjopen-2023-080024

Korsvold (2017)	A content analysis of emotional concerns expressed at the time of receiving a cancer diagnosis: An observational study of consultations with adolescent and young adult patients and their family members	Norway	exploratory mixed methods study<br><br>audio recorded consultations	To investigate the emotional concerns of AYACs at the time of diagnosis and how to quantify how healthcare professionals respond	18 participants<br>**9 AYACs (age range 13-23)**<br>Present with mother (n=9), father (n=1), sister (n=1) or mother and father (n=2)	Four major themes of emotional concerns expressed by AYA patients and their family members during consultations for a cancer diagnosis:<br>1) side effects/late effects or infertility,<br>2) "what happens in the near future/practical aspects",<br>3) fear<br>4) sadness<br>AYA patients and family members expressed emotional concerns. HCPs typically responded by providing information, rather than affective aspects of the concerns In the sadness theme however, an explicit provide space affective response was the most common response (n=8) followed by an explicit provide space content response (n=7)<br>To make patients 'feel known' HCP should pay attention to the affective aspect of the expressed concern.	Medium
Lyon (2013)	Family-Centered Advance Care Planning for Teens With Cancer	USA<br>single centre	Qualitative<br>a randomised control pilot study	To examine the efficacy of family-centres ACP	30 dyads<br>**mean age of AYACs 16**<br>17 were randomised to intervention and 13 were randomised to control<br>87% of surrogates were biological parents and were female	The model (ACP) increased congruence in the triad compared to the control standard of care group– so it is key. The family centres ACP AYACs reported feeling more informed that the control group	Low

Mack (2019)	Adolescent and Young Adult Cancer Patients' Experiences with Treatment Decision-Making	USA<br>Single Centre	Quantitative – <u>multivariate analysis, logistic regression</u><br><br>*Surveys at diagnosis, 4 and 12 months.*	Treatment decision-making	<u>203 participants:</u><br>**203 AYACs (age range: 15-29 years)**	A majority of AYACs (58%) want to share decision-making with oncologists. The remainder were split between the AYAC wanting primary responsibility in decision-making (20%) or wanting their oncologist to have primary responsibility (22%).<br>A lower proportion of younger AYACs wanted sole responsibility but this did not achieve statistical significance (P = 0.07).<br>The majority (90%) of AYACs who lived with a parent/guardian wanted some form of input from their parents (either collaborative or considering their opinion).<br>Younger AYACs (15-17 years) were more likely to want greater involvement by their parents but were also more likely to be less involved than they wanted to be relative to their parents.<br>Decisional regret was less likely among AYACs who trusted oncologists completely, and who reported that oncologists understood what was important to them when treatment started.	Medium
Mobley (2023)	Clinical Trial Participation: A qualitative study of Adolescents and Younger Adults Recently Diagnosed with Cancer	USA	<u>Qualitative</u><br>Grounded theory analysis of semi-structured interviews	Clinical trial participation	<u>9 participants:</u><br>**9 AYACs (age range 16-20)**	Consent encompassed the first discussion of CCT. Patients reflected positive and negative effects of timing, decisional role, and emotional impact.<br>Informing participation involved decision-making processes, specific knowledge, understanding and external influence.<br>Participant relationships emphasized the importance of communication and relationships with providers and parents. Patient determinants centered on motives from different perspectives, pre-conceived attitudes, and understanding of CCTs.	Medium

Critoph DJ, *et al. BMJ Open* 2024; 14:e080024. doi: 10.1136/bmjopen-2023-080024

Olsavsky (2021)	Family communication about fertility preservation in adolescent males newly diagnosed with cancer	USA	Qualitative	To explore fertility preservation communication among mothers, fathers and their male adolescents newly diagnosed with cancer.	87 participants:<br>**33 AYAC aged 12-25**<br>32 mothers<br>22 fathers<br><br>Representing 37 families in total.	Five process themes:<br>(1) Reliance on health care team and social support networks to facilitate FP decisions (noted just by parents),<br>(2) withholding parental opinion and deferring the decision to the adolescent,<br>(3) ease of communication,<br>(4) communication barriers and facilitators,<br>(5) not being present or not remembering details of FP conversations.<br>Four content themes:<br>(1) preference for biological parenthood (or grandparenthood),<br>(2) consideration of future partner of AYAC's desire for biological parenthood,<br>(3) sperm banking whilst it is a viable option,<br>(4) openness to alternative parenthood options	Medium

Patterson (2012)	The Unmet Needs of Emerging Adults With a Cancer Diagnosis	Australasia	Qualitative	Aim to contribute to the limited research base and inform our understanding of the needs of emerging adults with a diagnosis of cancer from a developmental perspective that appreciates the key transitional tasks of emerging adulthood identified by Arnett	<u>14 Participants:</u><br>**14 AYAC aged 20-25**, average age of 22	A cancer experience poses the potential for significant impact on the four requirements for achievement of adulthood.<br><br>The needs of these emerging adults were grouped into six themes; information, healthcare provision, daily living, interpersonal support, identity renegotiation and emotional distress.<br><br>These themes relate directly to the four requirements of adulthood.<br>1. The task of accepting responsibility for oneself emphasises the importance of empowering AYAC in their communication with HCP.<br>2. The task of deciding on personal beliefs and values highlights the importance of keeping the AYAC informed and encouraging them in decisions giving maximum opportunity to explore beliefs.<br>3. Establishing a relationship with parents as equals highlights the importance of maximising AYAC autonomy in relation to supporters.<br>4. The task of becoming financially independent highlights the importance of minimising disruption to daily life.	Low

Pennant (2020)	The Role of Social Support in Adolescent/Young Adults Coping with Cancer Treatment	USA	Qualitative	To explore specific actions that help AYAC and what behaviours they want from their social supports	20 Participants:<br>**10 AYAC ages 15-26**, mean age 18.9 years<br>10 parents	Themes of support included; presence, distraction, positive attitude, maintaining AYAC autonomy, communication and advocacy.<br>Mothers were the most noted family support.<br>AYAC patients can differ in their preferences throughout treatment and this can, at times, appear contradictory.<br>AYACs appear to want autonomy and independence, but appreciate help with daily tasks from their parents.<br>They express the desire for privacy, but also value physical presence and communication<br>Parents must oscillate between being involved in and catering to their AYAC child's needs during treatment while allowing space for independence and autonomy.<br>The findings underscore the importance of maintaining open communication with AYAC patients about their preferences and needs throughout the course of treatment and asking them about both individual and social preferences, which may change frequently.	Medium
Pyke-Grimm (2020)	3 Dimensions of Treatment Decision Making in Adolescents and Young Adults with Cancer.	USA Multicentre	Qualitative – ethnographic<br><br>*Semi-structured interviews, field notes.*	Explore the preferences of AYACs for involvement in healthcare decisions	16 participants:<br>**16 AYACs (age range: 14.7-20 years)**	Emotions around diagnosis inhibit information receptiveness and ability to engage in treatment decisions (especially important decisions).<br>Initially AYACs struggle with the jargon and plethora of medical terms which are being used. They have limited knowledge which limits their questions, this increases over time.<br>The importance of decisions differs from one AYACs to the next. Also, some decisions are seen as having only one 'real' option.<br>AYACs engaged in minor decisions much earlier in their treatment, and some began engaging in more important decisions later in treatment.<br>AYACs could adopt an active (sole decision maker), collaborative (with healthcare professionals/supporters) or passive (healthcare professionals/supporters as decision makers) role.	Medium

Pyke-Grimm (2022)	Day-to-Day Decision Making by Adolescents and Young Adults with Cancer	USA	Qualitative Interpretive focused ethnography within the socio-logic tradition, informed by symbolic interactionism	To explore involvement of AYAs with cancer in day-to-day decisions affected by their cancer and treatment.	<u>16 Participants:</u><br>**16 AYAC aged 15-20 (at time of interview - with an average of one year from diagnosis)**	Factors influence the involvement of AYAC in decision making such as the type of decision, the point in the cancer journey. They want to be involved.<br>Four day to day decision making categories were identified: mental mindset, self care practices, self-advocacy and negotiating relationships.<br>Parents were often present and staying strong was a recurring theme across mental mindset and negotiating relationships.<br>HCP are critical to facilitate AYAC participation in day to day decision making by encouraging autonomy and with effective communication.	Medium
Sawyer (2019)	Developmentally Appropriate Care for Adolescents and Young Adults with Cancer: How Well is Australia Doing?	Australasia Multicentre	Quantitative – <u>Chi-squared and Fisher's exact test</u><br><br>*Single time point survey.*	Explore quality of AYAC care in Australia.	<u>196 participants:</u><br>**196 AYACs (age range: 15-25 years)**	>90% of AYACs reported positive responses for 11 of the 14 experience of care items which related to the quality of communication and general interactions with the cancer care team.<br>The most highly endorsed of these experiences of care items related to staff being friendly and respectful, communicating in ways that the AYAC understood, being supportive of AYACs asking questions and engaging families in discussion and decisions as the AYAC wished.<br>Older AYAC (20-25 years) report more empowerment to make decisions than younger AYACs and were more likely to report that healthcare professionals included their family in discussions and decision-making the way they wanted them to be included.	Low

Sisk (2022)	Interdependent Functions of Communication with Adolescents and Young Adults in Oncology	USA Multicentre	Qualitative – <u>content analysis</u> *Semi-structured interviews.*	Define communication functions from perspective of AYACs.	<u>37 participants:</u> **37 AYACs (age range: 12-20 years; mean: 16 years)**	Building relationships: demonstrating clinical competence, reliability, empathy, and showing care and concern. When clinicians demonstrated these attributes, AYACs described feelings of trust in the clinicians' ability and intent to care for them.<br>Exchanging information: providing accurate and transparent information that was adapted to AYACs' needs. These needs related to the amount, complexity, timing, and pacing of information, and balancing communication between parents and AYACs.<br>AYACs think honesty and transparency is important. However, transparency could be burdensome.<br>Exploring uncertainties and fears of the future mad AYACs feel better prepared and decreasing anxiety. There was variation between AYACs for exploring these unknowns.<br>AYACs varied in their preferences in sharing distressing information and whether healthcare professionals should remain present and or give AYACs their privacy.<br>AYACs often feel that treatment related decisions realistically only have one choice giving a sense of powerlessness. They played a greater role in decisions outside of treatment related areas.<br>While some AYACs preferred very passive or active roles most described an interdependent process of communication involving them, their parents, and their clinicians.<br>Parents often served as a conduit and buffer of communication between the AYAC and healthcare professional. Many described the integral role of parents in communication regardless of their age.	High

Sisk (2022)	Co-management of communication and care in adolescent and young adult oncology	USA	Qualitative Semi-structured interviews	Study aimed to learn how AYAs and parents shared and delegated roles in communication and care during and after treatment for cancer	37 Participants:<br>**37 AYAC aged 12-24**<br>**Mean age 16 years**	There are 6 roles that AYAC co-manage with parents; managing information, managing social and emotional needs, managing health, advocacy and empowerment, making decisions and managing logistics.<br>Five factors that influence AYAC roles in communication were:<br>AYAC agency<br>Clinical encouragement<br>Emotional and physical well-being<br>Personality, preferences and values<br>Insights and skills<br><br>There are multiple benefits of engagement of the adolescent.	Medium
Son (2023)	Family Communication About Cancer in Korea: A Dyadic Analysis of Parent-Adolescent Conversation	Korea	Qualitative descriptive study	The aim was to understand communication experiences of Korean AYAC and their parents in the context of young adult cancer.	14 participants:<br>**7 AYAC (ages 14-19 years)** and 7 parent pairs	The main theme was "experience the same thing but see it differently" along with three subthemes.<br>Different expectations for parent-adolescent communication, different views on communication challenges and limited sharing and progress in the conversation.<br>This study offers insights into different communication expectations and preferences between Korean adolescents and parents, and reasons for communication challenges, while emphasizing the individualized assessment of parent-adolescent communication between them.	Medium

Viola (2018)	Problem-solving skills, parent–adolescent communication, dyadic functioning, and distress among adolescents with cancer	USA	Mixed methods study	The aim was to describe and assess how intrapersonal (i.e., problem-solving ability) and social–ecological factors (i.e., cancer- related communication with parents and parent–adolescent dyadic relationship quality) are associated with adolescent adjustment (i.e., distress).	78 participants:<br>39 AYAC and 39 parent pairs<br>**AYAC 14-20 mean age 16.1**<br>39 Parents - 79.5 % mothers	Better adolescent problem solving skills and better parent problem solving skills were associated with lower adolescent distress.<br>Parents and adolescents reported similar moderate levels of cancer related communication problems.<br>The most commonly endorsed cancer-related problem was “not talking about what to do if the AYAC got significantly worse’.<br>Parents reported better problem solving ability and better dyadic functioning than their adolescent.	Low
Weaver (2016)	“Being a Good Patient” During Times of Illness as Defined by Adolescent Patients With Cancer	USA	Qualitative - semantic content analysis<br><br>Semi-structured interviews		40 participants<br>**AYAC ages 12-19**<br>Mean age of 15.5 years	The concepts of adherence and compliance were the primary phrases used to describe the good patient role, but always within the context of a relationship. Of note: A total of 23 adolescents requested to be interviewed alone with the interviewer (57.5%)	Medium
Weaver (2015)	Adolescents’ Preferences for Treatment Decisional Involvement During Their Cancer	USA Multicentre	Qualitative – semantic content analysis<br><br>*Semi-structured interviews.*	Investigate AYACs’ decision-making preferences and how supports and healthcare professionals can support involvement.	40 participants:<br>**40 AYACs (age range at interview: 12-18.9 years; 0.5-6 months from diagnosis/relapse)**<br><br>*NB: 34 AYACs primary diagnosis, 6 AYACs relapse.*	AYACs indicated a spectrum of preferred decisional roles, with the most common being an actively involved role (65%), although a shared decision-making approach was still valued.<br>AYACs recognized that situational and social contexts might shift their preferred level of involvement in medical decisions.<br>Although adolescents wanted to be involved in decisions, they also expressed an appreciation of family insight, parental presence, and clinician guidance.<br>AYACs can retrospectively identify their preferences for inclusion in medical decision-making, and even when preferring involvement, they value the input of trusted others.	Medium

Critoph DJ, *et al. BMJ Open* 2024; 14:e080024. doi: 10.1136/bmjopen-2023-080024

Wu (2021)	Decisional conflicts, anxiety, and perceptions of shared decision-making in cancer treatment trajectory among adolescents with cancer: A longitudinal study	Taiwan	Qualitative. An explanatory mixed method was used, incorporating questionnaires and individual interviews.	To describe the perception on levels of decision-making during cancer treatment for adolescents with cancer and examine the trajectory of their decisional conflict	<u>44 participants:</u><br>**22 AYAC** 11 male and 11 female<br>mean age 15.39<br>**22 Supporters**:<br>father n=1<br>mothers n=12<br>both n=6<br>other n=3	Different levels of participation in shared decision making (SDM) during the treatment trajectory were found.<br>Participants experienced the highest decisional conflict during diagnosis.<br>Roles in healthcare communication varied from direct participation to indirect involvement.<br>Overall, participants reported that doctors and parents decided their level of involvement, communication and or decision making.	Medium
Zarnegar et al (2018)	Recall of Fertility Discussion by Adolescent Female Cancer Patients: A Survey-Based Pilot Study	USA	Qualitative	To assess: recall of a fertility discussion, satisfaction with fertility knowledge, and identify factors that may influence recall.	<u>19 participants:</u><br>**19 AYAC aged** 13-18 years and a mean age of 15.6	42% and 52% of AYAC did not recall discussion regarding treatment related infertility or fertility preservation during initial treatment planning.<br>63% of AYAC reported that parents made all or most of the decisions whereas 30.8% reported making decisions with parents.<br>Key Finding - A greater percentage of AYAC who reported making a joint decision with parents recalled 71% of fertility discussions than those who reported parents made most or all of medical decisions.	Medium

¥Age range at diagnosis is given except where this was not provided in the article in which alternative metrics are presented and this is highlighted.
AYA(C) = adolescent and young adult (with cancer); NOS = not otherwise specified; RM-ANOVA = repeated measures analysis of variance.

Critoph DJ, *et al. BMJ Open* 2024; 14:e080024. doi: 10.1136/bmjopen-2023-080024
