# Supplementary material for: Triadic communication with teenagers and young adults with cancer: a systematic literature review – ‘make me feel like I’m not the third person’
Source: BMJ Open. 2024 Feb 17;14(2):e080024. doi: 10.1136/bmjopen-2023-080024 (PMC10875529; doi:10.1136/bmjopen-2023-080024)
Supplement: Author's manuscript [file bmjopen-2023-080024.draft_revisions.pdf]

BMJ Open is committed to open peer review. As part of this commitment we make the peer review history of every article we publish publicly available.

When an article is published we post the peer reviewers' comments and the authors' responses online. We also post the versions of the paper that were used during peer review. These are the versions that the peer review comments apply to.

The versions of the paper that follow are the versions that were submitted during the peer review process. They are not the versions of record or the final published versions. They should not be cited or distributed as the published version of this manuscript.

BMJ Open is an open access journal and the full, final, typeset and author-corrected version of record of the manuscript is available on our site with no access controls, subscription charges or pay-per-view fees (http://bmjopen.bmj.com).

If you have any questions on BMJ Open's open peer review process please email info.bmjopen@bmj.com

BMJ Open

Triadic communication with teenagers and young adults with cancer: a systematic literature review: "Make me feel like I'm not the third person"

Journal:	*BMJ Open*
Manuscript ID	bmjopen-2023-080024
Article Type:	Original research
Date Submitted by the Author:	18-Sep-2023
Complete List of Authors:	Critoph, Deborah; University of Cambridge, Department of Public Health & Primary Care<br>Taylor, Rachel; University College London Hospitals NHS Foundation Trust, CNMAR;<br>Spathis, Anna; University of Cambridge<br>Duschinsky, Robbie; University of Cambridge<br>Clyne, Ella; University of Cambridge<br>Hatcher, Helen; Cambridge University Hospitals NHS Foundation Trust<br>Smith, Luke; Cambridge University Hospitals NHS Foundation Trust
Keywords:	Adolescents < Adolescent, Decision Making, EDUCATION & TRAINING (see Medical Education & Training), ONCOLOGY

SCHOLARONE™
Manuscripts

I, the Submitting Author has the right to grant and does grant on behalf of all authors of the Work (as defined in the below author licence), an exclusive licence and/or a non-exclusive licence for contributions from authors who are: i) UK Crown employees; ii) where BMJ has agreed a CC-BY licence shall apply, and/or iii) in accordance with the terms applicable for US Federal Government officers or employees acting as part of their official duties; on a worldwide, perpetual, irrevocable, royalty-free basis to BMJ Publishing Group Ltd ("BMJ") its licensees and where the relevant Journal is co-owned by BMJ to the co-owners of the Journal, to publish the Work in this journal and any other BMJ products and to exploit all rights, as set out in our licence.

The Submitting Author accepts and understands that any supply made under these terms is made by BMJ to the Submitting Author unless you are acting as an employee on behalf of your employer or a postgraduate student of an affiliated institution which is paying any applicable article publishing charge ("APC") for Open Access articles. Where the Submitting Author wishes to make the Work available on an Open Access basis (and intends to pay the relevant APC), the terms of reuse of such Open Access shall be governed by a Creative Commons licence – details of these licences and which Creative Commons licence will apply to this Work are set out in our licence referred to above.

Other than as permitted in any relevant BMJ Author's Self Archiving Policies, I confirm this Work has not been accepted for publication elsewhere, is not being considered for publication elsewhere and does not duplicate material already published. I confirm all authors consent to publication of this Work and authorise the granting of this licence.

For peer review only

Triadic communication with teenagers and young adults with cancer: a systematic literature review: "Make me feel like I'm not the third person"

Deborah J Critoph MSc, Rachel M Taylor PhD, Anna Spathis MD, Robbie Duschinsky PhD, Helen Hatcher MD PhD, Ella Clyne, Isla Kuhn MSc, Luke AM Smith

Deborah Critoph, PhD student, Department of Public Health & Primary Care, University of Cambridge

Rachel Taylor, Director of the CNMAR, Centre for Nurse, Midwife and Allied Health Profession Led Research, University College London Hospitals NHS Foundation Trust, London, NW1 2PG

Anna Spathis, Assistant Professor, Department of Public Health and Primary Care, University of Cambridge

Robbie Duschinsky, Professor of Social Science & Health, Department of Public Health & Primary Care, University of Cambridge

Helen Hatcher, Consultant in Medical and TYA Oncology, Clinical and Regional Lead for Sarcoma and Young People with Cancer, Clinical Director EoE TYAc ODN

Ella Clyne, Medical Student, University of Cambridge

Isla Kuhn, Head of Medical Library Services, University of Cambridge Medical Library

Luke Smith, Junior Clinical Fellow in TYA Haematology and Oncology, Cambridge University Hospitals NHS Foundation Trust

Correspondence: Deborah Critoph, dc625@medschl.cam.ac.uk

Keywords: Teenagers and young adults with cancer (TYAC), adolescents, triadic communication, communication, supporters, third person, parents, support network, health care, professionals, experiences, impact.

Abstract = 299/300

Word count = 3928

Number of tables = 4

Number of figures = 1

For peer review only - http://bmjopen.bmj.com/site/about/guidelines.xhtml

Abstract

Background

Although most young people have limited encounters with healthcare, around 2,500 young people in the United Kingdom (UK) are diagnosed with cancer each year. Clinical communication needs of teenagers and young adults with cancer (TYAC) are increasingly recognised to differ significantly from younger children and older adults. Triadic communication refers to the presence of a third party, such as a parent, carer, or companion in clinical encounters and is a key feature of TYAC care.

Aim

We sought to understand who is present with TYACs, synthesise TYACs experiences of triadic communication with HCPs and supporter(s), and explore the impact of triadic communication for TYACs. We generated three research questions to focus this review:

Review questions:

1. Who is present with TYACs in healthcare consultations/communication? For example, who are the supporters?
2. What are TYACs' experiences of communication with the supporter present?
3. What is the impact of a TYAC's supporter being present in the communication?

Methods

We conducted a systematic review and narrative synthesis of empirical evidence published since 2005. An inductive thematic analysis was undertaken to identify the main, recurrent, and important data across the studies in answering each research question.

Results

A total of 7,727 studies were identified in the search, of which 33 fulfilled the inclusion criteria. We found that mothers were the most common supporter in clinical communication encounters. The experience of communication in the presence of a third person is paradoxical in nature – the supporter can help or hinder the involvement of the young person in their care. Overall, young people are not included in communication and decisions about their care to the level they want.

Conclusion

Triadic communication in TYAC is common, complex, and dynamic. Due to the degree of challenge and nuances raised, HCPs need further training on effective triadic communication.

For peer review only - http://bmjopen.bmj.com/site/about/guidelines.xhtml

PROSPERO registration CRD42022374528

Strengths & limitations of this study

- We searched systematically and thoroughly for eligible studies, but this is not a well-indexed field of research, and therefore it is possible that some relevant studies were not included in the review.
- We limited the review to a UK TYAC age range and not the broader age used elsewhere, so the conclusions are applicable to younger adults, up to aged 24 only and not necessarily the age of young adulthood used in some countries (between 29 to 39).
- We only included papers published in English and the results may not be applicable to other countries especially where cultural differences affect parental-TYAC or other familial/romantic relational dynamics.
- International representation was seen in the eligible studies and TYAC ages were included across the entirety of the specified UK age range.
- Studies represented the journey throughout the cancer experience from diagnosis to survivorship and end of life care.

Introduction

Adolescence is a time of transition where young people navigate monumental physical, cognitive, emotional, and behavioural milestones to develop a sense of self-identity and gain independence. Although most young people have limited encounters with healthcare, around 2,500 young people in the United Kingdom (UK) are diagnosed with cancer each year, which is the leading cause of non-traumatic death in young people in the United States (US) and Europe.(1) Teenagers and young adults with cancer (TYACs) have unique healthcare needs and there has been an international drive to develop developmentally appropriate evidence-based specialist care, provided by appropriately trained healthcare professionals (HCPs).(2)

Communication with TYACs can be particularly challenging: a life-limiting condition intersects an age associated with emotional reactivity and variable maturity. TYACs clinical communication needs are increasingly recognised to differ significantly from younger children and older adults. Research indicates TYACs can have little meaningful involvement in conversations with HCPs: almost half of children and young people reported not being involved in decisions about their care.(3) HCPs recognise this and consider young people amongst the hardest patients to communicate with.(4) However, HCPs receive little training about how best to manage these clinical encounters. TYACs who are not heard or understood can be labelled as 'challenging', 'hard to reach' and 'disengaged'. This may adversely impact care and contribute to poor physical and psychological outcomes. Despite these issues, there are limited opportunities for formal postgraduate education in communication with TYACs for HCPs, with most training being ad hoc and not interprofessional.(5,6) Effective communication with TYACs has been recognised as a key national research priority. In a UK-wide survey of young patients' own research priorities, communication was a striking cross-cutting theme.(7)

For peer review only

Recent research into clinical communication with TYACs has offered some insight into the complexities of communication with this specialist patient group.(8–12) Yet one area that has received less attention is triadic communication. Triadic communication refers to the presence of a third party, such as a parent, carer, or companion in clinical encounters (13) and the presence of such a person was found to occur in 87% of TYAC consultations.(11) As a commonly occurring form of communication in TYAC care, there is a need to understand the theoretical basis and relevance of triadic communication to clinical practice. For the purposes of this review, we refer to this third person as a supporter. Triadic communication literature from children and older adults exists. (14–17) Notably this includes a meta-analytic review of provider-patient-companion of adults,(18) one large systematic review of physician-patient-companion communication and decision-making in adults (19) and one review of doctor-parent-child communication.(20) Whilst informative, these studies are with children and adults, not this unique age-group of emerging adulthood with a significant life threatening diagnosis such as cancer. Also, these studies focus on doctor-patient-third person communication, whereas TYAC care involves a range of interdisciplinary professionals. This review aims to understand what is known about triadic communication with TYACs in healthcare communication.

Aim

We sought to understand who is present with TYACs, synthesise TYACs experiences of triadic communication with HCPs and supporter(s), and develop insights into the impact of triadic communication for TYACs.

Review questions:

1. Who is the supporter present with TYACs in healthcare consultations and communication?
2. What are TYACs' experiences of communication with the supporter present?
3. What is the impact on a TYAC's supporter being present in the communication?

Methods

We conducted a systematic review and narrative synthesis (21,22) of empirical evidence published since 2005, the year of publication of the National Institute for Care Excellence (NICE) Improving Outcomes Guidance, the guidance document underpinning TYAC services in England.(2) The review protocol was prospectively registered with PROSPERO (CRD42022374528). We designed the search to identify and map the available evidence using a broad scope to gain an overview of the pertinent literature, identify knowledge gaps and clarify concepts. The search strategy was developed and refined with an information scientist (I.K.). Keywords were generated across five strands detailed in Table 1, with strands combined with the Boolean operator 'AND'. The search was conducted across five databases: Medline, CINAHL, Embase, PsycINFO and AMED (supplemental file).

For peer review only - http://bmjopen.bmj.com/site/about/guidelines.xhtml

Table 1. Search Terms

Strand 1 – TYAC
TYA cancer or TYA oncology or teenage and young adult adj5 cancer or teenage and young adult adj5 oncology or teenage* adj5 cancer or teenage* adj5 oncology or adolescen* adj 5 cancer or adolescen* adj 5 oncology or young people adj 5 cancer or young people adj 5 oncology
Strand 2 – communication
Cancer OR oncology OR malignancy OR leukaemia OR lymphoma
Strand 3 – supporters
Communication skills OR communicat* OR discuss* OR disclos* OR inform* OR interact OR relationship building OR decision making OR communication tools OR communication aids OR psychosocial assessment
Strand 4 - impact
affect OR effect OR influence OR result OR resultant OR impact
Strand 5 - experience
encounter OR involvement OR occurrence OR feel OR "go through" OR experience*

TYAC: teenage and young adult with cancer

Database searches were compiled and de-duplicated in Mendeley, abstracts were screened in Rayyan by two researchers (D.J.C and L.A.M.S.), and 155 full articles were read by three researchers (L.A.M.S., D.J.C., and R.M.T) for eligibility of inclusion in the final analysis, with disagreements resolved by discussion. Papers were included if: they presented empirical research published after 2005; participants had malignant disease, diagnosed aged 13-24 years (for over 50% of participants); the research addressed any area of clinical communication; and the research included supporters (parents, partners, carers, friends etc). Papers were excluded if they were: conference abstracts, unpublished articles, systematic reviews, single case studies, validation research methodology, studies using retrospective documentation in clinical notes, articles focusing on information needs rather than communication skills, or were not in English.

A review-specific data extraction form was used to record participant characteristics and methods from each included paper and results relevant to the three review questions. The final number of included articles totalled 33, the remaining 122 were excluded based on the participants' ages, focus on HCPs or information giving. In tandem to the data extraction process, two members of the review team (E.C. and D.J.C.) independently assessed each paper in terms of its internal validity, appropriateness, and contribution to answering the review questions, using a review-specific version of Gough's Weight of Evidence criteria.(23) Discrepancies in assessment decisions were discussed between reviewers and final scores were agreed through consensus.

Extracted data were entered into Excel to aid the narrative synthesis of the included papers.(21,22) All articles, irrespective of relevance and quality, were included in the review. However, those rated 'medium' and 'high' were given greater weight in the synthesis. An inductive thematic analysis was undertaken to identify the main, recurrent, and important

For peer review only - http://bmjopen.bmj.com/site/about/guidelines.xhtml

data across the studies related to answering each research question. D.J.C. and E.C. explored heterogeneity across the studies. The integration of results from studies utilising different methods and epistemological positions was supported by L.A.M.S. and R.M.T., and consensus in synthesis was reached. The synthesis was further refined through discussion of the review of results and their implications with clinicians, interdisciplinary academic audiences, and all of the co-authors.

Results

A total of 7,727 studies were identified in the search, of which 33 fulfilled the inclusion criteria (Figure 1). The included articles are summarised in Table 2. (table 2 uploaded separately)

All points across the cancer trajectory were represented in the final papers: diagnosis (n=6); (12,24–28) on treatment (n=17); (29–45) end of treatment (completed within one year) (n=2); (46,47) survivorship (more than one-year post-treatment) (n=2); (5,48) and end of life care (n=5). (49–53) One study included patients at more than one point along the cancer care continuum. (54) Most studies (n=18) were conducted in the US (24,27,28,30,34–36,38–45,49,51,53) other countries included the UK, (25,31,32) Australia, (37,47,48) Norway, (12,52) Israel, (46) Iran, (29) Mexico, (50) France, (33) Denmark, (26) and Taiwan, (54) one study recruited from three European countries. (5) Studies used predominantly qualitative methods (n=29) but there were two mixed methods studies and two using quantitative methods. Weight of evidence criteria indicated four were high evidence, (24,30,34,44) twenty-two were medium (5,12,25,27–29,31–33,35,36,38–41,43,45,46,48–50,54) and seven were low evidence. (26,37,42,47,51–53)

The categories used to separate the age groups were lower adolescence (11-14 years), middle adolescence (15-17 years), upper adolescence (18-21 years) and emerging adulthood (22 onwards). Of the papers where the age range at diagnosis could be deduced, the majority of these (19 out of 24) spanned three or more age categories (Table 3). All the papers spanned two or more age categories. In nine of the papers, the age ranges at diagnosis were not available (as age at diagnosis was expressed as a mean or median). Given these factors, it is difficult to ascertain whether any between age group differences exist.

Table 3 – Age range of patients at diagnosis

**Category**	**Number of papers**
Lower and middle adolescence	4
Lower, middle, and upper adolescence	8
Lower, middle, upper adolescence and emerging adulthood	6
Middle and upper adolescence	1
Middle, upper adolescence and emerging adulthood	5
Not specified	9

For peer review only - http://bmjopen.bmj.com/site/about/guidelines.xhtml

Who is present with TYACs in healthcare consultations and communication?

The majority of supporters were mothers (63.5%). When combined, parents represented nearly all the supporters in the included studies (93.5%), see Table 4. Non-parental supporters (2.6%) included partners, sisters, aunts, and grandmothers. The remaining supporters were not categorised due to insufficient information in the article's demographics data (3.9%).(52,53)

Table 4 – Table of supporters

Supporter type	Number of supporters	Percentage quoted to 1 decimal place (%)
"Mother"	342	63.5
"Father"	122	22.6
"Both parents"	20	3.7
"parents" no further specification	20	3.7
"Grandmother"	2	0.4
"Sister"	3	0.6
"Partner"	3	0.6
"Aunt"	3	0.6
"supporters" no further specification	21	3.9
"other"	3	0.6
**Total**	539	100.2

What are TYACs' experiences of communication with the supporter present?

The presence of supporters was concurrently helpful and challenging for TYACs. Supporters undertook several helpful roles and responsibilities: they asked questions on behalf of the TYAC, retained information from HCPs, acted as a conduit of information between the TYAC and HCP, and acted as a ''sounding board'' for the young person.(25,30,44) Some supporters promoted self-advocacy and autonomy for the young person (27,38,40,45). Some reported symptoms on their behalf (44) and proactively negotiated changes to treatment schedules in the interest of the young person.(38)

Findings also suggested that young people could experience limited or ineffective communication in the presence of a supporter. Communication could be directed towards the supporter, not the young person.(27,30,35) Supporters could receive information in the absence of the TYAC and subsequently filter the content before delivering the information to TYACs.(29,32,33,54): *"The parents had hidden a truth that was not theirs to hide"*p533.(33) This reflected the broader predicament that supporters' priorities at times might have competed with those of young people. (25,33,49,50) Supporters could dominate the communication encounter, for instance, parents were seen to interrupt young people, especially when time was limited. (50) Frederick et al found the mean time for adolescent to

For peer review only - http://bmjopen.bmj.com/site/about/guidelines.xhtml

clinician communication was only 5.5% of the total consultation and parent conversation turns directed towards clinicians comprised a mean of 37.5% of all conversation turns. Clinicians directed most communication at the parent rather than the adolescent and none of the clinicians offered patients the opportunity to speak with them alone. (34)

Mutual protectionism appeared to occur, with TYACs and supporters seeking to protect each other from difficult information leading to non-disclosure when both were present. A diagnosis of cancer is devastating for the young person, supporter(s), family, and the wider social network. Repeatedly, there were references to reduced disclosure between the young person and their supporter, in an attempt to shield each other from emotional distress.(12,30,35,37,38,40,44,52) TYACs could experience discomfort and guilt in seeing parents tearful and worried, and felt a burden in response to observing the emotions of supporters.(37,38,51) Some TYACs sought to limit this by withholding concerns to protect their supporters: "*I couldn't talk to mum about my concerns because I didn't want to hurt her*" p 37.(37) In equal measure, supporters were characterised as working hard to stay in control of emotions, be strong and stay in the "now", and they channelled energy into helping.(12,30) Yet this could contribute to an environment of non-disclosure that had the potential to create future communication challenges, such as supporters not knowing the young person's wishes. Examples of this were evident within the end of life care studies.(51,52) Friebert et al found that 86% of young people wanted to receive prognostic information as soon as possible but only 39% of families knew that.(51) Similarly, Jacobs et al found that young people's end of life wishes were not known by their families.(52) In instances where the young person may not be able to communicate, it may help families relieve the impossible burden of making difficult decisions or feelings of regret, if the young person's perspective and wishes are known.(53)

What is the impact of a TYAC's supporter being present in the communication?

Supporters have the potential to facilitate, complicate or obstruct the young person's involvement in decision-making. Involvement had a positive impact on recall,(41) and may improve autonomy, efficacy, adherence, and future self-management.(24) However, the participation of supporters may be experienced as stressful by TYAC as they may become side-lined. (25,39,54) The presence of supporters impacted the young person's level of involvement in decision-making in several ways. In some cases, supporters empowered TYACs to make decisions by withholding their opinion (27) and deferring the final decision to TYACs.(30) However, supporters and TYACs did not perceive decision-making in the same way.(46) Supporters believed that young people oversaw decisions about their care; however, this was not what young people recounted.(24) TYACs reported a lack of communication and limited involvement in decisions (24,29,45) associated later with decisional regret.(24,36)

Deferral of communication and decisions from the young person to supporters was commonplace.(27,30,35) When supporters responded to this pathway of communication, young people then did not see a need to participate in decisions, knowing that their supporter was taking the mantle.(35) In parallel, clinicians were found to direct communication towards supporters and in extreme cases young people were completely excluded from communication and decisions. (29,34,46) An atmosphere characterised by a lack of trust, unanswered questions and uncertainty contributed to the exclusion of young people who

For peer review only - http://bmjopen.bmj.com/site/about/guidelines.xhtml

then sought information from other sources.(29,35,38) Not allowing TYACs to choose their involvement in decision-making violated their autonomy, and increased distrust or resentment of providers and supporters and resulted in lower treatment adherence.(29,35,38)

The decisional involvement preferences of young people were not static: they were context and environment dependent. At diagnosis, heightened emotions and poor health rendered young people unable to engage in communication. (24,25,27,30,36,40) TYACs expressed a desire to be involved in decision making at different levels: some wanted limited involvement from their supporter(s) so they could take the leading role in consultations and their care;(55) several wanted collaboration with supporters and clinicians;(26,27,43) and some completely relied on supporters and HCP's to make decisions on their behalf.(44,45) Davies et al described this as agency, the ability to make free and independent choices. They highlighted the normality of this fluctuation between personal (acting independently), proxy (decisions made on behalf of someone) and collective (decisions are shared) decision making. Whilst this was not always linear, it was part of the cancer trajectory and demonstrated the fluctuating personal agency for TYACs.(31) Some young people reported that supporters and clinicians decided on the their level of involvement in communication and decision-making,(54) and TYACs commented that they did not feel the decision was theirs.(46) Decisional involvement was an interactive, complex, and multifaceted process within the context of the triad, and young people often wanted to be in control of their level of involvement.(28,30) The evidence highlighted that in the presence of a supporter, young people's choice in the their level of involvement in decisions was challenged and not routinely achieved.

Most TYACs felt that it was important for the healthcare team to communicate with them directly and openly.(29,30,32,37,38,48,49) Time alone helped facilitate communication between TYAC and HCP, to ensure that the young person's needs were fully met.(30,35) However, time alone with HCPs was not routinely integrated as a part of consultations with TYACs**.** (34,47) In fact, clinicians were reported as frequently speaking more to parents and TYACs received limited communication from HCPs.(27,30,34,35) In the presence of supporters, as well as withholding concerning information, young people reported feeling discomfort when discussing sensitive topics such as sex or fertility preservation.(27,35)

Young people wanted time alone to communicate with HCPs directly for a variety of reasons. This private line of communication offered a sense of personal agency and allowed them to feel "in the loop" and promoted a sense of autonomy that was threatened by the cancer diagnosis, particularly at the point of diagnosis.(31,49) Young people wanted space to think and privacy during the cancer journey; private lines of communication with HCPs actively promoted this.(30,38,44,45) It also enabled HCPs to get to know the young person and allowed them to ask questions that they may be reluctant to ask in the presence of their supporter, because of embarrassment or emotional shielding.(30) Darabos et al found that 87.5% of oncology providers considered it important to talk to the TYAC without their parents present.(30) Whilst the importance has been highlighted within the data it is also evident that this does not happen as part of routine clinical practice. This could be for several reasons such as not wanting to challenge rules of authority, uncertainty around how best to ask a parent to leave and lack of confidence when communicating with a young person alone.

For peer review only - http://bmjopen.bmj.com/site/about/guidelines.xhtml

Discussion

Principal Findings

Who is present with TYACs in healthcare consultations and communication? For example, who are the supporters?

The included papers in our review demonstrated that most supporters were parents, more commonly mothers. The frequent presence of mothers in consultations is consistent with previous findings. For example, in a UK study in which TYAC nominated a caregiver, 85% were parents, and of those 80% were female.(56) We note that there is a paucity of data for non-parental supporters, and this may represent a reality of clinical practice or a bias towards TYAC-parental dyads over other relational-dyads in this field of research to date.

What are TYACs' experiences of communication with the supporter present?

TYACs experienced supporters facilitating communication by obtaining information, asking questions, advocating, and supporting personal agency of the young person; conversely supporters could hinder communication by gatekeeping information, or dominating communication and thereby rendering young people as bystanders. Young people experienced negative emotions in response to witnessing their supporters in distress.

What is the impact of a TYAC's supporter being present in the communication?

Bidirectional non-disclosure was a coping strategy used by both TYACs and supporters to protect one another from concerns and emotional burden. This limited HCPs ability to effectively assess ideas, concerns, and expectations from both parties when together. In the presence of supporters some young people were less informed, which could impair their ability to engage in decision-making conversations.

Meaning of the study

This is the first review to look specifically at triadic communication in teenagers and young adults with cancer and has demonstrated that there is a paucity of evidence focussed specifically on triadic communication with TYACs. Of the thirty-three studies in the review only one third included all three parties in the triadic communication encounter. However, the review has enabled us to provide answers to the review questions and identify knowledge gaps, including a lack of theory describing triadic communication. Some preliminary theoretical models, such as family involvement in interpersonal healthcare processes,(57) depict the interaction pathways between patients, families and HCP and hypothesise the influence of family on interpersonal processes and outcomes of medical consultations.

The data has clearly identified that parents are the predominating supporter for TYACs, which may be surprising given the inclusion of participants up to the age of 25. Parents can play a significant role when a young person is diagnosed with cancer. Developmentally, a major characteristic that differentiates TYACs from younger children or older adults is the

For peer review only - http://bmjopen.bmj.com/site/about/guidelines.xhtml

progressive increase in their desire and capacity for independence, personal agency, and autonomy. This process is disrupted by a cancer diagnosis: increased parental presence can be perceived as intrusive and reflect reversion to an earlier family dynamic, anchoring adolescents in dependency, restricting self-exploration, and limiting development of a TYACs internal value and belief system.(37,58–60) This has been phrased as “retreating to family” and can impede the maintenance of sustaining a network of peers and cancer negatively impacts peer relationships.(39,61,62) Young people may often be accepting of this, particularly in the early stages of the cancer diagnosis. However, the presence of parents alters the experience and impact of communication with HCPs. It is important to highlight that there is limited literature on TYAC communication encounters with supporters other than parents.(59,63,64) Partners felt relegated to a supporting role by a parent, and mothers struggled to relinquish their existing role as primary supporter.(59,64) It is relevant to note that the participants in these three studies were in their early 20’s.

A key impact of triadic communication is that young people may not be involved in decision making to the level they want. This is consistent with related paediatric oncology literature which consistently reports children’s limited participation in decision-making. (65–67) Clinicians attempted to protect children from ‘too much’ information because of the perception that children are not capable or too vulnerable.(17) The important difference between paediatric and TYAC populations are the legal and ethical obligations towards TYACs who are autonomous, capacitous patients rather than to parents with parental responsibility.

The findings of this review demonstrate the presence of a supporter impacts the involvement of young people in healthcare decisions. Therefore, there are legal and ethical issues, which are critically important, both in research and clinically in TYAC care particularly related to informed consent, capacity, and autonomy. The law relating to children and young people is complex and differs across the UK and internationally. The General Medical Council guidelines in the UK state, “the patient must be the first concern”.(68) HCPs have ethical and legal obligations outlined in UK best practice guidance, statute, and case law.(69) In the UK, parents can legally make decisions for children under 16 years unless the child disagrees and is deemed ‘Gillick Competent’.(70) Moreover, studies have shown children aged 14 and older can approach the level of understanding of adults.(71,72) In contrast, people aged 16 and above are legally able to make decisions for themselves in the UK and are automatically assumed to have capacity (73) and therefore, HCPs must communicate with them in developmentally appropriate ways. Clinicians face a challenge in identifying the best way to communicate with TYACs and their supporter (s). TYACs need parental involvement whilst simultaneously desiring autonomy (35) necessitating careful balancing of the needs of both parties to ensure that the young person is not relegated to a non-participant status.

Strengths and weaknesses

Our review had a number of limitations. We searched systematically and thoroughly for eligible studies, but this is not a well-indexed field of research, and therefore it is possible that some relevant studies were not included in the review. We limited the review to a UK TYAC age range and not the broader age used elsewhere, so the conclusions are applicable to younger adults, up to aged 24 only and not necessarily the age of young adulthood used in

For peer review only - http://bmjopen.bmj.com/site/about/guidelines.xhtml

some countries (between 29 to 39). We also only included papers published in English and therefore papers reflect practices in primarily North America, Australia and Europe, the results may not be applicable to other countries especially where cultural differences affect parental-TYAC or other familial/romantic relational dynamics and where the healthcare culture may be different, e.g., more paternalistic. Despite these limitations, international representation was seen in the eligible studies, TYAC ages were included across the entirety of the specified UK age range and studies represented the journey throughout the cancer experience.

Implications for clinicians and policy makers

Given the degree of challenge and nuance raised, HCPs need training on effective triadic communication. Fourneret concluded that the relationship between TYACs, their parents and HCPs "as being the most difficult one in oncology".(33) Professionals described challenges communicating with both TYACs and parents, especially when loyalties were torn between the two.(5) However, training is currently ad hoc and not interdisciplinary.(74–77) Furthermore, HCPs can find it difficult to apply teaching in this area in clinical practice.(52,78) HCPs need education and training to navigate triadic communication to optimise involvement of the young person whilst attending to a supporter's needs. Experiential learning is the gold standard in teaching methods for clinical communication and is designed to bring about changes in learners' skills. These evidence-based methods are through small group, problem-based simulation in a classroom, with repeated practise and rehearsal of skills under observation with detailed and descriptive feedback. This is arguably warranted here.(79,80)

Triadic communication is a key feature of TYAC care but requires further attention and inclusion in future iterations of key policy documents and guidelines such as the Blueprint of Care (BoC).(81) The BoC is a UK document that helps shape and deliver developmentally appropriate care to TYAC. However, it is recognised that age is poorly correlated with developmental maturity and therefore any communication framework needs to be specific to TYACs, recognising the transitional nature of adolescence meaning a one size fits all approach is likely inadequate.

Unanswered questions and future research

Future research is warranted to triangulate triadic perspectives and understand more about the interactional dynamics of these complex communication encounters. A key research need is investigating how best to support decision-making whilst engaging supporters, understanding their priorities and information needs may conflict.(30,35,36,39) Conflict management must also be understood in the emotional context of young adult oncology. How to effectively educate HCPs to communicate within the triad, to ensure the young person and the supporters needs are met is a priority. This needs to include how best we facilitate time alone between young patients and HCPs. Continued development and utilisation of comprehensive triadic theoretical frameworks may provide guidance and direction for future research, allowing for greater integration and progress with this diverse research area and commonly occurring form of healthcare communication.

For peer review only - http://bmjopen.bmj.com/site/about/guidelines.xhtml

Conclusion

Triadic communication is a pivotal component of communicating with TYACs and the presence of supporters impacts clinical communication both positively and negatively. Young people desire a sense of personal agency, autonomy and control related to information flow and decision making. This includes private lines of communication with HCPs without the presence of supporters. HCPs recognise the importance of time alone with young people; however this does not translate to clinical practice. Therefore, further research on communication dynamics is needed to allow for the development of bespoke, TYAC focussed clinical communication training for HCPs to allow them to effectively facilitate and navigate triadic communication. This then needs to be formally embedded in national guidance and postgraduate training for HCPs working in TYAC care to allow equitable access for TYACs.

For peer review only

Author Contributions:
DC, LS and RT were involved in developing the protocol; DC, IK and LS coordinated the running of the study and were responsible for data acquisition; DC, LS, RT and EC contributed to the analysis; DC drafted the manuscript. All authors have critically reviewed the manuscript for important intellectual content and have read and agreed to the published version of the manuscript. All authors agree to be accountable for all aspects of the work in ensuring that questions related to the accuracy or integrity of any part of the work are appropriately investigated and resolved.

Conflicts of Interest:
The authors declare no conflict of interest.

Ethics Approval:
This was a systematic review and therefore ethics approval was not required.

Funding:
This paper presents work supported by the Wellcome Trust, (grant number G115288) under its Programme PhD for healthcare professionals course awarded to the first author Deborah Critoph, University of Cambridge. The views expressed are those of the author (s) and not necessarily those of the Wellcome Trust.

Data availability statement:
All data relevant to the study are included in the article or uploaded as supplementary information. No previously unpublished primary data are included in the paper. All data relevant to the systematic review are included in the paper or uploaded as supplementary information.

References:

1. Cancer Research UK. Young peoples' cancers incidence statistics [Internet]. 2016 [cited 2019 Jul 10]. Available from: https://www.cancerresearchuk.org/health-professional/cancer-statistics/young-people-cancers/incidence
2. National Institute of Health and Clinical Excellence. Guidance on Cancer Services Improving Outcomes in Children and Young People with Cancer National Institute for Health and Clinical Excellence [Internet]. Nice. 2005. 198 p. Available from: https://www.nice.org.uk/guidance/csg7/resources/improving-outcomes-in-children-and-young-people-with-cancer-update-pdf-773378893
3. Care Quality Commission., NHS Patient Survey Programme. 2018 Children and Young People's patient experience survey. Statistical Release. 2019;(November).
4. White B, Viner RM. Improving communication with adolescents. Arch Dis Child Educ Pract Ed. 2012;97(3):93–7.
5. Essig S, Steiner C, Kuehni CE, Weber H, Kiss A. Improving Communication in Adolescent Cancer Care: A Multiperspective Study. Pediatr Blood Cancer [Internet]. 2016;63(8):1423–30. Available from: http://onlinelibrary.wiley.com/journal/10.1002/(ISSN)1545-5017
6. Lea S, Gibson F, Taylor RM. Holistic Competence": How Is it Developed, Shared, and Shaped by Health Care Professionals Caring for Adolescents and Young Adults with Cancer? J Adolesc Young Adult Oncol. 2021;10(5):503–11.
7. Gibson F, Aldiss S, Fern LA, Phillips B, Gravestock H, Malik S, et al. Reporting the whole story: Analysis of the "out-of-scope" questions from the James Lind Alliance Teenage and Young Adult Cancer Priority Setting Partnership Survey. Health Expect. 2021;
8. Smith LAM, Critoph DJ, Hatcher HM. How Can Health Care Professionals Communicate Effectively with Adolescent and Young Adults Who Have Completed Cancer Treatment? A Systematic Review. J Adolesc Young Adult Oncol [Internet]. 2020 Jan 14; Available from: https://doi.org/10.1089/jayao.2019.0133

For peer review only

9. Reid J, McKeaveney C, Martin P. Communicating with Adolescents and Young Adults about Cancer-Associated Weight Loss. Curr Oncol Rep. 2019;21(2):6.
10. Phillips CR, Haase JE. A Connectedness Primer for Healthcare Providers: Adolescents/Young Adult Cancer Survivors' Perspectives on Behaviors That Foster Connectedness during Cancer Treatment and the Resulting Positive Outcomes. J Adolesc Young Adult Oncol [Internet]. 2018;7(2):174–80. Available from: http://www.liebertpub.com/products/product.aspx?pid=387
11. Mellblom A V, Finset A, Korsvold L, Loge JH, Ruud E, Lie HC. Emotional concerns in follow-up consultations between paediatric oncologists and adolescent survivors: a video-based observational study. Psychooncology [Internet]. 2014 Dec;23(12):1365–72. Available from: https://search.ebscohost.com/login.aspx?direct=true&AuthType=athens&db=cin20&AN=109768272&site=ehost-live
12. Korsvold L, Mellblom AV, Finset A, Ruud E, Lie HC. A content analysis of emotional concerns expressed at the time of receiving a cancer diagnosis: An observational study of consultations with adolescent and young adult patients and their family members. European Journal of Oncology Nursing [Internet]. 2017;26:1–8. Available from: https://search.ebscohost.com/login.aspx?direct=true&AuthType=sso&db=cin20&AN=120589318&site=ehost-live&custid=ns123475
13. Ward A, Critoph D, Westacott R, Williams R, Dogra N. A collaboration on teaching and assessing triadic consultation skills. PEC Innovation [Internet]. 2022;1(October):100091. Available from: https://doi.org/10.1016/j.pecinn.2022.100091
14. van Staa AL. Unraveling triadic communication in hospital consultations with adolescents with chronic conditions: The added value of mixed methods research. Patient Educ Couns. 2011;82(3):455–64.
15. Cahill P, Papageorgiou A. Triadic communication in the primary care paediatric consultation: A review of the literature. British Journal of General Practice. 2007;57(544):904–11.
16. Maras M. Hidden in plain sight. Int J Psychiatry Med. 2022;57(6):481–5.
17. Young B, Dixon-woods M, Windridge KC, Heney D. Study of Patients and Parents. Br Med J. 2003;326(7384):305.
18. Wolff JL, Roter DL. Family presence in routine medical visits: A meta-analytical review. Soc Sci Med [Internet]. 2011;72(6):823–31. Available from: http://dx.doi.org/10.1016/j.socscimed.2011.01.015
19. Laidsaar-Powell RC, Butow PN, Bu S, Charles C, Gafni A, Lam WWT, et al. Physician-patient-companion communication and decision-making: A systematic review of triadic medical consultations. Patient Educ Couns. 2013;91(1):3–13.
20. Tates K, Meeuwesen L. Doctor-parent-child communication. A (re)view of the literature. Soc Sci Med. 2001;52(6):839–51.
21. Popay J, Roberts, H SA et al. Guidance on the Conduct of Narrative Synthesis in Systematic Reviews.
22. Petticrew M RH. Systematic Reviews in the social sciences. In: Systematic reviews in the social sciences [Internet]. Available from: https://onlinelibrary.wiley.com/doi/book/10.1002/9780470754887

For peer review only

23. Gough D. Weight of Evidence: a framework for the appraisal of the quality and relevance of evidence. 2007 [cited 2023 Jul 2]; Available from: https://www.tandfonline.com/action/journalInformation?journalCode=rred20
24. Barakat LP, Schwartz LA, Reilly A, Deatrick JA, Balis F. A Qualitative Study of Phase III Cancer Clinical Trial Enrollment Decision-Making: Perspectives from Adolescents, Young Adults, Caregivers, and Providers. J Adolesc Young Adult Oncol. 2014;3(1):3–11.
25. Hart RI, Cameron DA, Cowie FJ, Harden J, Heaney NB, Rankin D, et al. The challenges of making informed decisions about treatment and trial participation following a cancer diagnosis: a qualitative study involving adolescents and young adults with cancer and their caregivers. BMC Health Serv Res. 2020;20(1).
26. Ingersgaard MV, Tulstrup M, Larsen HB, Schmiegelow Kjeld AO - Ingersgaard MVO http://orcid. org/0000 0002 7037 2104. A qualitative study of decision-making on Phase III randomized clinical trial participation in paediatric oncology: Adolescents' and parents' perspectives and preferences. J Adv Nurs. 2018;74(1):110–8.
27. Olsavsky AL, Theroux CI, Dattilo TM, Klosky JL, O'Brien SH, Quinn GP, et al. Family communication about fertility preservation in adolescent males newly diagnosed with cancer. Pediatr Blood Cancer. 2021;68(7).
28. Weaver MS, Baker JN, Gibson D V, Gattuso JS, Hinds PS, Gibson D V, et al. "Being a good patient" during times of illness as defined by adolescent patients with cancer. Cancer [Internet]. 2016 Jul 15;122(14):2224–33. Available from: http://onlinelibrary.wiley.com/journal/10.1002/(ISSN)1097-0142
29. Bahrami M, Namnabati M, Mokarian F, Oujian P, Arbon P. Information-sharing challenges between adolescents with cancer, their parents and health care providers: a qualitative study. Supportive Care in Cancer [Internet]. 2017;25(5):1587–96. Available from: https://search.ebscohost.com/login.aspx?direct=true&AuthType=athens&db=cin20&AN=122279626&site=ehost-live
30. Darabos K, Berger AJ, Barakat LP, Schwartz LA. Cancer-Related Decision-Making Among Adolescents, Young Adults, Caregivers, and Oncology Providers. Qual Health Res. 2021 Nov 1;31(13):2355–63.
31. Davies J, Kelly D, Hannigan B. "Life then", "life interrupted", "life reclaimed": The fluctuation of agency in teenagers and young adults with cancer. Eur J Oncol Nurs. 2018;36(100885136):48–55.
32. Fern LA, Taylor RM, Whelan J, Pearce S, Grew T, Brooman K, et al. The Art of Age-Appropriate Care. Cancer Nurs [Internet]. 2013;36(5):E27–38. Available from: https://search.ebscohost.com/login.aspx?direct=true&AuthType=athens&db=cin20&AN=110243702&site=ehost-live
33. Fourneret E. Breaking Bad News about Cancer to Adolescents and Young Adults: The French Experience. J Law Med [Internet]. 2018 [cited 2021 Nov 22];25(2):530–7. Available from: https://pubmed.ncbi.nlm.nih.gov/29978652/
34. Frederick NN, Mack JW. Adolescent patient involvement in discussions about relapsed or refractory cancer with oncology clinicians. Pediatr Blood Cancer [Internet]. 2018;65(4):1. Available from: https://search.ebscohost.com/login.aspx?direct=true&AuthType=athens&db=cin20&AN=128132562&site=ehost-live

For peer review only - http://bmjopen.bmj.com/site/about/guidelines.xhtml

35. Hong MK, Wilcox L, Machado D, Olson TA, Simoneaux SF. Care Partnerships: Toward Technology to Support Teens' Participation in Their Health Care. Proc SIGCHI Conf Hum Factor Comput Syst [Internet]. 2016 May 7 [cited 2018 Jun 2];2016:5337–49. Available from: http://www.ncbi.nlm.nih.gov/pubmed/28164178

36. Mack JW, Fasciano KM, Block SD. Adolescent and Young Adult Cancer Patients' Experiences With Treatment Decision-making. Pediatrics. 2019;143(5).

37. Patterson P, Millar B, Desille N, McDonald F. The Unmet Needs of Emerging Adults With a Cancer Diagnosis A Qualitative Study. Cancer Nurs. 2012;35(3):E32–40.

38. Pennant S, Lee SC, Holm S, Triplett KN, Howe-Martin L, Campbell R, et al. The Role of Social Support in Adolescent/Young Adults Coping with Cancer Treatment. Children [Internet]. 2020;7(1):1–25. Available from: https://search.ebscohost.com/login.aspx?direct=true&AuthType=athens&db=cin20&AN=142468088&site=ehost-live

39. Pyke-Grimm KA, Franck LS, Halpern-Felsher B, Goldsby RE, Rehm RS. 3 Dimensions of Treatment Decision Making in Adolescents and Young Adults With Cancer. Cancer Nurs [Internet]. 2020 Nov;43(6):436–45. Available from: https://search.ebscohost.com/login.aspx?direct=true&AuthType=athens&db=cin20&AN=146822019&site=ehost-live

40. Pyke-Grimm KA, Franck LS, Halpern-Felsher B, Goldsby RE, Rehm RS. Day-to-Day Decision Making by Adolescents and Young Adults with Cancer. Journal of Pediatric Hematology/Oncology Nursing [Internet]. 2022;39(5):290–303. Available from: https://ovidsp.ovid.com/athens/ovidweb.cgi?T=JS&CSC=Y&NEWS=N&PAGE=fulltext&D=medl&AN=35538622 https://cambridge-primo.hosted.exlibrisgroup.com/openurl/44CAM/44CAM_services_page?sid=OVID:medline&id=pmid:35538622&id=doi:10.1177%2F27527530211068718&issn=

41. Zarnegar S, Gosiengfiao Y, Rademaker A, Casey R, Albritton KH. Recall of Fertility Discussion by Adolescent Female Cancer Patients: A Survey-Based Pilot Study. J Adolesc Young Adult Oncol [Internet]. 2018 Apr;7(2):249–53. Available from: http://www.liebertpub.com/products/product.aspx?pid=387

42. Viola A, Taggi-Pinto A, Sahler OJZ, Alderfer MA, Devine KA, Ed M, et al. Problem-solving skills, parent-adolescent communication, dyadic functioning, and distress among adolescents with cancer. Pediatr Blood Cancer. 2018;65(5).

43. Weaver MS, Baker JN, Gibson D V, Gattuso JS, Sykes AD, Hinds PS. Adolescents' preferences for treatment decisional involvement during their cancer. Cancer [Internet]. 2015;121(24):4416–24. Available from: http://onlinelibrary.wiley.com/journal/10.1002/(ISSN)1097-0142

44. Sisk BA, Keenan M, Kaye EC, Baker JN, Mack JW, DuBois JM. Co-management of communication and care in adolescent and young adult oncology. Pediatr Blood Cancer [Internet]. 2022;69(10):e29813. Available from: https://ovidsp.ovid.com/athens/ovidweb.cgi?T=JS&CSC=Y&NEWS=N&PAGE=fulltext&D=mesx&AN=35719025 https://cambridge-primo.hosted.exlibrisgroup.com/openurl/44CAM/44CAM_services_page?sid=OVID:medline&id=pmid:35719025&id=doi:10.1002%2Fpbc.29813&issn=1545-500

45. Sisk BA, Keenan M, Schulz GL, Kaye E, Baker JN, Mack JW, et al. Interdependent functions of communication with adolescents and young adults in oncology. Pediatr Blood Cancer [Internet]. 2022; Available from: http://onlinelibrary.wiley.com/journal/10.1002/(ISSN)1545-5017

For peer review only - http://bmjopen.bmj.com/site/about/guidelines.xhtml

46. Barlevy D, Wangmo T, Ash S, Elger BS, Ravitsky V. Oncofertility decision making: Findings from Israeli adolescents and parents. J Adolesc Young Adult Oncol [Internet]. 2019;8(1):74–83. Available from: https://www.proquest.com/scholarly-journals/oncofertility-decision-making-findings-israeli/docview/2426222863/se-2?accountid=47868
47. Sawyer SM, McNeil R, Thompson K, Orme LM, McCarthy MAOSSM; O http://orcid. org/0000 0002 9095 358X, Sawyer SM, et al. Developmentally appropriate care for adolescents and young adults with cancer: how well is Australia doing? Supportive Care in Cancer [Internet]. 2019;27(5):1783–92. Available from: http://link.springer.de/link/service/journals/00520/index.htm
48. Ellis SJ, Wakefield CE, McLoone JK, Robertson EG, Cohn RJ. Fertility concerns among child and adolescent cancer survivors and their parents: A qualitative analysis. J Psychosoc Oncol. 2016;34(5):347–62.
49. Ananth P, Mun S, Reffat N, Li R, Sedghi T, Avery M, et al. A Stakeholder-Driven Qualitative Study to Define High Quality End-of-Life Care for Children With Cancer. J Pain Symptom Manage [Internet]. 2021;62(3):492–502. Available from: https://ovidsp.ovid.com/athens/ovidweb.cgi?T=JS&CSC=Y&NEWS=N&PAGE=fulltext&D=med19&AN=33556497 https://cambridge-primo.hosted.exlibrisgroup.com/openurl/44CAM/44CAM_services_page?sid=OVID:medline&id=pmid:33556497&id=doi:10.1016%2Fj.jpainsymman.2021.01.
50. Cicero-Oneto CE, Valdez-Martinez E, Bedolla M. Decision-making on therapeutic futility in Mexican adolescents with cancer: a qualitative study. BMC Med Ethics [Internet]. 2017;18:74. Available from: https://search.ebscohost.com/login.aspx?direct=true&AuthType=athens&db=cin20&AN=127010047&site=ehost-live
51. Friebert S, Grossoehme DH, Baker JN, Needle J, Thompkins JD, Cheng YI, et al. Congruence Gaps Between Adolescents With Cancer and Their Families Regarding Values, Goals, and Beliefs About End-of-Life Care. JAMA Netw Open [Internet]. 2020 May 19;e205424–e205424. Available from: https://search.ebscohost.com/login.aspx?direct=true&AuthType=athens&db=cin20&AN=143389091&site=ehost-live
52. Jacobs S, Perez J, Cheng YI, Sill A, Wang J, Lyon ME. Adolescent end of life preferences and congruence with their parents' preferences: Results of a survey of adolescents with cancer. Pediatr Blood Cancer [Internet]. 2015;62(4):710–4. Available from: https://search.ebscohost.com/login.aspx?direct=true&AuthType=athens&db=cin20&AN=109777063&site=ehost-live
53. Lyon ME, Jacobs S, Briggs L, Cheng YI, Wang J. Family-centered advance care planning for teens with cancer. JAMA Pediatr. 2013;167(5):460–7.
54. Wu LM, Chiou SS, Lin PC, Liao YM, Su HL. Decisional conflicts, anxiety, and perceptions of shared decision-making in cancer treatment trajectory among adolescents with cancer: A longitudinal study. Journal of Nursing Scholarship. 2022;54(5):589–97.
55. Mack JW, Fasciano KM, Block SD. Adolescent and Young Adult Cancer Patients' Experiences With Treatment Decision-making. Pediatrics. 2019;143(5).
56. Martins A, Alvarez-Galvez J, Fern LA, Vindrola-Padros C, Barber JA, Gibson F, et al. The BRIGHTLIGHT National Survey of the Impact of Specialist Teenage and Young Adult

For peer review only - http://bmjopen.bmj.com/site/about/guidelines.xhtml

Cancer Care on Caregivers' Information and Support Needs. Cancer Nurs. 2021;44(3):235–43.
57. Dalton JM. Development and testing of the theory of collaborative decision-making in nursing practice for triads. J Adv Nurs. 2003;41(1):22–33.
58. Grinyer A. Young people living with cancer:implications for policy and practice. Open University Press; 2007. 182 p.
59. Moules NJ, Laing CM, Estefan A, Schulte F, Guilcher GMT. "Family Is Who They Say They Are"(a): Examining the Effects of Cancer on the Romantic Partners of Adolescents and Young Adults. J Fam Nurs. 2018;24(3):374–404.
60. Silva M, Barretta F, Luksch R, Terenziani M, Casanova M, Spreafico F, et al. Adolescents with cancer on privacy: Fact-finding survey on the need for confidentiality and space. Tumori. 2021 Oct 1;107(5):452–7.
61. KM B, Smith A, Schmidt S, TH K, Zebrack B, CF L, et al. Positive and negative psychosocial impact of being diagnosed with cancer as an adolescent or young adult. Cancer (0008543X) [Internet]. 2012 Oct 15;118(20):5155–62. Available from: https://search.ebscohost.com/login.aspx?direct=true&AuthType=athens&db=cin20&AN=104372613&site=ehost-live
62. Pyke-Grimm KA, Franck LS, Patterson Kelly K, Halpern-Felsher B, Goldsby RE, Kleiman A, et al. Treatment Decision-Making Involvement in Adolescents and Young Adults With Cancer. Oncol Nurs Forum [Internet]. 2019;46(1):E22–37. Available from: https://search.ebscohost.com/login.aspx?direct=true&AuthType=athens&db=cin20&AN=133553348&site=ehost-live
63. Davies J. The experience and role of partners in helping to meet the support needs of adolescents and young adults with cancer. Journal of Advanced Nursing (John Wiley & Sons, Inc) [Internet]. 2019 May;75(5):1119–25. Available from: https://search.ebscohost.com/login.aspx?direct=true&AuthType=athens&db=cin20&AN=135934208&site=ehost-live
64. Iannarino NT. "It's My Job Now, I Guess": Biographical disruption and communication work in supporters of young adult cancer survivors. Commun Monogr [Internet]. 2018;85(4):491–514. Available from: https://doi.org/10.1080/03637751.2018.1468916
65. Coyne I, Amory A, Gibson F, Kiernan G. Information-sharing between healthcare professionals, parents and children with cancer: More than a matter of information exchange. Eur J Cancer Care (Engl). 2016;25(1):141–56.
66. de Vries MC, Wit JM, Engberts DP, Kaspers GJL, van Leeuwen E. Pediatric Oncologists' Attitudes Towards Involving Adolescents in Decision-Making Concerning Research Participation. Pediatr Blood Cancer. 2010;55(1):123–8.
67. Frederick NN, Bingen K, Bober SL, Cherven B, Xu X, Quinn GP, et al. Pediatric oncology clinician communication about sexual health with adolescents and young adults: A report from the children's oncology group. Cancer Med [Internet]. 2021;10(15):5110–9. Available from: https://ovidsp.ovid.com/athens/ovidweb.cgi?T=JS&CSC=Y&NEWS=N&PAGE=fulltext&D=med20&AN=34128352 https://cambridge-primo.hosted.exlibrisgroup.com/openurl/44CAM/44CAM_services_page?sid=OVID:medline&id=pmid:34128352&id=doi:10.1002%2Fcam4.4077&issn=2045-76

For peer review only - http://bmjopen.bmj.com/site/about/guidelines.xhtml

68. General Medical Council. Guidance for All Doctors. 0-18 Years: Guidance for All Doctors [Internet]. 2018;11. Available from: https://www.gmc-uk.org/ethical-guidance/ethical-guidance-for-doctors/0-18-years/making-decisions#paragraph-29
69. British Medical Association. Children and young people toolkit A toolkit for doctors. BMA.org.uk [Internet]. 2021; Available from: https://www.bma.org.uk
70. Gillick T. Gillick competence What is Gillick Here ' s what you need to know about capacity and The Gillick case. 2022;
71. Joffe S, Fernandez C V., Pentz RD, Ungar DR, Mathew NA, Turner CW, et al. Involving children with cancer in decision-making about research participation. Journal of Pediatrics. 2006;149(6).
72. Mårtenson EK, Fägerskiöld AM. A review of children's decision-making competence in health care. J Clin Nurs. 2008;17(23):3131–41.
73. Department for Constitutional Affairs. Mental Capactity Act 2005: Code of Practice. Mental Capactity Act 2005: Code of Practice. 2007;1–301.
74. Lea S, Gibson F, Taylor RM. Holistic Competence": How Is it Developed, Shared, and Shaped by Health Care Professionals Caring for Adolescents and Young Adults with Cancer? J Adolesc Young Adult Oncol. 2021;10(5):503–11.
75. Essig S, Steiner C, Kuehni CE, Weber H, Kiss A. Improving Communication in Adolescent Cancer Care: A Multiperspective Study. Pediatr Blood Cancer. 2016;63(8):1423–30.
76. Essig S, Steiner C, Kühne T, Kremens B, Langewitz W, Kiss A. Communication Skills Training for Professionals Working with Adolescent Patients with Cancer Based on Participants' Needs: A Pilot. https://home.liebertpub.com/jayao. 2019 Jun 4;8(3):354–62.
77. Coad J, Smith J, Pontin D, Gibson F. Consult, Negotiate, and Involve: Evaluation of an Advanced Communication Skills Program for Health Care Professionals. Journal of Pediatric Oncology Nursing. 2018 Jul 1;35(4):296–307.
78. Ruhe KM, Badarau DO, Brazzola P, Hengartner H, Elger BS, Wangmo T, et al. Participation in pediatric oncology: views of child and adolescent patients. Psychooncology. 2016;25(9):1036–42.
79. Blackmore A, Kasfiki EV, Purva M. Simulation-based education to improve communication skills: A systematic review and identification of current best practice. BMJ Simul Technol Enhanc Learn. 2018;4(4):159–64.
80. Mahoney P, Macfarlane S, Ajjawi R. A qualitative synthesis of video feedback in higher education. Teaching in Higher Education [Internet]. 2019;24(2):157–79. Available from: https://doi.org/10.1080/13562517.2018.1471457
81. Smith S, Mooney S, Cable M, Taylor RM. THE BLUEPRINT OF CARE for teenagers and young adults with cancer Second edition. 2016; Available from: https://www.teenagecancertrust.org/sites/default/files/BlueprintOfCare_2ndEdition.pdf

For peer review only

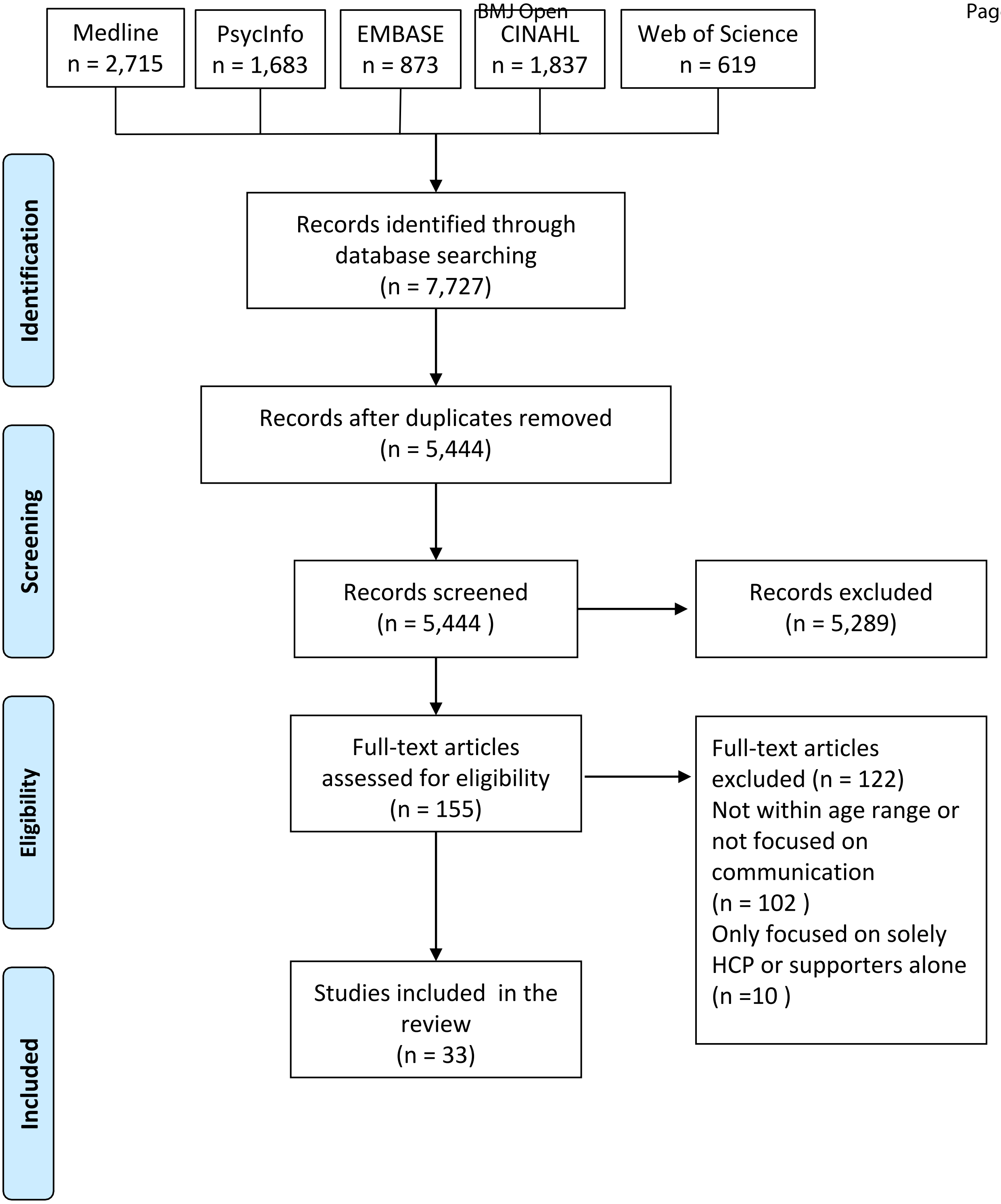
Medline
n = 2,715
PsycInfo
n = 1,683
EMBASE
n = 873
CINAHL
n = 1,837
Web of Science
n = 619
Identification
Records identified through database searching
(n = 7,727)
Records after duplicates removed
(n = 5,444)
Screening
Records screened
(n = 5,444)
Records excluded
(n = 5,289)
Eligibility
Full-text articles assessed for eligibility
(n = 155)
Full-text articles excluded (n = 122)
Not within age range or not focused on communication
(n = 102)
Only focused on solely HCP or supporters alone
(n =10)
Included
Studies included in the review
(n = 33)

Table 2. Summary of Articles

First author (year)	Title	Setting	Study Type - analysis method *Data collection*	Focus	Participant Characteristics¥	Key findings
Ananth (2021)	A Stakeholder-Driven Qualitative Study to Define High Quality End-Of-Life Care for Children with Cancer	USA Multicentre	Qualitative - thematic analysis<br>*Semi-structured interviews and focus groups.*	To explore end of life care (EOLC) priorities for children with cancer and their families.	54 participants:<br>**10 AYACs (age range: 17-23 years)**<br>25 parents (including 12 bereaved parents)<br>19 healthcare professionals	Important to have direct communication with the child or young person regarding decision-making.<br>Interdisciplinary care with integrated teams is vital for high quality end of life care. Continuity of healthcare professionals was positive.<br>AYACs would prefer to die at home but family and healthcare professionals may be hesitant.
Bahrami (2017)	Information Sharing Challenges Between Adolescents with Cancer, their Parents and Health Care Providers: A Qualitative Study	Iran Single centre	Qualitative descriptive-exploratory study - grounded theory analysis<br>*Semi-structured interviews.*	Information sharing between AYACs, parents and health professionals.	33 participants:<br>**12 AYACs (age range at interview: 15-20 years, within 1 year of diagnosis)**<br>6 supporters<br>6 healthcare professionals	AYACs feel they are excluded from information-sharing sessions between parents and healthcare professionals. This leads to disaffiliation, confusion and AYACs seek information from 'inferior' sources.<br>Parents were often the first receivers of information allowing them to act as gatekeepers controlling information to flow to AYACs. Parents may want to shield AYACs from bad news.<br>Trust and honesty are the foundations of effective communication between AYACs and healthcare professionals. AYACs reacted negatively towards dishonesty.
Barakat (2014)	A Qualitative Study of Phase III Cancer Clinical Trial Enrollment Decision Making Perspectives from AYAC, Caregivers and Providers	USA Single centre	Qualitative - thematic analysis<br>*Semi-structured interviews.*	Clinical trial enrollment.	40 participants:<br>**13 AYACs (age range: 15-21 years)**<br>16 supporters<br>11 healthcare professionals	Four patterns of decision-making patterns identified:<br>1. AYAC abdicates to caregiver,<br>2. Caregiver based and AYAC approved,<br>3. Collaborative,<br>4. AYAC in charge of decision-making.<br>Caregivers perceived AYAC to be in charge of decision making most of the time whereas the AYACs felt that "AYAC abdicates to carer" was the most common form of decision making.<br>Distress and poor health limited AYAC involvement in the decision.<br>Developmental and emotional maturity facilitated involvement.

For peer review only - http://bmjopen.bmj.com/site/about/guidelines.xhtml

Barlevy (2019)	Oncofertility decision making: findings from Israeli adolescents and parents	Israel<br>Single centre	Qualitative- thematic analysis<br><br>*semi-structured interviews*	To understand adolescent oncofertilty decision making in Israel, from perspectives of parents and adolescents	35 participants<br>**16 AYACs (age range 12-16 years)**<br>19 parents	As in other cultural contexts, Israeli adolescents and parents demonstrate multifaceted decision making with respect to oncofertility. A significant finding from this study suggests that health professional shy from discussing posthumous planning of cryopreserved materials with adolescent cancer patients and their parents. 5 out of 16 AYAs felt that the decision was not theirs and that it was instead the parents' or the pysicians' to make. Some parents felt that the decision was made by the clinician - explicit or implicit recommendations from the clinician strongly influence decision making. No decisional regret expressed by any members of the dyad.
Cicero-Oneto (2017)	Decision-making on therapeutic futility in Mexican adolescents with cancer: a qualitative study	Mexico<br>Multicentre	Qualitative - thematic analysis<br><br>*Semi-structured interviews*	Decision making on therapeutic futility	32 Participants<br>13 paediatric oncologists<br>13 parents or primary carers<br>**6 AYACs (age range 13-18 years)**	Four themes were identified<br>1. flow of information to inform decision making<br>2. disclosure of prognosis<br>3. decision maker and stakeholder involved in decision making<br>4. barriers and facilitators to decision making<br>Differing values and agendas. The parents valued messages to “life the spirits” whereas the AYACs values honesty from the healthcare professionals<br>Gatekeeping of information.<br>Theme of “deference to authority”

For peer review only

Darabos (2021)	Cancer Related Decision Making Among AYAC, Care Givers and Oncology Providers	USA Single centre	Qualitative – content analysis with hypothesis coding<br><br>*Semi-structured interviews.*	Decision-making and triadic communication	30 Participants:<br>**11 AYACs (age range: 15-24 years)**<br>11 supporters<br>8 healthcare professionals	Four decision-making patterns were identified:<br>1. AYAC driven,<br>2. Collaborative,<br>3. Deferral to parents,<br>4. Provider-based, AYA/Caregiver-approved.<br>Collaborative decision-making and AYAC-driven decisions were most commonly described. There was recognition that some decision-making was day/context dependent.<br>AYACs were more likely to drive decisions regarding supportive care than treatment related decisions.<br>AYACs and caregivers explained how cognitive and emotional processes influenced cancer related decisions.<br>Emotional coping was more common than problem-based coping<br>Direct and honest communication contributes to a stronger relationship.<br>Individuality is key along with flexibility.<br>Time alone is important.
Davies (2019)	'Life then', 'life interrupted', 'life reclaimed': the fluctuation of agency in teenagers and young adults with cancer	UK single centre	qualitative - thematic analysis<br><br>*case studies - multiple interviews*	Fluctuation of agency across time and between cases	22 participants<br>**5 AYACs (16-24 years)**<br>**5 parents carers** (2 fathers, 3 mothers, 1 couple),<br>5 healthcare professionals (4 nurses and 1 oncology consultant)<br>5 other supporters (1 boyfriend, 1 girlfriend, 1 aunt, 2 friends)	Agency fluctuates over time within cases and between cases. Agency can fluctuate between personal, proxy and collective perspectives. Personal agency is high prior to diagnosis, decreases after diagnosis and is reclaimed after treatment.
Ellis (2016)	Fertility concerns among child and adolescent survivors and parents: a qualitative study	Australia single centre	Qualitative semi-structured telephone interviews	Fertility related themes with AYACs who are recently off treatment and with their parents	97 participants from 45 families<br>**19 AYACs (age range 7-17 at diagnosis, mean age 13.3)**<br>44 mothers and 34 fathers	Both parents and AYACs are concerned about the potential impacts on fertility of treatment. Poor doctor-patient communication was reported and conversations about fertility were frequently interrupted to discuss illness and treatment. These fertility discussions were not then continues once the AYAC was off treatment

For peer review only - http://bmjopen.bmj.com/site/about/guidelines.xhtml

Essig (2016)	Improving Communication in Adolescent Cancer Care: A Multiperspective Study	Germany, Austria, Switzerland<br><br>Number of centres not stated	Qualitative – inductive thematic analysis<br><br>*Focus groups.*	Explore effective communication with AYACs for communication skills training.	54 participants:<br>**16 AYACs (age range: 13-19 years)**<br>8 parents<br>30 healthcare professionals	Decision-making can cause conflict when adolescents are cognitively mature but legally lack the ability to make decisions.<br>AYACs feel a loss of autonomy.<br>Age-appropriate environments are important.<br>Effective communication differs depending on the type of professional (i.e., doctor vs nurse)<br>Adolescents negatively affect communication when:<br>1. They are indifferent.<br>2. There priorities conflict with treatment<br>3. They conflict with parents.<br>Healthcare professionals negatively affect communication when:<br>1. They don't treat the adolescent in an age-appropriate way.<br>2. They don't take the adolescent seriously.<br>3. They give too much information or withhold important information
Fern (2013)	The Art of Age-Appropriate Care	UK<br>Number of centres not stated	Qualitative – thematic analysis<br><br>*Peer-to-peer interviews, field notes and spider diagrams from focus groups.*	Review a conceptual model of AYACs' cancer care experiences.	11 participants:<br>**11 AYACs (age range: 13-25 years)**	Young people must be kept at the centre of interactions in recognition of their stated needs:<br>1. Engagement.<br>2. Individually tailored information.<br>3. Support unproxied by parents/family.<br>AYACs did not want information to be directed at parents but at them.<br>AYACs found it embarrassing when sensitive information was revealed in the presence of their parents.<br>Lack of continuity of healthcare professionals leads to AYACs dissatisfaction and irritation having to repeat their cancer story.

For peer review only - http://bmjopen.bmj.com/site/about/guidelines.xhtml

Fourneret (2018)	Breaking bad news about cancer to adolescents and young adults: the french experience	France Multicentre	qualitative semi-structured interviews	Explore the effectiveness and implementation of the French announcement protocol in 7 french paediatric oncology centres	90 participants<br>**27 AYACs (21 were 14-17 and 6 were 18-22)**<br>30 parents (16 mothers, 5 fathers, 9 parents together at the appointment)<br>33 healthcare professionals	Parents and AYACs have different needs - both of which need to be accounted for when breaking bad news. Awkward and premature announcements were noted<br>The announcement consultation – young patients were never alone when informed of their disease; either with parents (n=31) (parents were informed before their child 10 out of 31 times - this was motivated by a compassionate goal of preparing the parents so they can better support the child when the bad news is broken) or close family member/sibling or boyfriend or girlfriend (n=2).<br>Some parents withheld info and some AYACs preferred parents not to know their diagnosis<br>Asymmetry in the triad discussed - but the key quality needed in the triad is mutual trust<br>HCP found parental presence helpful in the study. HCPs should show empathy (no neutrality) and attention to detail
Frederick (2018)	Adolescent Patient Involvement in Discussions About Relapsed or Refractory Cancer with Oncology Clinicians.	USA Single centre	Qualitative – content analysis<br><br>*Audiotaped conversations.*	Breaking bad news of relapsed or refractory cancer.	75 participants:<br>**11 AYACs (age range: 12.6-17.5 years)**<br>44 supporters<br>20 healthcare professionals	Adolescent patients' involvement in conversations about relapsed or refractory cancer is limited.<br>Adolescents were accompanied by one (27%) two (64%) or more than two (18%) family members in the discussion.<br>Adolescents spoke 3.5% of words compared to 66.9% clinicians and 30% parents.<br>No conversations included instances in which the clinicians' asked adolescents for their communication preferences or desired role in decision-making.
Friebert (2020)	Congruence gaps between adolescents with cancer and their families regarding values, goals and beliefs about end-of-life care	USA multicentre	*Qualitative cross-sectional study*	End of life care	126 parent-AYAC dyads AYACs (14-20 years, mean age 16.9)	Young people wanted early information (86%) but only 39% families knew this. Families understanding of what was important to their adolescents when dealing with their own dying was excellent for wanting honest answers from their physician and understanding treatment choices but poor for dying a natural death and being off machines that extend life, if dying.<br>Parents do not know what AYACs want at the end of life

For peer review only - http://bmjopen.bmj.com/site/about/guidelines.xhtml

Hart (2020)	The Challenges of Making Informed Decisions About Treatment and Trial Participation Following Cancer: A Qualitative Study with Adolescent and Young Adults with Cancer and Care Givers	UK Multicentre	Qualitative – thematic analysis<br><br>*Semi-structured interviews.*	Shared decision-making – primary treatment and trial participation – at diagnosis.	33 participants:<br>**18 AYACs (age range: 16-24 years)**<br>15 supporters	AYACs struggled to process information around diagnosis, exacerbated by symptom burden, emotions, and the fast pace of clinical activity.<br>Some AYACs disengaged from conversation topics which were distressing.<br>There are limited options for 'real' decision-making at diagnosis. However, many preferred this when they were already overwhelmed by emotions/symptoms.<br>For trial enrollment, many AYACs allowed themselves to be steered by the recommendation of the healthcare professional who recruited them, thinking they were acting in their best interests.
Hong (2016)	Care Partnerships: toward technology to support teen's participation in their health care	US multicentre	Qualitative semi-structured interviews and observations	To investigate how technology can support the partnerships between AYACs, parents and clinicians when the AYAC is experiencing complex chronic illness	33 interviews.<br>**15 with AYACs (13 of whom had cancer. age range 13-17)**<br>15 parents (10 mothers, 1 fathers, 1 aunt and 2 fathers and mothers together)<br>8 clinician caregivers	Participants faced challenges concerning:<br>1) Teens' limited participation in their care<br>2) communicating emotionally sensitive information<br>3) managing physical and emotional responses<br>Time alone with clinicians was important. Mutual protectionism or the need to "emotionally protect eachother" was prevalent.
Ingersgaard (2018)	A qualitative study on decision-making on Phase III randomized clinical trial participation in paediatric oncology: adolescents' and parents' perspectives and preferences	Denmark	qualitative exploratory study - in-depth semi-structured interviews with thematic analysis	To explore patients' and AYACs' motivs for accepting/ declining participation in the AL2008 trial and adolescents' involvement in decision making	16 participants<br>**5 AYACs (age range 12-16)**<br>6 parents of AYACs<br>5 parents of children aged 3-10 years with cancer	Key themes<br>1) altruism - wanting to help future AYACs<br>2) trust in the clinicians<br>3) individuals perceptions of cure contra toxicity<br>4) adolescents as active participants in the decision making process<br>5) parental responsibility and authority<br>6) the difficulty of uncertainty

For peer review only - http://bmjopen.bmj.com/site/about/guidelines.xhtml

Jacobs	Adolescent end of life preferences and congruence with their parents' preferences: results of a survey of adolescents with cancer	Norway	Qualitative<br><br>three sessions of dyadic interviews	To explore AYACs' end of life preferences and to assess the congruence of these preferences with the parents' beliefs	<u>17 adolescent/ family dyads</u><br>**17 AYACs (age range 14-21, 71% under 18)**	Adolescents with cancer were comfortable discussing EOL, and the majority preferred to talk about EOL issues before they are facing EOL. There were substantive areas of agreement between adolescents and their surrogates, but important facets of adolescents' EOL wishes were not known by their families, reinforcing the importance of eliciting individual preferences and engaging dyads so parents can understand their children's wishes. 53% of AYACs had never spoken about their end of life preferences but 82% considered it important to let their loved ones know their wishes.
Korsvold (2017)	A content analysis of emotional concerns expressed at the time of receiving a cancer diagnosis: An observational study of consultations with adolescent and young adult patients and their family members	Norway	exploratory mixed methods study<br><br>audio recorded consultations	To investigate the emotional concerns of AYACs at the time of diagnosis and how to quantify how healthcare professionals respond	<u>18 participants</u><br>**9 AYACs (age range 13-23)**<br>Present with mother (n=9), father (n=1), sister (n=1) or mother and father (n=2)	Four major themes of emotional concerns expressed by AYA patients and their family members during consultations for a cancer diagnosis:<br>1) side effects/late effects or infertility,<br>2) "what happens in the near future/practical aspects",<br>3) fear<br>4) sadness<br>AYA patients and family members expressed emotional concerns. HCPs typically responded by providing information, rather than affective aspects of the concerns In the sadness theme however, an explicit provide space affective response was the most common response (n=8) followed by an explicit provide space content response (n=7)<br>To make patients 'feel known' HCP should pay attention to the affective aspect of the expressed concern.
Lyon (2013)	Family-Centered Advance Care Planning for Teens With Cancer	USA single centre	Qualitative a randomised control pilot study	To examine the efficacy of family-centres ACP	<u>30 dyads</u><br>**mean age of AYACs 16**<br>17 were randomised to intervention and 13 were randomised to control<br>87% of surrogates were biological parents and were female	The model (ACP) increased congruence in the triad compared to the control standard of care group– so it is key. The family centres ACP AYACs reported feeling more informed that the control group

For peer review only - http://bmjopen.bmj.com/site/about/guidelines.xhtml

Mack (2019)	Adolescent and Young Adult Cancer Patients' Experiences with Treatment Decision-Making	USA Single Centre	Quantitative – multivariate analysis, logistic regression<br><br>*Surveys at diagnosis, 4 and 12 months.*	Treatment decision-making	203 participants:<br>**203 AYACs (age range: 15-29 years)**	A majority of AYACs (58%) want to share decision-making with oncologists. The remainder were split between the AYAC wanting primary responsibility in decision-making (20%) or wanting their oncologist to have primary responsibility (22%).<br>A lower proportion of younger AYACs wanted sole responsibility but this did not achieve statistical significance (P = 0.07).<br>The majority (90%) of AYACs who lived with a parent/guardian wanted some form of input from their parents (either collaborative or considering their opinion).<br>Younger AYACs (15-17 years) were more likely to want greater involvement by their parents but were also more likely to be less involved than they wanted to be relative to their parents.<br>Decisional regret was less likely among AYACs who trusted oncologists completely, and who reported that oncologists understood what was important to them when treatment started.
Olsavsky (2021)	Family communication about fertility preservation in adolescent males newly diagnosed with cancer	USA	Qualitative	To explore fertility preservation communication among mothers, fathers and their male adolescents newly diagnosed with cancer.	87 participants:<br>**33 AYAC aged 12-25**<br>32 mothers<br>22 fathers<br><br>Representing 37 families in total.	Five process themes:<br>(1) Reliance on health care team and social support networks to facilitate FP decisions (noted just by parents),<br>(2) withholding parental opinion and deferring the decision to the adolescent,<br>(3) ease of communication,<br>(4) communication barriers and facilitators,<br>(5) not being present or not remembering details of FP conversations.<br>Four content themes:<br>(1) preference for biological parenthood (or grandparenthood),<br>(2) consideration of future partner of AYAC's desire for biological parenthood,<br>(3) sperm banking whilst it is a viable option,<br>(4) openness to alternative parenthood options

For peer review only - http://bmjopen.bmj.com/site/about/guidelines.xhtml

Patterson (2012)	The Unmet Needs of Emerging Adults With a Cancer Diagnosis	Australia	Qualitative	Aim to contribute to the limited research base and inform our understanding of the needs of emerging adults with a diagnosis of cancer from a developmental perspective that appreciates the key transitional tasks of emerging adulthood identified by Arnett	14 Participants:<br>14 AYAC aged 20-25, average age of 22	A cancer experience poses the potential for significant impact on the four requirements for achievement of adulthood.<br><br>The needs of these emerging adults were grouped into six themes; information, healthcare provision, daily living, interpersonal support, identity renegotiation and emotional distress.<br><br>These themes relate directly to the four requirements of adulthood.<br>1. The task of accepting responsibility for oneself emphasises the importance of empowering AYAC in their communication with HCP.<br>2. The task of deciding on personal beliefs and values highlights the importance of keeping the AYAC informed and encouraging them in decisions giving maximum opportunity to explore beliefs.<br>3. Establishing a relationship with parents as equals highlights the importance of maximising AYAC autonomy in relation to supporters.<br>4. The task of becoming financially independent highlights the importance of minimising disruption to daily life.

For peer review only

Pennant (2020)	The Role of Social Support in Adolescent/Young Adults Coping with Cancer Treatment	USA	Qualitative	To explore specific actions that help AYAC and what behaviours they want from their social supports	20 Participants:<br>10 AYAC ages 15-26, mean age 18.9 years<br>10 parents	Themes of support included; presence, distraction, positive attitude, maintaining AYAC autonomy, communication and advocacy.<br>Mothers were the most noted family support.<br>AYAC patients can differ in their preferences throughout treatment and this can, at times, appear contradictory.<br>AYACs appear to want autonomy and independence, but appreciate help with daily tasks from their parents.<br>They express the desire for privacy, but also value physical presence and communication<br>Parents must oscillate between being involved in and catering to their AYAC child's needs during treatment while allowing space for independence and autonomy.<br>The findings underscore the importance of maintaining open communication with AYAC patients about their preferences and needs throughout the course of treatment and asking them about both individual and social preferences, which may change frequently.
Pyke-Grimm (2020)	3 Dimensions of Treatment Decision Making in Adolescents and Young Adults with Cancer.	USA Multicentre	Qualitative – ethnographic<br><br>*Semi-structured interviews, field notes.*	Explore the preferences of AYACs for involvement in healthcare decisions	16 participants:<br>**16 AYACs (age range: 14.7-20 years)**	Emotions around diagnosis inhibit information receptiveness and ability to engage in treatment decisions (especially important decisions).<br>Initially AYACs struggle with the jargon and plethora of medical terms which are being used. They have limited knowledge which limits their questions, this increases over time.<br>The importance of decisions differs from one AYACs to the next. Also, some decisions are seen as having only one 'real' option.<br>AYACs engaged in minor decisions much earlier in their treatment, and some began engaging in more important decisions later in treatment.<br>AYACs could adopt an active (sole decision maker), collaborative (with healthcare professionals/supporters) or passive (healthcare professionals/supporters as decision makers) role.

For peer review only - http://bmjopen.bmj.com/site/about/guidelines.xhtml

Pyke-Grimm (2022)	Day-to-Day Decision Making by Adolescents and Young Adults with Cancer	USA	Qualitative Interpretive focused ethnography within the socio-logic tradition, informed by symbolic interactionism	To explore involvement of AYAs with cancer in day-to-day decisions affected by their cancer and treatment.	16 Participants:<br>16 AYAC aged 15-20 (at time of interview - with an average of one year from diagnosis)	Factors influence the involvement of AYAC in decision making such as the type of decision, the point in the cancer journey. They want to be involved.<br>Four day to day decision making categories were identified: mental mindset, self care practices, self-advocacy and negotiating relationships.<br>Parents were often present and staying strong was a recurring theme across mental mindset and negotiating relationships.<br>HCP are critical to facilitate AYAC participation in day to day decision making by encouraging autonomy and with effective communication.
Sawyer (2019)	Developmentally Appropriate Care for Adolescents and Young Adults with Cancer: How Well is Australia Doing?	Australasia Multicentre	Quantitative – Chi-squared and Fisher's exact test<br><br>*Single time point survey.*	Explore quality of AYAC care in Australia.	196 participants:<br>**196 AYACs (age range: 15-25 years)**	>90% of AYACs reported positive responses for 11 of the 14 experience of care items which related to the quality of communication and general interactions with the cancer care team.<br>The most highly endorsed of these experiences of care items related to staff being friendly and respectful, communicating in ways that the AYAC understood, being supportive of AYACs asking questions and engaging families in discussion and decisions as the AYAC wished.<br>Older AYAC (20-25 years) report more empowerment to make decisions than younger AYACs and were more likely to report that healthcare professionals included their family in discussions and decision-making the way they wanted them to be included.

For peer review only

Sisk (2022)	Interdependent Functions of Communication with Adolescents and Young Adults in Oncology	USA Multicentre	Qualitative – content analysis *Semi-structured interviews.*	Define communication functions from perspective of AYACs.	37 participants: **37 AYACs (age range: 12-20 years; mean: 16 years)**	Building relationships: demonstrating clinical competence, reliability, empathy, and showing care and concern. When clinicians demonstrated these attributes, AYACs described feelings of trust in the clinicians' ability and intent to care for them.<br>Exchanging information: providing accurate and transparent information that was adapted to AYACs' needs. These needs related to the amount, complexity, timing, and pacing of information, and balancing communication between parents and AYACs.<br>AYACs think honesty and transparency is important. However, transparency could be burdensome.<br>Exploring uncertainties and fears of the future mad AYACs feel better prepared and decreasing anxiety. There was variation between AYACs for exploring these unknowns.<br>AYACs varied in their preferences in sharing distressing information and whether healthcare professionals should remain present and or give AYACs their privacy.<br>AYACs often feel that treatment related decisions realistically only have one choice giving a sense of powerlessness. They played a greater role in decisions outside of treatment related areas.<br>While some AYACs preferred very passive or active roles most described an interdependent process of communication involving them, their parents, and their clinicians.<br>Parents often served as a conduit and buffer of communication between the AYAC and healthcare professional. Many described the integral role of parents in communication regardless of their age.

For peer review only - http://bmjopen.bmj.com/site/about/guidelines.xhtml

Sisk (2022)	Co-management of communication and care in adolescent and young adult oncology	USA	Qualitative Semi-structured interviews	Study aimed to learn how AYAs and parents shared and delegated roles in communication and care during and after treatment for cancer	37 Participants:<br>37 AYAC aged 12-24<br>Mean age 16 years	There are 6 roles that AYAC co-manage with parents; managing information, managing social and emotional needs, managing health, advocacy and empowerment, making decisions and managing logistics.<br>Five factors that influence AYAC roles in communication were:<br>AYAC agency<br>Clinical encouragement<br>Emotional and physical well-being<br>Personality, preferences and values<br>Insights and skills<br><br>There are multiple benefits of engagement of the adolescent.
Viola (2018)	Problem-solving skills, parent–adolescent communication, dyadic functioning, and distress among adolescents with cancer	USA	Mixed methods study	The aim was to describe and assess how intrapersonal (i.e., problem-solving ability) and social–ecological factors (i.e., cancer- related communication with parents and parent–adolescent dyadic relationship quality) are associated with adolescent adjustment (i.e., distress).	78 participants:<br>39 AYAC and 39 parent pairs<br>AYAC 14-20 mean age 16.1<br>39 Parents - 79.5 % mothers	Better adolescent problem solving skills and better parent problem solving skills were associated with lower adolescent distress.<br>Parents and adolescents reported similar moderate levels of cancer related communication problems.<br>The most commonly endorsed cancer-related problem was "not talking about what to do if the AYAC got significantly worse'.<br>Parents reported better problem solving ability and better dyadic functioning than their adolescent.
Weaver (2016)	"Being a Good Patient" During Times of Illness as Defined by Adolescent Patients With Cancer	USA	Qualitative - semantic content analysis<br><br>Semi-structured interviews		40 participants<br>AYAC ages 12-19<br>Mean age of 15.5 years	The concepts of adherence and compliance were the primary phrases used to describe the good patient role, but always within the context of a relationship. Of note: A total of 23 adolescents requested to be interviewed alone with the interviewer (57.5%)

For peer review only

For peer review only - http://bmjopen.bmj.com/site/about/guidelines.xhtml

Weaver (2015)	Adolescents' Preferences for Treatment Decisional Involvement During Their Cancer	USA Multicentre	Qualitative – semantic content analysis<br><br>*Semi-structured interviews.*	Investigate AYACs' decision-making preferences and how supports and healthcare professionals can support involvement.	40 participants:<br>**40 AYACs (age range at interview: 12-18.9 years; 0.5-6 months from diagnosis/relapse)**<br><br>*NB: 34 AYACs primary diagnosis, 6 AYACs relapse.*	AYACs indicated a spectrum of preferred decisional roles, with the most common being an actively involved role (65%), although a shared decision-making approach was still valued.<br>AYACs recognized that situational and social contexts might shift their preferred level of involvement in medical decisions.<br>Although adolescents wanted to be involved in decisions, they also expressed an appreciation of family insight, parental presence, and clinician guidance.<br>AYACs can retrospectively identify their preferences for inclusion in medical decision-making, and even when preferring involvement, they value the input of trusted others.
Wu (2021)	Decisional conflicts, anxiety, and perceptions of shared decision-making in cancer treatment trajectory among adolescents with cancer: A longitudinal study	Taiwan	Qualitative. An explanatory mixed method was used, incorporating questionnaires and individual interviews.	To describe the perception on levels of decision-making during cancer treatment for adolescents with cancer and examine the trajectory of their decisional conflict	44 participants:<br>**22 AYAC** 11 male and 11 female<br>mean age 15.39<br>**22 Supporters**:<br>father n=1<br>mothers n=12<br>both n=6<br>other n=3	Different levels of participation in shared decision making (SDM) during the treatment trajectory were found.<br>Participants experienced the highest decisional conflict during diagnosis.<br>Roles in healthcare communication varied from direct participation to indirect involvement.<br>Overall, participants reported that doctors and parents decided their level of involvement, communication and or decision making.
Zarnegar et al (2018)	Recall of Fertility Discussion by Adolescent Female Cancer Patients: A Survey-Based Pilot Study	USA	Qualitative	To assess: recall of a fertility discussion, satisfaction with fertility knowledge, and identify factors that may influence recall.	19 participants:<br>**19 AYAC aged** 13-18 years and a mean age of 15.6	42% and 52% of AYAC did not recall discussion regarding treatment related infertility or fertility preservation during initial treatment planning.<br>63% of AYAC reported that parents made all or most of the decisions whereas 30.8% reported making decisions with parents.<br>Key Finding - A greater percentage of AYAC who reported making a joint decision with parents recalled 71% of fertility discussions than those who reported parents made most or all of medical decisions.

¥Age range at diagnosis is given except where this was not provided in the article in which alternative metrics are presented and this is highlighted.
AYA(C) = adolescent and young adult (with cancer); NOS = not otherwise specified; RM-ANOVA = repeated measures analysis of variance.

For peer review only - http://bmjopen.bmj.com/site/about/guidelines.xhtml

Supplementary File – Search strategy and history

Searches run November 2022

Limited to 2005 onwards, and to English language only.

For peer review only

For peer review only - http://bmjopen.bmj.com/site/about/guidelines.xhtml

Medline (via Ovid)

Ovid MEDLINE(R) and Epub Ahead of Print, In-Process, In-Data-Review & Other Non-Indexed Citations, Daily and Versions <1946 to November 23, 2022>

1 (Parent* or guardian* or mother* or father* or partner or wife or wives or husband* or boyfriend* or girlfriend* or sibling* or friend* or carer* or "third person" or caregiver* or "care-giver*" or spouse* or supporter* or support network*).ti,ab. or parents/ or fathers/ or mothers/ or spouses/ or caregivers/ or siblings/ or friends/ or legal guardians/ 1074121

2 (TYA cancer or TYA oncology or AYA cancer or AYA oncology or (young adult adj3 (cancer or oncology or leuk?em* or lymphom* or h?ematol*)) or ("teenage and young adult" adj3 cancer) or ("teenage and young adult" adj3 oncology) or (teenage* adj3 cancer) or (teenage* adj3 oncology) or (adolescen* adj3 cancer) or (adolescen* adj3 oncology) or (young people adj3 cancer) or (young people adj3 oncology) or ("teenage and young adult" adj3 leuk?emia*) or (teenage* adj3 leuk?emia*) or (adolescen* adj3 leuk?emia*) or (young people adj3 leuk?emia*) or (young adult adj3 leuk?emia*) or ("teenage and young adult" adj3 h?ematol*) or (teenage* adj3 h?ematol*) or (adolescen* adj3 h?ematol*) or (young people adj3 h?ematol*) or (young adult adj3 h?ematol*) or ("teenage and young adult" adj3 lymphom*) or (teenage* adj3 lymphom*) or (adolescen* adj3 lymphom*) or (young people adj3 lymphom*) or (young adult adj3 lymphom*)).ti,ab. or ((exp adolescent/ or exp young adult/) and exp neoplasms/) 333070

3 (Communicat* or Disclos* or inform* or Interact* or relationship* or Conversation* or Dialogue* or triad* or Interview* or consult* or decision making).ti,ab. or exp communication/ or exp disclosure/ or exp information dissemination/ or exp physician-patient relations/ 5715959

4 (affect* or effect* or influenc* or resultant or impact* or perception* or perspective* or encounter* or preference or opinion or involvement or occurance* or feel or "go through" or experienc*).ti,ab. 12406352

5 1 and 2 and 3 and 4 3380

6 limit 5 to (english language and yr="2005 -Current") 2715

For peer review only

Embase (via Ovid)

Embase <1974 to 2022 November 23>

1 (TYA cancer or TYA oncology or AYA cancer or AYA oncology or (young adult adj3 (cancer or oncology or leuk?em* or lymphom* or h?ematol*)) or ("teenage and young adult" adj3 cancer) or ("teenage and young adult" adj3 oncology) or (teenage* adj3 cancer) or (teenage* adj3 oncology) or (adolescen* adj3 cancer) or (adolescen* adj3 oncology) or (young people adj3 cancer) or (young people adj3 oncology) or ("teenage and young adult" adj3 leuk?emia*) or (teenage* adj3 leuk?emia*) or (adolescen* adj3 leuk?emia*) or (young people adj3 leuk?emia*) or (young adult adj3 leuk?emia*) or ("teenage and young adult" adj3 h?ematol*) or (teenage* adj3 h?ematol*) or (adolescen* adj3 h?ematol*) or (young people adj3 h?ematol*) or (young adult adj3 h?ematol*) or ("teenage and young adult" adj3 lymphom*) or (teenage* adj3 lymphom*) or (adolescen* adj3 lymphom*) or (young people adj3 lymphom*) or (young adult adj3 lymphom*)).ti,ab. or ((exp *adolescent/ or exp *young adult/) and exp *neoplasm/) 9638

2 (Communicat* or Disclos* or inform* or Interact* or relationship* or Conversation* or Dialogue* or triad* or Interview* or consult* or decision making).ti,ab. or exp *interpersonal communication/ or exp *professional-patient relationship/ or exp *information dissemination/ or exp *conversation/ 6997005

3 (Parent* or guardian* or mother* or father* or partner or wife or wives or husband* or boyfriend* or girlfriend* or sibling* or friend* or carer* or "third person" or caregiver* or "care-giver*" or spouse* or supporter* or support network*).ti,ab. or *parent/ or *father/ or *mother/ or *spouse/ or *caregiver/ or *social worker/ or *sibling/ or *friend/ or *legal guardian/ 1339977

4 (affect* or effect* or influenc* or resultant or impact* or perception* or perspective* or encounter* or preference or opinion or involvement or occurance* or feel or "go through" or experienc*).ti,ab. 15453173

5 1 and 2 and 3 and 4 939

6 limit 5 to (english language and yr="2005 -Current") 873

For peer review only

PsycInfo (via Ebscohost)

#	Query	Limiters/Expanders	Last Run Via	Results
S11	S1 AND S2 AND S7 AND S8	Limiters - Publication Year: 2005-2022 Expanders - Apply equivalent subjects Narrow by Language: - english Search modes - Boolean/Phrase	Interface - EBSCOhost Research Databases Search Screen - Basic Search Database - APA PsycInfo	1,683
S10	S1 AND S2 AND S7 AND S8	Expanders - Apply equivalent subjects Narrow by Language: - english Search modes - Boolean/Phrase	Interface - EBSCOhost Research Databases Search Screen - Basic Search Database - APA PsycInfo	1,981
S9	S1 AND S2 AND S7 AND S8	Expanders - Apply equivalent subjects Search modes - Boolean/Phrase	Interface - EBSCOhost Research Databases Search Screen - Basic Search Database - APA PsycInfo	2,017
S8	(affect* or effect* or influenc* or resultant or impact* or perception* or perspective* or encounter* or preference or opinion or involvement or occurance* or feel or "go through" or experienc*)	Expanders - Apply equivalent subjects Search modes - Boolean/Phrase	Interface - EBSCOhost Research Databases Search Screen - Basic Search Database - APA PsycInfo	3,366,619
S7	(S5) or (S3)	Expanders - Apply equivalent subjects Search modes - Boolean/Phrase	Interface - EBSCOhost Research Databases Search Screen - Basic Search Database - APA PsycInfo	13,719
S6	S4 AND S5	Expanders - Apply equivalent subjects	Interface - EBSCOhost Research Databases	13,275

For peer review only - http://bmjopen.bmj.com/site/about/guidelines.xhtml

		Search modes - Boolean/Phrase	Search Screen - Basic Search<br>Database - APA PsycInfo	
S5	((DE “neoplasms” OR DE "Benign Neoplasms" OR DE "Breast Neoplasms" OR DE "Endocrine Neoplasms" OR DE "Leukemias" OR DE "Melanoma" OR DE "Metastasis" OR DE "Nervous System Neoplasms" OR DE "Terminal Cancer"))	Expanders - Apply equivalent subjects<br>Narrow by SubjectAge: - adolescence (13-17 yrs)<br>Narrow by SubjectAge: - young adulthood (18-29 yrs)<br>Search modes - Boolean/Phrase	Interface - EBSCOhost Research Databases<br>Search Screen - Basic Search<br>Database - APA PsycInfo	13,275
S4	((DE “neoplasms” OR DE "Benign Neoplasms" OR DE "Breast Neoplasms" OR DE "Endocrine Neoplasms" OR DE "Leukemias" OR DE "Melanoma" OR DE "Metastasis" OR DE "Nervous System Neoplasms" OR DE "Terminal Cancer"))	Expanders - Apply equivalent subjects<br>Search modes - Boolean/Phrase	Interface - EBSCOhost Research Databases<br>Search Screen - Basic Search<br>Database - APA PsycInfo	58,767
S3	("TYA cancer" or "TYA oncology" or "AYA cancer" or "AYA oncology" or ("young adult" n3 (cancer or oncology or leuk?em* or lymphom* or h?ematol*)) or ("teenage and young adult" n3 cancer) or ("teenage and young adult" n3 oncology) or (teenage* n3 cancer) or (teenage* n3 oncology) or (adolescen* n3 cancer) or (adolescen* n3 oncology) or ("young people" n3 cancer) or ("young people" n3 oncology) or ("teenage and young adult" n3 leuk?emia*) or (teenage* n3 leuk?emia*) or (adolescen* n3 leuk?emia*) or ("young people" n3 leuk?emia*) or ("young adult" n3 leuk?emia*) or ("teenage and young adult" n3 h?ematol*) or (teenage* n3 h?ematol*) or (adolescen* n3 h?ematol*) or ("young people" n3 h?ematol*) or ("young adult" n3 h?ematol*) or ("teenage and young adult" n3 lymphom*) or (teenage* n3 lymphom*) or (adolescen* n3 lymphom*) or ("young people" n3 lymphom*) or ("young adult" n3 lymphom*))	Expanders - Apply equivalent subjects<br>Search modes - Boolean/Phrase	Interface - EBSCOhost Research Databases<br>Search Screen - Basic Search<br>Database - APA PsycInfo	1,864
S2	(Communicat* or Disclos* or inform* or Interact* or relationship* or Conversation* or Dialogue* or triad* or Interview* or consult* or "decision making") or DE “communication” OR DE “information dissemination” OR DE “conversation”	Expanders - Apply equivalent subjects<br>Search modes - Boolean/Phrase	Interface - EBSCOhost Research Databases<br>Search Screen - Basic Search<br>Database - APA PsycInfo	2,423,980
S1	(Parent* or guardian* or mother* or father* or partner or wife or wives or husband* or boyfriend* or girlfriend* or sibling* or friend* or teacher* or social worker* or carer* or "third	Expanders - Apply equivalent subjects<br>Search modes - Boolean/Phrase	Interface - EBSCOhost Research Databases<br>Search Screen - Basic Search	894,375

For peer review only - http://bmjopen.bmj.com/site/about/guidelines.xhtml

person" or caregiver* or "care-giver*" or spouse* or chaperone*) OR DE “parents” OR DE “mothers” OR DE “fathers” OR DE “spouses” OR DE “wives” OR DE “husbands” OR DE “siblings” OR DE “significant others” OR DE “social workers” OR DE “guardianship” OR DE “caregivers”		Database - APA PsycInfo

CINAHL (via Ebscohost)

Thursday, November 24, 2022 6:21:27 PM

#	Query	Limiters/Expanders	Last Run Via	Results
S7	S1 AND S2 AND S3 AND S4	Limiters - Published Date: 20050101-20221231<br>Expanders - Apply equivalent subjects<br>Narrow by Language: - english<br>Search modes - Boolean/Phrase	Interface - EBSCOhost Research Databases<br>Search Screen - Advanced Search<br>Database - CINAHL	1,837
S6	S1 AND S2 AND S3 AND S4	Limiters - Published Date: 20050101-20221231<br>Expanders - Apply equivalent subjects<br>Search modes - Boolean/Phrase	Interface - EBSCOhost Research Databases<br>Search Screen - Advanced Search<br>Database - CINAHL	1,866
S5	S1 AND S2 AND S3 AND S4	Expanders - Apply equivalent subjects<br>Search modes - Boolean/Phrase	Interface - EBSCOhost Research Databases<br>Search Screen - Advanced Search<br>Database - CINAHL	2,106

For peer review only - http://bmjopen.bmj.com/site/about/guidelines.xhtml

S4	(affect* or effect* or influenc* or resultant or impact* or perception* or perspective* or encounter* or preference or opinion or involvement or occurance* or feel or "go through" or experienc*)	Expanders - Apply equivalent subjects Search modes - Boolean/Phrase	Interface - EBSCOhost Research Databases Search Screen - Advanced Search Database - CINAHL	3,016,184
S3	("TYA cancer" or "TYA oncology" or "AYA cancer" or "AYA oncology" or ("young adult" n3 (cancer or oncology or leuk?em* or lymphom* or h?ematol*)) or ("teenage and young adult" n3 cancer) or ("teenage and young adult" n3 oncology) or (teenage* n3 cancer) or (teenage* n3 oncology) or (adolescen* n3 cancer) or (adolescen* n3 oncology) or ("young people" n3 cancer) or ("young people" n3 oncology) or ("teenage and young adult" n3 leuk?emia*) or (teenage* n3 leuk?emia*) or (adolescen* n3 leuk?emia*) or ("young people" n3 leuk?emia*) or ("young adult" n3 leuk?emia*) or ("teenage and young adult" n3 h?ematol*) or (teenage* n3 h?ematol*) or (adolescen* n3 h?ematol*) or ("young people" n3 h?ematol*) or ("young adult" n3 h?ematol*) or ("teenage and young adult" n3 lymphom*) or (teenage* n3 lymphom*) or (adolescen* n3 lymphom*) or ("young people" n3 lymphom*) or ("young adult" n3 lymphom*)) OR ((MH "adolescence+" OR MH "young adult+") AND (MH "neoplasms+"))	Expanders - Apply equivalent subjects Search modes - Boolean/Phrase	Interface - EBSCOhost Research Databases Search Screen - Advanced Search Database - CINAHL	59,927
S2	(Communicat* or Disclos* or inform* or Interact* or relationship* or Conversation* or Dialogue* or triad* or Interview* or consult* or "decision making") or MH "communication+" OR MH "discussion" OR MH	Expanders - Apply equivalent subjects Search modes - Boolean/Phrase	Interface - EBSCOhost Research Databases Search Screen - Advanced	2,016,086

For peer review only

	"conversation" OR (MH "Professional-Patient Relations+")		Search Database - CINAHL	
S1	(Parent* or guardian* or mother* or father* or partner or wife or wives or husband* or boyfriend* or girlfriend* or sibling* or friend* or teacher* or social worker* or carer* or "third person" or caregiver* or "care-giver*" or spouse* or chaperone*) OR MH "parents" OR MH "mothers" OR MH "fathers" OR MH "spouses" OR MH "siblings" OR MH "teachers" OR MH "social workers" OR MH "caregivers"	Expanders - Apply equivalent subjects Search modes - Boolean/Phrase	Interface - EBSCOhost Research Databases Search Screen - Advanced Search Database - CINAHL	544,991

Web of Science Core Collection

Web of Science Search Strategy (v0.1)

Database: Web of Science Core Collection

Entitlements:

- WOS.IC: 1993 to 2022
- WOS.CCR: 1985 to 2022
- WOS.SCI: 1900 to 2022
- WOS.AHCI: 1975 to 2022
- WOS.BHCI: 2008 to 2022
- WOS.BSCI: 2008 to 2022
- WOS.ESCI: 2017 to 2022
- WOS.ISTP: 1990 to 2022
- WOS.SSCI: 1956 to 2022
- WOS.ISSHP: 1990 to 2022

Searches:

1: TS=(Parent* or guardian* or mother* or father* or partner or wife or wives or husband* or boyfriend* or girlfriend* or sibling* or friend* or teacher* or social worker* or carer* or "third person" or caregiver* or "care-giver*" or spouse* or chaperone*)
Results: 2129759

2: TS=("TYA cancer" or "TYA oncology" or "AYA cancer" or "AYA oncology" or ("young adult" near/3 (cancer or oncology or leuk?em* or lymphom* or h?ematol*)) or ("teenage and young adult" near/3 cancer) or ("teenage and young adult" near/3 oncology) or (teenage* near/3 cancer) or (teenage* near/3 oncology) or (adolescen* near/3 cancer) or (adolescen*

For peer review only - http://bmjopen.bmj.com/site/about/guidelines.xhtml

near/3 oncology) or ("young people" near/3 cancer) or ("young people" near/3 oncology) or ("teenage and young adult" near/3 leuk?emia*) or (teenage* near/3 leuk?emia*) or (adolescen* near/3 leuk?emia*) or ("young people" near/3 leuk?emia*) or ("young adult" near/3 leuk?emia*) or ("teenage and young adult" near/3 h?ematol*) or (teenage* near/3 h?ematol*) or (adolescen* near/3 h?ematol*) or ("young people" near/3 h?ematol*) or ("young adult" near/3 h?ematol*) or ("teenage and young adult" near/3 lymphom*) or (teenage* near/3 lymphom*) or (adolescen* near/3 lymphom*) or ("young people" near/3 lymphom*) or ("young adult" near/3 lymphom*)) Results: 7793

3: TS=(Communicat* or Disclos* or inform* or Interact* or relationship* or Conversation* or Dialogue* or triad* or Interview* or consult* or "decision making") Results: 11889093

4: TS= (affect* or effect* or influenc* or resultant or impact* or perception* or perspective* or encounter* or preference or opinion or involvement or occurance* or feel or "go through" or experienc*) Results: 24306121

5: #4 AND #3 AND #2 AND #1 Results: 684

6: #4 AND #3 AND #2 AND #1 Results: 684

7: #4 AND #3 AND #2 AND #1 and 2005 or 2006 or 2007 or 2008 or 2009 or 2010 or 2011 or 2012 or 2013 or 2014 or 2015 or 2016 or 2017 or 2018 or 2019 or 2020 or 2021 or 2022 (Publication Years) Results: 644

8: #4 AND #3 AND #2 AND #1 and 2005 or 2006 or 2007 or 2008 or 2009 or 2010 or 2011 or 2012 or 2013 or 2014 or 2015 or 2016 or 2017 or 2018 or 2019 or 2020 or 2021 or 2022 (Publication Years) and English (Languages) Results: 619

For peer review only

PRISMA 2020 Checklist

Section and Topic	Item #	Checklist item	Location where item is reported
**TITLE**			
Title	1	Identify the report as a systematic review.	Page 1
**ABSTRACT**			
Abstract	2	See the PRISMA 2020 for Abstracts checklist.	Page 2
**INTRODUCTION**			
Rationale	3	Describe the rationale for the review in the context of existing knowledge.	Pages 3 and 4
Objectives	4	Provide an explicit statement of the objective(s) or question(s) the review addresses.	Page 4
**METHODS**			
Eligibility criteria	5	Specify the inclusion and exclusion criteria for the review and how studies were grouped for the syntheses.	Page 5
Information sources	6	Specify all databases, registers, websites, organisations, reference lists and other sources searched or consulted to identify studies. Specify the date when each source was last searched or consulted.	Page 4 and supplemental file
Search strategy	7	Present the full search strategies for all databases, registers and websites, including any filters and limits used.	Page 4, Table 1 and supplemental file 1
Selection process	8	Specify the methods used to decide whether a study met the inclusion criteria of the review, including how many reviewers screened each record and each report retrieved, whether they worked independently, and if applicable, details of automation tools used in the process.	Page 5
Data collection process	9	Specify the methods used to collect data from reports, including how many reviewers collected data from each report, whether they worked independently, any processes for obtaining or confirming data from study investigators, and if applicable, details of automation tools used in the process.	Pages 4 and 5
Data items	10a	List and define all outcomes for which data were sought. Specify whether all results that were compatible with each outcome domain in each study were sought (e.g. for all measures, time points, analyses), and if not, the methods used to decide which results to collect.	Page 5
	10b	List and define all other variables for which data were sought (e.g. participant and intervention characteristics, funding sources). Describe any assumptions made about any missing or unclear information.	Page 5
Study risk of bias assessment	11	Specify the methods used to assess risk of bias in the included studies, including details of the tool(s) used, how many reviewers assessed each study and whether they worked independently, and if applicable, details of automation tools used in the process.	Page 5
Effect measures	12	Specify for each outcome the effect measure(s) (e.g. risk ratio, mean difference) used in the synthesis or presentation of results.	Page 5
Synthesis methods	13a	Describe the processes used to decide which studies were eligible for each synthesis (e.g. tabulating the study intervention characteristics and comparing against the planned groups for each synthesis (item #5)).	Page 5
	13b	Describe any methods required to prepare the data for presentation or synthesis, such as handling of missing summary statistics, or data conversions.	Page 5
	13c	Describe any methods used to tabulate or visually display results of individual studies and syntheses.	Page 5
	13d	Describe any methods used to synthesize results and provide a rationale for the choice(s). If meta-analysis was performed, describe the model(s), method(s) to identify the presence and extent of statistical heterogeneity, and software package(s) used.	Page 5

For peer review only - http://bmjopen.bmj.com/site/about/guidelines.xhtml

PRISMA 2020 Checklist

Section and Topic	Item #	Checklist item	Location where item is reported
	13e	Describe any methods used to explore possible causes of heterogeneity among study results (e.g. subgroup analysis, meta-regression).	Pages 5 and 6
	13f	Describe any sensitivity analyses conducted to assess robustness of the synthesized results.	Not applicable
Reporting bias assessment	14	Describe any methods used to assess risk of bias due to missing results in a synthesis (arising from reporting biases).	Not applicable
Certainty assessment	15	Describe any methods used to assess certainty (or confidence) in the body of evidence for an outcome.	Not applicable
**RESULTS**			
Study selection	16a	Describe the results of the search and selection process, from the number of records identified in the search to the number of studies included in the review, ideally using a flow diagram.	Figure 1 page 6
	16b	Cite studies that might appear to meet the inclusion criteria, but which were excluded, and explain why they were excluded.	Figure 1 page 6
Study characteristics	17	Cite each included study and present its characteristics.	Table 2 – summary of articles pages
Risk of bias in studies	18	Present assessments of risk of bias for each included study.	Not reported
Results of individual studies	19	For all outcomes, present, for each study: (a) summary statistics for each group (where appropriate) and (b) an effect estimate and its precision (e.g. confidence/credible interval), ideally using structured tables or plots.	Not applicable
Results of syntheses	20a	For each synthesis, briefly summarise the characteristics and risk of bias among contributing studies.	Not applicable
	20b	Present results of all statistical syntheses conducted. If meta-analysis was done, present for each the summary estimate and its precision (e.g. confidence/credible interval) and measures of statistical heterogeneity. If comparing groups, describe the direction of the effect.	Not applicable
	20c	Present results of all investigations of possible causes of heterogeneity among study results.	Not applicable
	20d	Present results of all sensitivity analyses conducted to assess the robustness of the synthesized results.	Not applicable
Reporting biases	21	Present assessments of risk of bias due to missing results (arising from reporting biases) for each synthesis assessed.	Not applicable
Certainty of evidence	22	Present assessments of certainty (or confidence) in the body of evidence for each outcome assessed.	Not applicable
**DISCUSSION**			
Discussion	23a	Provide a general interpretation of the results in the context of other evidence.	Pages 10 and 11
	23b	Discuss any limitations of the evidence included in the review.	Pages 11 and 12

For peer review only

PRISMA 2020 Checklist

Section and Topic	Item #	Checklist item	Location where item is reported
	23c	Discuss any limitations of the review processes used.	Pages 11 and 12
	23d	Discuss implications of the results for practice, policy, and future research.	Page 12
**OTHER INFORMATION**			
Registration and protocol	24a	Provide registration information for the review, including register name and registration number, or state that the review was not registered.	Page 3
	24b	Indicate where the review protocol can be accessed, or state that a protocol was not prepared.	Supplemental file
	24c	Describe and explain any amendments to information provided at registration or in the protocol.	Not appliable
Support	25	Describe sources of financial or non-financial support for the review, and the role of the funders or sponsors in the review.	Page 13
Competing interests	26	Declare any competing interests of review authors.	Page 13
Availability of data, code and other materials	27	Report which of the following are publicly available and where they can be found: template data collection forms; data extracted from included studies; data used for all analyses; analytic code; any other materials used in the review.	Page 13

From: Page MJ, McKenzie JE, Bossuyt PM, Boutron I, Hoffmann TC, Mulrow CD, et al. The PRISMA 2020 statement: an updated guideline for reporting systematic reviews. BMJ 2021;372:n71. doi: 10.1136/bmj.n71

For more information, visit: http://www.prisma-statement.org/

For peer review only

BMJ Open

Triadic communication with teenagers and young adults with cancer: a systematic literature review: “Make me feel like I’m not the third person”

Journal:	*BMJ Open*
Manuscript ID	bmjopen-2023-080024.R1
Article Type:	Original research
Date Submitted by the Author:	10-Jan-2024

Complete List of Authors:	Critoph, Deborah; University of Cambridge, Department of Public Health & Primary Care<br>Taylor, Rachel; University College London Hospitals NHS Foundation Trust, CNMAR;<br>Spathis, Anna; University of Cambridge<br>Duschinsky, Robbie; University of Cambridge<br>Hatcher, Helen; Cambridge University Hospitals NHS Foundation Trust<br>Clyne, Ella; University of Cambridge<br>Kuhn, Isla; University of Cambridge, Medical Library, School of Clinical Medicine<br>Smith, Luke; Cambridge University Hospitals NHS Foundation Trust

**Primary Subject Heading**:	Communication
Secondary Subject Heading:	Communication, Oncology, Patient-centred medicine, Paediatrics, Medical education and training
Keywords:	Adolescents < Adolescent, Decision Making, EDUCATION & TRAINING (see Medical Education & Training), ONCOLOGY

SCHOLARONE™
Manuscripts

I, the Submitting Author has the right to grant and does grant on behalf of all authors of the Work (as defined in the below author licence), an exclusive licence and/or a non-exclusive licence for contributions from authors who are: i) UK Crown employees; ii) where BMJ has agreed a CC-BY licence shall apply, and/or iii) in accordance with the terms applicable for US Federal Government officers or employees acting as part of their official duties; on a worldwide, perpetual, irrevocable, royalty-free basis to BMJ Publishing Group Ltd ("BMJ") its licensees and where the relevant Journal is co-owned by BMJ to the co-owners of the Journal, to publish the Work in this journal and any other BMJ products and to exploit all rights, as set out in our licence.

The Submitting Author accepts and understands that any supply made under these terms is made by BMJ to the Submitting Author unless you are acting as an employee on behalf of your employer or a postgraduate student of an affiliated institution which is paying any applicable article publishing charge ("APC") for Open Access articles. Where the Submitting Author wishes to make the Work available on an Open Access basis (and intends to pay the relevant APC), the terms of reuse of such Open Access shall be governed by a Creative Commons licence – details of these licences and which Creative Commons licence will apply to this Work are set out in our licence referred to above.

Other than as permitted in any relevant BMJ Author's Self Archiving Policies, I confirm this Work has not been accepted for publication elsewhere, is not being considered for publication elsewhere and does not duplicate material already published. I confirm all authors consent to publication of this Work and authorise the granting of this licence.

For peer review only

Triadic communication with teenagers and young adults with cancer: a systematic literature review: "Make me feel like I'm not the third person"

Deborah J Critoph MSc, Rachel M Taylor PhD, Anna Spathis MD, Robbie Duschinsky PhD, Helen Hatcher MD PhD, Ella Clyne, Isla Kuhn MSc, Luke AM Smith

Deborah Critoph, PhD student, Department of Public Health & Primary Care, University of Cambridge

Rachel Taylor, Director of the CNMAR, Centre for Nurse, Midwife and Allied Health Profession Led Research, University College London Hospitals NHS Foundation Trust, London, NW1 2PG

Anna Spathis, Assistant Professor, Department of Public Health and Primary Care, University of Cambridge

Robbie Duschinsky, Professor of Social Science & Health, Department of Public Health & Primary Care, University of Cambridge

Helen Hatcher, Consultant in Medical and TYA Oncology, Clinical and Regional Lead for Sarcoma and Young People with Cancer, Clinical Director EoE TYAc ODN

Ella Clyne, Medical Student, University of Cambridge

Isla Kuhn, Head of Medical Library Services, University of Cambridge Medical Library

Luke Smith, Junior Clinical Fellow in TYA Haematology and Oncology, Cambridge University Hospitals NHS Foundation Trust

Corresponding author: Deborah Critoph, dc625@cam.ac.uk

Keywords: Teenagers and young adults with cancer (TYAC), adolescents, triadic communication, communication, supporters, third person, parents, support network, health care, professionals, experiences, impact.

Abstract = 290/300
Word count = 4,468
Number of tables = 4
Number of figures = 1

For peer review only - http://bmjopen.bmj.com/site/about/guidelines.xhtml

Abstract

Objectives
Clinical communication needs of teenagers and young adults with cancer (TYAC) are increasingly recognised to differ significantly from younger children and older adults. We sought to understand who is present with TYACs, TYACs experiences of triadic communication and its impact. We generated three research questions to focus this review:

1. Who is present with TYACs in healthcare consultations/communication?
2. What are TYACs' experiences of communication with the supporter present?
3. What is the impact of a TYAC's supporter being present in the communication?

Design
Systematic review with narrative synthesis.

Data sources
The search was conducted across six databases: Medline, CINAHL, Embase, PsycINFO, Web of Science and AMED for all publications up to December 2023.

Eligibility criteria for selecting studies
Included papers were empirical research published after 2005; participants had malignant disease, diagnosed aged 13-24 years (for over 50% of participants); the research addressed any area of clinical communication.

Data extraction and synthesis
Three independent reviewers undertook full text screening. A review-specific data extraction form was used to record participant characteristics and methods from each included paper and results relevant to the three review questions.

Results
A total of 8,480 studies were identified in the search, of which 36 fulfilled the inclusion criteria. We found that mothers were the most common supporter present in clinical communication encounters. TYACs experiences of triadic communication are paradoxical in nature – the supporter can help or hinder the involvement of the young person in care related communication. Overall, young people are not included in clinical communication and decisions at their preferred level.

Conclusion
Triadic communication in TYAC care is common, complex, and dynamic. Due to the degree of challenge and nuances raised, HCPs need further training on effective triadic communication.

PROSPERO registration number CRD42022374528

For peer review only

Strengths & limitations of this study

- We searched systematically and thoroughly for eligible studies, but this is not a well-indexed field of research, and therefore it is possible that some relevant studies were not included in the review.
- We limited the review to a UK TYAC age range and not the broader age used elsewhere, so the conclusions are applicable to younger adults, up to aged 24 only and not necessarily the age of young adulthood used in some countries (between 29 to 39).
- We only included papers published in English and the results may not be applicable to other countries especially where cultural differences affect parental-TYAC or other familial/romantic relational dynamics.
- International representation was seen in the eligible studies and TYAC ages were included across the entirety of the specified UK age range.
- Studies represented the journey throughout the cancer experience from diagnosis to survivorship and end of life care.

Introduction

Adolescence is a time of transition where young people navigate monumental physical, cognitive, emotional, and behavioural milestones to develop a sense of self-identity and gain independence. Although most young people have limited encounters with healthcare, around 2,500 young people in the United Kingdom (UK) are diagnosed with cancer each year, which is the leading cause of non-traumatic death in young people in the United States (US) and Europe.(1) Teenagers and young adults with cancer (TYACs) have unique healthcare needs and there has been an international drive to develop developmentally appropriate evidence-based specialist care, provided by appropriately trained healthcare professionals (HCPs).(2)

Communication with TYACs can be particularly challenging: a life-limiting condition intersects an age associated with emotional reactivity and variable maturity. TYACs clinical communication needs are increasingly recognised to differ significantly from younger children and older adults. Research indicates TYACs can have little meaningful involvement in conversations with HCPs: almost half of children and young people reported not being involved in decisions about their care.(3) HCPs recognise this and consider young people amongst the hardest patients to communicate with.(4) However, HCPs receive little training about how best to manage these clinical encounters. TYACs perceive that HCPs do not make efforts to understand how their cancer impacts their life outside of the healthcare setting. As a result, they may withdraw and subsequently be labelled as 'challenging', 'hard to reach' and 'disengaged'. This may adversely impact care and contribute to poor physical and psychological outcomes. Despite these issues, there are limited opportunities for formal postgraduate education in communication with TYACs for HCPs, with most training being ad hoc and not interprofessional.(5,6) Effective communication with TYACs has been recognised as a key national research priority. In a UK-wide survey of young patients' own research priorities, communication was a striking cross-cutting theme.(7)

Recent research into clinical communication with TYACs has offered some insight into the complexities of communication with this specialist patient group.(8–12) Yet one area that has

For peer review only - http://bmjopen.bmj.com/site/about/guidelines.xhtml

received less attention is triadic communication. Triadic communication refers to the presence of a third party, such as a parent, carer, or companion in clinical encounters (13) and the presence of such a person was found to occur in 87% of TYAC consultations.(11) As a commonly occurring form of communication in TYAC care, there is a need to understand the theoretical basis and relevance of triadic communication to clinical practice. For the purposes of this review, we refer to this third person as a supporter. Triadic communication literature from children and older adults exists. (14–17) Notably this includes a meta-analytic review of provider-patient-companion of adults,(18) one large systematic review of physician-patient-companion communication and decision-making in adults (19) and one review of doctor-parent-child communication.(20) Whilst informative, these studies are with children and adults, not this unique age-group of emerging adulthood with a significant life threatening diagnosis such as cancer. Also, these studies focus on doctor-patient-third person communication, whereas TYAC care involves a range of interdisciplinary professionals. This review aims to understand what is known about triadic communication with TYACs in healthcare communication.

Aim

We sought to understand who is present with TYACs, synthesise TYACs experiences of triadic communication with HCPs and supporter(s), and develop insights into the impact of triadic communication for TYACs.

Review questions:

1. Who is the supporter present with TYACs in healthcare consultations and communication?
2. What are TYACs' experiences of communication with the supporter present?
3. What is the impact on a TYAC's supporter being present in the communication?

Methods

We conducted a systematic review and narrative synthesis (21,22) of empirical evidence published since 2005, the year of publication of the National Institute for Care Excellence (NICE) Improving Outcomes Guidance, the guidance document underpinning TYAC services in England.(2) The review protocol was prospectively registered with PROSPERO (CRD42022374528). We designed the search to identify and map the available evidence using a broad scope to gain an overview of the pertinent literature, identify knowledge gaps and clarify concepts. The search strategy was developed and refined with an information scientist (I.K.). Keywords were generated across five strands detailed in Table 1, with strands combined with the Boolean operator 'AND'. The search was conducted across six databases: Medline, CINAHL, Embase, PsycINFO, Web of Science and AMED (supplemental file).

For peer review only - http://bmjopen.bmj.com/site/about/guidelines.xhtml

Table 1. Search Terms

Strand 1 – TYAC<br>TYA cancer or TYA oncology or teenage and young adult adj5 cancer or teenage and young adult adj5 oncology or teenage* adj5 cancer or teenage* adj5 oncology or adolescen* adj 5 cancer or adolescen* adj 5 oncology or young people adj 5 cancer or young people adj 5 oncology
Strand 2 – communication<br>Communication skills OR communicat* OR discuss* OR disclos* OR inform* OR interact OR relationship building OR decision making OR communication tools OR communication aids OR psychosocial assessment
Strand 3 – supporters<br>Parent* or guardian* or mother* or father* or partner or wife or wives or husband* or boyfriend* or girlfriend* or sibling* or friend* or carer* or "third person" or caregiver* or "care-giver*" or spouse* or supporter* or support network*.
Strand 4 - impact<br>affect OR effect OR influence OR result OR resultant OR impact
Strand 5 - experience<br>encounter OR involvement OR occurrence OR feel OR "go through" OR experience*

TYAC: teenage and young adult with cancer

Database searches were compiled and de-duplicated in Mendeley, abstracts were screened in Rayyan by two researchers (D.J.C and L.A.M.S.), and 172 full articles were read by three researchers (L.A.M.S., D.J.C., and R.M.T) for eligibility of inclusion in the final analysis, with disagreements resolved by discussion. Papers were included if: they presented empirical research published after 2005; participants had malignant disease, diagnosed aged 13-24 years (for over 50% of participants); the research addressed any area of clinical communication; and the research included supporters (parents, partners, carers, friends etc). Papers were excluded if they were: conference abstracts, unpublished articles, systematic reviews, single case studies, validation research methodology, studies using retrospective documentation in clinical notes, articles focusing on information needs rather than communication skills, or were not in English.

A review-specific data extraction form was used to record participant characteristics and methods from each included paper and results relevant to the three review questions. The final number of included articles totalled 36, the remaining 136 were excluded based on the participants' ages, focus on HCPs or information giving. In tandem to the data extraction process, two members of the review team (E.C. and D.J.C.) independently assessed each paper in terms of its internal validity, appropriateness, and contribution to answering the review questions, using a review-specific version of Gough's Weight of Evidence criteria.(23) Discrepancies in assessment decisions were discussed between reviewers and final scores were agreed through consensus.

Extracted data were entered into Excel to aid the narrative synthesis of the included papers.(21,22) All articles, irrespective of relevance and quality, were included in the review.

For peer review only - http://bmjopen.bmj.com/site/about/guidelines.xhtml

However, those rated ‘medium’ and ‘high’ were given greater weight in the synthesis. An inductive thematic analysis was undertaken to identify the main, recurrent, and important data across the studies related to answering each research question. D.J.C. and E.C. explored heterogeneity across the studies. The integration of results from studies utilising different methods and epistemological positions was supported by L.A.M.S. and R.M.T., and consensus in synthesis was reached. The synthesis was further refined through discussion of the review of results and their implications with clinicians, interdisciplinary academic audiences, and all of the co-authors.

Patient and Public Involvement Statement

None

For peer review only

Results

A total of 8,480 studies were identified in the search, of which 36 fulfilled the inclusion criteria (Figure 1). The included articles are summarised in Table 2. (table 2 uploaded separately)

All points across the cancer trajectory were represented in the final papers: diagnosis (n=7); (12,24–29) on treatment (n=17); (30–46) end of treatment (completed within one year) (n=2); (47,48) survivorship (more than one-year post-treatment) (n=2); (5,49) and end of life care (n=5). (50–54) Three studies included patients at more than one point along the cancer care continuum. (55–57) Most studies (n=19) were conducted in the US (24,27–29,31,35–37,39–46,50,52,54) other countries included the UK, (25,32,33) Australia, (38,48,49,57) Norway, (12,53) Israel, (47) Iran, (30) Mexico, (51) France, (34) Denmark, (26) Korea (56) and Taiwan, (55) one study recruited from three European countries. (5) Studies used predominantly qualitative methods (n=32) but there were two mixed methods studies and two using quantitative methods. Weight of evidence (WoE) criteria indicated five were high evidence, (24,31,35,45,56) twenty-four were medium (5,12,25,27–30,32–34,36,37,39–42,44,46,47,49–51,55,57) and seven were low evidence. (26,38,43,48,52–54) We used Gough’s review specific criteria to weight the quality of each paper. (23) To do this, we used three parameters:

A) The integrity of the evidence on its own terms
B) The appropriateness of the method for answering the review questions
C) The appropriateness of the focus or relevance for answering the review questions

Each of the above was either rated as low, medium, or high. These 3 parameters were combined to create WoE D which was the overall rating seen above and is the extent to which a study contributes evidence to answering the review questions. Factors that made the method highly appropriate included the use of semi-structured interviews to understand TYAC experiences and speaking to the TYAC and supporter separately. The high scoring papers included papers that focussed on communication in the triad, but this only occurred in 10 papers. In 9 papers the age at diagnosis was not specified and this decreased the weighting of these papers. (5,34–36,50–54)

Of the included studies just less than one third researched the triad (n=10) of TYAC, supporters and HCP (5,24,30–32,34–36,50,51), one third TYAC only (n=12)

(28,29,33,37,38,40–42,44–46,48) and just over a third TYAC and supporters (n=14). (12,25–27,39,43,47,49,52–57)

Table 3 Study population

Participants included in the study and numbers of papers included for each of the three participant groups

Triad? Dyad? Single?	Who is studied in the paper?	Number of papers
Triad	TYAC, supporter, HCA	10
Dyad	TYAC and supporter	14
Single	TYAC only	12

The categories used to separate the age groups were lower adolescence (11-14 years), middle adolescence (15-17 years), upper adolescence (18-21 years) and emerging adulthood (22 onwards). Of the papers where the age range at diagnosis could be deduced, the majority of these (21 out of 24) spanned three or more age categories. All the papers spanned two or more age categories. In nine of the papers, the age ranges at diagnosis were not available (as age at diagnosis was expressed as a mean or median). Given these factors, it is difficult to ascertain whether any between age group differences exist.

Who is present with TYACs in healthcare consultations and communication?

The majority of supporters were mothers (68.9%). When combined, parents represented nearly all the supporters in the included studies (94.6%), see Table 4. Non-parental supporters (1.8%) included partners, sisters, aunts, and grandmothers. The remaining supporters were not categorised due to insufficient information in the article's demographics data (3.9%).(53,54)

Table 4 Supporter Demographics

Details of the supporter demographics and percentages of within the included publications

Supporter type	Number of supporters	Percentage quoted to 1 decimal place (%)
**"Mother"**	453	68.9
**"Father"**	128	19.5
**"Both parents"**	20	3.0
**"Parents" no further specification**	20	3.0
**"Stepmother"**	1	0.2
**"Grandmother"**	2	0.3
**"Sister"**	3	0.5
**"Partner"**	3	0.5

For peer review only - http://bmjopen.bmj.com/site/about/guidelines.xhtml

**"Aunt"**	3	0.5
**"Supporters" no further specification**	21	3.2
**"Other"**	3	0.5
**Total**	657	100.1

What are TYACs' experiences of communication with the supporter present?

The presence of supporters was concurrently helpful and challenging for TYACs. Supporters undertook several helpful roles and responsibilities: they asked questions on behalf of the TYAC, retained information from HCPs, acted as a conduit of information between the TYAC and HCP, and acted as a ''sounding board'' for the young person.(25,31,45) Some supporters promoted self-advocacy and autonomy for the young person.(27,39,41,46,57) Some reported symptoms on their behalf (45) and proactively negotiated changes to treatment schedules in the interest of the young person.(39)

Findings also suggested that young people could experience limited or ineffective communication in the presence of a supporter. Communication could be directed towards the supporter, not the young person.(27,29,31,36) Supporters could receive information in the absence of the TYAC and subsequently filter the content before delivering the information to TYACs.(30,33,34,55,56): *"The parents had hidden a truth that was not theirs to hide"*p533.(34) This reflected the broader predicament that supporters' priorities at times might have competed with those of young people. (25,34,50,51) Supporters could dominate the communication encounter, for instance, parents were seen to interrupt young people, especially when time was limited. (51) Frederick et al found the mean time for adolescent to clinician communication was only 5.5% of the total consultation and parent conversation turns directed towards clinicians comprised a mean of 37.5% of all conversation turns. Clinicians directed most communication at the parent rather than the adolescent and spoke for 66.9% of the conversation and none of the clinicians offered patients the opportunity to speak with them alone. (35)

Mutual protectionism appeared to occur, with TYACs and supporters seeking to protect each other from difficult information leading to non-disclosure when both were present. A diagnosis of cancer is devastating for the young person, supporter(s), family, and the wider social network. Repeatedly, there were references to reduced disclosure between the young person and their supporter, in an attempt to shield each other from emotional distress.(12,31,36,38,39,41,45,53,56) TYACs could experience discomfort and guilt in seeing parents tearful and worried, and felt a burden in response to observing the emotions of supporters.(38,39,52) Some TYACs sought to limit this by withholding concerns to protect their supporters: "*I couldn't talk to mum about my concerns because I didn't want to hurt her*" p 37.(38) In equal measure, supporters were characterised as working hard to stay in control of emotions, be strong and stay in the "now", and they channelled energy into helping.(12,31,56) Yet this could contribute to an environment of non-disclosure that had the potential to create future communication challenges, such as supporters not knowing the young person's wishes. Examples of this were evident within the end of life care

studies.(52,53) Friebert et al found that 86% of young people wanted to receive prognostic information as soon as possible but only 39% of families knew that.(52) Similarly, Jacobs et al found that young people's end of life wishes were not known by their families.(53) In instances where the young person may not be able to communicate, it may help families relieve the impossible burden of making difficult decisions or feelings of regret, if the young person's perspective and wishes are known.(54)

What is the impact of a TYAC's supporter being present in the communication?

Supporters have the potential to facilitate, complicate or obstruct the young person's involvement in decision-making. Involvement had a positive impact on recall,(42) and may improve autonomy, efficacy, adherence, and future self-management.(24,57) However, the participation of supporters may be experienced as stressful by TYAC as they may become side-lined. (25,40,55) The presence of supporters impacted the young person's level of involvement in decision-making in several ways. In some cases, supporters empowered TYACs to make decisions by withholding their opinion (27) and deferring the final decision to TYACs.(31) However, supporters and TYACs did not perceive decision-making in the same way.(47,56) Supporters believed that young people oversaw decisions about their care; however, this was not what young people recounted.(24) TYACs reported a lack of communication and limited involvement in decisions (24,29,30,46) associated later with decisional regret.(24,37)

Deferral of communication and decisions from the young person to supporters was commonplace.(27,31,36) When supporters responded to this pathway of communication, young people then did not see a need to participate in decisions, knowing that their supporter was taking the mantle.(36) In parallel, clinicians were found to direct communication towards supporters and in extreme cases young people were completely excluded from communication and decisions. (29,30,35,47) An atmosphere characterised by a lack of trust, unanswered questions and uncertainty contributed to the exclusion of young people who then sought information from other sources.(30,36,39,56) Not allowing TYACs to choose their involvement in decision-making violated their autonomy, and increased distrust or resentment of providers and supporters and resulted in lower treatment adherence.(30,36,39)

The decisional involvement preferences of young people were not static: they were context and environment dependent. At diagnosis, heightened emotions and poor health rendered young people unable to engage in communication. (24,25,27,29,31,37,41) TYACs expressed a desire to be involved in decision making at different levels: some wanted limited involvement from their supporter(s) so they could take the leading role in consultations and their care;(58) several wanted collaboration with supporters and clinicians;(26,27,44,57) and some completely relied on supporters and HCP's to make decisions on their behalf.(45,46) Davies et al described this as agency, the ability to make free and independent choices. They highlighted the normality of this fluctuation between personal (acting independently), proxy (decisions made on behalf of someone) and collective (decisions are shared) decision making. Whilst this was not always linear, it was part of the cancer trajectory and demonstrated the fluctuating personal agency for TYACs.(32) Some young people reported that supporters and clinicians decided on the their level of involvement in communication and decision-

making,(55) and TYACs commented that they did not feel the decision was theirs.(47) Decisional involvement was an interactive, complex, and multifaceted process within the context of the triad, and young people often wanted to be in control of their level of involvement.(28,31) The evidence highlighted that in the presence of a supporter, young people's choice in the their level of involvement in decisions was challenged and not routinely achieved.

Most TYACs felt that it was important for the healthcare team to communicate with them directly and openly.(30,31,33,38,39,49,50) Time alone helped facilitate communication between TYAC and HCP, to ensure that the young person's needs were fully met.(31,36) However, time alone with HCPs was not routinely integrated as a part of consultations with TYACs**.** (35,48) In fact, clinicians were reported as frequently speaking more to parents and TYACs received limited communication from HCPs.(27,31,35,36) In the presence of supporters, as well as withholding concerning information, young people reported feeling discomfort when discussing sensitive topics such as sex or fertility preservation.(27,36)

Young people wanted time alone to communicate with HCPs directly for a variety of reasons. This private line of communication offered a sense of personal agency and allowed them to feel "in the loop" and promoted a sense of autonomy that was threatened by the cancer diagnosis, particularly at the point of diagnosis.(32,50) Young people wanted space to think and privacy during the cancer journey; private lines of communication with HCPs actively promoted this.(31,39,45,46) It also enabled HCPs to get to know the young person and allowed them to ask questions that they may be reluctant to ask in the presence of their supporter, because of embarrassment or emotional shielding.(31) Darabos et al found that 87.5% of oncology providers considered it important to talk to the TYAC without their parents present.(31) Whilst the importance has been highlighted within the data it is also evident that this does not happen as part of routine clinical practice. This could be for several reasons such as not wanting to challenge rules of authority, uncertainty around how best to ask a parent to leave and lack of confidence when communicating with a young person alone.

Discussion

Principal Findings

Who is present with TYACs in healthcare consultations and communication? For example, who are the supporters?

The included papers in our review demonstrated that most supporters were parents, more commonly mothers. The frequent presence of mothers in consultations is consistent with previous findings. For example, in a UK study in which TYAC nominated a caregiver, 85% were parents, and of those 80% were female.(59) We note that there is a paucity of data for non-parental supporters, and this may represent a reality of clinical practice or a bias towards TYAC-parental dyads over other relational-dyads in this field of research to date.

What are TYACs' s present?

For peer review only

TYACs experienced supporters facilitating communication by obtaining information, asking questions, advocating, and supporting personal agency of the young person; conversely supporters could hinder communication by gatekeeping information, or dominating communication and thereby rendering young people as bystanders. Young people experienced negative emotions in response to witnessing their supporters in distress.

What is the impact of a TYAC's supporter being present in the communication?

Bidirectional non-disclosure was a coping strategy used by both TYACs and supporters to protect one another from concerns and emotional burden. This limited HCPs ability to effectively assess ideas, concerns, and expectations from both parties when together. In the presence of supporters some young people were less informed, which could impair their ability to engage in decision-making conversations.

Meaning of the study

This is the first review to look specifically at triadic communication in teenagers and young adults with cancer and has demonstrated that there is a paucity of evidence focussed specifically on triadic communication with TYACs. Of the thirty-six studies in the review less than one third included all three parties in the triadic communication encounter. However, the review has enabled us to provide answers to the review questions and identify knowledge gaps, including a lack of theory describing triadic communication. Some preliminary theoretical models, such as family involvement in interpersonal healthcare processes,(60) depict the interaction pathways between patients, families and HCP and hypothesise the influence of family on interpersonal processes and outcomes of medical consultations.

The data has clearly identified that parents are the predominating supporter for TYACs, which may be surprising given the inclusion of participants up to the age of 25. Parents can play a significant role when a young person is diagnosed with cancer. Developmentally, a major characteristic that differentiates TYACs from younger children or older adults is the progressive increase in their desire and capacity for independence, personal agency, and autonomy. This process is disrupted by a cancer diagnosis: increased parental presence can be perceived as intrusive and reflect reversion to an earlier family dynamic, anchoring TYACs in dependency, restricting self-exploration, and limiting their developing of an internal value and belief system.(38,61–63) This has been phrased as 'retreating to family' and can negatively impact peer relationships by impeding development and maintenance of a peer network.(40,64,65) Young people may often be accepting of this, particularly in the early stages of the cancer diagnosis. However, as this review demonstrates, the presence of parents alters the experience and impact of communication with HCPs. It is important to highlight that there is limited literature on TYAC communication encounters with supporters other than parents.(62,66,67) Partners felt relegated to a non-participatory role by a parent, and mothers struggled to relinquish their existing role as primary supporter.(62,67) It is relevant to note that the participants in these three studies were in their early 20's.

A key impact of triadic communication is that young people may not be involved in decision making to the level they want. This is consistent with related paediatric oncology literature

For peer review only - http://bmjopen.bmj.com/site/about/guidelines.xhtml

which consistently reports children's limited participation in decision-making. (68–70) Clinicians attempted to protect children from 'too much' information because of the perception that children are not capable or too vulnerable.(17) The important difference between paediatric and TYAC populations are the legal and ethical obligations towards TYACs who are autonomous, capacitous patients rather than to parents with parental responsibility.

The findings of this review demonstrate the presence of a supporter impacts the involvement of young people in healthcare decisions. Therefore, there are legal and ethical issues, which are critically important, both in research and clinically in TYAC care particularly related to informed consent, capacity, and autonomy. The law relating to children and young people is complex and differs across the UK and internationally. The General Medical Council guidelines in the UK state, "the patient must be the first concern".(71) HCPs have ethical and legal obligations outlined in UK best practice guidance, statute, and case law.(72) In the UK, parents can legally make decisions for children under 16 years unless the child disagrees and is deemed 'Gillick Competent'.(73) Moreover, studies have shown children aged 14 and older can approach the level of understanding of adults.(74,75) In contrast, people aged 16 and above are legally able to make decisions for themselves in the UK and are automatically assumed to have capacity (76) and therefore, HCPs must communicate with them in developmentally appropriate ways. Clinicians face a challenge in identifying the best way to communicate with TYACs and their supporter (s). TYACs need parental involvement whilst simultaneously desiring autonomy (36) necessitating careful balancing of the needs of both parties to ensure that the young person is not relegated to a non-participant status.

Strengths and weaknesses

Our review had a number of limitations. We searched systematically and thoroughly for eligible studies, but this is not a well-indexed field of research, and therefore it is possible that some relevant studies were not included in the review. We limited the review to a UK TYAC age range and not the broader age used elsewhere, so the conclusions are applicable to younger adults, up to aged 24 only and not necessarily the age of young adulthood used in some countries (between 29 to 39). We also only included papers published in English and therefore papers reflect practices in primarily North America, Australia and Europe, the results may not be applicable to other countries especially where cultural differences affect parental-TYAC or other familial/romantic relational dynamics and where the healthcare culture may be different, e.g., more paternalistic. Despite these limitations, international representation was seen in the eligible studies, TYAC ages were included across the entirety of the specified UK age range and studies represented the journey throughout the cancer experience.

Implications for clinicians and policy makers

Given the degree of challenge and nuance raised, HCPs need training on effective triadic communication. Fourneret concluded that the relationship between TYACs, their parents and HCPs "as being the most difficult one in oncology".(34) Professionals described challenges communicating with both TYACs and parents, especially when loyalties were torn between the two.(5) However, training is currently ad hoc and not interdisciplinary.(77–80)

For peer review only - http://bmjopen.bmj.com/site/about/guidelines.xhtml

Furthermore, HCPs can find it difficult to apply teaching in this area in clinical practice.(53,81) HCPs need education and training to navigate triadic communication to optimise involvement of the young person whilst attending to a supporter's needs. Experiential learning is the gold standard in teaching methods for clinical communication and is designed to bring about changes in learners' skills. These evidence-based methods are through small group, problem-based simulation in a classroom, with repeated practise and rehearsal of skills under observation with detailed and descriptive feedback. This is arguably warranted here.(82,83)

Triadic communication is a key feature of TYAC care but requires further attention and inclusion in future iterations of key policy documents and guidelines such as the Blueprint of Care (BoC).(84) The BoC is a UK document that helps shape and deliver developmentally appropriate care to TYAC. However, it is recognised that age is poorly correlated with developmental maturity and therefore any communication framework needs to be specific to TYACs, recognising the transitional nature of adolescence meaning a one size fits all approach is likely inadequate.

Unanswered questions and future research

Future research is warranted to triangulate triadic perspectives and understand more about the interactional dynamics of these complex communication encounters. A key research need is investigating how best to support decision-making whilst engaging supporters, understanding their priorities and information needs may conflict.(31,36,37,40) Conflict management must also be understood in the emotional context of young adult oncology. How to effectively educate HCPs to communicate within the triad, to ensure the young person and the supporters needs are met is a priority. This needs to include how best we facilitate time alone between young patients and HCPs. Continued development and utilisation of comprehensive triadic theoretical frameworks may provide guidance and direction for future research, allowing for greater integration and progress with this diverse research area and commonly occurring form of healthcare communication.

Conclusion

Triadic communication is a pivotal component of communicating with TYACs and the presence of supporters impacts clinical communication both positively and negatively. Young people desire a sense of personal agency, autonomy and control related to information flow and decision making. This includes private lines of communication with HCPs without the presence of supporters. HCPs recognise the importance of time alone with young people; however this does not translate to clinical practice. Therefore, further research on communication dynamics is needed to allow for the development of bespoke, TYAC focussed clinical communication training for HCPs to allow them to effectively facilitate and navigate triadic communication. This then needs to be formally embedded in national guidance and postgraduate training for HCPs working in TYAC care to allow equitable access for TYACs.

For peer review only - http://bmjopen.bmj.com/site/about/guidelines.xhtml

Author Contributions:
DC, LS and RT were involved in developing the protocol; DC, IK and LS coordinated the running of the study and were responsible for data acquisition; DC, LS, RT and EC contributed to the analysis; DC drafted the manuscript. All authors have critically reviewed the manuscript for important intellectual content and have read and agreed to the published version of the manuscript. All authors agree to be accountable for all aspects of the work in ensuring that questions related to the accuracy or integrity of any part of the work are appropriately investigated and resolved.

Conflicts of Interest:
The authors declare no conflict of interest.

Ethics Approval:
This was a systematic review and therefore ethics approval was not required.

Funding:
This paper presents work supported by the Wellcome Trust, (grant number G115288) under its Programme PhD for healthcare professionals course awarded to the first author Deborah Critoph, University of Cambridge. R.M.T. is partially funded through UCLH Charity. The views expressed are those of the author (s) and not necessarily those of the Wellcome Trust or UCLH Charity.

Data availability statement:
All data relevant to the study are included in the article or uploaded as supplementary information. No previously unpublished primary data are included in the paper. All data relevant to the systematic review are included in the paper or uploaded as supplementary information.

Figure Legends:

Figure 1: PRISMA Flow diagram

Table 1: Search terms

Table 2: Summary of articles

Table 3: Study Population
Participants included in the study and numbers of papers included for each of the three participant groups

Table 4: Supporter Demographics
Details of the supporter demographics and percentages of within the included publications

For peer review only

References:

1. Cancer Research UK. Young peoples' cancers incidence statistics [Internet]. 2016 [cited 2019 Jul 10]. Available from: https://www.cancerresearchuk.org/health-professional/cancer-statistics/young-people-cancers/incidence
2. National Institute of Health and Clinical Excellence. Guidance on Cancer Services Improving Outcomes in Children and Young People with Cancer National Institute for Health and Clinical Excellence [Internet]. Nice. 2005. 198 p. Available from: https://www.nice.org.uk/guidance/csg7/resources/improving-outcomes-in-children-and-young-people-with-cancer-update-pdf-773378893
3. Care Quality Commission., NHS Patient Survey Programme. 2018 Children and Young People's patient experience survey. Statistical Release. 2019;(November).
4. White B, Viner RM. Improving communication with adolescents. Arch Dis Child Educ Pract Ed. 2012;97(3):93–7.
5. Essig S, Steiner C, Kuehni CE, Weber H, Kiss A. Improving Communication in Adolescent Cancer Care: A Multiperspective Study. Pediatr Blood Cancer [Internet]. 2016;63(8):1423–30. Available from: http://onlinelibrary.wiley.com/journal/10.1002/(ISSN)1545-5017
6. Lea S, Gibson F, Taylor RM. Holistic Competence": How Is it Developed, Shared, and Shaped by Health Care Professionals Caring for Adolescents and Young Adults with Cancer? J Adolesc Young Adult Oncol. 2021;10(5):503–11.
7. Gibson F, Aldiss S, Fern LA, Phillips B, Gravestock H, Malik S, et al. Reporting the whole story: Analysis of the "out-of-scope" questions from the James Lind Alliance Teenage and Young Adult Cancer Priority Setting Partnership Survey. Health Expect. 2021;
8. Smith LAM, Critoph DJ, Hatcher HM. How Can Health Care Professionals Communicate Effectively with Adolescent and Young Adults Who Have Completed Cancer Treatment? A Systematic Review. J Adolesc Young Adult Oncol [Internet]. 2020 Jan 14; Available from: https://doi.org/10.1089/jayao.2019.0133
9. Reid J, McKeaveney C, Martin P. Communicating with Adolescents and Young Adults about Cancer-Associated Weight Loss. Curr Oncol Rep. 2019;21(2):6.
10. Phillips CR, Haase JE. A Connectedness Primer for Healthcare Providers: Adolescents/Young Adult Cancer Survivors' Perspectives on Behaviors That Foster Connectedness during Cancer Treatment and the Resulting Positive Outcomes. J Adolesc Young Adult Oncol [Internet]. 2018;7(2):174–80. Available from: http://www.liebertpub.com/products/product.aspx?pid=387
11. Mellblom A V, Finset A, Korsvold L, Loge JH, Ruud E, Lie HC. Emotional concerns in follow-up consultations between paediatric oncologists and adolescent survivors: a video-based observational study. Psychooncology [Internet]. 2014 Dec;23(12):1365–72. Available from: https://search.ebscohost.com/login.aspx?direct=true&AuthType=athens&db=cin20&AN=109768272&site=ehost-live
12. Korsvold L, Mellblom AV, Finset A, Ruud E, Lie HC. A content analysis of emotional concerns expressed at the time of receiving a cancer diagnosis: An observational study of consultations with adolescent and young adult patients and their family members. European Journal of Oncology Nursing [Internet]. 2017;26:1–8. Available

For peer review only - http://bmjopen.bmj.com/site/about/guidelines.xhtml

from:
https://search.ebscohost.com/login.aspx?direct=true&AuthType=sso&db=cin20&AN=120589318&site=ehost-live&custid=ns123475
13. Ward A, Critoph D, Westacott R, Williams R, Dogra N. A collaboration on teaching and assessing triadic consultation skills. PEC Innovation [Internet]. 2022;1(October):100091. Available from: https://doi.org/10.1016/j.pecinn.2022.100091
14. van Staa AL. Unraveling triadic communication in hospital consultations with adolescents with chronic conditions: The added value of mixed methods research. Patient Educ Couns. 2011;82(3):455–64.
15. Cahill P, Papageorgiou A. Triadic communication in the primary care paediatric consultation: A review of the literature. British Journal of General Practice. 2007;57(544):904–11.
16. Maras M. Hidden in plain sight. Int J Psychiatry Med. 2022;57(6):481–5.
17. Young B, Dixon-woods M, Windridge KC, Heney D. Study of Patients and Parents. Br Med J. 2003;326(7384):305.
18. Wolff JL, Roter DL. Family presence in routine medical visits: A meta-analytical review. Soc Sci Med [Internet]. 2011;72(6):823–31. Available from: http://dx.doi.org/10.1016/j.socscimed.2011.01.015
19. Laidsaar-Powell RC, Butow PN, Bu S, Charles C, Gafni A, Lam WWT, et al. Physician-patient-companion communication and decision-making: A systematic review of triadic medical consultations. Patient Educ Couns. 2013;91(1):3–13.
20. Tates K, Meeuwesen L. Doctor-parent-child communication. A (re)view of the literature. Soc Sci Med. 2001;52(6):839–51.
21. Popay J, Roberts, H SA et al. Guidance on the Conduct of Narrative Synthesis in Systematic Reviews.
22. Petticrew M RH. Systematic Reviews in the social sciences. In: Systematic reviews in the social sciences [Internet]. Available from: https://onlinelibrary.wiley.com/doi/book/10.1002/9780470754887
23. Gough D. Weight of Evidence: a framework for the appraisal of the quality and relevance of evidence. 2007 [cited 2023 Jul 2]; Available from: https://www.tandfonline.com/action/journalInformation?journalCode=rred20
24. Barakat LP, Schwartz LA, Reilly A, Deatrick JA, Balis F. A Qualitative Study of Phase III Cancer Clinical Trial Enrollment Decision-Making: Perspectives from Adolescents, Young Adults, Caregivers, and Providers. J Adolesc Young Adult Oncol. 2014;3(1):3–11.
25. Hart RI, Cameron DA, Cowie FJ, Harden J, Heaney NB, Rankin D, et al. The challenges of making informed decisions about treatment and trial participation following a cancer diagnosis: a qualitative study involving adolescents and young adults with cancer and their caregivers. BMC Health Serv Res. 2020;20(1).
26. Ingersgaard MV, Tulstrup M, Larsen HB, Schmiegelow Kjeld AO - Ingersgaard MVO http://orcid. org/0000 0002 7037 2104. A qualitative study of decision-making on Phase III randomized clinical trial participation in paediatric oncology: Adolescents' and parents' perspectives and preferences. J Adv Nurs. 2018;74(1):110–8.
27. Olsavsky AL, Theroux CI, Dattilo TM, Klosky JL, O'Brien SH, Quinn GP, et al. Family communication about fertility preservation in adolescent males newly diagnosed with cancer. Pediatr Blood Cancer. 2021;68(7).

For peer review only - http://bmjopen.bmj.com/site/about/guidelines.xhtml

28. Weaver MS, Baker JN, Gibson D V, Gattuso JS, Hinds PS, Gibson D V, et al. "Being a good patient" during times of illness as defined by adolescent patients with cancer. Cancer [Internet]. 2016 Jul 15;122(14):2224–33. Available from: http://onlinelibrary.wiley.com/journal/10.1002/(ISSN)1097-0142
29. Mobley EM, Thomas SM, Brailsford J, Ochoa CY, Miller K, Applebaum A, et al. Clinical Trial Participation: A Qualitative Study of Adolescents and Younger Adults Recently Diagnosed with Cancer. J Adolesc Young Adult Oncol. 2023 Jun 1;12(3):303–13.
30. Bahrami M, Namnabati M, Mokarian F, Oujian P, Arbon P. Information-sharing challenges between adolescents with cancer, their parents and health care providers: a qualitative study. Supportive Care in Cancer [Internet]. 2017;25(5):1587–96. Available from: https://search.ebscohost.com/login.aspx?direct=true&AuthType=athens&db=cin20&AN=122279626&site=ehost-live
31. Darabos K, Berger AJ, Barakat LP, Schwartz LA. Cancer-Related Decision-Making Among Adolescents, Young Adults, Caregivers, and Oncology Providers. Qual Health Res. 2021 Nov 1;31(13):2355–63.
32. Davies J, Kelly D, Hannigan B. "Life then", "life interrupted", "life reclaimed": The fluctuation of agency in teenagers and young adults with cancer. Eur J Oncol Nurs. 2018;36(100885136):48–55.
33. Fern LA, Taylor RM, Whelan J, Pearce S, Grew T, Brooman K, et al. The Art of Age-Appropriate Care. Cancer Nurs [Internet]. 2013;36(5):E27–38. Available from: https://search.ebscohost.com/login.aspx?direct=true&AuthType=athens&db=cin20&AN=110243702&site=ehost-live
34. Fourneret E. Breaking Bad News about Cancer to Adolescents and Young Adults: The French Experience. J Law Med [Internet]. 2018 [cited 2021 Nov 22];25(2):530–7. Available from: https://pubmed.ncbi.nlm.nih.gov/29978652/
35. Frederick NN, Mack JW. Adolescent patient involvement in discussions about relapsed or refractory cancer with oncology clinicians. Pediatr Blood Cancer [Internet]. 2018;65(4):1. Available from: https://search.ebscohost.com/login.aspx?direct=true&AuthType=athens&db=cin20&AN=128132562&site=ehost-live
36. Hong MK, Wilcox L, Machado D, Olson TA, Simoneaux SF. Care Partnerships: Toward Technology to Support Teens' Participation in Their Health Care. Proc SIGCHI Conf Hum Factor Comput Syst [Internet]. 2016 May 7 [cited 2018 Jun 2];2016:5337–49. Available from: http://www.ncbi.nlm.nih.gov/pubmed/28164178
37. Mack JW, Fasciano KM, Block SD. Adolescent and Young Adult Cancer Patients' Experiences With Treatment Decision-making. Pediatrics. 2019;143(5).
38. Patterson P, Millar B, Desille N, McDonald F. The Unmet Needs of Emerging Adults With a Cancer Diagnosis A Qualitative Study. Cancer Nurs. 2012;35(3):E32–40.
39. Pennant S, Lee SC, Holm S, Triplett KN, Howe-Martin L, Campbell R, et al. The Role of Social Support in Adolescent/Young Adults Coping with Cancer Treatment. Children [Internet]. 2020;7(1):1–25. Available from: https://search.ebscohost.com/login.aspx?direct=true&AuthType=athens&db=cin20&AN=142468088&site=ehost-live
40. Pyke-Grimm KA, Franck LS, Halpern-Felsher B, Goldsby RE, Rehm RS. 3 Dimensions of Treatment Decision Making in Adolescents and Young Adults With Cancer. Cancer Nurs [Internet]. 2020 Nov;43(6):436–45. Available from:

For peer review only - http://bmjopen.bmj.com/site/about/guidelines.xhtml

https://search.ebscohost.com/login.aspx?direct=true&AuthType=athens&db=cin20&AN=146822019&site=ehost-live
41. Pyke-Grimm KA, Franck LS, Halpern-Felsher B, Goldsby RE, Rehm RS. Day-to-Day Decision Making by Adolescents and Young Adults with Cancer. Journal of Pediatric Hematology/Oncology Nursing [Internet]. 2022;39(5):290–303. Available from: https://ovidsp.ovid.com/athens/ovidweb.cgi?T=JS&CSC=Y&NEWS=N&PAGE=fulltext&D=medl&AN=35538622 https://cambridge-primo.hosted.exlibrisgroup.com/openurl/44CAM/44CAM_services_page?sid=OVID:medline&id=pmid:35538622&id=doi:10.1177%2F27527530211068718&issn=
42. Zarnegar S, Gosiengfiao Y, Rademaker A, Casey R, Albritton KH. Recall of Fertility Discussion by Adolescent Female Cancer Patients: A Survey-Based Pilot Study. J Adolesc Young Adult Oncol [Internet]. 2018 Apr;7(2):249–53. Available from: http://www.liebertpub.com/products/product.aspx?pid=387
43. Viola A, Taggi-Pinto A, Sahler OJZ, Alderfer MA, Devine KA, Ed M, et al. Problem-solving skills, parent-adolescent communication, dyadic functioning, and distress among adolescents with cancer. Pediatr Blood Cancer. 2018;65(5).
44. Weaver MS, Baker JN, Gibson D V, Gattuso JS, Sykes AD, Hinds PS. Adolescents' preferences for treatment decisional involvement during their cancer. Cancer [Internet]. 2015;121(24):4416–24. Available from: http://onlinelibrary.wiley.com/journal/10.1002/(ISSN)1097-0142
45. Sisk BA, Keenan M, Kaye EC, Baker JN, Mack JW, DuBois JM. Co-management of communication and care in adolescent and young adult oncology. Pediatr Blood Cancer [Internet]. 2022;69(10):e29813. Available from: https://ovidsp.ovid.com/athens/ovidweb.cgi?T=JS&CSC=Y&NEWS=N&PAGE=fulltext&D=mesx&AN=35719025 https://cambridge-primo.hosted.exlibrisgroup.com/openurl/44CAM/44CAM_services_page?sid=OVID:medline&id=pmid:35719025&id=doi:10.1002%2Fpbc.29813&issn=1545-500
46. Sisk BA, Keenan M, Schulz GL, Kaye E, Baker JN, Mack JW, et al. Interdependent functions of communication with adolescents and young adults in oncology. Pediatr Blood Cancer [Internet]. 2022; Available from: http://onlinelibrary.wiley.com/journal/10.1002/(ISSN)1545-5017
47. Barlevy D, Wangmo T, Ash S, Elger BS, Ravitsky V. Oncofertility decision making: Findings from Israeli adolescents and parents. J Adolesc Young Adult Oncol [Internet]. 2019;8(1):74–83. Available from: https://www.proquest.com/scholarly-journals/oncofertility-decision-making-findings-israeli/docview/2426222863/se-2?accountid=47868
48. Sawyer SM, McNeil R, Thompson K, Orme LM, McCarthy MAOSSM; O http://orcid.org/0000 0002 9095 358X, Sawyer SM, et al. Developmentally appropriate care for adolescents and young adults with cancer: how well is Australia doing? Supportive Care in Cancer [Internet]. 2019;27(5):1783–92. Available from: http://link.springer.de/link/service/journals/00520/index.htm
49. Ellis SJ, Wakefield CE, McLoone JK, Robertson EG, Cohn RJ. Fertility concerns among child and adolescent cancer survivors and their parents: A qualitative analysis. J Psychosoc Oncol. 2016;34(5):347–62.
50. Ananth P, Mun S, Reffat N, Li R, Sedghi T, Avery M, et al. A Stakeholder-Driven Qualitative Study to Define High Quality End-of-Life Care for Children With Cancer. J Pain Symptom Manage [Internet]. 2021;62(3):492–502. Available from:

For peer review only - http://bmjopen.bmj.com/site/about/guidelines.xhtml

https://ovidsp.ovid.com/athens/ovidweb.cgi?T=JS&CSC=Y&NEWS=N&PAGE=fulltext&D=med19&AN=33556497 https://cambridge-primo.hosted.exlibrisgroup.com/openurl/44CAM/44CAM_services_page?sid=OVID:medline&id=pmid:33556497&id=doi:10.1016%2Fj.jpainsymman.2021.01.

51. Cicero-Oneto CE, Valdez-Martinez E, Bedolla M. Decision-making on therapeutic futility in Mexican adolescents with cancer: a qualitative study. BMC Med Ethics [Internet]. 2017;18:74. Available from: https://search.ebscohost.com/login.aspx?direct=true&AuthType=athens&db=cin20&AN=127010047&site=ehost-live
52. Friebert S, Grossoehme DH, Baker JN, Needle J, Thompkins JD, Cheng YI, et al. Congruence Gaps Between Adolescents With Cancer and Their Families Regarding Values, Goals, and Beliefs About End-of-Life Care. JAMA Netw Open [Internet]. 2020 May 19;e205424–e205424. Available from: https://search.ebscohost.com/login.aspx?direct=true&AuthType=athens&db=cin20&AN=143389091&site=ehost-live
53. Jacobs S, Perez J, Cheng YI, Sill A, Wang J, Lyon ME. Adolescent end of life preferences and congruence with their parents' preferences: Results of a survey of adolescents with cancer. Pediatr Blood Cancer [Internet]. 2015;62(4):710–4. Available from: https://search.ebscohost.com/login.aspx?direct=true&AuthType=athens&db=cin20&AN=109777063&site=ehost-live
54. Lyon ME, Jacobs S, Briggs L, Cheng YI, Wang J. Family-centered advance care planning for teens with cancer. JAMA Pediatr. 2013;167(5):460–7.
55. Wu LM, Chiou SS, Lin PC, Liao YM, Su HL. Decisional conflicts, anxiety, and perceptions of shared decision-making in cancer treatment trajectory among adolescents with cancer: A longitudinal study. Journal of Nursing Scholarship. 2022;54(5):589–97.
56. Son H, Miller LE. Family Communication About Cancer in Korea: A Dyadic Analysis of Parent-Adolescent Conversation. Glob Qual Nurs Res. 2023 Jan 1;10.
57. Glackin A, Marino JL, Peate M, McNeil R, Orme LM, McCarthy MC, et al. Experiences of Oncofertility Decision-Making and Care in a National Sample of Adolescent and Young Adult Cancer Patients and Parents. J Adolesc Young Adult Oncol. 2023;
58. Mack JW, Fasciano KM, Block SD. Adolescent and Young Adult Cancer Patients' Experiences With Treatment Decision-making. Pediatrics. 2019;143(5).
59. Martins A, Alvarez-Galvez J, Fern LA, Vindrola-Padros C, Barber JA, Gibson F, et al. The BRIGHTLIGHT National Survey of the Impact of Specialist Teenage and Young Adult Cancer Care on Caregivers' Information and Support Needs. Cancer Nurs. 2021;44(3):235–43.
60. Dalton JM. Development and testing of the theory of collaborative decision-making in nursing practice for triads. J Adv Nurs. 2003;41(1):22–33.
61. Grinyer A. Young people living with cancer:implications for policy and practice. Open University Press; 2007. 182 p.
62. Moules NJ, Laing CM, Estefan A, Schulte F, Guilcher GMT. "Family Is Who They Say They Are"(a): Examining the Effects of Cancer on the Romantic Partners of Adolescents and Young Adults. J Fam Nurs. 2018;24(3):374–404.
63. Silva M, Barretta F, Luksch R, Terenziani M, Casanova M, Spreafico F, et al. Adolescents with cancer on privacy: Fact-finding survey on the need for confidentiality and space. Tumori. 2021 Oct 1;107(5):452–7.

For peer review only

64. KM B, Smith A, Schmidt S, TH K, Zebrack B, CF L, et al. Positive and negative psychosocial impact of being diagnosed with cancer as an adolescent or young adult. Cancer (0008543X) [Internet]. 2012 Oct 15;118(20):5155–62. Available from: https://search.ebscohost.com/login.aspx?direct=true&AuthType=athens&db=cin20&AN=104372613&site=ehost-live
65. Pyke-Grimm KA, Franck LS, Patterson Kelly K, Halpern-Felsher B, Goldsby RE, Kleiman A, et al. Treatment Decision-Making Involvement in Adolescents and Young Adults With Cancer. Oncol Nurs Forum [Internet]. 2019;46(1):E22–37. Available from: https://search.ebscohost.com/login.aspx?direct=true&AuthType=athens&db=cin20&AN=133553348&site=ehost-live
66. Davies J. The experience and role of partners in helping to meet the support needs of adolescents and young adults with cancer. Journal of Advanced Nursing (John Wiley & Sons, Inc) [Internet]. 2019 May;75(5):1119–25. Available from: https://search.ebscohost.com/login.aspx?direct=true&AuthType=athens&db=cin20&AN=135934208&site=ehost-live
67. Iannarino NT. "It's My Job Now, I Guess": Biographical disruption and communication work in supporters of young adult cancer survivors. Commun Monogr [Internet]. 2018;85(4):491–514. Available from: https://doi.org/10.1080/03637751.2018.1468916
68. Coyne I, Amory A, Gibson F, Kiernan G. Information-sharing between healthcare professionals, parents and children with cancer: More than a matter of information exchange. Eur J Cancer Care (Engl). 2016;25(1):141–56.
69. de Vries MC, Wit JM, Engberts DP, Kaspers GJL, van Leeuwen E. Pediatric Oncologists' Attitudes Towards Involving Adolescents in Decision-Making Concerning Research Participation. Pediatr Blood Cancer. 2010;55(1):123–8.
70. Frederick NN, Bingen K, Bober SL, Cherven B, Xu X, Quinn GP, et al. Pediatric oncology clinician communication about sexual health with adolescents and young adults: A report from the children's oncology group. Cancer Med [Internet]. 2021;10(15):5110–9. Available from: https://ovidsp.ovid.com/athens/ovidweb.cgi?T=JS&CSC=Y&NEWS=N&PAGE=fulltext&D=med20&AN=34128352 https://cambridge-primo.hosted.exlibrisgroup.com/openurl/44CAM/44CAM_services_page?sid=OVID:medline&id=pmid:34128352&id=doi:10.1002%2Fcam4.4077&issn=2045-76
71. General Medical Council. Guidance for All Doctors. 0-18 Years: Guidance for All Doctors [Internet]. 2018;11. Available from: https://www.gmc-uk.org/ethical-guidance/ethical-guidance-for-doctors/0-18-years/making-decisions#paragraph-29
72. British Medical Association. Children and young people toolkit A toolkit for doctors. BMA.org.uk [Internet]. 2021; Available from: https://www.bma.org.uk
73. Gillick T. Gillick competence What is Gillick Here ' s what you need to know about capacity and The Gillick case. 2022;
74. Joffe S, Fernandez C V., Pentz RD, Ungar DR, Mathew NA, Turner CW, et al. Involving children with cancer in decision-making about research participation. Journal of Pediatrics. 2006;149(6).
75. Mårtenson EK, Fägerskiöld AM. A review of children's decision-making competence in health care. J Clin Nurs. 2008;17(23):3131–41.
76. Department for Constitutional Affairs. Mental Capactity Act 2005: Code of Practice. Mental Capactity Act 2005: Code of Practice. 2007;1–301.

For peer review only - http://bmjopen.bmj.com/site/about/guidelines.xhtml

77. Lea S, Gibson F, Taylor RM. Holistic Competence": How Is it Developed, Shared, and Shaped by Health Care Professionals Caring for Adolescents and Young Adults with Cancer? J Adolesc Young Adult Oncol. 2021;10(5):503–11.
78. Essig S, Steiner C, Kuehni CE, Weber H, Kiss A. Improving Communication in Adolescent Cancer Care: A Multiperspective Study. Pediatr Blood Cancer. 2016;63(8):1423–30.
79. Essig S, Steiner C, Kühne T, Kremens B, Langewitz W, Kiss A. Communication Skills Training for Professionals Working with Adolescent Patients with Cancer Based on Participants' Needs: A Pilot. https://home.liebertpub.com/jayao. 2019 Jun 4;8(3):354–62.
80. Coad J, Smith J, Pontin D, Gibson F. Consult, Negotiate, and Involve: Evaluation of an Advanced Communication Skills Program for Health Care Professionals. Journal of Pediatric Oncology Nursing. 2018 Jul 1;35(4):296–307.
81. Ruhe KM, Badarau DO, Brazzola P, Hengartner H, Elger BS, Wangmo T, et al. Participation in pediatric oncology: views of child and adolescent patients. Psychooncology. 2016;25(9):1036–42.
82. Blackmore A, Kasfiki EV, Purva M. Simulation-based education to improve communication skills: A systematic review and identification of current best practice. BMJ Simul Technol Enhanc Learn. 2018;4(4):159–64.
83. Mahoney P, Macfarlane S, Ajjawi R. A qualitative synthesis of video feedback in higher education. Teaching in Higher Education [Internet]. 2019;24(2):157–79. Available from: https://doi.org/10.1080/13562517.2018.1471457
84. Smith S, Mooney S, Cable M, Taylor RM. THE BLUEPRINT OF CARE for teenagers and young adults with cancer Second edition. 2016; Available from: https://www.teenagecancertrust.org/sites/default/files/BlueprintOfCare_2ndEdition.pdf

For peer review only - http://bmjopen.bmj.com/site/about/guidelines.xhtml

PROSPERO
International prospective register of systematic reviews

National Institute for Health Research

University of York
Centre for Reviews and Dissemination

Systematic review

A list of fields that can be edited in an update can be found here

1. * Review title.

Give the title of the review in English

What is the impact and experience on communication for teenagers and young adults with cancer when supporters are present during healthcare consultations?

2. Original language title.

For reviews in languages other than English, give the title in the original language. This will be displayed with the English language title.

3. * Anticipated or actual start date.

Give the date the systematic review started or is expected to start.

05/12/2022

4. * Anticipated completion date.

Give the date by which the review is expected to be completed.

07/08/2023

5. * Stage of review at time of this submission.

This field uses answers to initial screening questions. It cannot be edited until after registration.

Tick the boxes to show which review tasks have been started and which have been completed.

Update this field each time any amendments are made to a published record.

The review has not yet started: Yes

For peer review only

PROSPERO
International prospective register of systematic reviews

Review stage	Started	Completed
Preliminary searches	No	No
Piloting of the study selection process	No	No
Formal screening of search results against eligibility criteria	No	No
Data extraction	No	No
Risk of bias (quality) assessment	No	No
Data analysis	No	No

Provide any other relevant information about the stage of the review here.

6. * Named contact.

The named contact is the guarantor for the accuracy of the information in the register record. This may be any member of the review team.

Deborah Critoph

Email salutation (e.g. "Dr Smith" or "Joanne") for correspondence:

Mrs Critoph

7. * Named contact email.

Give the electronic email address of the named contact.

dc625@medschl.cam.ac.uk

8. Named contact address

Give the full institutional/organisational postal address for the named contact.

Forvie Site

Addenbrookes Hospital

Department of Public Health and Primary Care

University of Cambridge

Cambridge

CB2 OPY

For peer review only

PROSPERO
International prospective register of systematic reviews

National Institute for Health Research

9. Named contact phone number.

Give the telephone number for the named contact, including international dialling code.

+44 (0)1223 330300

10. * Organisational affiliation of the review.

Full title of the organisational affiliations for this review and website address if available. This field may be completed as 'None' if the review is not affiliated to any organisation.

University of Cambridge

Organisation web address:

11. * Review team members and their organisational affiliations.

Give the personal details and the organisational affiliations of each member of the review team. Affiliation refers to groups or organisations to which review team members belong. **NOTE: email and country now MUST be entered for each person, unless you are amending a published record.**

Mrs Deborah Critoph. University of Cambridge
Dr Luke Smith. Cambridge University NHS Hospital Trust
Assistant/Associate Professor Rachel Taylor. University College London
Assistant/Associate Professor Anna Spathis. University of Cambridge
Assistant/Associate Professor Robbie Duschinsky. University of Cambridge
Dr Helen Hatcher. Cambridge University NHS Hospital Trust
Mrs Isla Kuhn. Cambridge University

12. * Funding sources/sponsors.

Details of the individuals, organizations, groups, companies or other legal entities who have funded or sponsored the review.

Wellcome Trust

Grant number(s)

State the funder, grant or award number and the date of award

Wellcome Trust Health Professionals PhD 01/10/2022 - 30/06/2026
Award number: 223497/Z/21/Z

13. * Conflicts of interest.

List actual or perceived conflicts of interest (financial or academic).

None

14. Collaborators.

Give the name and affiliation of any individuals or organisations who are working on the review but who are

For peer review only

not listed as review team members. **NOTE: email and country must be completed for each person, unless you are amending a published record.**

15. * Review question.

State the review question(s) clearly and precisely. It may be appropriate to break very broad questions down into a series of related more specific questions. Questions may be framed or refined using PI(E)COS or similar where relevant.

Overarching Theme

When supporters are present with teenagers and young adults with cancer what is the impact on the communication dynamics and the experiences of care for the young person?

Research Questions:

1. Who is present with teenagers and young adults with cancer in healthcare communication? For example, who are the supporters, how often do they attend and in what context?

2. What are the teenagers and young adults experiences of care with the supporter being present?

3. What is the impact of a supporter being present in the communication?

16. * Searches.

State the sources that will be searched (e.g. Medline). Give the search dates, and any restrictions (e.g. language or publication date). Do NOT enter the full search strategy (it may be provided as a link or attachment below.)

Searches will be run in:

- MEDLINE via Ovid

- Embase via Ovid

- CINAHL via EBSCO

- PsycINFO via EBSCO

- Web of Science Core Collection.

Searches will be limited by date, English language and human studies.

Inclusion criteria:

Participants had malignant disease (or BMT for non-malignant disease)

Diagnosis aged 13-24 at the time of the study (for over 50% participants)

Articles focussing on any area of clinical communication

Supporters - parents, romantic partners, friends, informal carers etc.

For peer review only

PROSPERO
International prospective register of systematic reviews

Papers published after 2005.

Exclusion criteria:

Non-English language

Systematic reviews

Conference abstracts

Articles focusing on information needs rather than communication skills.

Unpublished articles

Validation research methodology

Single case studies

Studies using retrospective documentation in clinical notes.

Additional search strategy information can be found in the attached PDF document (link provided below).

17. URL to search strategy.

Upload a file with your search strategy, or an example of a search strategy for a specific database, (including the keywords) in pdf or word format. In doing so you are consenting to the file being made publicly accessible. Or provide a URL or link to the strategy. Do NOT provide links to your search **results**.

https://www.crd.york.ac.uk/PROSPEROFILES/374528_STRATEGY_20221205.pdf

Alternatively, upload your search strategy to CRD in pdf format. Please note that by doing so you are consenting to the file being made publicly accessible.

Yes I give permission for this file to be made publicly available

18. * Condition or domain being studied.

Give a short description of the disease, condition or healthcare domain being studied in your systematic review.

The disease is a cancer diagnosis, this includes all oncological and haematological malignant diagnoses.

This is in the teenage and young adult cancer (TYAC) population, ages 13-24 birthday as per UK age ranges for the TYAC speciality.

19. * Participants/population.

Specify the participants or populations being studied in the review. The preferred format includes details of both inclusion and exclusion criteria.

Inclusion criteria:

Participants that had malignant disease (or BMT for non-malignant disease)

Diagnosis aged 13-24 at the time of the study (for over 50% participants)

Articles focussing on any area of clinical communication

Supporters - parents, romantic partners, friends, informal carers etc.

Papers published after 2005.

Exclusion criteria:

Non-English language

Systematic reviews

Conference abstracts

Articles focusing on information needs rather than communication skills.

Unpublished articles

Validation research methodology

Single case studies

Studies using retrospective documentation in clinical notes.

20. * Intervention(s), exposure(s).

Give full and clear descriptions or definitions of the interventions or the exposures to be reviewed. The preferred format includes details of both inclusion and exclusion criteria.

All interventions, any frameworks or assessment tools related to communication would be included in the review.

21. * Comparator(s)/control.

Where relevant, give details of the alternatives against which the intervention/exposure will be compared

PROSPERO
International prospective register of systematic reviews

National Institute for Health Research

(e.g. another intervention or a non-exposed control group). The preferred format includes details of both inclusion and exclusion criteria.

Any relevant comparator.

22. * Types of study to be included.

Give details of the study designs (e.g. RCT) that are eligible for inclusion in the review. The preferred format includes both inclusion and exclusion criteria. If there are no restrictions on the types of study, this should be stated.

We expect data returned will largely be qualitative, however will support the use of quantitative data where available.

These study designs that are not eligible for inclusion in the review include:

Systematic reviews, validation research methodology, single case studies and studies using retrospective documentation in clinical notes.

For peer review only

23. Context.

Give summary details of the setting or other relevant characteristics, which help define the inclusion or exclusion criteria.

Teenagers and young adults with cancer can be treated as in-patients and as outpatients and hospices, all settings will be included in the literature review. The cancer journey is complex including significant time points and transitions; at diagnosis, on treatment, relapse, completed treatment, survivorship and end of life care. All parts of the cancer journey if relevant will be included in the systematic review.

24. * Main outcome(s).

Give the pre-specified main (most important) outcomes of the review, including details of how the outcome is defined and measured and when these measurement are made, if these are part of the review inclusion criteria.

To gain a greater understanding of who the supporters may be when present with a TYAC. What context are they with the TYAC - when an inpatient, at outpatient appointments and what sort of roles do they play - active, passive, helpful, unhelpful, informational, emotional support, care.

To gain understanding of the interactional communication dynamics between TYAC, supporters and HCP communicating with them. What impact does this then have for the TYAC.

Measures of effect

Please specify the effect measure(s) for you main outcome(s) e.g. relative risks, odds ratios, risk difference, and/or 'number needed to treat.

Not applicable.

25. * Additional outcome(s).

List the pre-specified additional outcomes of the review, with a similar level of detail to that required for main outcomes. Where there are no additional outcomes please state ‘None’ or ‘Not applicable’ as appropriate to the review

None.

Measures of effect

Please specify the effect measure(s) for you additional outcome(s) e.g. relative risks, odds ratios, risk difference, and/or 'number needed to treat.

Not applicable.

26. * Data extraction (selection and coding). [1 change]

Describe how studies will be selected for inclusion. State what data will be extracted or obtained. State how this will be done and recorded.

All paper titles and abstracts will be assessed for eligibility by one independent reviewer, DC, and at least 25% of the papers (a random sample) assessed by a second independent reviewer, LS. The systematic literature review software Rayyan will be used to support this. Any papers where inclusion eligibility is unclear will be reviewed by a second independent reviewer with any disagreements on eligibility resolved by achieving consensus; a third independent reviewer will assess the eligibility of papers if needed. Full text review will be completed by the same three blinded reviewers and disagreements resolved by discussion.

Data extraction will be undertaken by one independent reviewer, DC using an Excel spreadsheet and reviewed by all paper authors. The data to be extracted will be:

1. Authors

2. Year of publication

3. Study location

4. Title

5. Study aims

6. Who were the supporters: a.) informal carers/support network b.) parents, romantic partners, friends, siblings.

7. Setting (treatment setting)

8. Point in the cancer journey: a.) pre diagnosis b.) diagnosis (up to 2 months) c.) treatment d.) survivorship (up to 2 years) e.) survivorship (up to 5 years) f.) survivorship (longer than 5 years)

9. Study type: a.) qualitative b.) quantitative c.) multi-method

PROSPERO
International prospective register of systematic reviews

National Institute for Health Research

10. Participant characteristics: a.) TYAC b.) HCP c.) supporters

11. Ages of patients at diagnosis – extract what information they give

12. Ages of patients at the time of the research – extract what information they give

13. Key findings

27. * Risk of bias (quality) assessment.

State which characteristics of the studies will be assessed and/or any formal risk of bias/quality assessment tools that will be used.

Qualitative articles will be assessed using the RATS guidelines (https://bmjopen.bmj.com/content/suppl/2012/01/12/bmjopen-2011-000138.DC1/BMJ_Open_IMG_Physician_Migration_RATS_Checklist.pdf).

Quantitative articles will be assessed using the Cochrane Risk of Bias/Comparison.

Studies will be assessed using the Mixed Methods Appraisal Tool (MMAT).

Quality assessment will be completed by three reviewers. Disagreements will be discussed for consensus.

Article quality and risk of bias will be included in the review publication.

28. * Strategy for data synthesis.

[1 change]

Describe the methods you plan to use to synthesise data. This **must not be generic text** but should be **specific to your review** and describe how the proposed approach will be applied to your data. If meta-analysis is planned, describe the models to be used, methods to explore statistical heterogeneity, and software package to be used.

We have planned a narrative synthesis. Study findings will be synthesised based on the reoccurring themes identified within included publications. This method will aid our inductive research synthesis and seek to generate new insights and recommendations in this previously unexplored area. Our approach will follow the four steps as outlined by Popay et al (2006):

• Exploring relationships in the data

• Developing a preliminary synthesis of findings of included studies

• Assessing the robustness of the synthesis

• Developing a theory of how the intervention works, why and for whom

Three researchers, DC, LS and RT, will analyse the extracted data from included papers. Data will be fully extracted into an Excel spreadsheet by DC as detailed in Q26, tabulating the study findings as the first step in the narrative synthesis of qualitative and quantitative data. This will allow us to answer research question one: Who are the supporters and in what context. By extracting data related to patient ages, we will also be

For peer review only

able to narratively synthase data for commonalities and differences by age categorised as per lower adolescence, middle adolescence, and upper adolescence. We hypothesise that as the age of the TYAC increases, the person in the role of a supporter may change from a parent to another important person. We will also be able to identify and describe if there is a difference in supporter, and role of supporter, at different time points in the cancer trajectory. We hypothesise that TYAC may need more from a supporter in the earlier stages of the cancer experience.

We are anticipating a reasonable number of publications in this review. It is likely that triadic communication is not the primary focus of most of the research studies, but may form part of the findings presented. We are also anticipating mainly qualitative research to detail experiences of communication, and our aim will be to draw the findings from a wide range of individual studies, with diverse methods, together to answer the research questions. We hypothesise that communication experiences for TYAC when a supporter is present are both helpful and unhelpful.

These steps detailed above will be used iteratively. The three researchers will all seek to undertake these steps and come together at regular intervals to review and debate insights, refine the narrative synthesis and reach conclusions by consensus. Researcher triangulation, of three unique backgrounds – doctor, nurse researcher in TYAC, and PhD student, will reduce researcher bias.

29. * Analysis of subgroups or subsets.

State any planned investigation of ‘subgroups’. Be clear and specific about which type of study or participant will be included in each group or covariate investigated. State the planned analytic approach.

It is likely that the final publications that meet the inclusion criteria will be including patients across a wider age range than this review is specifically looking at (13-24). It will therefore be critical to establish the focus within each study on the three subgroups of patient:
It is likely that we have this age range.

Lower adolescence - 13-16

Middle adolescence - 16-19

Upper adolescent - 19-24

There may be similarities in themes across the subgroups and differences and this will need to form part of the analysis and be presented clearly.

For peer review only

A supplementary table will be created to identify which age range each publication focussed on to aid accessibility for the reader.

30. * Type and method of review.

Select the type of review, review method and health area from the lists below.

Type of review

Cost effectiveness

No

Diagnostic

No

Epidemiologic

No

Individual patient data (IPD) meta-analysis

No

Intervention

No

Living systematic review

No

Meta-analysis

No

Methodology

No

Narrative synthesis

Yes

Network meta-analysis

No

Pre-clinical

No

Prevention

No

Prognostic

No

Prospective meta-analysis (PMA)

No

Review of reviews

No

For peer review only

Service delivery

No

Synthesis of qualitative studies

No

Systematic review

Yes

Other

No

Health area of the review

Alcohol/substance misuse/abuse

No

Blood and immune system

No

Cancer

Yes

Cardiovascular

No

Care of the elderly

No

Child health

Yes

Complementary therapies

No

COVID-19

No

Crime and justice

No

Dental

No

Digestive system

No

Ear, nose and throat

No

Education

No

For peer review only

PROSPERO
International prospective register of systematic reviews

National Institute for Health Research

Endocrine and metabolic disorders

No

Eye disorders

No

General interest

Yes

Genetics

No

Health inequalities/health equity

No

Infections and infestations

No

International development

No

Mental health and behavioural conditions

No

Musculoskeletal

No

Neurological

No

Nursing

No

Obstetrics and gynaecology

No

Oral health

No

Palliative care

No

Perioperative care

No

Physiotherapy

No

Pregnancy and childbirth

No

Public health (including social determinants of health)

Yes

Rehabilitation

For peer review only

PROSPERO
International prospective register of systematic reviews

No

Respiratory disorders

No

Service delivery

Yes

Skin disorders

No

Social care

No

Surgery

No

Tropical Medicine

No

Urological

No

Wounds, injuries and accidents

No

Violence and abuse

No

31. Language.

Select each language individually to add it to the list below, use the bin icon to remove any added in error.

English

There is not an English language summary

32. * Country.

Select the country in which the review is being carried out. For multi-national collaborations select all the countries involved.

England

33. Other registration details.

Name any other organisation where the systematic review title or protocol is registered (e.g. Campbell, or The Joanna Briggs Institute) together with any unique identification number assigned by them. If extracted data will be stored and made available through a repository such as the Systematic Review Data Repository (SRDR), details and a link should be included here. If none, leave blank.

34. Reference and/or URL for published protocol.

If the protocol for this review is published provide details (authors, title and journal details, preferably in

For peer review only

PROSPERO
International prospective register of systematic reviews

Vancouver format)

Add web link to the published protocol.

Or, upload your published protocol here in pdf format. Note that the upload will be publicly accessible.

Yes I give permission for this file to be made publicly available

Please note that the information required in the PROSPERO registration form must be completed in full even if access to a protocol is given.

35. Dissemination plans.

Do you intend to publish the review on completion?

Yes

Give brief details of plans for communicating review findings.?

This will be published in the peer reviewed journal, European Journal of Cancer Care with open access. The results of the literature review will also be shared at national level via the professional organisation - TYAC. It will be shared at international, national and local conferences and shared via OrcID, Research gate and professional twitter accounts to share this publication and findings widely.

36. Keywords.

Give words or phrases that best describe the review. Separate keywords with a semicolon or new line. Keywords help PROSPERO users find your review (keywords do not appear in the public record but are included in searches). Be as specific and precise as possible. Avoid acronyms and abbreviations unless these are in wide use.

Teenagers and young adults with cancer; TYAC; Adolescents; Triadic communication; Communication; Supporters; Third person; Parents; Support network; Health care professionals; Experiences; Impact

37. Details of any existing review of the same topic by the same authors.

If you are registering an update of an existing review give details of the earlier versions and include a full bibliographic reference, if available.

The systematic review that underpins this line of enquiry by three of the same authors can be found here:

DOI: 10.1089/jayao.2019.0133

This is not an update of an existing review but adding to the growing knowledge base related to

For peer review only

communicating with teenagers and young adults with cancer.

38. * Current review status.

Update review status when the review is completed and when it is published.New registrations must be ongoing so this field is not editable for initial submission.

Please provide anticipated publication date

Review_Ongoing

39. Any additional information. [1 change]

Provide any other information relevant to the registration of this review.

Please note this is a re-submission with revisions made to questions 26 and 28.

40. Details of final report/publication(s) or preprints if available.

Leave empty until publication details are available OR you have a link to a preprint (NOTE: this field is not editable for initial submission). List authors, title and journal details preferably in Vancouver format.

Give the link to the published review or preprint.

For peer review only

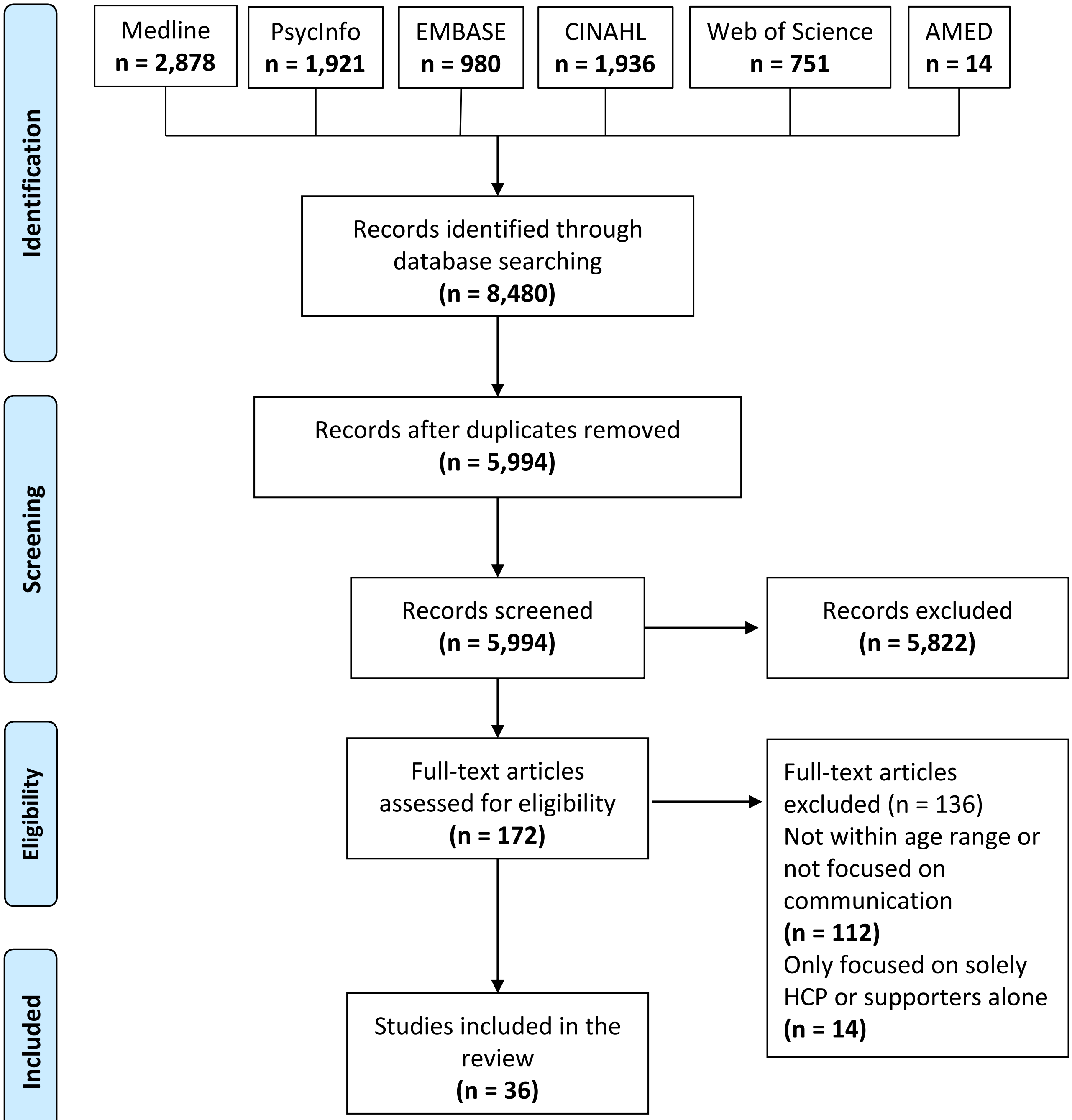
Identification
Screening
Eligibility
Included
Medline
n = 2,878
PsycInfo
n = 1,921
EMBASE
n = 980
CINAHL
n = 1,936
Web of Science
n = 751
AMED
n = 14
Records identified through database searching
(n = 8,480)
Records after duplicates removed
(n = 5,994)
Records screened
(n = 5,994)
Records excluded
(n = 5,822)
Full-text articles assessed for eligibility
(n = 172)
Full-text articles excluded (n = 136)
Not within age range or not focused on communication
(n = 112)
Only focused on solely HCP or supporters alone
(n = 14)
Studies included in the review
(n = 36)

Table 2. Summary of Articles

First author (year)	Title	Setting	Study Type - analysis method *Data collection*	Focus	Participant Characteristics	Key findings	WoE Score
Ananth (2021)	A Stakeholder-Driven Qualitative Study to Define High Quality End-Of-Life Care for Children with Cancer	USA Multicentre	Qualitative - thematic analysis *Semi-structured interviews and focus groups.*	To explore end of life care (EOLC) priorities for children with cancer and their families.	54 participants: **10 AYACs (age range: 17-23 years)** 25 parents (including 12 bereaved parents) 19 healthcare professionals	Important to have direct communication with the child or young person regarding decision-making. Interdisciplinary care with integrated teams is vital for high quality end of life care. Continuity of healthcare professionals was positive. AYACs would prefer to die at home but family and healthcare professionals may be hesitant.	Medium
Bahrami (2017)	Information Sharing Challenges Between Adolescents with Cancer, their Parents and Health Care Providers: A Qualitative Study	Iran Single centre	Qualitative descriptive-exploratory study - grounded theory analysis *Semi-structured interviews.*	Information sharing between AYACs, parents and health professionals.	33 participants: **12 AYACs (age range at interview: 15-20 years, within 1 year of diagnosis)** 6 supporters 6 healthcare professionals	AYACs feel they are excluded from information-sharing sessions between parents and healthcare professionals. This leads to disaffiliation, confusion and AYACs seek information from 'inferior' sources. Parents were often the first receivers of information allowing them to act as gatekeepers controlling information to flow to AYACs. Parents may want to shield AYACs from bad news. Trust and honesty are the foundations of effective communication between AYACs and healthcare professionals. AYACs reacted negatively towards dishonesty.	Medium
Barakat (2014)	A Qualitative Study of Phase III Cancer Clinical Trial Enrollment Decision Making Perspectives from AYAC, Caregivers and Providers	USA Single centre	Qualitative - thematic analysis *Semi-structured interviews.*	Clinical trial enrollment.	40 participants: **13 AYACs (age range: 15-21 years)** 16 supporters 11 healthcare professionals	Four patterns of decision-making patterns identified: 1. AYAC abdicates to caregiver, 2. Caregiver based and AYAC approved, 3. Collaborative, 4. AYAC in charge of decision-making. Caregivers perceived AYAC to be in charge of decision making most of the time whereas the AYACs felt that "AYAC abdicates to carer" was the most common form of decision making. Distress and poor health limited AYAC involvement in the decision. Developmental and emotional maturity facilitated involvement.	High

For peer review only - http://bmjopen.bmj.com/site/about/guidelines.xhtml

Barlevy (2019)	Oncofertility decision making: findings from Israeli adolescents and parents	Israel<br>Single centre	Qualitative- thematic analysis<br><br>*semi-structured interviews*	To understand adolescent oncofertilty decision making in Israel, from perspectives of parents and adolescents	35 participants<br>**16 AYACs (age range 12-16 years)**<br>19 parents	As in other cultural contexts, Israeli adolescents and parents demonstrate multifaceted decision making with respect to oncofertility. A significant finding from this study suggests that health professional shy from discussing posthumous planning of cryopreserved materials with adolescent cancer patients and their parents. 5 out of 16 AYAs felt that the decision was not theirs and that it was instead the parents' or the pysicians' to make. Some parents felt that the decision was made by the clinician - explicit or implicit recommendations from the clinician strongly influence decision making. No decisional regret expressed by any members of the dyad.	Medium
Cicero-Oneto (2017)	Decision-making on therapeutic futility in Mexican adolescents with cancer: a qualitative study	Mexico<br>Multicentre	Qualitative - thematic analysis<br><br>*Semi-structured interviews*	Decision making on therapeutic futility	32 Participants<br>13 paediatric oncologists<br>13 parents or primary carers<br>**6 AYACs (age range 13-18 years)**	Four themes were identified<br>1. flow of information to inform decision making<br>2. disclosure of prognosis<br>3. decision maker and stakeholder involved in decision making<br>4. barriers and facilitators to decision making<br>Differing values and agendas. The parents valued messages to "life the spirits" whereas the AYACs values honesty from the healthcare professionals<br>Gatekeeping of information.<br>Theme of "deference to authority"	Medium

For peer review only - http://bmjopen.bmj.com/site/about/guidelines.xhtml

For peer review only

Darabos (2021)	Cancer Related Decision Making Among AYAC, Care Givers and Oncology Providers	USA Single centre	Qualitative – content analysis with hypothesis coding<br><br>*Semi-structured interviews.*	Decision-making and triadic communication	30 Participants:<br>**11 AYACs (age range: 15-24 years)**<br>11 supporters<br>8 healthcare professionals	Four decision-making patterns were identified:<br>1. AYAC driven,<br>2. Collaborative,<br>3. Deferral to parents,<br>4. Provider-based, AYA/Caregiver-approved.<br>Collaborative decision-making and AYAC-driven decisions were most commonly described. There was recognition that some decision-making was day/context dependent.<br>AYACs were more likely to drive decisions regarding supportive care than treatment related decisions.<br>AYACs and caregivers explained how cognitive and emotional processes influenced cancer related decisions.<br>Emotional coping was more common than problem-based coping<br>Direct and honest communication contributes to a stronger relationship.<br>Individuality is key along with flexibility.<br>Time alone is important.	High
Davies (2019)	'Life then', 'life interrupted', 'life reclaimed': the fluctuation of agency in teenagers and young adults with cancer	UK single centre	qualitative - thematic analysis<br><br>*case studies - multiple interviews*	Fluctuation of agency across time and between cases	22 participants<br>**5 AYACs (16-24 years)**<br>**5 parents carers** (2 fathers, 3 mothers, 1 couple),<br>5 healthcare professionals (4 nurses and 1 oncology consultant)<br>5 other supporters (1 boyfriend, 1 girlfriend, 1 aunt, 2 friends)	Agency fluctuates over time within cases and between cases. Agency can fluctuate between personal, proxy and collective perspectives. Personal agency is high prior to diagnosis, decreases after diagnosis and is reclaimed after treatment.	Medium
Ellis (2016)	Fertility concerns among child and adolescent survivors and parents: a qualitative study	Australia single centre	Qualitative semi-structured telephone interviews	Fertility related themes with AYACs who are recently off treatment and with their parents	97 participants from 45 families<br>**19 AYACs (age range 7-17 at diagnosis, mean age 13.3)**<br>44 mothers and 34 fathers	Both parents and AYACs are concerned about the potential impacts on fertility of treatment. Poor doctor-patient communication was reported and conversations about fertility were frequently interrupted to discuss illness and treatment. These fertility discussions were not then continues once the AYAC was off treatment	Medium

For peer review only - http://bmjopen.bmj.com/site/about/guidelines.xhtml

Essig (2016)	Improving Communication in Adolescent Cancer Care: A Multiperspective Study	Germany, Austria, Switzerland<br><br>Number of centres not stated	Qualitative – <u>inductive thematic analysis</u><br><br>*Focus groups.*	Explore effective communication with AYACs for communication skills training.	<u>54 participants:</u><br>**16 AYACs (age range: 13-19 years)**<br>8 parents<br>30 healthcare professionals	Decision-making can cause conflict when adolescents are cognitively mature but legally lack the ability to make decisions.<br>AYACs feel a loss of autonomy.<br>Age-appropriate environments are important.<br>Effective communication differs depending on the type of professional (i.e., doctor vs nurse)<br>Adolescents negatively affect communication when:<br>1. They are indifferent.<br>2. There priorities conflict with treatment<br>3. They conflict with parents.<br>Healthcare professionals negatively affect communication when:<br>1. They don't treat the adolescent in an age-appropriate way.<br>2. They don't take the adolescent seriously.<br>3. They give too much information or withhold important information	Medium
Fern (2013)	The Art of Age-Appropriate Care	UK<br>Number of centres not stated	Qualitative – <u>thematic analysis</u><br><br>*Peer-to-peer interviews, field notes and spider diagrams from focus groups.*	Review a conceptual model of AYACs' cancer care experiences.	<u>11 participants:</u><br>**11 AYACs (age range: 13-25 years)**	Young people must be kept at the centre of interactions in recognition of their stated needs:<br>1. Engagement.<br>2. Individually tailored information.<br>3. Support unproxied by parents/family.<br>AYACs did not want information to be directed at parents but at them.<br>AYACs found it embarrassing when sensitive information was revealed in the presence of their parents.<br>Lack of continuity of healthcare professionals leads to AYACs dissatisfaction and irritation having to repeat their cancer story.	Medium

For peer review only - http://bmjopen.bmj.com/site/about/guidelines.xhtml

Fourneret (2018)	Breaking bad news about cancer to adolescents and young adults: the french experience	France Multicentre	qualitative semi-structured interviews	Explore the effectiveness and implementation of the French announcement protocol in 7 french paediatric oncology centres	<u>90 participants</u><br>**27 AYACs (21 were 14-17 and 6 were 18-22)**<br>30 parents (16 mothers, 5 fathers, 9 parents together at the appointment)<br>33 healthcare professionals	Parents and AYACs have different needs - both of which need to be accounted for when breaking bad news. Awkward and premature announcements were noted<br>The announcement consultation – young patients were never alone when informed of their disease; either with parents (n=31) (parents were informed before their child 10 out of 31 times - this was motivated by a compassionate goal of preparing the parents so they can better support the child when the bad news is broken) or close family member/sibling or boyfriend or girlfriend (n=2).<br>Some parents withheld info and some AYACs preferred parents not to know their diagnosis<br>Asymmetry in the triad discussed - but the key quality needed in the triad is mutual trust<br>HCP found parental presence helpful in the study. HCPs should show empathy (no neutrality) and attention to detail	Medium
Frederick (2018)	Adolescent Patient Involvement in Discussions About Relapsed or Refractory Cancer with Oncology Clinicians.	USA Single centre	Qualitative – <u>content analysis</u><br><br>*Audiotaped conversations.*	Breaking bad news of relapsed or refractory cancer.	<u>75 participants:</u><br>**11 AYACs (age range: 12.6-17.5 years)**<br>44 supporters<br>20 healthcare professionals	Adolescent patients' involvement in conversations about relapsed or refractory cancer is limited.<br>Adolescents were accompanied by one (27%) two (64%) or more than two (18%) family members in the discussion.<br>Adolescents spoke 3.5% of words compared to 66.9% clinicians and 30% parents.<br>No conversations included instances in which the clinicians' asked adolescents for their communication preferences or desired role in decision-making.	High
Friebert (2020)	Congruence gaps between adolescents with cancer and their families regarding values, goals and beliefs about end-of-life care	USA multicentre	*Qualitative cross-sectional study*	End of life care	<u>126 participants</u>:<br>**126 parent-AYAC dyads AYACs (14-20 years, mean age 16.9)**	Young people wanted early information (86%) but only 39% families knew this. Families understanding of what was important to their adolescents when dealing with their own dying was excellent for wanting honest answers from their physician and understanding treatment choices but poor for dying a natural death and being off machines that extend life, if dying.<br>Parents do not know what AYACs want at the end of life	Low

For peer review only - http://bmjopen.bmj.com/site/about/guidelines.xhtml

Glackin (2023)	Experiences of Oncofertility Decision-Making and Care in a National Sample of Adolescent and Young Adult Cancer Patients and Parents	Australasia multicentre	Qualitative – cross sectional survey. Reflexive thematic analysis	Oncofertility decision making	210 participants:<br>**99 AYACs (age range 15-25 years)**<br>111 parents<br>41 AYAC parent dyads from the same family	Four themes were identified: emotional care needs; parent-AYA dynamics including autonomy and agendcy; decision-making considerations including values and practicalities; and reflections on oncofertility and follow-up.<br>Both AYAC and parents placed importanceon AYA autonomy in fertility decision-making but, but many AYAs appreciated the role of parents in providing support and guidance throughout the process.<br>Healthcare professionals are encouraged to autonomously engage AYA's around fertility decision making, while concurrently offering opportunities that promotes parental support.	Low
Hart (2020)	The Challenges of Making Informed Decisions About Treatment and Trial Participation Following Cancer: A Qualitative Study with Adolescent and Young Adults with Cancer and Care Givers	UK Multicentre	Qualitative – thematic analysis<br><br>*Semi-structured interviews.*	Shared decision-making – primary treatment and trial participation – at diagnosis.	33 participants:<br>**18 AYACs (age range: 16-24 years)**<br>15 supporters	AYACs struggled to process information around diagnosis, exacerbated by symptom burden, emotions, and the fast pace of clinical activity.<br>Some AYACs disengaged from conversation topics which were distressing.<br>There are limited options for 'real' decision-making at diagnosis. However, many preferred this when they were already overwhelmed by emotions/symptoms.<br>For trial enrollment, many AYACs allowed themselves to be steered by the recommendation of the healthcare professional who recruited them, thinking they were acting in their best interests.	Medium
Hong (2016)	Care Partnerships: toward technology to support teen's participation in their health care	US multicentre	Qualitative semi-structured interviews and observations	To investigate how technology can support the partnerships between AYACs, parents and clinicians when the AYAC is experiencing complex chronic illness	33 interviews.<br>**15 with AYACs (13 of whom had cancer. age range 13-17)**<br>15 parents (10 mothers, 1 fathers, 1 aunt and 2 fathers and mothers together)<br>8 clinician caregivers	Participants faced challenges concerning:<br>1) Teens' limited participation in their care<br>2) communicating emotionally sensitive information<br>3) managing physical and emotional responses<br>Time alone with clinicians was important. Mutual protectionism or the need to "emotionally protect eachother" was prevalent.	Medium

For peer review only - http://bmjopen.bmj.com/site/about/guidelines.xhtml

Ingersgaard (2018)	A qualitative study on decision-making on Phase III randomized clinical trial participation in paediatric oncology: adolescents' and parents' perspectives and preferences	Denmark	qualitative exploratory study - in-depth semi-structured interviews with thematic analysis	To explore patients' and AYACs' motivs for accepting/ declining participation in the AL2008 trial and adolescents' involvement in decision making	16 participants<br>**5 AYACs (age range 12-16)**<br>6 parents of AYACs<br>5 parents of children aged 3-10 years with cancer	Key themes<br>1) altruism - wanting to help future AYACs<br>2) trust in the clinicians<br>3) individuals perceptions of cure contra toxicity<br>4) adolescents as active participants in the decision making process<br>5) parental responsibility and authority<br>6) the difficulty of uncertainty	Low
Jacobs (2015)	Adolescent end of life preferences and congruence with their parents' preferences: results of a survey of adolescents with cancer	Norway	Qualitative<br><br>three sessions of dyadic interviews	To explore AYACs' end of life preferences and to assess the congruence of these preferences with the parents' beliefs	17 adolescent/ family dyads<br>**17 AYACs (age range 14-21, 71% under 18)**	Adolescents with cancer were comfortable discussing EOL, and the majority preferred to talk about EOL issues before they are facing EOL. There were substantive areas of agreement between adolescents and their surrogates, but important facets of adolescents' EOL wishes were not known by their families, reinforcing the importance of eliciting individual preferences and engaging dyads so parents can understand their children's wishes. 53% of AYACs had never spoken about their end of life preferences but 82% considered it important to let their loved ones know their wishes.	Low

For peer review only

For peer review only - http://bmjopen.bmj.com/site/about/guidelines.xhtml

Korsvold (2017)	A content analysis of emotional concerns expressed at the time of receiving a cancer diagnosis: An observational study of consultations with adolescent and young adult patients and their family members	Norway	exploratory mixed methods study<br><br>audio recorded consultations	To investigate the emotional concerns of AYACs at the time of diagnosis and how to quantify how healthcare professionals respond	18 participants<br>**9 AYACs (age range 13-23)**<br>Present with mother (n=9), father (n=1), sister (n=1) or mother and father (n=2)	Four major themes of emotional concerns expressed by AYA patients and their family members during consultations for a cancer diagnosis:<br>1) side effects/late effects or infertility,<br>2) "what happens in the near future/practical aspects",<br>3) fear<br>4) sadness<br>AYA patients and family members expressed emotional concerns. HCPs typically responded by providing information, rather than affective aspects of the concerns In the sadness theme however, an explicit provide space affective response was the most common response (n=8) followed by an explicit provide space content response (n=7)<br>To make patients 'feel known' HCP should pay attention to the affective aspect of the expressed concern.	Medium
Lyon (2013)	Family-Centered Advance Care Planning for Teens With Cancer	USA single centre	Qualitative a randomised control pilot study	To examine the efficacy of family-centres ACP	30 dyads<br>**mean age of AYACs 16**<br>17 were randomised to intervention and 13 were randomised to control<br>87% of surrogates were biological parents and were female	The model (ACP) increased congruence in the triad compared to the control standard of care group– so it is key. The family centres ACP AYACs reported feeling more informed that the control group	Low

For peer review only - http://bmjopen.bmj.com/site/about/guidelines.xhtml

Mack (2019)	Adolescent and Young Adult Cancer Patients' Experiences with Treatment Decision-Making	USA<br>Single Centre	Quantitative – multivariate analysis, logistic regression<br><br>*Surveys at diagnosis, 4 and 12 months.*	Treatment decision-making	203 participants:<br>**203 AYACs (age range: 15-29 years)**	A majority of AYACs (58%) want to share decision-making with oncologists. The remainder were split between the AYAC wanting primary responsibility in decision-making (20%) or wanting their oncologist to have primary responsibility (22%).<br>A lower proportion of younger AYACs wanted sole responsibility but this did not achieve statistical significance (P = 0.07).<br>The majority (90%) of AYACs who lived with a parent/guardian wanted some form of input from their parents (either collaborative or considering their opinion).<br>Younger AYACs (15-17 years) were more likely to want greater involvement by their parents but were also more likely to be less involved than they wanted to be relative to their parents.<br>Decisional regret was less likely among AYACs who trusted oncologists completely, and who reported that oncologists understood what was important to them when treatment started.	Medium
Mobley (2023)	Clinical Trial Participation: A qualitative study of Adolescents and Younger Adults Recently Diagnosed with Cancer	USA	Qualitative<br>Grounded theory analysis of semi-structured interviews	Clinical trial participation	9 participants:<br>**9 AYACs (age range 16-20)**	Consent encompassed the first discussion of CCT. Patients reflected positive and negative effects of timing, decisional role, and emotional impact.<br>Informing participation involved decision-making processes, specific knowledge, understanding and external influence.<br>Participant relationships emphasized the importance of communication and relationships with providers and parents. Patient determinants centered on motives from different perspectives, pre-conceived attitudes, and understanding of CCTs.	Medium

For peer review only

Olsavsky (2021)	Family communication about fertility preservation in adolescent males newly diagnosed with cancer	USA	Qualitative	To explore fertility preservation communication among mothers, fathers and their male adolescents newly diagnosed with cancer.	87 participants:<br>**33 AYAC aged 12-25**<br>32 mothers<br>22 fathers<br><br>Representing 37 families in total.	Five process themes:<br>(1) Reliance on health care team and social support networks to facilitate FP decisions (noted just by parents),<br>(2) withholding parental opinion and deferring the decision to the adolescent,<br>(3) ease of communication,<br>(4) communication barriers and facilitators,<br>(5) not being present or not remembering details of FP conversations.<br>Four content themes:<br>(1) preference for biological parenthood (or grandparenthood),<br>(2) consideration of future partner of AYAC's desire for biological parenthood,<br>(3) sperm banking whilst it is a viable option,<br>(4) openness to alternative parenthood options	Medium

For peer review only - http://bmjopen.bmj.com/site/about/guidelines.xhtml

Patterson (2012)	The Unmet Needs of Emerging Adults With a Cancer Diagnosis	Australasia	Qualitative	Aim to contribute to the limited research base and inform our understanding of the needs of emerging adults with a diagnosis of cancer from a developmental perspective that appreciates the key transitional tasks of emerging adulthood identified by Arnett	14 Participants:<br>**14 AYAC aged 20-25**, average age of 22	A cancer experience poses the potential for significant impact on the four requirements for achievement of adulthood.<br><br>The needs of these emerging adults were grouped into six themes; information, healthcare provision, daily living, interpersonal support, identity renegotiation and emotional distress.<br><br>These themes relate directly to the four requirements of adulthood.<br>1. The task of accepting responsibility for oneself emphasises the importance of empowering AYAC in their communication with HCP.<br>2. The task of deciding on personal beliefs and values highlights the importance of keeping the AYAC informed and encouraging them in decisions giving maximum opportunity to explore beliefs.<br>3. Establishing a relationship with parents as equals highlights the importance of maximising AYAC autonomy in relation to supporters.<br>4. The task of becoming financially independent highlights the importance of minimising disruption to daily life.	Low

For peer review only - http://bmjopen.bmj.com/site/about/guidelines.xhtml

Pennant (2020)	The Role of Social Support in Adolescent/Young Adults Coping with Cancer Treatment	USA	Qualitative	To explore specific actions that help AYAC and what behaviours they want from their social supports	20 Participants:<br>**10 AYAC ages 15-26**, mean age 18.9 years<br>10 parents	Themes of support included; presence, distraction, positive attitude, maintaining AYAC autonomy, communication and advocacy.<br>Mothers were the most noted family support.<br>AYAC patients can differ in their preferences throughout treatment and this can, at times, appear contradictory.<br>AYACs appear to want autonomy and independence, but appreciate help with daily tasks from their parents.<br>They express the desire for privacy, but also value physical presence and communication<br>Parents must oscillate between being involved in and catering to their AYAC child's needs during treatment while allowing space for independence and autonomy.<br>The findings underscore the importance of maintaining open communication with AYAC patients about their preferences and needs throughout the course of treatment and asking them about both individual and social preferences, which may change frequently.	Medium
Pyke-Grimm (2020)	3 Dimensions of Treatment Decision Making in Adolescents and Young Adults with Cancer.	USA Multicentre	Qualitative – ethnographic<br><br>*Semi-structured interviews, field notes.*	Explore the preferences of AYACs for involvement in healthcare decisions	16 participants:<br>**16 AYACs (age range: 14.7-20 years)**	Emotions around diagnosis inhibit information receptiveness and ability to engage in treatment decisions (especially important decisions).<br>Initially AYACs struggle with the jargon and plethora of medical terms which are being used. They have limited knowledge which limits their questions, this increases over time.<br>The importance of decisions differs from one AYACs to the next. Also, some decisions are seen as having only one 'real' option.<br>AYACs engaged in minor decisions much earlier in their treatment, and some began engaging in more important decisions later in treatment.<br>AYACs could adopt an active (sole decision maker), collaborative (with healthcare professionals/supporters) or passive (healthcare professionals/supporters as decision makers) role.	Medium

For peer review only - http://bmjopen.bmj.com/site/about/guidelines.xhtml

Pyke-Grimm (2022)	Day-to-Day Decision Making by Adolescents and Young Adults with Cancer	USA	Qualitative Interpretive focused ethnography within the socio-logic tradition, informed by symbolic interactionism	To explore involvement of AYAs with cancer in day-to-day decisions affected by their cancer and treatment.	16 Participants: **16 AYAC aged 15-20 (at time of interview - with an average of one year from diagnosis)**	Factors influence the involvement of AYAC in decision making such as the type of decision, the point in the cancer journey. They want to be involved.<br>Four day to day decision making categories were identified: mental mindset, self care practices, self-advocacy and negotiating relationships.<br>Parents were often present and staying strong was a recurring theme across mental mindset and negotiating relationships.<br>HCP are critical to facilitate AYAC participation in day to day decision making by encouraging autonomy and with effective communication.	Medium
Sawyer (2019)	Developmentally Appropriate Care for Adolescents and Young Adults with Cancer: How Well is Australia Doing?	Australasia Multicentre	Quantitative – Chi-squared and Fisher's exact test<br><br>*Single time point survey.*	Explore quality of AYAC care in Australia.	196 participants: **196 AYACs (age range: 15-25 years)**	>90% of AYACs reported positive responses for 11 of the 14 experience of care items which related to the quality of communication and general interactions with the cancer care team.<br>The most highly endorsed of these experiences of care items related to staff being friendly and respectful, communicating in ways that the AYAC understood, being supportive of AYACs asking questions and engaging families in discussion and decisions as the AYAC wished.<br>Older AYAC (20-25 years) report more empowerment to make decisions than younger AYACs and were more likely to report that healthcare professionals included their family in discussions and decision-making the way they wanted them to be included.	Low

For peer review only - http://bmjopen.bmj.com/site/about/guidelines.xhtml

Sisk (2022)	Interdependent Functions of Communication with Adolescents and Young Adults in Oncology	USA Multicentre	Qualitative – <u>content analysis</u> *Semi-structured interviews.*	Define communication functions from perspective of AYACs.	<u>37 participants:</u> **37 AYACs (age range: 12-20 years; mean: 16 years)**	Building relationships: demonstrating clinical competence, reliability, empathy, and showing care and concern. When clinicians demonstrated these attributes, AYACs described feelings of trust in the clinicians' ability and intent to care for them.<br>Exchanging information: providing accurate and transparent information that was adapted to AYACs' needs. These needs related to the amount, complexity, timing, and pacing of information, and balancing communication between parents and AYACs.<br>AYACs think honesty and transparency is important. However, transparency could be burdensome.<br>Exploring uncertainties and fears of the future mad AYACs feel better prepared and decreasing anxiety. There was variation between AYACs for exploring these unknowns.<br>AYACs varied in their preferences in sharing distressing information and whether healthcare professionals should remain present and or give AYACs their privacy.<br>AYACs often feel that treatment related decisions realistically only have one choice giving a sense of powerlessness. They played a greater role in decisions outside of treatment related areas.<br>While some AYACs preferred very passive or active roles most described an interdependent process of communication involving them, their parents, and their clinicians.<br>Parents often served as a conduit and buffer of communication between the AYAC and healthcare professional. Many described the integral role of parents in communication regardless of their age.	High

For peer review only - http://bmjopen.bmj.com/site/about/guidelines.xhtml

Sisk (2022)	Co-management of communication and care in adolescent and young adult oncology	USA	Qualitative Semi-structured interviews	Study aimed to learn how AYAs and parents shared and delegated roles in communication and care during and after treatment for cancer	<u>37 Participants:</u><br>**37 AYAC aged 12-24**<br>**Mean age 16 years**	There are 6 roles that AYAC co-manage with parents; managing information, managing social and emotional needs, managing health, advocacy and empowerment, making decisions and managing logistics.<br>Five factors that influence AYAC roles in communication were:<br>AYAC agency<br>Clinical encouragement<br>Emotional and physical well-being<br>Personality, preferences and values<br>Insights and skills<br><br>There are multiple benefits of engagement of the adolescent.	Medium
Son (2023)	Family Communication About Cancer in Korea: A Dyadic Analysis of Parent-Adolescent Conversation	Korea	Qualitative descriptive study	The aim was to understand communication experiences of Korean AYAC and their parents in the context of young adult cancer.	<u>14 participants:</u><br>**7 AYAC (ages 14-19 years)** and 7 parent pairs	The main theme was "experience the same thing but see it differently" along with three subthemes.<br>Different expectations for parent-adolescent communication, different views on communication challenges and limited sharing and progress in the conversation.<br>This study offers insights into different communication expectations and preferences between Korean adolescents and parents, and reasons for communication challenges, while emphasizing the individualized assessment of parent-adolescent communication between them.	Medium

For peer review only - http://bmjopen.bmj.com/site/about/guidelines.xhtml

Viola (2018)	Problem-solving skills, parent–adolescent communication, dyadic functioning, and distress among adolescents with cancer	USA	Mixed methods study	The aim was to describe and assess how intrapersonal (i.e., problem-solving ability) and social–ecological factors (i.e., cancer- related communication with parents and parent–adolescent dyadic relationship quality) are associated with adolescent adjustment (i.e., distress).	<u>78 participants:</u><br>39 AYAC and 39 parent pairs<br>**AYAC 14-20 mean age 16.1**<br>39 Parents - 79.5 % mothers	Better adolescent problem solving skills and better parent problem solving skills were associated with lower adolescent distress.<br>Parents and adolescents reported similar moderate levels of cancer related communication problems.<br>The most commonly endorsed cancer-related problem was "not talking about what to do if the AYAC got significantly worse'.<br>Parents reported better problem solving ability and better dyadic functioning than their adolescent.	Low
Weaver (2016)	"Being a Good Patient" During Times of Illness as Defined by Adolescent Patients With Cancer	USA	Qualitative - <u>semantic content analysis</u><br><br>Semi-structured interviews		<u>40 participants</u><br>**AYAC ages 12-19**<br>Mean age of 15.5 years	The concepts of adherence and compliance were the primary phrases used to describe the good patient role, but always within the context of a relationship. Of note: A total of 23 adolescents requested to be interviewed alone with the interviewer (57.5%)	Medium
Weaver (2015)	Adolescents' Preferences for Treatment Decisional Involvement During Their Cancer	USA Multicentre	Qualitative – <u>semantic content analysis</u><br><br>*Semi-structured interviews.*	Investigate AYACs' decision-making preferences and how supports and healthcare professionals can support involvement.	<u>40 participants:</u><br>**40 AYACs (age range at interview: 12-18.9 years; 0.5-6 months from diagnosis/relapse)**<br><br>*NB: 34 AYACs primary diagnosis, 6 AYACs relapse.*	AYACs indicated a spectrum of preferred decisional roles, with the most common being an actively involved role (65%), although a shared decision-making approach was still valued.<br>AYACs recognized that situational and social contexts might shift their preferred level of involvement in medical decisions.<br>Although adolescents wanted to be involved in decisions, they also expressed an appreciation of family insight, parental presence, and clinician guidance.<br>AYACs can retrospectively identify their preferences for inclusion in medical decision-making, and even when preferring involvement, they value the input of trusted others.	Medium

For peer review only - http://bmjopen.bmj.com/site/about/guidelines.xhtml

Wu (2021)	Decisional conflicts, anxiety, and perceptions of shared decision-making in cancer treatment trajectory among adolescents with cancer: A longitudinal study	Taiwan	Qualitative. An explanatory mixed method was used, incorporating questionnaires and individual interviews.	To describe the perception on levels of decision-making during cancer treatment for adolescents with cancer and examine the trajectory of their decisional conflict	44 participants:<br>**22 AYAC** 11 male and 11 female<br>mean age 15.39<br>**22 Supporters**:<br>father n=1<br>mothers n=12<br>both n=6<br>other n=3	Different levels of participation in shared decision making (SDM) during the treatment trajectory were found.<br>Participants experienced the highest decisional conflict during diagnosis.<br>Roles in healthcare communication varied from direct participation to indirect involvement.<br>Overall, participants reported that doctors and parents decided their level of involvement, communication and or decision making.	Medium
Zarnegar et al (2018)	Recall of Fertility Discussion by Adolescent Female Cancer Patients: A Survey-Based Pilot Study	USA	Qualitative	To assess: recall of a fertility discussion, satisfaction with fertility knowledge, and identify factors that may influence recall.	19 participants:<br>**19 AYAC aged** 13-18 years and a mean age of 15.6	42% and 52% of AYAC did not recall discussion regarding treatment related infertility or fertility preservation during initial treatment planning.<br>63% of AYAC reported that parents made all or most of the decisions whereas 30.8% reported making decisions with parents.<br>Key Finding - A greater percentage of AYAC who reported making a joint decision with parents recalled 71% of fertility discussions than those who reported parents made most or all of medical decisions.	Medium

¥Age range at diagnosis is given except where this was not provided in the article in which alternative metrics are presented and this is highlighted.
AYA(C) = adolescent and young adult (with cancer); NOS = not otherwise specified; RM-ANOVA = repeated measures analysis of variance.

For peer review only - http://bmjopen.bmj.com/site/about/guidelines.xhtml

Update – December 2023

Medline

Ovid MEDLINE(R) and Epub Ahead of Print, In-Process, In-Data-Review & Other Non-Indexed Citations, Daily and Versions <1946 to December 19, 2023>

1 (Parent* or guardian* or mother* or father* or partner or wife or wives or husband* or boyfriend* or girlfriend* or sibling* or friend* or carer* or "third person" or caregiver* or "care-giver*" or spouse* or supporter* or support network*).ti,ab. or parents/ or fathers/ or mothers/ or spouses/ or caregivers/ or siblings/ or friends/ or legal guardians/ 1148168

2 (TYA cancer or TYA oncology or AYA cancer or AYA oncology or (young adult adj3 (cancer or oncology or leuk?em* or lymphom* or h?ematol*)) or ("teenage and young adult" adj3 cancer) or ("teenage and young adult" adj3 oncology) or (teenage* adj3 cancer) or (teenage* adj3 oncology) or (adolescen* adj3 cancer) or (adolescen* adj3 oncology) or (young people adj3 cancer) or (young people adj3 oncology) or ("teenage and young adult" adj3 leuk?emia*) or (teenage* adj3 leuk?emia*) or (adolescen* adj3 leuk?emia*) or (young people adj3 leuk?emia*) or (young adult adj3 leuk?emia*) or ("teenage and young adult" adj3 h?ematol*) or (teenage* adj3 h?ematol*) or (adolescen* adj3 h?ematol*) or (young people adj3 h?ematol*) or (young adult adj3 h?ematol*) or ("teenage and young adult" adj3 lymphom*) or (teenage* adj3 lymphom*) or (adolescen* adj3 lymphom*) or (young people adj3 lymphom*) or (young adult adj3 lymphom*)).ti,ab. or ((exp adolescent/ or exp young adult/) and exp neoplasms/) 337284

3 (Communicat* or Disclos* or inform* or Interact* or relationship* or Conversation* or Dialogue* or triad* or Interview* or consult* or decision making).ti,ab. or exp communication/ or exp disclosure/ or exp information dissemination/ or exp physician-patient relations/ 6147070

4 (affect* or effect* or influenc* or resultant or impact* or perception* or perspective* or encounter* or preference or opinion or involvement or occurance* or feel or "go through" or experienc*).ti,ab. 13278879

5 1 and 2 and 3 and 4 3519

6 limit 5 to english language 3399

7 (202211* or 202212* or 2023* or 2024*).dp. or (202211* or 202212* or 2023* or 2024*).ez. or (202211* or 202212* or 2023* or 2024*).ed. or (202211* or 202212* or 2023* or 2024*).ep. 1958643

8 6 and 7 163

Embase

Embase <1974 to 2023 December 19>

For peer review only - http://bmjopen.bmj.com/site/about/guidelines.xhtml

1 (TYA cancer or TYA oncology or AYA cancer or AYA oncology or (young adult adj3 (cancer or oncology or leuk?em* or lymphom* or h?ematol*)) or ("teenage and young adult" adj3 cancer) or ("teenage and young adult" adj3 oncology) or (teenage* adj3 cancer) or (teenage* adj3 oncology) or (adolescen* adj3 cancer) or (adolescen* adj3 oncology) or (young people adj3 cancer) or (young people adj3 oncology) or ("teenage and young adult" adj3 leuk?emia*) or (teenage* adj3 leuk?emia*) or (adolescen* adj3 leuk?emia*) or (young people adj3 leuk?emia*) or (young adult adj3 leuk?emia*) or ("teenage and young adult" adj3 h?ematol*) or (teenage* adj3 h?ematol*) or (adolescen* adj3 h?ematol*) or (young people adj3 h?ematol*) or (young adult adj3 h?ematol*) or ("teenage and young adult" adj3 lymphom*) or (teenage* adj3 lymphom*) or (adolescen* adj3 lymphom*) or (young people adj3 lymphom*) or (young adult adj3 lymphom*)).ti,ab. or ((exp *adolescent/ or exp *young adult/) and exp *neoplasm/) 10359

2 (Communicat* or Disclos* or inform* or Interact* or relationship* or Conversation* or Dialogue* or triad* or Interview* or consult* or decision making).ti,ab. or exp *interpersonal communication/ or exp *professional-patient relationship/ or exp *information dissemination/ or exp *conversation/ 7565603

3 (Parent* or guardian* or mother* or father* or partner or wife or wives or husband* or boyfriend* or girlfriend* or sibling* or friend* or carer* or "third person" or caregiver* or "care-giver*" or spouse* or supporter* or support network*).ti,ab. or *parent/ or *father/ or *mother/ or *spouse/ or *caregiver/ or *social worker/ or *sibling/ or *friend/ or *legal guardian/ 1440315

4 (affect* or effect* or influenc* or resultant or impact* or perception* or perspective* or encounter* or preference or opinion or involvement or occurance* or feel or "go through" or experienc*).ti,ab. 16593794

5 1 and 2 and 3 and 4 1032

6 limit 5 to english language 1010

7 (202211* or 202212* or 2023* or 2024*).dc. or (202211* or 202212* or 2023* or 2024*).dd. or (202211* or 202212* or 2023* or 2024*).dp.2647560

8 6 and 7 107

PsycINFO (via Ebsco)

#	Query	Limiters/Expanders	Last Run Via	Results
S11	S1 AND S2 AND S7 AND S8	Limiters - Publication Date: 20221101-20241231 Expanders - Apply equivalent subjects Search modes - Boolean/Phrase	Interface - EBSCOhost Research Databases Search Screen - Basic Search Database - APA PsycInfo	238
S10	S1 AND S2 AND S7 AND S8	Expanders - Apply equivalent subjects Narrow by	Interface - EBSCOhost Research	6,315

For peer review only

		Language: - english<br>Search modes - Boolean/Phrase	Databases Search Screen - Basic Search<br>Database - APA PsycInfo	
S9	S1 AND S2 AND S7 AND S8	Expanders - Apply equivalent subjects<br>Search modes - Boolean/Phrase	Interface - EBSCOhost Research Databases<br>Search Screen - Basic Search<br>Database - APA PsycInfo	6,549
S8	(affect* or effect* or influenc* or resultant or impact* or perception* or perspective* or encounter* or preference or opinion or involvement or occurance* or feel or "go through" or experienc*)	Expanders - Apply equivalent subjects<br>Search modes - Boolean/Phrase	Interface - EBSCOhost Research Databases<br>Search Screen - Basic Search<br>Database - APA PsycInfo	3,524,144
S7	(S5) or (S3)	Expanders - Apply equivalent subjects<br>Search modes - Boolean/Phrase	Interface - EBSCOhost Research Databases<br>Search Screen - Basic Search<br>Database - APA PsycInfo	62,557
S6	S4 AND S5	Expanders - Apply equivalent subjects<br>Search modes - Boolean/Phrase	Interface - EBSCOhost Research Databases<br>Search Screen - Basic Search<br>Database - APA PsycInfo	62,384

For peer review only

S5	((DE “neoplasms” OR DE "Benign Neoplasms" OR DE "Breast Neoplasms" OR DE "Endocrine Neoplasms" OR DE "Leukemias" OR DE "Melanoma" OR DE "Metastasis" OR DE "Nervous System Neoplasms" OR DE "Terminal Cancer"))	Expanders - Apply equivalent subjects Search modes - Boolean/Phrase	Interface - EBSCOhost Research Databases Search Screen - Basic Search Database - APA PsycInfo	62,384
S4	((DE “neoplasms” OR DE "Benign Neoplasms" OR DE "Breast Neoplasms" OR DE "Endocrine Neoplasms" OR DE "Leukemias" OR DE "Melanoma" OR DE "Metastasis" OR DE "Nervous System Neoplasms" OR DE "Terminal Cancer"))	Expanders - Apply equivalent subjects Search modes - Boolean/Phrase	Interface - EBSCOhost Research Databases Search Screen - Basic Search Database - APA PsycInfo	62,384
S3	("TYA cancer" or "TYA oncology" or "AYA cancer" or "AYA oncology" or ("young adult" n3 (cancer or oncology or leuk?em* or lymphom* or h?ematol*)) or ("teenage and young adult" n3 cancer) or ("teenage and young adult" n3 oncology) or (teenage* n3 cancer) or (teenage* n3 oncology) or (adolescen* n3 cancer) or (adolescen* n3 oncology) or ("young people" n3 cancer) or ("young people" n3 oncology) or ("teenage and young adult" n3 leuk?emia*) or (teenage* n3 leuk?emia*) or (adolescen* n3 leuk?emia*) or ("young people" n3 leuk?emia*) or ("young adult" n3 leuk?emia*) or ("teenage and young adult" n3 h?ematol*) or (teenage* n3 h?ematol*) or (adolescen* n3 h?ematol*) or ("young people" n3 h?ematol*) or ("young adult" n3 h?ematol*) or ("teenage and young adult" n3 lymphom*) or (teenage* n3 lymphom*) or (adolescen* n3 lymphom*) or ("young people" n3 lymphom*) or ("young adult" n3 lymphom*))	Expanders - Apply equivalent subjects Search modes - Boolean/Phrase	Interface - EBSCOhost Research Databases Search Screen - Basic Search Database - APA PsycInfo	2,072
S2	(Communicat* or Disclos* or inform* or Interact* or relationship* or Conversation* or Dialogue* or triad* or Interview* or consult* or "decision making") or DE “communication” OR DE “information dissemination” OR DE “conversation”	Expanders - Apply equivalent subjects Search modes - Boolean/Phrase	Interface - EBSCOhost Research Databases Search Screen - Basic Search Database - APA PsycInfo	2,545,968

For peer review only

S1	(Parent* or guardian* or mother* or father* or partner or wife or wives or husband* or boyfriend* or girlfriend* or sibling* or friend* or teacher* or social worker* or carer* or "third person" or caregiver* or "care-giver*" or spouse* or chaperone*) OR DE “parents” OR DE “mothers” OR DE “fathers” OR DE “spouses” OR DE “wives” OR DE “husbands” OR DE “siblings” OR DE “significant others” OR DE “social workers” OR DE “guardianship” OR DE “caregivers”	Expanders - Apply equivalent subjects Search modes - Boolean/Phrase	Interface - EBSCOhost Research Databases Search Screen - Basic Search Database - APA PsycInfo	937,154

CINAHL (via Ebsco)

Wednesday, December 20, 2023 4:07:56 PM

#	Query	Limiters/Expanders	Last Run Via	Results
S7	S1 AND S2 AND S3 AND S4	Limiters - Publication Date: 20221101-20241231 Expanders - Apply equivalent subjects Search modes - Boolean/Phrase	Interface - EBSCOhost Research Databases Search Screen - Advanced Search Database - CINAHL	99
S6	S1 AND S2 AND S3 AND S4	Expanders - Apply equivalent subjects Narrow by Language: - english Search modes - Boolean/Phrase	Interface - EBSCOhost Research Databases Search Screen - Advanced Search Database - CINAHL	2,152
S5	S1 AND S2 AND S3 AND S4	Expanders - Apply equivalent subjects Search modes - Boolean/Phrase	Interface - EBSCOhost Research Databases Search Screen - Advanced Search Database - CINAHL	2,189

For peer review only

S4	(affect* or effect* or influenc* or resultant or impact* or perception* or perspective* or encounter* or preference or opinion or involvement or occurance* or feel or "go through" or experienc*)	Expanders - Apply equivalent subjects Search modes - Boolean/Phrase	Interface - EBSCOhost Research Databases Search Screen - Advanced Search Database - CINAHL	3,149,468
S3	("TYA cancer" or "TYA oncology" or "AYA cancer" or "AYA oncology" or ("young adult" n3 (cancer or oncology or leuk?em* or lymphom* or h?ematol*)) or ("teenage and young adult" n3 cancer) or ("teenage and young adult" n3 oncology) or (teenage* n3 cancer) or (teenage* n3 oncology) or (adolescen* n3 cancer) or (adolescen* n3 oncology) or ("young people" n3 cancer) or ("young people" n3 oncology) or ("teenage and young adult" n3 leuk?emia*) or (teenage* n3 leuk?emia*) or (adolescen* n3 leuk?emia*) or ("young people" n3 leuk?emia*) or ("young adult" n3 leuk?emia*) or ("teenage and young adult" n3 h?ematol*) or (teenage* n3 h?ematol*) or (adolescen* n3 h?ematol*) or ("young people" n3 h?ematol*) or ("young adult" n3 h?ematol*) or ("teenage and young adult" n3 lymphom*) or (teenage* n3 lymphom*) or (adolescen* n3 lymphom*) or ("young people" n3 lymphom*) or ("young adult" n3 lymphom*)) OR ((MH "adolescence+" OR MH "young adult+") AND (MH "neoplasms+"))	Expanders - Apply equivalent subjects Search modes - Boolean/Phrase	Interface - EBSCOhost Research Databases Search Screen - Advanced Search Database - CINAHL	61,263
S2	(Communicat* or Disclos* or inform* or Interact* or relationship* or Conversation* or Dialogue* or triad* or Interview* or consult* or "decision making") or MH "communication+" OR MH "discussion" OR MH "conversation"	Expanders - Apply equivalent subjects Search modes - Boolean/Phrase	Interface - EBSCOhost Research Databases Search Screen - Advanced	2,080,061

For peer review only

	OR (MH "Professional-Patient Relations+")		Search Database - CINAHL	
S1	(Parent* or guardian* or mother* or father* or partner or wife or wives or husband* or boyfriend* or girlfriend* or sibling* or friend* or teacher* or social worker* or carer* or "third person" or caregiver* or "care-giver*" or spouse* or chaperone*) OR MH "parents" OR MH "mothers" OR MH "fathers" OR MH "spouses" OR MH "siblings" OR MH "teachers" OR MH "social workers" OR MH "caregivers"	Expanders - Apply equivalent subjects Search modes - Boolean/Phrase	Interface - EBSCOhost Research Databases Search Screen - Advanced Search Database - CINAHL	567,768

Web of Science Core Collection

Web of Science Search Strategy (v0.1)

Database: Web of Science Core Collection

Entitlements:

- WOS.IC: 1993 to 2023
- WOS.CCR: 1985 to 2023
- WOS.SCI: 1900 to 2023
- WOS.AHCI: 1975 to 2023
- WOS.BHCI: 2008 to 2023
- WOS.BSCI: 2008 to 2023
- WOS.ESCI: 2018 to 2023
- WOS.ISTP: 1990 to 2023
- WOS.SSCI: 1956 to 2023
- WOS.ISSHP: 1990 to 2023

Searches:

1: TS=(Parent* or guardian* or mother* or father* or partner or wife or wives or husband* or boyfriend* or girlfriend* or sibling* or friend* or teacher* or social worker* or carer* or "third person" or caregiver* or "care-giver*" or spouse* or chaperone*)

Date Run: Wed Dec 20 2023 16:03:59 GMT+0000 (Greenwich Mean Time)

Results: 2283955

2: TS=("TYA cancer" or "TYA oncology" or "AYA cancer" or "AYA oncology" or ("young adult" near/3 (cancer or oncology or leuk?em* or lymphom* or h?ematol*)) or ("teenage and young adult" near/3 cancer) or ("teenage and young adult" near/3 oncology) or (teenage*

For peer review only

near/3 cancer) or (teenage* near/3 oncology) or (adolescen* near/3 cancer) or (adolescen* near/3 oncology) or ("young people" near/3 cancer) or ("young people" near/3 oncology) or ("teenage and young adult" near/3 leuk?emia*) or (teenage* near/3 leuk?emia*) or (adolescen* near/3 leuk?emia*) or ("young people" near/3 leuk?emia*) or ("young adult" near/3 leuk?emia*) or ("teenage and young adult" near/3 h?ematol*) or (teenage* near/3 h?ematol*) or (adolescen* near/3 h?ematol*) or ("young people" near/3 h?ematol*) or ("young adult" near/3 h?ematol*) or ("teenage and young adult" near/3 lymphom*) or (teenage* near/3 lymphom*) or (adolescen* near/3 lymphom*) or ("young people" near/3 lymphom*) or ("young adult" near/3 lymphom*)) Date Run: Wed Dec 20 2023 16:04:06 GMT+0000 (Greenwich Mean Time) Results: 8540

3: TS=(Communicat* or Disclos* or inform* or Interact* or relationship* or Conversation* or Dialogue* or triad* or Interview* or consult* or "decision making")
Date Run: Wed Dec 20 2023 16:04:13 GMT+0000 (Greenwich Mean Time)
Results: 12748181

4: TS= (affect* or effect* or influenc* or resultant or impact* or perception* or perspective* or encounter* or preference or opinion or involvement or occurance* or feel or "go through" or experienc*) Date Run: Wed Dec 20 2023 16:04:19 GMT+0000 (Greenwich Mean Time) Results: 26006930

5: #4 AND #3 AND #2 AND #1 Date Run: Wed Dec 20 2023 16:04:26 GMT+0000 (Greenwich Mean Time) Results: 764

6: #4 AND #3 AND #2 AND #1 and English (Languages) Date Run: Wed Dec 20 2023 16:04:35 GMT+0000 (Greenwich Mean Time) Results: 737

7: #4 AND #3 AND #2 AND #1 and English (Languages) and 2022 or 2023 (Publication Years) Date Run: Wed Dec 20 2023 16:04:39 GMT+0000 (Greenwich Mean Time) Results: 132

AMED via Ovid

AMED (Allied and Complementary Medicine) <1985 to October 2023>

1 (Parent* or guardian* or mother* or father* or partner or wife or wives or husband* or boyfriend* or girlfriend* or sibling* or friend* or carer* or "third person" or caregiver* or "care-giver*" or spouse* or supporter* or support network*).ti,ab. 14291
2 (TYA cancer or TYA oncology or AYA cancer or AYA oncology or (young adult adj3 (cancer or oncology or leuk?em* or lymphom* or h?ematol*)) or ("teenage and young adult" adj3 cancer) or ("teenage and young adult" adj3 oncology) or (teenage* adj3 cancer) or (teenage* adj3 oncology) or (adolescen* adj3 cancer) or (adolescen* adj3 oncology) or (young people adj3 cancer) or (young people adj3 oncology) or ("teenage and young adult" adj3 leuk?emia*) or (teenage* adj3 leuk?emia*) or (adolescen* adj3 leuk?emia*) or (young people adj3 leuk?emia*) or (young adult adj3 leuk?emia*) or ("teenage and young adult" adj3 h?ematol*) or (teenage* adj3 h?ematol*) or (adolescen* adj3 h?ematol*) or (young people adj3 h?ematol*) or (young adult adj3 h?ematol*) or ("teenage and young adult" adj3

For peer review only

lymphom*) or (teenage* adj3 lymphom*) or (adolescen* adj3 lymphom*) or (young people adj3 lymphom*) or (young adult adj3 lymphom*)).ti,ab. 120
3 (Communicat* or Disclos* or inform* or Interact* or relationship* or Conversation* or Dialogue* or triad* or Interview* or consult* or decision making).ti,ab. 60609
4 (affect* or effect* or influenc* or resultant or impact* or perception* or perspective* or encounter* or preference or opinion or involvement or occurance* or feel or "go through" or experienc*).ti,ab. 143225
5 1 and 2 and 3 and 4 19
6 limit 5 to yr="2005 -Current" 14

For peer review only

First run – November 2022

Medline (via Ovid)

Ovid MEDLINE(R) and Epub Ahead of Print, In-Process, In-Data-Review & Other Non-Indexed Citations, Daily and Versions <1946 to November 23, 2022>

1 (Parent* or guardian* or mother* or father* or partner or wife or wives or husband* or boyfriend* or girlfriend* or sibling* or friend* or carer* or "third person" or caregiver* or "care-giver*" or spouse* or supporter* or support network*).ti,ab. or parents/ or fathers/ or mothers/ or spouses/ or caregivers/ or siblings/ or friends/ or legal guardians/ 1074121

2 (TYA cancer or TYA oncology or AYA cancer or AYA oncology or (young adult adj3 (cancer or oncology or leuk?em* or lymphom* or h?ematol*)) or ("teenage and young adult" adj3 cancer) or ("teenage and young adult" adj3 oncology) or (teenage* adj3 cancer) or (teenage* adj3 oncology) or (adolescen* adj3 cancer) or (adolescen* adj3 oncology) or (young people adj3 cancer) or (young people adj3 oncology) or ("teenage and young adult" adj3 leuk?emia*) or (teenage* adj3 leuk?emia*) or (adolescen* adj3 leuk?emia*) or (young people adj3 leuk?emia*) or (young adult adj3 leuk?emia*) or ("teenage and young adult" adj3 h?ematol*) or (teenage* adj3 h?ematol*) or (adolescen* adj3 h?ematol*) or (young people adj3 h?ematol*) or (young adult adj3 h?ematol*) or ("teenage and young adult" adj3 lymphom*) or (teenage* adj3 lymphom*) or (adolescen* adj3 lymphom*) or (young people adj3 lymphom*) or (young adult adj3 lymphom*)).ti,ab. or ((exp adolescent/ or exp young adult/) and exp neoplasms/) 333070

3 (Communicat* or Disclos* or inform* or Interact* or relationship* or Conversation* or Dialogue* or triad* or Interview* or consult* or decision making).ti,ab. or exp communication/ or exp disclosure/ or exp information dissemination/ or exp physician-patient relations/ 5715959

4 (affect* or effect* or influenc* or resultant or impact* or perception* or perspective* or encounter* or preference or opinion or involvement or occurance* or feel or "go through" or experienc*).ti,ab. 12406352

5 1 and 2 and 3 and 4 3380

6 limit 5 to (english language and yr="2005 -Current") 2715

Embase (via Ovid)

Embase <1974 to 2022 November 23>

For peer review only

1 (TYA cancer or TYA oncology or AYA cancer or AYA oncology or (young adult adj3 (cancer or oncology or leuk?em* or lymphom* or h?ematol*)) or ("teenage and young adult" adj3 cancer) or ("teenage and young adult" adj3 oncology) or (teenage* adj3 cancer) or (teenage* adj3 oncology) or (adolescen* adj3 cancer) or (adolescen* adj3 oncology) or (young people adj3 cancer) or (young people adj3 oncology) or ("teenage and young adult" adj3 leuk?emia*) or (teenage* adj3 leuk?emia*) or (adolescen* adj3 leuk?emia*) or (young people adj3 leuk?emia*) or (young adult adj3 leuk?emia*) or ("teenage and young adult" adj3 h?ematol*) or (teenage* adj3 h?ematol*) or (adolescen* adj3 h?ematol*) or (young people adj3 h?ematol*) or (young adult adj3 h?ematol*) or ("teenage and young adult" adj3 lymphom*) or (teenage* adj3 lymphom*) or (adolescen* adj3 lymphom*) or (young people adj3 lymphom*) or (young adult adj3 lymphom*)).ti,ab. or ((exp *adolescent/ or exp *young adult/) and exp *neoplasm/) 9638

2 (Communicat* or Disclos* or inform* or Interact* or relationship* or Conversation* or Dialogue* or triad* or Interview* or consult* or decision making).ti,ab. or exp *interpersonal communication/ or exp *professional-patient relationship/ or exp *information dissemination/ or exp *conversation/ 6997005

3 (Parent* or guardian* or mother* or father* or partner or wife or wives or husband* or boyfriend* or girlfriend* or sibling* or friend* or carer* or "third person" or caregiver* or "care-giver*" or spouse* or supporter* or support network*).ti,ab. or *parent/ or *father/ or *mother/ or *spouse/ or *caregiver/ or *social worker/ or *sibling/ or *friend/ or *legal guardian/ 1339977

4 (affect* or effect* or influenc* or resultant or impact* or perception* or perspective* or encounter* or preference or opinion or involvement or occurance* or feel or "go through" or experienc*).ti,ab. 15453173

5 1 and 2 and 3 and 4 939

6 limit 5 to (english language and yr="2005 -Current") 873

PsycInfo (via Ebscohost)

#	Query	Limiters/Expanders	Last Run Via	Results
S11	S1 AND S2 AND S7 AND S8	Limiters - Publication Year: 2005-2022<br>Expanders - Apply equivalent subjects<br>Narrow by Language: - english<br>Search modes - Boolean/Phrase	Interface - EBSCOhost Research Databases<br>Search Screen - Basic Search<br>Database - APA PsycInfo	1,683
S10	S1 AND S2 AND S7 AND S8	Expanders - Apply equivalent subjects<br>Narrow by Language: - english	Interface - EBSCOhost Research Databases<br>Search Screen - Basic Search	1,981

For peer review only

		Search modes - Boolean/Phrase	Database - APA PsycInfo	
S9	S1 AND S2 AND S7 AND S8	Expanders - Apply equivalent subjects Search modes - Boolean/Phrase	Interface - EBSCOhost Research Databases Search Screen - Basic Search Database - APA PsycInfo	2,017
S8	(affect* or effect* or influenc* or resultant or impact* or perception* or perspective* or encounter* or preference or opinion or involvement or occurance* or feel or "go through" or experienc*)	Expanders - Apply equivalent subjects Search modes - Boolean/Phrase	Interface - EBSCOhost Research Databases Search Screen - Basic Search Database - APA PsycInfo	3,366,619
S7	(S5) or (S3)	Expanders - Apply equivalent subjects Search modes - Boolean/Phrase	Interface - EBSCOhost Research Databases Search Screen - Basic Search Database - APA PsycInfo	13,719
S6	S4 AND S5	Expanders - Apply equivalent subjects Search modes - Boolean/Phrase	Interface - EBSCOhost Research Databases Search Screen - Basic Search Database - APA PsycInfo	13,275
S5	((DE “neoplasms” OR DE "Benign Neoplasms" OR DE "Breast Neoplasms" OR DE "Endocrine Neoplasms" OR DE "Leukemias" OR DE "Melanoma" OR DE "Metastasis" OR DE "Nervous System Neoplasms" OR DE "Terminal Cancer"))	Expanders - Apply equivalent subjects Narrow by SubjectAge: - adolescence (13-17 yrs) Narrow by SubjectAge: - young adulthood (18-29 yrs) Search modes - Boolean/Phrase	Interface - EBSCOhost Research Databases Search Screen - Basic Search Database - APA PsycInfo	13,275
S4	((DE “neoplasms” OR DE "Benign Neoplasms" OR DE "Breast Neoplasms" OR DE "Endocrine Neoplasms" OR DE "Leukemias" OR DE "Melanoma" OR DE "Metastasis" OR DE "Nervous System Neoplasms" OR DE "Terminal Cancer"))	Expanders - Apply equivalent subjects Search modes - Boolean/Phrase	Interface - EBSCOhost Research Databases Search Screen - Basic Search Database - APA PsycInfo	58,767
S3	("TYA cancer" or "TYA oncology" or "AYA cancer" or "AYA oncology" or ("young adult" n3 (cancer or oncology or leuk?em* or lymphom* or h?ematol*)) or ("teenage and young adult" n3 cancer) or ("teenage and young adult" n3 oncology) or (teenage* n3 cancer) or (teenage* n3 oncology) or (adolescen* n3 cancer) or	Expanders - Apply equivalent subjects Search modes - Boolean/Phrase	Interface - EBSCOhost Research Databases Search Screen - Basic Search Database - APA PsycInfo	1,864

For peer review only

	(adolescen* n3 oncology) or ("young people" n3 cancer) or ("young people" n3 oncology) or ("teenage and young adult" n3 leuk?emia*) or (teenage* n3 leuk?emia*) or (adolescen* n3 leuk?emia*) or ("young people" n3 leuk?emia*) or ("young adult" n3 leuk?emia*) or ("teenage and young adult" n3 h?ematol*) or (teenage* n3 h?ematol*) or (adolescen* n3 h?ematol*) or ("young people" n3 h?ematol*) or ("young adult" n3 h?ematol*) or ("teenage and young adult" n3 lymphom*) or (teenage* n3 lymphom*) or (adolescen* n3 lymphom*) or ("young people" n3 lymphom*) or ("young adult" n3 lymphom*))			
S2	(Communicat* or Disclos* or inform* or Interact* or relationship* or Conversation* or Dialogue* or triad* or Interview* or consult* or "decision making") or DE “communication” OR DE “information dissemination” OR DE “conversation”	Expanders - Apply equivalent subjects<br>Search modes - Boolean/Phrase	Interface - EBSCOhost Research Databases<br>Search Screen - Basic Search<br>Database - APA PsycInfo	2,423,980
S1	(Parent* or guardian* or mother* or father* or partner or wife or wives or husband* or boyfriend* or girlfriend* or sibling* or friend* or teacher* or social worker* or carer* or "third person" or caregiver* or "care-giver*" or spouse* or chaperone*) OR DE “parents” OR DE “mothers” OR DE “fathers” OR DE “spouses” OR DE “wives” OR DE “husbands” OR DE “siblings” OR DE “significant others” OR DE “social workers” OR DE “guardianship” OR DE “caregivers”	Expanders - Apply equivalent subjects<br>Search modes - Boolean/Phrase	Interface - EBSCOhost Research Databases<br>Search Screen - Basic Search<br>Database - APA PsycInfo	894,375

CINAHL (via Ebscohost)

Accessibility Information and Tips

Print Search History

Thursday, November 24, 2022 6:21:27 PM

#	Query	Limiters/Expanders	Last Run Via	Results
S7	S1 AND S2 AND S3 AND S4	Limiters - Published Date: 20050101-20221231<br>Expanders - Apply equivalent subjects<br>Narrow by Language: - english<br>Search modes - Boolean/Phrase	Interface - EBSCOhost Research Databases<br>Search Screen - Advanced Search<br>Database - CINAHL	1,837

For peer review only

S6	S1 AND S2 AND S3 AND S4	Limiters - Published Date: 20050101-20221231 Expanders - Apply equivalent subjects Search modes - Boolean/Phrase	Interface - EBSCOhost Research Databases Search Screen - Advanced Search Database - CINAHL	1,866
S5	S1 AND S2 AND S3 AND S4	Expanders - Apply equivalent subjects Search modes - Boolean/Phrase	Interface - EBSCOhost Research Databases Search Screen - Advanced Search Database - CINAHL	2,106
S4	(affect* or effect* or influenc* or resultant or impact* or perception* or perspective* or encounter* or preference or opinion or involvement or occurance* or feel or "go through" or experienc*)	Expanders - Apply equivalent subjects Search modes - Boolean/Phrase	Interface - EBSCOhost Research Databases Search Screen - Advanced Search Database - CINAHL	3,016,184
S3	("TYA cancer" or "TYA oncology" or "AYA cancer" or "AYA oncology" or ("young adult" n3 (cancer or oncology or leuk?em* or lymphom* or h?ematol*)) or ("teenage and young adult" n3 cancer) or ("teenage and young adult" n3 oncology) or (teenage* n3 cancer) or (teenage* n3 oncology) or (adolescen* n3 cancer) or (adolescen* n3 oncology) or ("young people" n3 cancer) or ("young people" n3 oncology) or ("teenage and young adult" n3 leuk?emia*) or (teenage* n3 leuk?emia*) or (adolescen* n3 leuk?emia*) or ("young people" n3	Expanders - Apply equivalent subjects Search modes - Boolean/Phrase	Interface - EBSCOhost Research Databases Search Screen - Advanced Search Database - CINAHL	59,927

For peer review only

	leuk?emia*) or ("young adult" n3 leuk?emia*) or ("teenage and young adult" n3 h?ematol*) or (teenage* n3 h?ematol*) or (adolescen* n3 h?ematol*) or ("young people" n3 h?ematol*) or ("young adult" n3 h?ematol*) or ("teenage and young adult" n3 lymphom*) or (teenage* n3 lymphom*) or (adolescen* n3 lymphom*) or ("young people" n3 lymphom*) or ("young adult" n3 lymphom*)) OR ((MH "adolescence+" OR MH "young adult+") AND (MH "neoplasms+"))			
S2	(Communicat* or Disclos* or inform* or Interact* or relationship* or Conversation* or Dialogue* or triad* or Interview* or consult* or "decision making") or MH "communication+" OR MH "discussion" OR MH "conversation" OR (MH "Professional-Patient Relations+")	Expanders - Apply equivalent subjects Search modes - Boolean/Phrase	Interface - EBSCOhost Research Databases Search Screen - Advanced Search Database - CINAHL	2,016,086
S1	(Parent* or guardian* or mother* or father* or partner or wife or wives or husband* or boyfriend* or girlfriend* or sibling* or friend* or teacher* or social worker* or carer* or "third person" or caregiver* or "care-giver*" or spouse* or chaperone*) OR MH "parents" OR MH "mothers" OR MH "fathers" OR MH "spouses" OR MH "siblings" OR MH "teachers" OR MH "social workers" OR MH "caregivers"	Expanders - Apply equivalent subjects Search modes - Boolean/Phrase	Interface - EBSCOhost Research Databases Search Screen - Advanced Search Database - CINAHL	544,991

Web of Science Core Collection

Web of Science Search Strategy (v0.1)

Database: Web of Science Core Collection

Entitlements:

- WOS.IC: 1993 to 2022
- WOS.CCR: 1985 to 2022

For peer review only

- WOS.SCI: 1900 to 2022
- WOS.AHCI: 1975 to 2022
- WOS.BHCI: 2008 to 2022
- WOS.BSCI: 2008 to 2022
- WOS.ESCI: 2017 to 2022
- WOS.ISTP: 1990 to 2022
- WOS.SSCI: 1956 to 2022
- WOS.ISSHP: 1990 to 2022

Searches:

1: TS=(Parent* or guardian* or mother* or father* or partner or wife or wives or husband* or boyfriend* or girlfriend* or sibling* or friend* or teacher* or social worker* or carer* or "third person" or caregiver* or "care-giver*" or spouse* or chaperone*)
Results: 2129759

2: TS=("TYA cancer" or "TYA oncology" or "AYA cancer" or "AYA oncology" or ("young adult" near/3 (cancer or oncology or leuk?em* or lymphom* or h?ematol*)) or ("teenage and young adult" near/3 cancer) or ("teenage and young adult" near/3 oncology) or (teenage* near/3 cancer) or (teenage* near/3 oncology) or (adolescen* near/3 cancer) or (adolescen* near/3 oncology) or ("young people" near/3 cancer) or ("young people" near/3 oncology) or ("teenage and young adult" near/3 leuk?emia*) or (teenage* near/3 leuk?emia*) or (adolescen* near/3 leuk?emia*) or ("young people" near/3 leuk?emia*) or ("young adult" near/3 leuk?emia*) or ("teenage and young adult" near/3 h?ematol*) or (teenage* near/3 h?ematol*) or (adolescen* near/3 h?ematol*) or ("young people" near/3 h?ematol*) or ("young adult" near/3 h?ematol*) or ("teenage and young adult" near/3 lymphom*) or (teenage* near/3 lymphom*) or (adolescen* near/3 lymphom*) or ("young people" near/3 lymphom*) or ("young adult" near/3 lymphom*)) Results: 7793

3: TS=(Communicat* or Disclos* or inform* or Interact* or relationship* or Conversation* or Dialogue* or triad* or Interview* or consult* or "decision making")
Results: 11889093

4: TS= (affect* or effect* or influenc* or resultant or impact* or perception* or perspective* or encounter* or preference or opinion or involvement or occurance* or feel or "go through" or experienc*) Results: 24306121

5: #4 AND #3 AND #2 AND #1 Results: 684

6: #4 AND #3 AND #2 AND #1 Results: 684

7: #4 AND #3 AND #2 AND #1 and 2005 or 2006 or 2007 or 2008 or 2009 or 2010 or 2011 or 2012 or 2013 or 2014 or 2015 or 2016 or 2017 or 2018 or 2019 or 2020 or 2021 or 2022 (Publication Years) Results: 644

For peer review only

8: #4 AND #3 AND #2 AND #1 and 2005 or 2006 or 2007 or 2008 or 2009 or 2010 or 2011 or 2012 or 2013 or 2014 or 2015 or 2016 or 2017 or 2018 or 2019 or 2020 or 2021 or 2022 (Publication Years) and English (Languages) Results: 619

For peer review only

PRISMA 2020 Checklist

Section and Topic	Item #	Checklist item	Location where item is reported
**TITLE**			
Title	1	Identify the report as a systematic review.	Page 1
**ABSTRACT**			
Abstract	2	See the PRISMA 2020 for Abstracts checklist.	Page 2
**INTRODUCTION**			
Rationale	3	Describe the rationale for the review in the context of existing knowledge.	Pages 3 and 4
Objectives	4	Provide an explicit statement of the objective(s) or question(s) the review addresses.	Page 4
**METHODS**			
Eligibility criteria	5	Specify the inclusion and exclusion criteria for the review and how studies were grouped for the syntheses.	Page 5
Information sources	6	Specify all databases, registers, websites, organisations, reference lists and other sources searched or consulted to identify studies. Specify the date when each source was last searched or consulted.	Page 4 and supplemental file
Search strategy	7	Present the full search strategies for all databases, registers and websites, including any filters and limits used.	Page 4, Table 1 and supplemental file 1
Selection process	8	Specify the methods used to decide whether a study met the inclusion criteria of the review, including how many reviewers screened each record and each report retrieved, whether they worked independently, and if applicable, details of automation tools used in the process.	Page 5
Data collection process	9	Specify the methods used to collect data from reports, including how many reviewers collected data from each report, whether they worked independently, any processes for obtaining or confirming data from study investigators, and if applicable, details of automation tools used in the process.	Pages 4 and 5
Data items	10a	List and define all outcomes for which data were sought. Specify whether all results that were compatible with each outcome domain in each study were sought (e.g. for all measures, time points, analyses), and if not, the methods used to decide which results to collect.	Page 5
	10b	List and define all other variables for which data were sought (e.g. participant and intervention characteristics, funding sources). Describe any assumptions made about any missing or unclear information.	Page 5
Study risk of bias assessment	11	Specify the methods used to assess risk of bias in the included studies, including details of the tool(s) used, how many reviewers assessed each study and whether they worked independently, and if applicable, details of automation tools used in the process.	Page 5
Effect measures	12	Specify for each outcome the effect measure(s) (e.g. risk ratio, mean difference) used in the synthesis or presentation of results.	Page 5
Synthesis methods	13a	Describe the processes used to decide which studies were eligible for each synthesis (e.g. tabulating the study intervention characteristics and comparing against the planned groups for each synthesis (item #5)).	Page 5
	13b	Describe any methods required to prepare the data for presentation or synthesis, such as handling of missing summary statistics, or data conversions.	Page 5
	13c	Describe any methods used to tabulate or visually display results of individual studies and syntheses.	Page 5
	13d	Describe any methods used to synthesize results and provide a rationale for the choice(s). If meta-analysis was performed, describe the model(s), method(s) to identify the presence and extent of statistical heterogeneity, and software package(s) used.	Page 5

For peer review only - http://bmjopen.bmj.com/site/about/guidelines.xhtml

PRISMA 2020 Checklist

Section and Topic	Item #	Checklist item	Location where item is reported
	13e	Describe any methods used to explore possible causes of heterogeneity among study results (e.g. subgroup analysis, meta-regression).	Pages 5 and 6
	13f	Describe any sensitivity analyses conducted to assess robustness of the synthesized results.	Not applicable
Reporting bias assessment	14	Describe any methods used to assess risk of bias due to missing results in a synthesis (arising from reporting biases).	Not applicable
Certainty assessment	15	Describe any methods used to assess certainty (or confidence) in the body of evidence for an outcome.	Not applicable
**RESULTS**			
Study selection	16a	Describe the results of the search and selection process, from the number of records identified in the search to the number of studies included in the review, ideally using a flow diagram.	Figure 1 page 6
	16b	Cite studies that might appear to meet the inclusion criteria, but which were excluded, and explain why they were excluded.	Figure 1 page 6
Study characteristics	17	Cite each included study and present its characteristics.	Table 2 – summary of articles pages
Risk of bias in studies	18	Present assessments of risk of bias for each included study.	Not reported
Results of individual studies	19	For all outcomes, present, for each study: (a) summary statistics for each group (where appropriate) and (b) an effect estimate and its precision (e.g. confidence/credible interval), ideally using structured tables or plots.	Not applicable
Results of syntheses	20a	For each synthesis, briefly summarise the characteristics and risk of bias among contributing studies.	Not applicable
	20b	Present results of all statistical syntheses conducted. If meta-analysis was done, present for each the summary estimate and its precision (e.g. confidence/credible interval) and measures of statistical heterogeneity. If comparing groups, describe the direction of the effect.	Not applicable
	20c	Present results of all investigations of possible causes of heterogeneity among study results.	Not applicable
	20d	Present results of all sensitivity analyses conducted to assess the robustness of the synthesized results.	Not applicable
Reporting biases	21	Present assessments of risk of bias due to missing results (arising from reporting biases) for each synthesis assessed.	Not applicable
Certainty of evidence	22	Present assessments of certainty (or confidence) in the body of evidence for each outcome assessed.	Not applicable
**DISCUSSION**			
Discussion	23a	Provide a general interpretation of the results in the context of other evidence.	Pages 10 and 11
	23b	Discuss any limitations of the evidence included in the review.	Pages 11 and 12

For peer review only

PRISMA 2020 Checklist

Section and Topic	Item #	Checklist item	Location where item is reported
	23c	Discuss any limitations of the review processes used.	Pages 11 and 12
	23d	Discuss implications of the results for practice, policy, and future research.	Page 12
**OTHER INFORMATION**			
Registration and protocol	24a	Provide registration information for the review, including register name and registration number, or state that the review was not registered.	Page 3
	24b	Indicate where the review protocol can be accessed, or state that a protocol was not prepared.	Supplemental file
	24c	Describe and explain any amendments to information provided at registration or in the protocol.	Not appliable
Support	25	Describe sources of financial or non-financial support for the review, and the role of the funders or sponsors in the review.	Page 13
Competing interests	26	Declare any competing interests of review authors.	Page 13
Availability of data, code and other materials	27	Report which of the following are publicly available and where they can be found: template data collection forms; data extracted from included studies; data used for all analyses; analytic code; any other materials used in the review.	Page 13

From: Page MJ, McKenzie JE, Bossuyt PM, Boutron I, Hoffmann TC, Mulrow CD, et al. The PRISMA 2020 statement: an updated guideline for reporting systematic reviews. BMJ 2021;372:n71. doi: 10.1136/bmj.n71

For more information, visit: http://www.prisma-statement.org/

For peer review only

BMJ Open

Triadic communication with teenagers and young adults with cancer: a systematic literature review: “Make me feel like I’m not the third person”

Journal:	*BMJ Open*
Manuscript ID	bmjopen-2023-080024.R2
Article Type:	Original research
Date Submitted by the Author:	30-Jan-2024
Complete List of Authors:	Critoph, Deborah; University of Cambridge, Department of Public Health & Primary Care<br>Taylor, Rachel; University College London Hospitals NHS Foundation Trust, CNMAR;<br>Spathis, Anna; University of Cambridge<br>Duschinsky, Robbie; University of Cambridge<br>Hatcher, Helen; Cambridge University Hospitals NHS Foundation Trust<br>Clyne, Ella; University of Cambridge<br>Kuhn, Isla; University of Cambridge, Medical Library, School of Clinical Medicine<br>Smith, Luke; Cambridge University Hospitals NHS Foundation Trust
**Primary Subject Heading**:	Communication
Secondary Subject Heading:	Communication, Oncology, Patient-centred medicine, Paediatrics, Medical education and training
Keywords:	Adolescents < Adolescent, Decision Making, EDUCATION & TRAINING (see Medical Education & Training), ONCOLOGY

SCHOLARONE™
Manuscripts

I, the Submitting Author has the right to grant and does grant on behalf of all authors of the Work (as defined in the below author licence), an exclusive licence and/or a non-exclusive licence for contributions from authors who are: i) UK Crown employees; ii) where BMJ has agreed a CC-BY licence shall apply, and/or iii) in accordance with the terms applicable for US Federal Government officers or employees acting as part of their official duties; on a worldwide, perpetual, irrevocable, royalty-free basis to BMJ Publishing Group Ltd ("BMJ") its licensees and where the relevant Journal is co-owned by BMJ to the co-owners of the Journal, to publish the Work in this journal and any other BMJ products and to exploit all rights, as set out in our licence.

The Submitting Author accepts and understands that any supply made under these terms is made by BMJ to the Submitting Author unless you are acting as an employee on behalf of your employer or a postgraduate student of an affiliated institution which is paying any applicable article publishing charge ("APC") for Open Access articles. Where the Submitting Author wishes to make the Work available on an Open Access basis (and intends to pay the relevant APC), the terms of reuse of such Open Access shall be governed by a Creative Commons licence – details of these licences and which Creative Commons licence will apply to this Work are set out in our licence referred to above.

Other than as permitted in any relevant BMJ Author's Self Archiving Policies, I confirm this Work has not been accepted for publication elsewhere, is not being considered for publication elsewhere and does not duplicate material already published. I confirm all authors consent to publication of this Work and authorise the granting of this licence.

For peer review only

Triadic communication with teenagers and young adults with cancer: a systematic literature review: "Make me feel like I'm not the third person"

Deborah J Critoph MSc, Rachel M Taylor PhD, Anna Spathis MD, Robbie Duschinsky PhD, Helen Hatcher MD PhD, Ella Clyne, Isla Kuhn MSc, Luke AM Smith

Deborah Critoph, PhD student, Department of Public Health & Primary Care, University of Cambridge

Rachel Taylor, Director of the CNMAR, Centre for Nurse, Midwife and Allied Health Profession Led Research, University College London Hospitals NHS Foundation Trust, London, NW1 2PG

Anna Spathis, Assistant Professor, Department of Public Health and Primary Care, University of Cambridge

Robbie Duschinsky, Professor of Social Science & Health, Department of Public Health & Primary Care, University of Cambridge

Helen Hatcher, Consultant in Medical and TYA Oncology, Clinical and Regional Lead for Sarcoma and Young People with Cancer, Clinical Director EoE TYAc ODN

Ella Clyne, Medical Student, University of Cambridge

Isla Kuhn, Head of Medical Library Services, University of Cambridge Medical Library

Luke Smith, Junior Clinical Fellow in TYA Haematology and Oncology, Cambridge University Hospitals NHS Foundation Trust

Corresponding author: Deborah Critoph, dc625@cam.ac.uk

Keywords: Teenagers and young adults with cancer (TYAC), adolescents, triadic communication, communication, supporters, third person, parents, support network, health care, professionals, experiences, impact.

Abstract = 290/300
Word count = 4,468
Number of tables = 4
Number of figures = 1

For peer review only

Abstract

Objectives

Clinical communication needs of teenagers and young adults with cancer (TYAC) are increasingly recognised to differ significantly from younger children and older adults. We sought to understand who is present with TYACs, TYACs experiences of triadic communication and its impact. We generated three research questions to focus this review:

1. Who is present with TYACs in healthcare consultations/communication?
2. What are TYACs' experiences of communication with the supporter present?
3. What is the impact of a TYAC's supporter being present in the communication?

Design

Systematic review with narrative synthesis.

Data sources

The search was conducted across six databases: Medline, CINAHL, Embase, PsycINFO, Web of Science and AMED for all publications up to December 2023.

Eligibility criteria for selecting studies

Included papers were empirical research published after 2005; participants had malignant disease, diagnosed aged 13-24 years (for over 50% of participants); the research addressed any area of clinical communication.

Data extraction and synthesis

Three independent reviewers undertook full text screening. A review-specific data extraction form was used to record participant characteristics and methods from each included paper and results relevant to the three review questions.

Results

A total of 8,480 studies were identified in the search, of which 36 fulfilled the inclusion criteria. We found that mothers were the most common supporter present in clinical communication encounters. TYACs experiences of triadic communication are paradoxical in nature – the supporter can help or hinder the involvement of the young person in care related communication. Overall, young people are not included in clinical communication and decisions at their preferred level.

Conclusion

Triadic communication in TYAC care is common, complex, and dynamic. Due to the degree of challenge and nuances raised, HCPs need further training on effective triadic communication.

PROSPERO registration number CRD42022374528

For peer review only

Strengths & limitations of this study

- We searched systematically and thoroughly for eligible studies, but this is not a well-indexed field of research, and therefore it is possible that some relevant studies were not included in the review.
- We limited the review to a UK TYAC age range and not the broader age used elsewhere, so the conclusions are applicable to younger adults, up to aged 24 only and not necessarily the age of young adulthood used in some countries (between 29 to 39).
- We only included papers published in English and the results may not be applicable to other countries especially where cultural differences affect parental-TYAC or other familial/romantic relational dynamics.
- International representation was seen in the eligible studies and TYAC ages were included across the entirety of the specified UK age range.
- Studies represented the journey throughout the cancer experience from diagnosis to survivorship and end of life care.

Introduction

Adolescence is a time of transition where young people navigate monumental physical, cognitive, emotional, and behavioural milestones to develop a sense of self-identity and gain independence. Although most young people have limited encounters with healthcare, around 2,500 young people in the United Kingdom (UK) are diagnosed with cancer each year, which is the leading cause of non-traumatic death in young people in the United States (US) and Europe.(1) Teenagers and young adults with cancer (TYACs) have unique healthcare needs and there has been an international drive to develop developmentally appropriate evidence-based specialist care, provided by appropriately trained healthcare professionals (HCPs).(2)

Communication with TYACs can be particularly challenging: a life-limiting condition intersects an age associated with emotional reactivity and variable maturity. TYACs clinical communication needs are increasingly recognised to differ significantly from younger children and older adults. Research indicates TYACs can have little meaningful involvement in conversations with HCPs: almost half of children and young people reported not being involved in decisions about their care.(3) HCPs recognise this and consider young people amongst the hardest patients to communicate with.(4) However, HCPs receive little training about how best to manage these clinical encounters. TYACs perceive that HCPs do not make efforts to understand how their cancer impacts their life outside of the healthcare setting. As a result, they may withdraw and subsequently be labelled as 'challenging', 'hard to reach' and 'disengaged'. This may adversely impact care and contribute to poor physical and psychological outcomes. Despite these issues, there are limited opportunities for formal postgraduate education in communication with TYACs for HCPs, with most training being ad hoc and not interprofessional.(5,6) Effective communication with TYACs has been recognised as a key national research priority. In a UK-wide survey of young patients' own research priorities, communication was a striking cross-cutting theme.(7)

Recent research into clinical communication with TYACs has offered some insight into the complexities of communication with this specialist patient group.(8–12) Yet one area that has

For peer review only - http://bmjopen.bmj.com/site/about/guidelines.xhtml

received less attention is triadic communication. Triadic communication refers to the presence of a third party, such as a parent, carer, or companion in clinical encounters (13) and the presence of such a person was found to occur in 87% of TYAC consultations.(11) As a commonly occurring form of communication in TYAC care, there is a need to understand the theoretical basis and relevance of triadic communication to clinical practice. For the purposes of this review, we refer to this third person as a supporter. Triadic communication literature from children and older adults exists. (14–17) Notably this includes a meta-analytic review of provider-patient-companion of adults,(18) one large systematic review of physician-patient-companion communication and decision-making in adults (19) and one review of doctor-parent-child communication.(20) Whilst informative, these studies are with children and adults, not this unique age-group of emerging adulthood with a significant life threatening diagnosis such as cancer. Also, these studies focus on doctor-patient-third person communication, whereas TYAC care involves a range of interdisciplinary professionals. This review aims to understand what is known about triadic communication with TYACs in healthcare communication.

Aim

We sought to understand who is present with TYACs, synthesise TYACs experiences of triadic communication with HCPs and supporter(s), and develop insights into the impact of triadic communication for TYACs.

Review questions:

1. Who is the supporter present with TYACs in healthcare consultations and communication?
2. What are TYACs' experiences of communication with the supporter present?
3. What is the impact on a TYAC's supporter being present in the communication?

Methods

We conducted a systematic review and narrative synthesis (21,22) of empirical evidence published since 2005, the year of publication of the National Institute for Care Excellence (NICE) Improving Outcomes Guidance, the guidance document underpinning TYAC services in England.(2) The review protocol was prospectively registered with PROSPERO (CRD42022374528). We designed the search to identify and map the available evidence using a broad scope to gain an overview of the pertinent literature, identify knowledge gaps and clarify concepts. The search strategy was developed and refined with an information scientist (I.K.). Keywords were generated across five strands detailed in Table 1, with strands combined with the Boolean operator 'AND'. The search was conducted across six databases: Medline, CINAHL, Embase, PsycINFO, Web of Science and AMED (supplementary file 1).

For peer review only

Table 1. Search Terms

<u>Strand 1 – TYAC</u>
TYA cancer or TYA oncology or teenage and young adult adj5 cancer or teenage and young adult adj5 oncology or teenage* adj5 cancer or teenage* adj5 oncology or adolescen* adj 5 cancer or adolescen* adj 5 oncology or young people adj 5 cancer or young people adj 5 oncology
<u>Strand 2 – communication</u>
Communication skills OR communicat* OR discuss* OR disclos* OR inform* OR interact OR relationship building OR decision making OR communication tools OR communication aids OR psychosocial assessment
<u>Strand 3 – supporters</u>
Parent* or guardian* or mother* or father* or partner or wife or wives or husband* or boyfriend* or girlfriend* or sibling* or friend* or carer* or "third person" or caregiver* or "care-giver*" or spouse* or supporter* or support network*.
<u>Strand 4 - impact</u>
affect OR effect OR influence OR result OR resultant OR impact
<u>Strand 5 - experience</u>
encounter OR involvement OR occurrence OR feel OR "go through" OR experience*

TYAC: teenage and young adult with cancer

Database searches were compiled and de-duplicated in Mendeley, abstracts were screened in Rayyan by two researchers (D.J.C and L.A.M.S.), and 172 full articles were read by three researchers (L.A.M.S., D.J.C., and R.M.T) for eligibility of inclusion in the final analysis, with disagreements resolved by discussion. Papers were included if: they presented empirical research published after 2005; participants had malignant disease, diagnosed aged 13-24 years (for over 50% of participants); the research addressed any area of clinical communication; and the research included supporters (parents, partners, carers, friends etc). Papers were excluded if they were: conference abstracts, unpublished articles, systematic reviews, single case studies, validation research methodology, studies using retrospective documentation in clinical notes, articles focusing on information needs rather than communication skills, or were not in English.

A review-specific data extraction form was used to record participant characteristics and methods from each included paper and results relevant to the three review questions. The final number of included articles totalled 36, the remaining 136 were excluded based on the participants' ages, focus on HCPs or information giving. In tandem to the data extraction process, two members of the review team (E.C. and D.J.C.) independently assessed each paper in terms of its internal validity, appropriateness, and contribution to answering the review questions, using a review-specific version of Gough's Weight of Evidence criteria.(23) Discrepancies in assessment decisions were discussed between reviewers and final scores were agreed through consensus.

Extracted data were entered into Excel to aid the narrative synthesis of the included papers.(21,22) All articles, irrespective of relevance and quality, were included in the review.

For peer review only - http://bmjopen.bmj.com/site/about/guidelines.xhtml

However, those rated 'medium' and 'high' were given greater weight in the synthesis. An inductive thematic analysis was undertaken to identify the main, recurrent, and important data across the studies related to answering each research question. D.J.C. and E.C. explored heterogeneity across the studies. The integration of results from studies utilising different methods and epistemological positions was supported by L.A.M.S. and R.M.T., and consensus in synthesis was reached. The synthesis was further refined through discussion of the review of results and their implications with clinicians, interdisciplinary academic audiences, and all of the co-authors.

Patient and Public Involvement Statement

None

Results

A total of 8,480 studies were identified in the search, of which 36 fulfilled the inclusion criteria (Figure 1). The included articles are summarised in Table 2. (table 2 uploaded separately)

All points across the cancer trajectory were represented in the final papers: diagnosis (n=7); (12,24–29) on treatment (n=17); (30–46) end of treatment (completed within one year) (n=2); (47,48) survivorship (more than one-year post-treatment) (n=2); (5,49) and end of life care (n=5). (50–54) Three studies included patients at more than one point along the cancer care continuum. (55–57) Most studies (n=19) were conducted in the US (24,27–29,31,35–37,39–46,50,52,54) other countries included the UK, (25,32,33) Australia, (38,48,49,57) Norway, (12,53) Israel, (47) Iran, (30) Mexico, (51) France, (34) Denmark, (26) Korea (56) and Taiwan, (55) one study recruited from three European countries. (5) Studies used predominantly qualitative methods (n=32) but there were two mixed methods studies and two using quantitative methods. Weight of evidence (WoE) criteria indicated five were high evidence, (24,31,35,45,56) twenty-four were medium (5,12,25,27–30,32–34,36,37,39–42,44,46,47,49–51,55,57) and seven were low evidence. (26,38,43,48,52–54) We used Gough's review specific criteria to weight the quality of each paper. (23) To do this, we used three parameters:

A) The integrity of the evidence on its own terms
B) The appropriateness of the method for answering the review questions
C) The appropriateness of the focus or relevance for answering the review questions

Each of the above was either rated as low, medium, or high. These 3 parameters were combined to create WoE D which was the overall rating seen above and is the extent to which a study contributes evidence to answering the review questions. Factors that made the method highly appropriate included the use of semi-structured interviews to understand TYAC experiences and speaking to the TYAC and supporter separately. The high scoring papers included papers that focussed on communication in the triad, but this only occurred in 10 papers. In 9 papers the age at diagnosis was not specified and this decreased the weighting of these papers. (5,34–36,50–54)

Of the included studies just less than one third researched the triad (n=10) of TYAC, supporters and HCP (5,24,30–32,34–36,50,51), one third TYAC only (n=12)

For peer review only - http://bmjopen.bmj.com/site/about/guidelines.xhtml

(28,29,33,37,38,40–42,44–46,48) and just over a third TYAC and supporters (n=14). (12,25–27,39,43,47,49,52–57)

Table 3 Study population
Participants included in the study and numbers of papers included for each of the three participant groups

Triad? Dyad? Single?	Who is studied in the paper?	Number of papers	References
Triad	TYAC, supporter, HCA	10	(5,24,30–32,34–36,50,51)
Dyad	TYAC and supporter	14	(12,25–27,39,43,47,49,52–57)
Single	TYAC only	12	(28,29,33,37,38,40–42,44–46,48)

The categories used to separate the age groups were lower adolescence (11-14 years), middle adolescence (15-17 years), upper adolescence (18-21 years) and emerging adulthood (22 onwards). Of the papers where the age range at diagnosis could be deduced, the majority of these (21 out of 24) spanned three or more age categories. All the papers spanned two or more age categories. In nine of the papers, the age ranges at diagnosis were not available (as age at diagnosis was expressed as a mean or median). Given these factors, it is difficult to ascertain whether any between age group differences exist.

Who is present with TYACs in healthcare consultations and communication?

The majority of supporters were mothers (68.9%). When combined, parents represented nearly all the supporters in the included studies (94.6%), see Table 4. Non-parental supporters (1.8%) included partners, sisters, aunts, and grandmothers. The remaining supporters were not categorised due to insufficient information in the article's demographics data (3.9%).(53,54)

For peer review only

Table 4 Supporter Demographics
Details of the supporter demographics and percentages of within the included publications

Supporter type	Number of supporters	Percentage quoted to 1 decimal place (%)	References
**"Mother"**	453	68.9	(5,12,24–27,30–32,34,36,39,43,47,49–52,54,55)
**"Father"**	128	19.5	(5,12,25–27,30–32,34,36,39,43,47,49–52,55–57)
**"Both parents"**	20	3.0	(12,32,34,36,55)
**"Parents" no further specification**	20	3.0	(35)
**"Stepmother"**	1	0.2	(57)
**"Grandmother"**	2	0.3	(24)
**"Sister"**	3	0.5	(12,30,51)
**"Partner"**	3	0.5	(25,52)
**"Aunt"**	3	0.5	(36,51,52)
**"Supporters" no further specification**	21	3.2	(53,54)
**"Other"**	3	0.5	(55)
**Total**	657	100.1	

review only

What are TYACs' experiences of communication with the supporter present?

The presence of supporters was concurrently helpful and challenging for TYACs. Supporters undertook several helpful roles and responsibilities: they asked questions on behalf of the TYAC, retained information from HCPs, acted as a conduit of information between the TYAC and HCP, and acted as a ''sounding board'' for the young person.(25,31,45) Some supporters promoted self-advocacy and autonomy for the young person.(27,39,41,46,57) Some reported symptoms on their behalf (45) and proactively negotiated changes to treatment schedules in the interest of the young person.(39)

Findings also suggested that young people could experience limited or ineffective communication in the presence of a supporter. Communication could be directed towards the supporter, not the young person.(27,29,31,36) Supporters could receive information in the absence of the TYAC and subsequently filter the content before delivering the information to TYACs.(30,33,34,55,56): *"The parents had hidden a truth that was not theirs to hide"*p533.(34) This reflected the broader predicament that supporters' priorities at times might have competed with those of young people. (25,34,50,51) Supporters could dominate the communication encounter, for instance, parents were seen to interrupt young people, especially when time was limited. (51) Frederick et al found the mean time for adolescent to clinician communication was only 5.5% of the total consultation and parent conversation

turns directed towards clinicians comprised a mean of 37.5% of all conversation turns. Clinicians directed most communication at the parent rather than the adolescent and spoke for 66.9% of the conversation and none of the clinicians offered patients the opportunity to speak with them alone. (35)

Mutual protectionism appeared to occur, with TYACs and supporters seeking to protect each other from difficult information leading to non-disclosure when both were present. A diagnosis of cancer is devastating for the young person, supporter(s), family, and the wider social network. Repeatedly, there were references to reduced disclosure between the young person and their supporter, in an attempt to shield each other from emotional distress.(12,31,36,38,39,41,45,53,56) TYACs could experience discomfort and guilt in seeing parents tearful and worried, and felt a burden in response to observing the emotions of supporters.(38,39,52) Some TYACs sought to limit this by withholding concerns to protect their supporters: "*I couldn't talk to mum about my concerns because I didn't want to hurt her*" p 37.(38) In equal measure, supporters were characterised as working hard to stay in control of emotions, be strong and stay in the "now", and they channelled energy into helping.(12,31,56) Yet this could contribute to an environment of non-disclosure that had the potential to create future communication challenges, such as supporters not knowing the young person's wishes. Examples of this were evident within the end of life care studies.(52,53) Friebert et al found that 86% of young people wanted to receive prognostic information as soon as possible but only 39% of families knew that.(52) Similarly, Jacobs et al found that young people's end of life wishes were not known by their families.(53) In instances where the young person may not be able to communicate, it may help families relieve the impossible burden of making difficult decisions or feelings of regret, if the young person's perspective and wishes are known.(54)

What is the impact of a TYAC's supporter being present in the communication?

Supporters have the potential to facilitate, complicate or obstruct the young person's involvement in decision-making. Involvement had a positive impact on recall,(42) and may improve autonomy, efficacy, adherence, and future self-management.(24,57) However, the participation of supporters may be experienced as stressful by TYAC as they may become side-lined. (25,40,55) The presence of supporters impacted the young person's level of involvement in decision-making in several ways. In some cases, supporters empowered TYACs to make decisions by withholding their opinion (27) and deferring the final decision to TYACs.(31) However, supporters and TYACs did not perceive decision-making in the same way.(47,56) Supporters believed that young people oversaw decisions about their care; however, this was not what young people recounted.(24) TYACs reported a lack of communication and limited involvement in decisions (24,29,30,46) associated later with decisional regret.(24,37)

Deferral of communication and decisions from the young person to supporters was commonplace.(27,31,36) When supporters responded to this pathway of communication, young people then did not see a need to participate in decisions, knowing that their supporter was taking the mantle.(36) In parallel, clinicians were found to direct communication towards supporters and in extreme cases young people were completely excluded from communication and decisions. (29,30,35,47) An atmosphere characterised by a lack of trust,

For peer review only - http://bmjopen.bmj.com/site/about/guidelines.xhtml

unanswered questions and uncertainty contributed to the exclusion of young people who then sought information from other sources.(30,36,39,56) Not allowing TYACs to choose their involvement in decision-making violated their autonomy, and increased distrust or resentment of providers and supporters and resulted in lower treatment adherence.(30,36,39)

The decisional involvement preferences of young people were not static: they were context and environment dependent. At diagnosis, heightened emotions and poor health rendered young people unable to engage in communication. (24,25,27,29,31,37,41) TYACs expressed a desire to be involved in decision making at different levels: some wanted limited involvement from their supporter(s) so they could take the leading role in consultations and their care;(58) several wanted collaboration with supporters and clinicians;(26,27,44,57) and some completely relied on supporters and HCP's to make decisions on their behalf.(45,46) Davies et al described this as agency, the ability to make free and independent choices. They highlighted the normality of this fluctuation between personal (acting independently), proxy (decisions made on behalf of someone) and collective (decisions are shared) decision making. Whilst this was not always linear, it was part of the cancer trajectory and demonstrated the fluctuating personal agency for TYACs.(32) Some young people reported that supporters and clinicians decided on the their level of involvement in communication and decision-making,(55) and TYACs commented that they did not feel the decision was theirs.(47) Decisional involvement was an interactive, complex, and multifaceted process within the context of the triad, and young people often wanted to be in control of their level of involvement.(28,31) The evidence highlighted that in the presence of a supporter, young people's choice in the their level of involvement in decisions was challenged and not routinely achieved.

Most TYACs felt that it was important for the healthcare team to communicate with them directly and openly.(30,31,33,38,39,49,50) Time alone helped facilitate communication between TYAC and HCP, to ensure that the young person's needs were fully met.(31,36) However, time alone with HCPs was not routinely integrated as a part of consultations with TYACs**.** (35,48) In fact, clinicians were reported as frequently speaking more to parents and TYACs received limited communication from HCPs.(27,31,35,36) In the presence of supporters, as well as withholding concerning information, young people reported feeling discomfort when discussing sensitive topics such as sex or fertility preservation.(27,36)

Young people wanted time alone to communicate with HCPs directly for a variety of reasons. This private line of communication offered a sense of personal agency and allowed them to feel "in the loop" and promoted a sense of autonomy that was threatened by the cancer diagnosis, particularly at the point of diagnosis.(32,50) Young people wanted space to think and privacy during the cancer journey; private lines of communication with HCPs actively promoted this.(31,39,45,46) It also enabled HCPs to get to know the young person and allowed them to ask questions that they may be reluctant to ask in the presence of their supporter, because of embarrassment or emotional shielding.(31) Darabos et al found that 87.5% of oncology providers considered it important to talk to the TYAC without their parents present.(31) Whilst the importance has been highlighted within the data it is also evident that this does not happen as part of routine clinical practice. This could be for several reasons such

For peer review only - http://bmjopen.bmj.com/site/about/guidelines.xhtml

as not wanting to challenge rules of authority, uncertainty around how best to ask a parent to leave and lack of confidence when communicating with a young person alone.

Discussion

Principal Findings

Who is present with TYACs in healthcare consultations and communication? For example, who are the supporters?

The included papers in our review demonstrated that most supporters were parents, more commonly mothers. The frequent presence of mothers in consultations is consistent with previous findings. For example, in a UK study in which TYAC nominated a caregiver, 85% were parents, and of those 80% were female.(59) We note that there is a paucity of data for non-parental supporters, and this may represent a reality of clinical practice or a bias towards TYAC-parental dyads over other relational-dyads in this field of research to date.

What are TYACs' experiences of communication with the supporter present?

TYACs experienced supporters facilitating communication by obtaining information, asking questions, advocating, and supporting personal agency of the young person; conversely supporters could hinder communication by gatekeeping information, or dominating communication and thereby rendering young people as bystanders. Young people experienced negative emotions in response to witnessing their supporters in distress.

What is the impact of a TYAC's supporter being present in the communication?

Bidirectional non-disclosure was a coping strategy used by both TYACs and supporters to protect one another from concerns and emotional burden. This limited HCPs ability to effectively assess ideas, concerns, and expectations from both parties when together. In the presence of supporters some young people were less informed, which could impair their ability to engage in decision-making conversations.

Meaning of the study

This is the first review to look specifically at triadic communication in teenagers and young adults with cancer and has demonstrated that there is a paucity of evidence focussed specifically on triadic communication with TYACs. Of the thirty-six studies in the review less than one third included all three parties in the triadic communication encounter. However, the review has enabled us to provide answers to the review questions and identify knowledge gaps, including a lack of theory describing triadic communication. Some preliminary theoretical models, such as family involvement in interpersonal healthcare processes,(60) depict the interaction pathways between patients, families and HCP and hypothesise the influence of family on interpersonal processes and outcomes of medical consultations.

For peer review only - http://bmjopen.bmj.com/site/about/guidelines.xhtml

The data has clearly identified that parents are the predominating supporter for TYACs, which may be surprising given the inclusion of participants up to the age of 25. Parents can play a significant role when a young person is diagnosed with cancer. Developmentally, a major characteristic that differentiates TYACs from younger children or older adults is the progressive increase in their desire and capacity for independence, personal agency, and autonomy. This process is disrupted by a cancer diagnosis: increased parental presence can be perceived as intrusive and reflect reversion to an earlier family dynamic, anchoring TYACs in dependency, restricting self-exploration, and limiting their developing of an internal value and belief system.(38,61–63) This has been phrased as 'retreating to family' and can negatively impact peer relationships by impeding development and maintenance of a peer network.(40,64,65) Young people may often be accepting of this, particularly in the early stages of the cancer diagnosis. However, as this review demonstrates, the presence of parents alters the experience and impact of communication with HCPs. It is important to highlight that there is limited literature on TYAC communication encounters with supporters other than parents.(62,66,67) Partners felt relegated to a non-participatory role by a parent, and mothers struggled to relinquish their existing role as primary supporter.(62,67) It is relevant to note that the participants in these three studies were in their early 20's.

A key impact of triadic communication is that young people may not be involved in decision making to the level they want. This is consistent with related paediatric oncology literature which consistently reports children's limited participation in decision-making. (68–70) Clinicians attempted to protect children from 'too much' information because of the perception that children are not capable or too vulnerable.(17) The important difference between paediatric and TYAC populations are the legal and ethical obligations towards TYACs who are autonomous, capacitous patients rather than to parents with parental responsibility.

The findings of this review demonstrate the presence of a supporter impacts the involvement of young people in healthcare decisions. Therefore, there are legal and ethical issues, which are critically important, both in research and clinically in TYAC care particularly related to informed consent, capacity, and autonomy. The law relating to children and young people is complex and differs across the UK and internationally. The General Medical Council guidelines in the UK state, "the patient must be the first concern".(71) HCPs have ethical and legal obligations outlined in UK best practice guidance, statute, and case law.(72) In the UK, parents can legally make decisions for children under 16 years unless the child disagrees and is deemed 'Gillick Competent'.(73) Moreover, studies have shown children aged 14 and older can approach the level of understanding of adults.(74,75) In contrast, people aged 16 and above are legally able to make decisions for themselves in the UK and are automatically assumed to have capacity (76) and therefore, HCPs must communicate with them in developmentally appropriate ways. Clinicians face a challenge in identifying the best way to communicate with TYACs and their supporter (s). TYACs need parental involvement whilst simultaneously desiring autonomy (36) necessitating careful balancing of the needs of both parties to ensure that the young person is not relegated to a non-participant status.

Strengths and weaknesses

For peer review only - http://bmjopen.bmj.com/site/about/guidelines.xhtml

Our review had a number of limitations. We searched systematically and thoroughly for eligible studies, but this is not a well-indexed field of research, and therefore it is possible that some relevant studies were not included in the review. We limited the review to a UK TYAC age range and not the broader age used elsewhere, so the conclusions are applicable to younger adults, up to aged 24 only and not necessarily the age of young adulthood used in some countries (between 29 to 39). We also only included papers published in English and therefore papers reflect practices in primarily North America, Australia and Europe, the results may not be applicable to other countries especially where cultural differences affect parental-TYAC or other familial/romantic relational dynamics and where the healthcare culture may be different, e.g., more paternalistic. Despite these limitations, international representation was seen in the eligible studies, TYAC ages were included across the entirety of the specified UK age range and studies represented the journey throughout the cancer experience.

Implications for clinicians and policy makers

Given the degree of challenge and nuance raised, HCPs need training on effective triadic communication. Fourneret concluded that the relationship between TYACs, their parents and HCPs "as being the most difficult one in oncology".(34) Professionals described challenges communicating with both TYACs and parents, especially when loyalties were torn between the two.(5) However, training is currently ad hoc and not interdisciplinary.(77–80) Furthermore, HCPs can find it difficult to apply teaching in this area in clinical practice.(53,81) HCPs need education and training to navigate triadic communication to optimise involvement of the young person whilst attending to a supporter's needs. Experiential learning is the gold standard in teaching methods for clinical communication and is designed to bring about changes in learners' skills. These evidence-based methods are through small group, problem-based simulation in a classroom, with repeated practise and rehearsal of skills under observation with detailed and descriptive feedback. This is arguably warranted here.(82,83)

Triadic communication is a key feature of TYAC care but requires further attention and inclusion in future iterations of key policy documents and guidelines such as the Blueprint of Care (BoC).(84) The BoC is a UK document that helps shape and deliver developmentally appropriate care to TYAC. However, it is recognised that age is poorly correlated with developmental maturity and therefore any communication framework needs to be specific to TYACs, recognising the transitional nature of adolescence meaning a one size fits all approach is likely inadequate.

Unanswered questions and future research

Future research is warranted to triangulate triadic perspectives and understand more about the interactional dynamics of these complex communication encounters. A key research need is investigating how best to support decision-making whilst engaging supporters, understanding their priorities and information needs may conflict.(31,36,37,40) Conflict management must also be understood in the emotional context of young adult oncology. How to effectively educate HCPs to communicate within the triad, to ensure the young person and the supporters needs are met is a priority. This needs to include how best we facilitate time

For peer review only - http://bmjopen.bmj.com/site/about/guidelines.xhtml

alone between young patients and HCPs. Continued development and utilisation of comprehensive triadic theoretical frameworks may provide guidance and direction for future research, allowing for greater integration and progress with this diverse research area and commonly occurring form of healthcare communication.

Conclusion

Triadic communication is a pivotal component of communicating with TYACs and the presence of supporters impacts clinical communication both positively and negatively. Young people desire a sense of personal agency, autonomy and control related to information flow and decision making. This includes private lines of communication with HCPs without the presence of supporters. HCPs recognise the importance of time alone with young people; however this does not translate to clinical practice. Therefore, further research on communication dynamics is needed to allow for the development of bespoke, TYAC focussed clinical communication training for HCPs to allow them to effectively facilitate and navigate triadic communication. This then needs to be formally embedded in national guidance and postgraduate training for HCPs working in TYAC care to allow equitable access for TYACs.

Author Contributions:
DC, LS and RT were involved in developing the protocol; DC, IK and LS coordinated the running of the study and were responsible for data acquisition; DC, LS, RT and EC contributed to the analysis; DC drafted the manuscript. All authors have critically reviewed the manuscript for important intellectual content and have read and agreed to the published version of the manuscript. All authors agree to be accountable for all aspects of the work in ensuring that questions related to the accuracy or integrity of any part of the work are appropriately investigated and resolved.

Conflicts of Interest:
The authors declare no conflict of interest.

Ethics Approval:
This was a systematic review and therefore ethics approval was not required.

Funding:
This paper presents work supported by the Wellcome Trust, (grant number G115288) under its Programme PhD for healthcare professionals course awarded to the first author Deborah Critoph, University of Cambridge. R.M.T. is partially funded through UCLH Charity. The views expressed are those of the author (s) and not necessarily those of the Wellcome Trust or UCLH Charity.

Data availability statement:
All data relevant to the study are included in the article or uploaded as supplementary information. No previously unpublished primary data are included in the paper. All data

For peer review only

relevant to the systematic review are included in the paper or uploaded as supplementary information.

Figure Legends:

Figure 1: PRISMA Flow diagram

Table 1: Search terms

Table 2: Summary of articles

Table 3: Study Population
Participants included in the study and numbers of papers included for each of the three participant groups

Table 4: Supporter Demographics
Details of the supporter demographics and percentages of within the included publications

For peer review only

References:

1. Cancer Research UK. Young peoples' cancers incidence statistics [Internet]. 2016 [cited 2019 Jul 10]. Available from: https://www.cancerresearchuk.org/health-professional/cancer-statistics/young-people-cancers/incidence
2. National Institute of Health and Clinical Excellence. Guidance on Cancer Services Improving Outcomes in Children and Young People with Cancer National Institute for Health and Clinical Excellence [Internet]. Nice. 2005. 198 p. Available from: https://www.nice.org.uk/guidance/csg7/resources/improving-outcomes-in-children-and-young-people-with-cancer-update-pdf-773378893
3. Care Quality Commission., NHS Patient Survey Programme. 2018 Children and Young People's patient experience survey. Statistical Release. 2019;(November).
4. White B, Viner RM. Improving communication with adolescents. Arch Dis Child Educ Pract Ed. 2012;97(3):93–7.
5. Essig S, Steiner C, Kuehni CE, Weber H, Kiss A. Improving Communication in Adolescent Cancer Care: A Multiperspective Study. Pediatr Blood Cancer [Internet]. 2016;63(8):1423–30. Available from: http://onlinelibrary.wiley.com/journal/10.1002/(ISSN)1545-5017
6. Lea S, Gibson F, Taylor RM. Holistic Competence": How Is it Developed, Shared, and Shaped by Health Care Professionals Caring for Adolescents and Young Adults with Cancer? J Adolesc Young Adult Oncol. 2021;10(5):503–11.
7. Gibson F, Aldiss S, Fern LA, Phillips B, Gravestock H, Malik S, et al. Reporting the whole story: Analysis of the "out-of-scope" questions from the James Lind Alliance Teenage and Young Adult Cancer Priority Setting Partnership Survey. Health Expect. 2021;

8. Smith LAM, Critoph DJ, Hatcher HM. How Can Health Care Professionals Communicate Effectively with Adolescent and Young Adults Who Have Completed Cancer Treatment? A Systematic Review. J Adolesc Young Adult Oncol [Internet]. 2020 Jan 14; Available from: https://doi.org/10.1089/jayao.2019.0133
9. Reid J, McKeaveney C, Martin P. Communicating with Adolescents and Young Adults about Cancer-Associated Weight Loss. Curr Oncol Rep. 2019;21(2):6.
10. Phillips CR, Haase JE. A Connectedness Primer for Healthcare Providers: Adolescents/Young Adult Cancer Survivors' Perspectives on Behaviors That Foster Connectedness during Cancer Treatment and the Resulting Positive Outcomes. J Adolesc Young Adult Oncol [Internet]. 2018;7(2):174–80. Available from: http://www.liebertpub.com/products/product.aspx?pid=387
11. Mellblom A V, Finset A, Korsvold L, Loge JH, Ruud E, Lie HC. Emotional concerns in follow-up consultations between paediatric oncologists and adolescent survivors: a video-based observational study. Psychooncology [Internet]. 2014 Dec;23(12):1365–72. Available from: https://search.ebscohost.com/login.aspx?direct=true&AuthType=athens&db=cin20&AN=109768272&site=ehost-live
12. Korsvold L, Mellblom AV, Finset A, Ruud E, Lie HC. A content analysis of emotional concerns expressed at the time of receiving a cancer diagnosis: An observational study of consultations with adolescent and young adult patients and their family members. European Journal of Oncology Nursing [Internet]. 2017;26:1–8. Available from: https://search.ebscohost.com/login.aspx?direct=true&AuthType=sso&db=cin20&AN=120589318&site=ehost-live&custid=ns123475
13. Ward A, Critoph D, Westacott R, Williams R, Dogra N. A collaboration on teaching and assessing triadic consultation skills. PEC Innovation [Internet]. 2022;1(October):100091. Available from: https://doi.org/10.1016/j.pecinn.2022.100091
14. van Staa AL. Unraveling triadic communication in hospital consultations with adolescents with chronic conditions: The added value of mixed methods research. Patient Educ Couns. 2011;82(3):455–64.
15. Cahill P, Papageorgiou A. Triadic communication in the primary care paediatric consultation: A review of the literature. British Journal of General Practice. 2007;57(544):904–11.
16. Maras M. Hidden in plain sight. Int J Psychiatry Med. 2022;57(6):481–5.
17. Young B, Dixon-woods M, Windridge KC, Heney D. Study of Patients and Parents. Br Med J. 2003;326(7384):305.
18. Wolff JL, Roter DL. Family presence in routine medical visits: A meta-analytical review. Soc Sci Med [Internet]. 2011;72(6):823–31. Available from: http://dx.doi.org/10.1016/j.socscimed.2011.01.015
19. Laidsaar-Powell RC, Butow PN, Bu S, Charles C, Gafni A, Lam WWT, et al. Physician-patient-companion communication and decision-making: A systematic review of triadic medical consultations. Patient Educ Couns. 2013;91(1):3–13.
20. Tates K, Meeuwesen L. Doctor-parent-child communication. A (re)view of the literature. Soc Sci Med. 2001;52(6):839–51.
21. Popay J, Roberts, H SA et al. Guidance on the Conduct of Narrative Synthesis in Systematic Reviews.

For peer review only

22. Petticrew M RH. Systematic Reviews in the social sciences. In: Systematic reviews in the social sciences [Internet]. Available from: https://onlinelibrary.wiley.com/doi/book/10.1002/9780470754887
23. Gough D. Weight of Evidence: a framework for the appraisal of the quality and relevance of evidence. 2007 [cited 2023 Jul 2]; Available from: https://www.tandfonline.com/action/journalInformation?journalCode=rred20
24. Barakat LP, Schwartz LA, Reilly A, Deatrick JA, Balis F. A Qualitative Study of Phase III Cancer Clinical Trial Enrollment Decision-Making: Perspectives from Adolescents, Young Adults, Caregivers, and Providers. J Adolesc Young Adult Oncol. 2014;3(1):3–11.
25. Hart RI, Cameron DA, Cowie FJ, Harden J, Heaney NB, Rankin D, et al. The challenges of making informed decisions about treatment and trial participation following a cancer diagnosis: a qualitative study involving adolescents and young adults with cancer and their caregivers. BMC Health Serv Res. 2020;20(1).
26. Ingersgaard MV, Tulstrup M, Larsen HB, Schmiegelow Kjeld AO - Ingersgaard MVO http://orcid. org/0000 0002 7037 2104. A qualitative study of decision-making on Phase III randomized clinical trial participation in paediatric oncology: Adolescents' and parents' perspectives and preferences. J Adv Nurs. 2018;74(1):110–8.
27. Olsavsky AL, Theroux CI, Dattilo TM, Klosky JL, O'Brien SH, Quinn GP, et al. Family communication about fertility preservation in adolescent males newly diagnosed with cancer. Pediatr Blood Cancer. 2021;68(7).
28. Weaver MS, Baker JN, Gibson D V, Gattuso JS, Hinds PS, Gibson D V, et al. "Being a good patient" during times of illness as defined by adolescent patients with cancer. Cancer [Internet]. 2016 Jul 15;122(14):2224–33. Available from: http://onlinelibrary.wiley.com/journal/10.1002/(ISSN)1097-0142
29. Mobley EM, Thomas SM, Brailsford J, Ochoa CY, Miller K, Applebaum A, et al. Clinical Trial Participation: A Qualitative Study of Adolescents and Younger Adults Recently Diagnosed with Cancer. J Adolesc Young Adult Oncol. 2023 Jun 1;12(3):303–13.
30. Bahrami M, Namnabati M, Mokarian F, Oujian P, Arbon P. Information-sharing challenges between adolescents with cancer, their parents and health care providers: a qualitative study. Supportive Care in Cancer [Internet]. 2017;25(5):1587–96. Available from: https://search.ebscohost.com/login.aspx?direct=true&AuthType=athens&db=cin20&AN=122279626&site=ehost-live
31. Darabos K, Berger AJ, Barakat LP, Schwartz LA. Cancer-Related Decision-Making Among Adolescents, Young Adults, Caregivers, and Oncology Providers. Qual Health Res. 2021 Nov 1;31(13):2355–63.
32. Davies J, Kelly D, Hannigan B. "Life then", "life interrupted", "life reclaimed": The fluctuation of agency in teenagers and young adults with cancer. Eur J Oncol Nurs. 2018;36(100885136):48–55.
33. Fern LA, Taylor RM, Whelan J, Pearce S, Grew T, Brooman K, et al. The Art of Age-Appropriate Care. Cancer Nurs [Internet]. 2013;36(5):E27–38. Available from: https://search.ebscohost.com/login.aspx?direct=true&AuthType=athens&db=cin20&AN=110243702&site=ehost-live
34. Fourneret E. Breaking Bad News about Cancer to Adolescents and Young Adults: The French Experience. J Law Med [Internet]. 2018 [cited 2021 Nov 22];25(2):530–7. Available from: https://pubmed.ncbi.nlm.nih.gov/29978652/

For peer review only - http://bmjopen.bmj.com/site/about/guidelines.xhtml

35. Frederick NN, Mack JW. Adolescent patient involvement in discussions about relapsed or refractory cancer with oncology clinicians. Pediatr Blood Cancer [Internet]. 2018;65(4):1. Available from: https://search.ebscohost.com/login.aspx?direct=true&AuthType=athens&db=cin20&AN=128132562&site=ehost-live
36. Hong MK, Wilcox L, Machado D, Olson TA, Simoneaux SF. Care Partnerships: Toward Technology to Support Teens' Participation in Their Health Care. Proc SIGCHI Conf Hum Factor Comput Syst [Internet]. 2016 May 7 [cited 2018 Jun 2];2016:5337–49. Available from: http://www.ncbi.nlm.nih.gov/pubmed/28164178
37. Mack JW, Fasciano KM, Block SD. Adolescent and Young Adult Cancer Patients' Experiences With Treatment Decision-making. Pediatrics. 2019;143(5).
38. Patterson P, Millar B, Desille N, McDonald F. The Unmet Needs of Emerging Adults With a Cancer Diagnosis A Qualitative Study. Cancer Nurs. 2012;35(3):E32–40.
39. Pennant S, Lee SC, Holm S, Triplett KN, Howe-Martin L, Campbell R, et al. The Role of Social Support in Adolescent/Young Adults Coping with Cancer Treatment. Children [Internet]. 2020;7(1):1–25. Available from: https://search.ebscohost.com/login.aspx?direct=true&AuthType=athens&db=cin20&AN=142468088&site=ehost-live
40. Pyke-Grimm KA, Franck LS, Halpern-Felsher B, Goldsby RE, Rehm RS. 3 Dimensions of Treatment Decision Making in Adolescents and Young Adults With Cancer. Cancer Nurs [Internet]. 2020 Nov;43(6):436–45. Available from: https://search.ebscohost.com/login.aspx?direct=true&AuthType=athens&db=cin20&AN=146822019&site=ehost-live
41. Pyke-Grimm KA, Franck LS, Halpern-Felsher B, Goldsby RE, Rehm RS. Day-to-Day Decision Making by Adolescents and Young Adults with Cancer. Journal of Pediatric Hematology/Oncology Nursing [Internet]. 2022;39(5):290–303. Available from: https://ovidsp.ovid.com/athens/ovidweb.cgi?T=JS&CSC=Y&NEWS=N&PAGE=fulltext&D=medl&AN=35538622 https://cambridge-primo.hosted.exlibrisgroup.com/openurl/44CAM/44CAM_services_page?sid=OVID:medline&id=pmid:35538622&id=doi:10.1177%2F27527530211068718&issn=
42. Zarnegar S, Gosiengfiao Y, Rademaker A, Casey R, Albritton KH. Recall of Fertility Discussion by Adolescent Female Cancer Patients: A Survey-Based Pilot Study. J Adolesc Young Adult Oncol [Internet]. 2018 Apr;7(2):249–53. Available from: http://www.liebertpub.com/products/product.aspx?pid=387
43. Viola A, Taggi-Pinto A, Sahler OJZ, Alderfer MA, Devine KA, Ed M, et al. Problem-solving skills, parent-adolescent communication, dyadic functioning, and distress among adolescents with cancer. Pediatr Blood Cancer. 2018;65(5).
44. Weaver MS, Baker JN, Gibson D V, Gattuso JS, Sykes AD, Hinds PS. Adolescents' preferences for treatment decisional involvement during their cancer. Cancer [Internet]. 2015;121(24):4416–24. Available from: http://onlinelibrary.wiley.com/journal/10.1002/(ISSN)1097-0142
45. Sisk BA, Keenan M, Kaye EC, Baker JN, Mack JW, DuBois JM. Co-management of communication and care in adolescent and young adult oncology. Pediatr Blood Cancer [Internet]. 2022;69(10):e29813. Available from: https://ovidsp.ovid.com/athens/ovidweb.cgi?T=JS&CSC=Y&NEWS=N&PAGE=fulltext&D=mesx&AN=35719025 https://cambridge-

For peer review only - http://bmjopen.bmj.com/site/about/guidelines.xhtml

primo.hosted.exlibrisgroup.com/openurl/44CAM/44CAM_services_page?sid=OVID:medline&id=pmid:35719025&id=doi:10.1002%2Fpbc.29813&issn=1545-500
46. Sisk BA, Keenan M, Schulz GL, Kaye E, Baker JN, Mack JW, et al. Interdependent functions of communication with adolescents and young adults in oncology. Pediatr Blood Cancer [Internet]. 2022; Available from: http://onlinelibrary.wiley.com/journal/10.1002/(ISSN)1545-5017
47. Barlevy D, Wangmo T, Ash S, Elger BS, Ravitsky V. Oncofertility decision making: Findings from Israeli adolescents and parents. J Adolesc Young Adult Oncol [Internet]. 2019;8(1):74–83. Available from: https://www.proquest.com/scholarly-journals/oncofertility-decision-making-findings-israeli/docview/2426222863/se-2?accountid=47868
48. Sawyer SM, McNeil R, Thompson K, Orme LM, McCarthy MAOSSM; O http://orcid.org/0000 0002 9095 358X, Sawyer SM, et al. Developmentally appropriate care for adolescents and young adults with cancer: how well is Australia doing? Supportive Care in Cancer [Internet]. 2019;27(5):1783–92. Available from: http://link.springer.de/link/service/journals/00520/index.htm
49. Ellis SJ, Wakefield CE, McLoone JK, Robertson EG, Cohn RJ. Fertility concerns among child and adolescent cancer survivors and their parents: A qualitative analysis. J Psychosoc Oncol. 2016;34(5):347–62.
50. Ananth P, Mun S, Reffat N, Li R, Sedghi T, Avery M, et al. A Stakeholder-Driven Qualitative Study to Define High Quality End-of-Life Care for Children With Cancer. J Pain Symptom Manage [Internet]. 2021;62(3):492–502. Available from: https://ovidsp.ovid.com/athens/ovidweb.cgi?T=JS&CSC=Y&NEWS=N&PAGE=fulltext&D=med19&AN=33556497 https://cambridge-primo.hosted.exlibrisgroup.com/openurl/44CAM/44CAM_services_page?sid=OVID:medline&id=pmid:33556497&id=doi:10.1016%2Fj.jpainsymman.2021.01.
51. Cicero-Oneto CE, Valdez-Martinez E, Bedolla M. Decision-making on therapeutic futility in Mexican adolescents with cancer: a qualitative study. BMC Med Ethics [Internet]. 2017;18:74. Available from: https://search.ebscohost.com/login.aspx?direct=true&AuthType=athens&db=cin20&AN=127010047&site=ehost-live
52. Friebert S, Grossoehme DH, Baker JN, Needle J, Thompkins JD, Cheng YI, et al. Congruence Gaps Between Adolescents With Cancer and Their Families Regarding Values, Goals, and Beliefs About End-of-Life Care. JAMA Netw Open [Internet]. 2020 May 19;e205424–e205424. Available from: https://search.ebscohost.com/login.aspx?direct=true&AuthType=athens&db=cin20&AN=143389091&site=ehost-live
53. Jacobs S, Perez J, Cheng YI, Sill A, Wang J, Lyon ME. Adolescent end of life preferences and congruence with their parents' preferences: Results of a survey of adolescents with cancer. Pediatr Blood Cancer [Internet]. 2015;62(4):710–4. Available from: https://search.ebscohost.com/login.aspx?direct=true&AuthType=athens&db=cin20&AN=109777063&site=ehost-live
54. Lyon ME, Jacobs S, Briggs L, Cheng YI, Wang J. Family-centered advance care planning for teens with cancer. JAMA Pediatr. 2013;167(5):460–7.
55. Wu LM, Chiou SS, Lin PC, Liao YM, Su HL. Decisional conflicts, anxiety, and perceptions of shared decision-making in cancer treatment trajectory among

For peer review only

adolescents with cancer: A longitudinal study. Journal of Nursing Scholarship. 2022;54(5):589–97.
56. Son H, Miller LE. Family Communication About Cancer in Korea: A Dyadic Analysis of Parent-Adolescent Conversation. Glob Qual Nurs Res. 2023 Jan 1;10.
57. Glackin A, Marino JL, Peate M, McNeil R, Orme LM, McCarthy MC, et al. Experiences of Oncofertility Decision-Making and Care in a National Sample of Adolescent and Young Adult Cancer Patients and Parents. J Adolesc Young Adult Oncol. 2023;
58. Mack JW, Fasciano KM, Block SD. Adolescent and Young Adult Cancer Patients' Experiences With Treatment Decision-making. Pediatrics. 2019;143(5).
59. Martins A, Alvarez-Galvez J, Fern LA, Vindrola-Padros C, Barber JA, Gibson F, et al. The BRIGHTLIGHT National Survey of the Impact of Specialist Teenage and Young Adult Cancer Care on Caregivers' Information and Support Needs. Cancer Nurs. 2021;44(3):235–43.
60. Dalton JM. Development and testing of the theory of collaborative decision-making in nursing practice for triads. J Adv Nurs. 2003;41(1):22–33.
61. Grinyer A. Young people living with cancer:implications for policy and practice. Open University Press; 2007. 182 p.
62. Moules NJ, Laing CM, Estefan A, Schulte F, Guilcher GMT. "Family Is Who They Say They Are"(a): Examining the Effects of Cancer on the Romantic Partners of Adolescents and Young Adults. J Fam Nurs. 2018;24(3):374–404.
63. Silva M, Barretta F, Luksch R, Terenziani M, Casanova M, Spreafico F, et al. Adolescents with cancer on privacy: Fact-finding survey on the need for confidentiality and space. Tumori. 2021 Oct 1;107(5):452–7.
64. KM B, Smith A, Schmidt S, TH K, Zebrack B, CF L, et al. Positive and negative psychosocial impact of being diagnosed with cancer as an adolescent or young adult. Cancer (0008543X) [Internet]. 2012 Oct 15;118(20):5155–62. Available from: https://search.ebscohost.com/login.aspx?direct=true&AuthType=athens&db=cin20&AN=104372613&site=ehost-live
65. Pyke-Grimm KA, Franck LS, Patterson Kelly K, Halpern-Felsher B, Goldsby RE, Kleiman A, et al. Treatment Decision-Making Involvement in Adolescents and Young Adults With Cancer. Oncol Nurs Forum [Internet]. 2019;46(1):E22–37. Available from: https://search.ebscohost.com/login.aspx?direct=true&AuthType=athens&db=cin20&AN=133553348&site=ehost-live
66. Davies J. The experience and role of partners in helping to meet the support needs of adolescents and young adults with cancer. Journal of Advanced Nursing (John Wiley & Sons, Inc) [Internet]. 2019 May;75(5):1119–25. Available from: https://search.ebscohost.com/login.aspx?direct=true&AuthType=athens&db=cin20&AN=135934208&site=ehost-live
67. Iannarino NT. "It's My Job Now, I Guess": Biographical disruption and communication work in supporters of young adult cancer survivors. Commun Monogr [Internet]. 2018;85(4):491–514. Available from: https://doi.org/10.1080/03637751.2018.1468916
68. Coyne I, Amory A, Gibson F, Kiernan G. Information-sharing between healthcare professionals, parents and children with cancer: More than a matter of information exchange. Eur J Cancer Care (Engl). 2016;25(1):141–56.

For peer review only - http://bmjopen.bmj.com/site/about/guidelines.xhtml

69. de Vries MC, Wit JM, Engberts DP, Kaspers GJL, van Leeuwen E. Pediatric Oncologists' Attitudes Towards Involving Adolescents in Decision-Making Concerning Research Participation. Pediatr Blood Cancer. 2010;55(1):123–8.
70. Frederick NN, Bingen K, Bober SL, Cherven B, Xu X, Quinn GP, et al. Pediatric oncology clinician communication about sexual health with adolescents and young adults: A report from the children's oncology group. Cancer Med [Internet]. 2021;10(15):5110–9. Available from: https://ovidsp.ovid.com/athens/ovidweb.cgi?T=JS&CSC=Y&NEWS=N&PAGE=fulltext&D=med20&AN=34128352 https://cambridge-primo.hosted.exlibrisgroup.com/openurl/44CAM/44CAM_services_page?sid=OVID:medline&id=pmid:34128352&id=doi:10.1002%2Fcam4.4077&issn=2045-76
71. General Medical Council. Guidance for All Doctors. 0-18 Years: Guidance for All Doctors [Internet]. 2018;11. Available from: https://www.gmc-uk.org/ethical-guidance/ethical-guidance-for-doctors/0-18-years/making-decisions#paragraph-29
72. British Medical Association. Children and young people toolkit A toolkit for doctors. BMA.org.uk [Internet]. 2021; Available from: https://www.bma.org.uk
73. Gillick T. Gillick competence What is Gillick Here ' s what you need to know about capacity and The Gillick case. 2022;
74. Joffe S, Fernandez C V., Pentz RD, Ungar DR, Mathew NA, Turner CW, et al. Involving children with cancer in decision-making about research participation. Journal of Pediatrics. 2006;149(6).
75. Mårtenson EK, Fägerskiöld AM. A review of children's decision-making competence in health care. J Clin Nurs. 2008;17(23):3131–41.
76. Department for Constitutional Affairs. Mental Capactity Act 2005: Code of Practice. Mental Capactity Act 2005: Code of Practice. 2007;1–301.
77. Lea S, Gibson F, Taylor RM. Holistic Competence": How Is it Developed, Shared, and Shaped by Health Care Professionals Caring for Adolescents and Young Adults with Cancer? J Adolesc Young Adult Oncol. 2021;10(5):503–11.
78. Essig S, Steiner C, Kuehni CE, Weber H, Kiss A. Improving Communication in Adolescent Cancer Care: A Multiperspective Study. Pediatr Blood Cancer. 2016;63(8):1423–30.
79. Essig S, Steiner C, Kühne T, Kremens B, Langewitz W, Kiss A. Communication Skills Training for Professionals Working with Adolescent Patients with Cancer Based on Participants' Needs: A Pilot. https://home.liebertpub.com/jayao. 2019 Jun 4;8(3):354–62.
80. Coad J, Smith J, Pontin D, Gibson F. Consult, Negotiate, and Involve: Evaluation of an Advanced Communication Skills Program for Health Care Professionals. Journal of Pediatric Oncology Nursing. 2018 Jul 1;35(4):296–307.
81. Ruhe KM, Badarau DO, Brazzola P, Hengartner H, Elger BS, Wangmo T, et al. Participation in pediatric oncology: views of child and adolescent patients. Psychooncology. 2016;25(9):1036–42.
82. Blackmore A, Kasfiki EV, Purva M. Simulation-based education to improve communication skills: A systematic review and identification of current best practice. BMJ Simul Technol Enhanc Learn. 2018;4(4):159–64.
83. Mahoney P, Macfarlane S, Ajjawi R. A qualitative synthesis of video feedback in higher education. Teaching in Higher Education [Internet]. 2019;24(2):157–79. Available from: https://doi.org/10.1080/13562517.2018.1471457

For peer review only - http://bmjopen.bmj.com/site/about/guidelines.xhtml

84. Smith S, Mooney S, Cable M, Taylor RM. THE BLUEPRINT OF CARE for teenagers and young adults with cancer Second edition. 2016; Available from: https://www.teenagecancertrust.org/sites/default/files/BlueprintOfCare_2ndEdition.pdf

For peer review only

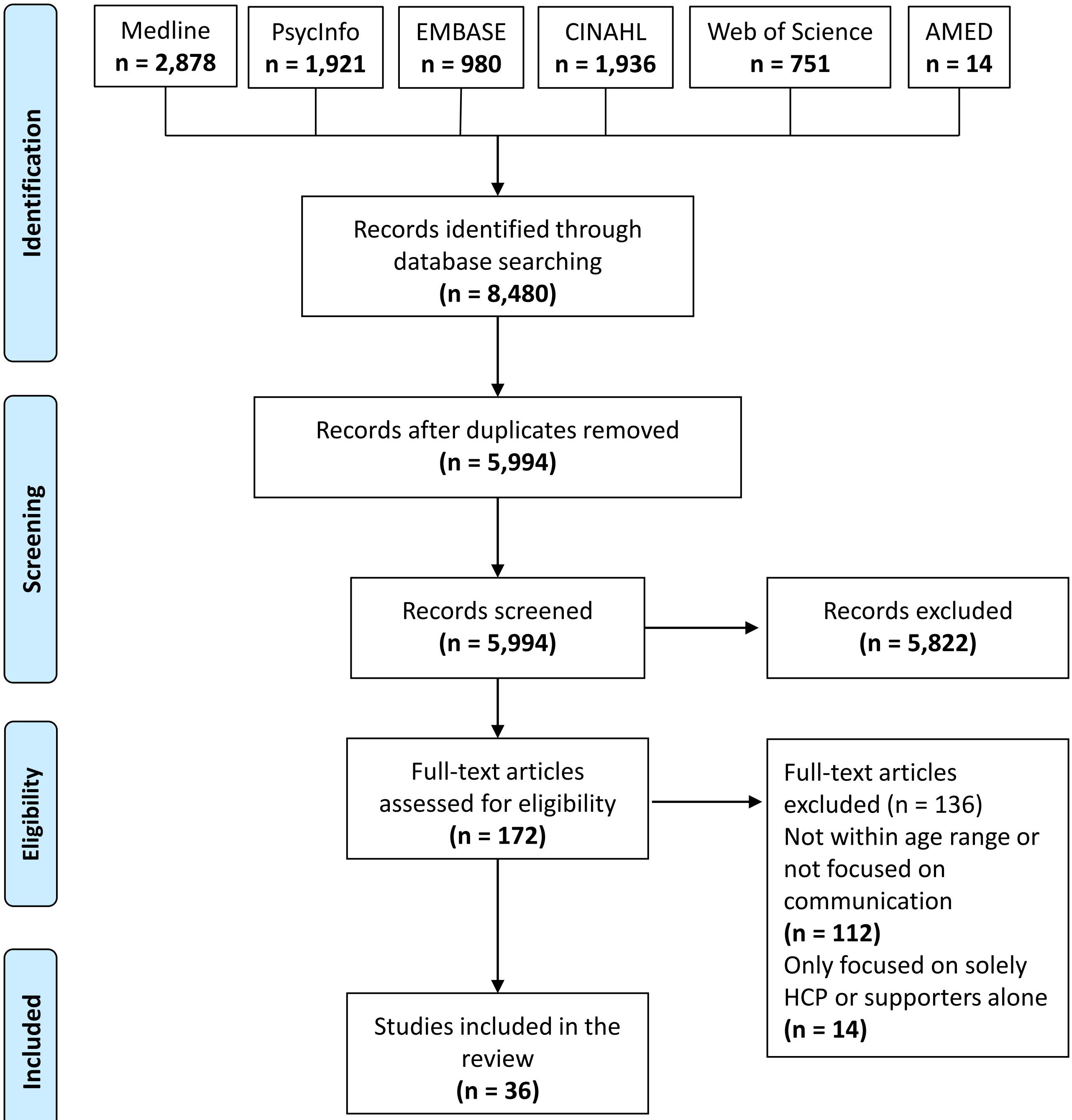
Identification
Screening
Eligibility
Included
Medline n = 2,878
PsycInfo n = 1,921
EMBASE n = 980
CINAHL n = 1,936
Web of Science n = 751
AMED n = 14
Records identified through database searching (n = 8,480)
Records after duplicates removed (n = 5,994)
Records screened (n = 5,994)
Records excluded (n = 5,822)
Full-text articles assessed for eligibility (n = 172)
Full-text articles excluded (n = 136)
Not within age range or not focused on communication (n = 112)
Only focused on solely HCP or supporters alone (n = 14)
Studies included in the review (n = 36)

Table 2. Summary of Articles

First author (year)	Title	Setting	Study Type - analysis method *Data collection*	Focus	Participant Characteristics	Key findings	WoE Score
Ananth (2021)	A Stakeholder-Driven Qualitative Study to Define High Quality End-Of-Life Care for Children with Cancer	USA Multicentre	Qualitative - thematic analysis<br><br>*Semi-structured interviews and focus groups.*	To explore end of life care (EOLC) priorities for children with cancer and their families.	54 participants:<br>**10 AYACs (age range: 17-23 years)**<br>25 parents (including 12 bereaved parents)<br>19 healthcare professionals	Important to have direct communication with the child or young person regarding decision-making.<br>Interdisciplinary care with integrated teams is vital for high quality end of life care. Continuity of healthcare professionals was positive.<br>AYACs would prefer to die at home but family and healthcare professionals may be hesitant.	Medium
Bahrami (2017)	Information Sharing Challenges Between Adolescents with Cancer, their Parents and Health Care Providers: A Qualitative Study	Iran Single centre	Qualitative descriptive-exploratory study - grounded theory analysis<br><br>*Semi-structured interviews.*	Information sharing between AYACs, parents and health professionals.	33 participants:<br>**12 AYACs (age range at interview: 15-20 years, within 1 year of diagnosis)**<br>6 supporters<br>6 healthcare professionals	AYACs feel they are excluded from information-sharing sessions between parents and healthcare professionals. This leads to disaffiliation, confusion and AYACs seek information from 'inferior' sources.<br>Parents were often the first receivers of information allowing them to act as gatekeepers controlling information to flow to AYACs. Parents may want to shield AYACs from bad news.<br>Trust and honesty are the foundations of effective communication between AYACs and healthcare professionals. AYACs reacted negatively towards dishonesty.	Medium
Barakat (2014)	A Qualitative Study of Phase III Cancer Clinical Trial Enrollment Decision Making Perspectives from AYAC, Caregivers and Providers	USA Single centre	Qualitative - thematic analysis<br><br>*Semi-structured interviews.*	Clinical trial enrollment.	40 participants:<br>**13 AYACs (age range: 15-21 years)**<br>16 supporters<br>11 healthcare professionals	Four patterns of decision-making patterns identified:<br>1. AYAC abdicates to caregiver,<br>2. Caregiver based and AYAC approved,<br>3. Collaborative,<br>4. AYAC in charge of decision-making.<br>Caregivers perceived AYAC to be in charge of decision making most of the time whereas the AYACs felt that "AYAC abdicates to carer" was the most common form of decision making.<br>Distress and poor health limited AYAC involvement in the decision.<br>Developmental and emotional maturity facilitated involvement.	High

For peer review only - http://bmjopen.bmj.com/site/about/guidelines.xhtml

Barlevy (2019)	Oncofertility decision making: findings from Israeli adolescents and parents	Israel<br>Single centre	Qualitative- thematic analysis<br><br>*semi-structured interviews*	To understand adolescent oncofertilty decision making in Israel, from perspectives of parents and adolescents	35 participants<br>**16 AYACs (age range 12-16 years)**<br>19 parents	As in other cultural contexts, Israeli adolescents and parents demonstrate multifaceted decision making with respect to oncofertility. A significant finding from this study suggests that health professional shy from discussing posthumous planning of cryopreserved materials with adolescent cancer patients and their parents. 5 out of 16 AYAs felt that the decision was not theirs and that it was instead the parents' or the pysicians' to make. Some parents felt that the decision was made by the clinician - explicit or implicit recommendations from the clinician strongly influence decision making. No decisional regret expressed by any members of the dyad.	Medium
Cicero-Oneto (2017)	Decision-making on therapeutic futility in Mexican adolescents with cancer: a qualitative study	Mexico<br>Multicentre	Qualitative - thematic analysis<br><br>*Semi-structured interviews*	Decision making on therapeutic futility	32 Participants<br>13 paediatric oncologists<br>13 parents or primary carers<br>**6 AYACs (age range 13-18 years)**	Four themes were identified<br>1. flow of information to inform decision making<br>2. disclosure of prognosis<br>3. decision maker and stakeholder involved in decision making<br>4. barriers and facilitators to decision making<br>Differing values and agendas. The parents valued messages to "life the spirits" whereas the AYACs values honesty from the healthcare professionals<br>Gatekeeping of information.<br>Theme of "deference to authority"	Medium

For peer review only - http://bmjopen.bmj.com/site/about/guidelines.xhtml

Darabos (2021)	Cancer Related Decision Making Among AYAC, Care Givers and Oncology Providers	USA<br>Single centre	Qualitative – content analysis with hypothesis coding<br><br>*Semi-structured interviews.*	Decision-making and triadic communication	30 Participants:<br>**11 AYACs (age range: 15-24 years)**<br>11 supporters<br>8 healthcare professionals	Four decision-making patterns were identified:<br>1. AYAC driven,<br>2. Collaborative,<br>3. Deferral to parents,<br>4. Provider-based, AYA/Caregiver-approved.<br>Collaborative decision-making and AYAC-driven decisions were most commonly described. There was recognition that some decision-making was day/context dependent.<br>AYACs were more likely to drive decisions regarding supportive care than treatment related decisions.<br>AYACs and caregivers explained how cognitive and emotional processes influenced cancer related decisions.<br>Emotional coping was more common than problem-based coping<br>Direct and honest communication contributes to a stronger relationship.<br>Individuality is key along with flexibility.<br>Time alone is important.	High
Davies (2019)	'Life then', 'life interrupted', 'life reclaimed': the fluctuation of agency in teenagers and young adults with cancer	UK<br>single centre	qualitative - thematic analysis<br><br>*case studies - multiple interviews*	Fluctuation of agency across time and between cases	22 participants<br>**5 AYACs (16-24 years)**<br>**5 parents carers** (2 fathers, 3 mothers, 1 couple),<br>5 healthcare professionals (4 nurses and 1 oncology consultant)<br>5 other supporters (1 boyfriend, 1 girlfriend, 1 aunt, 2 friends)	Agency fluctuates over time within cases and between cases. Agency can fluctuate between personal, proxy and collective perspectives. Personal agency is high prior to diagnosis, decreases after diagnosis and is reclaimed after treatment.	Medium
Ellis (2016)	Fertility concerns among child and adolescent survivors and parents: a qualitative study	Australia<br>single centre	Qualitative<br>semi-structured telephone interviews	Fertility related themes with AYACs who are recently off treatment and with their parents	97 participants from 45 families<br>**19 AYACs (age range 7-17 at diagnosis, mean age 13.3)**<br>44 mothers and 34 fathers	Both parents and AYACs are concerned about the potential impacts on fertility of treatment. Poor doctor-patient communication was reported and conversations about fertility were frequently interrupted to discuss illness and treatment. These fertility discussions were not then continues once the AYAC was off treatment	Medium

For peer review only - http://bmjopen.bmj.com/site/about/guidelines.xhtml

Essig (2016)	Improving Communication in Adolescent Cancer Care: A Multiperspective Study	Germany, Austria, Switzerland<br><br>Number of centres not stated	Qualitative – <u>inductive thematic analysis</u><br><br>*Focus groups.*	Explore effective communication with AYACs for communication skills training.	<u>54 participants:</u><br>**16 AYACs (age range: 13-19 years)**<br>8 parents<br>30 healthcare professionals	Decision-making can cause conflict when adolescents are cognitively mature but legally lack the ability to make decisions.<br>AYACs feel a loss of autonomy.<br>Age-appropriate environments are important.<br>Effective communication differs depending on the type of professional (i.e., doctor vs nurse)<br>Adolescents negatively affect communication when:<br>1. They are indifferent.<br>2. There priorities conflict with treatment<br>3. They conflict with parents.<br>Healthcare professionals negatively affect communication when:<br>1. They don't treat the adolescent in an age-appropriate way.<br>2. They don't take the adolescent seriously.<br>3. They give too much information or withhold important information	Medium
Fern (2013)	The Art of Age-Appropriate Care	UK<br>Number of centres not stated	Qualitative – <u>thematic analysis</u><br><br>*Peer-to-peer interviews, field notes and spider diagrams from focus groups.*	Review a conceptual model of AYACs' cancer care experiences.	<u>11 participants:</u><br>**11 AYACs (age range: 13-25 years)**	Young people must be kept at the centre of interactions in recognition of their stated needs:<br>1. Engagement.<br>2. Individually tailored information.<br>3. Support unproxied by parents/family.<br>AYACs did not want information to be directed at parents but at them.<br>AYACs found it embarrassing when sensitive information was revealed in the presence of their parents.<br>Lack of continuity of healthcare professionals leads to AYACs dissatisfaction and irritation having to repeat their cancer story.	Medium

For peer review only - http://bmjopen.bmj.com/site/about/guidelines.xhtml
For peer review only

Fourneret (2018)	Breaking bad news about cancer to adolescents and young adults: the french experience	France Multicentre	qualitative semi-structured interviews	Explore the effectiveness and implementation of the French announcement protocol in 7 french paediatric oncology centres	<u>90 participants</u><br>**27 AYACs (21 were 14-17 and 6 were 18-22)**<br>30 parents (16 mothers, 5 fathers, 9 parents together at the appointment)<br>33 healthcare professionals	Parents and AYACs have different needs - both of which need to be accounted for when breaking bad news. Awkward and premature announcements were noted<br>The announcement consultation – young patients were never alone when informed of their disease; either with parents (n=31) (parents were informed before their child 10 out of 31 times - this was motivated by a compassionate goal of preparing the parents so they can better support the child when the bad news is broken) or close family member/sibling or boyfriend or girlfriend (n=2).<br>Some parents withheld info and some AYACs preferred parents not to know their diagnosis<br>Asymmetry in the triad discussed - but the key quality needed in the triad is mutual trust<br>HCP found parental presence helpful in the study. HCPs should show empathy (no neutrality) and attention to detail	Medium
Frederick (2018)	Adolescent Patient Involvement in Discussions About Relapsed or Refractory Cancer with Oncology Clinicians.	USA Single centre	Qualitative – <u>content analysis</u><br><br>*Audiotaped conversations.*	Breaking bad news of relapsed or refractory cancer.	<u>75 participants:</u><br>**11 AYACs (age range: 12.6-17.5 years)**<br>44 supporters<br>20 healthcare professionals	Adolescent patients' involvement in conversations about relapsed or refractory cancer is limited.<br>Adolescents were accompanied by one (27%) two (64%) or more than two (18%) family members in the discussion.<br>Adolescents spoke 3.5% of words compared to 66.9% clinicians and 30% parents.<br>No conversations included instances in which the clinicians' asked adolescents for their communication preferences or desired role in decision-making.	High
Friebert (2020)	Congruence gaps between adolescents with cancer and their families regarding values, goals and beliefs about end-of-life care	USA multicentre	*Qualitative cross-sectional study*	End of life care	<u>126 participants</u>:<br>**126 parent-AYAC dyads AYACs (14-20 years, mean age 16.9)**	Young people wanted early information (86%) but only 39% families knew this. Families understanding of what was important to their adolescents when dealing with their own dying was excellent for wanting honest answers from their physician and understanding treatment choices but poor for dying a natural death and being off machines that extend life, if dying.<br>Parents do not know what AYACs want at the end of life	Low

For peer review only - http://bmjopen.bmj.com/site/about/guidelines.xhtml

Glackin (2023)	Experiences of Oncofertility Decision-Making and Care in a National Sample of Adolescent and Young Adult Cancer Patients and Parents	Australasia multicentre	Qualitative – cross sectional survey. Reflexive thematic analysis	Oncofertility decision making	210 participants:<br>**99 AYACs (age range 15-25 years)**<br>111 parents<br>41 AYAC parent dyads from the same family	Four themes were identified: emotional care needs; parent-AYA dynamics including autonomy and agendcy; decision-making considerations including values and practicalities; and reflections on oncofertility and follow-up.<br>Both AYAC and parents placed importanceon AYA autonomy in fertility decision-making but, but many AYAs appreciated the role of parents in providing support and guidance throughout the process.<br>Healthcare professionals are encouraged to autonomously engage AYA's around fertility decision making, while concurrently offering opportunities that promotes parental support.	Low
Hart (2020)	The Challenges of Making Informed Decisions About Treatment and Trial Participation Following Cancer: A Qualitative Study with Adolescent and Young Adults with Cancer and Care Givers	UK Multicentre	Qualitative – thematic analysis<br><br>*Semi-structured interviews.*	Shared decision-making – primary treatment and trial participation – at diagnosis.	33 participants:<br>**18 AYACs (age range: 16-24 years)**<br>15 supporters	AYACs struggled to process information around diagnosis, exacerbated by symptom burden, emotions, and the fast pace of clinical activity.<br>Some AYACs disengaged from conversation topics which were distressing.<br>There are limited options for 'real' decision-making at diagnosis. However, many preferred this when they were already overwhelmed by emotions/symptoms.<br>For trial enrollment, many AYACs allowed themselves to be steered by the recommendation of the healthcare professional who recruited them, thinking they were acting in their best interests.	Medium
Hong (2016)	Care Partnerships: toward technology to support teen's participation in their health care	US multicentre	Qualitative semi-structured interviews and observations	To investigate how technology can support the partnerships between AYACs, parents and clinicians when the AYAC is experiencing complex chronic illness	33 interviews.<br>**15 with AYACs (13 of whom had cancer. age range 13-17)**<br>15 parents (10 mothers, 1 fathers, 1 aunt and 2 fathers and mothers together)<br>8 clinician caregivers	Participants faced challenges concerning:<br>1) Teens' limited participation in their care<br>2) communicating emotionally sensitive information<br>3) managing physical and emotional responses<br>Time alone with clinicians was important. Mutual protectionism or the need to "emotionally protect eachother" was prevalent.	Medium

For peer review only - http://bmjopen.bmj.com/site/about/guidelines.xhtml

Ingersgaard (2018)	A qualitative study on decision-making on Phase III randomized clinical trial participation in paediatric oncology: adolescents' and parents' perspectives and preferences	Denmark	qualitative exploratory study - in-depth semi-structured interviews with thematic analysis	To explore patients' and AYACs' motivs for accepting/ declining participation in the AL2008 trial and adolescents' involvement in decision making	16 participants<br>**5 AYACs (age range 12-16)**<br>6 parents of AYACs<br>5 parents of children aged 3-10 years with cancer	Key themes<br>1) altruism - wanting to help future AYACs<br>2) trust in the clinicians<br>3) individuals perceptions of cure contra toxicity<br>4) adolescents as active participants in the decision making process<br>5) parental responsibility and authority<br>6) the difficulty of uncertainty	Low
Jacobs (2015)	Adolescent end of life preferences and congruence with their parents' preferences: results of a survey of adolescents with cancer	Norway	Qualitative<br><br>three sessions of dyadic interviews	To explore AYACs' end of life preferences and to assess the congruence of these preferences with the parents' beliefs	17 adolescent/ family dyads<br>**17 AYACs (age range 14-21, 71% under 18)**	Adolescents with cancer were comfortable discussing EOL, and the majority preferred to talk about EOL issues before they are facing EOL. There were substantive areas of agreement between adolescents and their surrogates, but important facets of adolescents' EOL wishes were not known by their families, reinforcing the importance of eliciting individual preferences and engaging dyads so parents can understand their children's wishes. 53% of AYACs had never spoken about their end of life preferences but 82% considered it important to let their loved ones know their wishes.	Low

For peer review only

Korsvold (2017)	A content analysis of emotional concerns expressed at the time of receiving a cancer diagnosis: An observational study of consultations with adolescent and young adult patients and their family members	Norway	exploratory mixed methods study<br><br>audio recorded consultations	To investigate the emotional concerns of AYACs at the time of diagnosis and how to quantify how healthcare professionals respond	18 participants<br>**9 AYACs (age range 13-23)**<br>Present with mother (n=9), father (n=1), sister (n=1) or mother and father (n=2)	Four major themes of emotional concerns expressed by AYA patients and their family members during consultations for a cancer diagnosis:<br>1) side effects/late effects or infertility,<br>2) "what happens in the near future/practical aspects",<br>3) fear<br>4) sadness<br>AYA patients and family members expressed emotional concerns. HCPs typically responded by providing information, rather than affective aspects of the concerns In the sadness theme however, an explicit provide space affective response was the most common response (n=8) followed by an explicit provide space content response (n=7)<br>To make patients 'feel known' HCP should pay attention to the affective aspect of the expressed concern.	Medium
Lyon (2013)	Family-Centered Advance Care Planning for Teens With Cancer	USA<br>single centre	Qualitative<br>a randomised control pilot study	To examine the efficacy of family-centres ACP	30 dyads<br>**mean age of AYACs 16**<br>17 were randomised to intervention and 13 were randomised to control<br>87% of surrogates were biological parents and were female	The model (ACP) increased congruence in the triad compared to the control standard of care group– so it is key. The family centres ACP AYACs reported feeling more informed that the control group	Low

For peer review only - http://bmjopen.bmj.com/site/about/guidelines.xhtml

Mack (2019)	Adolescent and Young Adult Cancer Patients' Experiences with Treatment Decision-Making	USA Single Centre	Quantitative – multivariate analysis, logistic regression<br><br>*Surveys at diagnosis, 4 and 12 months.*	Treatment decision-making	203 participants:<br>**203 AYACs (age range: 15-29 years)**	A majority of AYACs (58%) want to share decision-making with oncologists. The remainder were split between the AYAC wanting primary responsibility in decision-making (20%) or wanting their oncologist to have primary responsibility (22%).<br>A lower proportion of younger AYACs wanted sole responsibility but this did not achieve statistical significance (P = 0.07).<br>The majority (90%) of AYACs who lived with a parent/guardian wanted some form of input from their parents (either collaborative or considering their opinion).<br>Younger AYACs (15-17 years) were more likely to want greater involvement by their parents but were also more likely to be less involved than they wanted to be relative to their parents.<br>Decisional regret was less likely among AYACs who trusted oncologists completely, and who reported that oncologists understood what was important to them when treatment started.	Medium
Mobley (2023)	Clinical Trial Participation: A qualitative study of Adolescents and Younger Adults Recently Diagnosed with Cancer	USA	Qualitative<br>Grounded theory analysis of semi-structured interviews	Clinical trial participation	9 participants:<br>**9 AYACs (age range 16-20)**	Consent encompassed the first discussion of CCT. Patients reflected positive and negative effects of timing, decisional role, and emotional impact.<br>Informing participation involved decision-making processes, specific knowledge, understanding and external influence.<br>Participant relationships emphasized the importance of communication and relationships with providers and parents. Patient determinants centered on motives from different perspectives, pre-conceived attitudes, and understanding of CCTs.	Medium

For peer review only - http://bmjopen.bmj.com/site/about/guidelines.xhtml

Olsavsky (2021)	Family communication about fertility preservation in adolescent males newly diagnosed with cancer	USA	Qualitative	To explore fertility preservation communication among mothers, fathers and their male adolescents newly diagnosed with cancer.	<u>87 participants:</u><br>**33 AYAC aged 12-25**<br>32 mothers<br>22 fathers<br><br>Representing 37 families in total.	Five process themes:<br>(1) Reliance on health care team and social support networks to facilitate FP decisions (noted just by parents),<br>(2) withholding parental opinion and deferring the decision to the adolescent,<br>(3) ease of communication,<br>(4) communication barriers and facilitators,<br>(5) not being present or not remembering details of FP conversations.<br>Four content themes:<br>(1) preference for biological parenthood (or grandparenthood),<br>(2) consideration of future partner of AYAC's desire for biological parenthood,<br>(3) sperm banking whilst it is a viable option,<br>(4) openness to alternative parenthood options	Medium

For peer review only - http://bmjopen.bmj.com/site/about/guidelines.xhtml

Patterson (2012)	The Unmet Needs of Emerging Adults With a Cancer Diagnosis	Australasia	Qualitative	Aim to contribute to the limited research base and inform our understanding of the needs of emerging adults with a diagnosis of cancer from a developmental perspective that appreciates the key transitional tasks of emerging adulthood identified by Arnett	14 Participants:<br>**14 AYAC aged 20-25**, average age of 22	A cancer experience poses the potential for significant impact on the four requirements for achievement of adulthood.<br><br>The needs of these emerging adults were grouped into six themes; information, healthcare provision, daily living, interpersonal support, identity renegotiation and emotional distress.<br><br>These themes relate directly to the four requirements of adulthood.<br>1. The task of accepting responsibility for oneself emphasises the importance of empowering AYAC in their communication with HCP.<br>2. The task of deciding on personal beliefs and values highlights the importance of keeping the AYAC informed and encouraging them in decisions giving maximum opportunity to explore beliefs.<br>3. Establishing a relationship with parents as equals highlights the importance of maximising AYAC autonomy in relation to supporters.<br>4. The task of becoming financially independent highlights the importance of minimising disruption to daily life.	Low

For peer review only - http://bmjopen.bmj.com/site/about/guidelines.xhtml

Pennant (2020)	The Role of Social Support in Adolescent/Young Adults Coping with Cancer Treatment	USA	Qualitative	To explore specific actions that help AYAC and what behaviours they want from their social supports	<u>20 Participants:</u><br>**10 AYAC ages 15-26**, mean age 18.9 years<br>10 parents	Themes of support included; presence, distraction, positive attitude, maintaining AYAC autonomy, communication and advocacy.<br>Mothers were the most noted family support.<br>AYAC patients can differ in their preferences throughout treatment and this can, at times, appear contradictory.<br>AYACs appear to want autonomy and independence, but appreciate help with daily tasks from their parents.<br>They express the desire for privacy, but also value physical presence and communication<br>Parents must oscillate between being involved in and catering to their AYAC child's needs during treatment while allowing space for independence and autonomy.<br>The findings underscore the importance of maintaining open communication with AYAC patients about their preferences and needs throughout the course of treatment and asking them about both individual and social preferences, which may change frequently.	Medium
Pyke-Grimm (2020)	3 Dimensions of Treatment Decision Making in Adolescents and Young Adults with Cancer.	USA Multicentre	Qualitative – <u>ethnographic</u><br><br>*Semi-structured interviews, field notes.*	Explore the preferences of AYACs for involvement in healthcare decisions	<u>16 participants:</u><br>**16 AYACs (age range: 14.7-20 years)**	Emotions around diagnosis inhibit information receptiveness and ability to engage in treatment decisions (especially important decisions).<br>Initially AYACs struggle with the jargon and plethora of medical terms which are being used. They have limited knowledge which limits their questions, this increases over time.<br>The importance of decisions differs from one AYACs to the next. Also, some decisions are seen as having only one 'real' option.<br>AYACs engaged in minor decisions much earlier in their treatment, and some began engaging in more important decisions later in treatment.<br>AYACs could adopt an active (sole decision maker), collaborative (with healthcare professionals/supporters) or passive (healthcare professionals/supporters as decision makers) role.	Medium

For peer review only - http://bmjopen.bmj.com/site/about/guidelines.xhtml

Pyke-Grimm (2022)	Day-to-Day Decision Making by Adolescents and Young Adults with Cancer	USA	Qualitative Interpretive focused ethnography within the socio-logic tradition, informed by symbolic interactionism	To explore involvement of AYAs with cancer in day-to-day decisions affected by their cancer and treatment.	16 Participants:<br>**16 AYAC aged 15-20 (at time of interview - with an average of one year from diagnosis)**	Factors influence the involvement of AYAC in decision making such as the type of decision, the point in the cancer journey. They want to be involved.<br>Four day to day decision making categories were identified: mental mindset, self care practices, self-advocacy and negotiating relationships.<br>Parents were often present and staying strong was a recurring theme across mental mindset and negotiating relationships.<br>HCP are critical to facilitate AYAC participation in day to day decision making by encouraging autonomy and with effective communication.	Medium
Sawyer (2019)	Developmentally Appropriate Care for Adolescents and Young Adults with Cancer: How Well is Australia Doing?	Australasia Multicentre	Quantitative – Chi-squared and Fisher's exact test<br><br>*Single time point survey.*	Explore quality of AYAC care in Australia.	196 participants:<br>**196 AYACs (age range: 15-25 years)**	>90% of AYACs reported positive responses for 11 of the 14 experience of care items which related to the quality of communication and general interactions with the cancer care team.<br>The most highly endorsed of these experiences of care items related to staff being friendly and respectful, communicating in ways that the AYAC understood, being supportive of AYACs asking questions and engaging families in discussion and decisions as the AYAC wished.<br>Older AYAC (20-25 years) report more empowerment to make decisions than younger AYACs and were more likely to report that healthcare professionals included their family in discussions and decision-making the way they wanted them to be included.	Low

For peer review only

Sisk (2022)	Interdependent Functions of Communication with Adolescents and Young Adults in Oncology	USA Multicentre	Qualitative – content analysis *Semi-structured interviews.*	Define communication functions from perspective of AYACs.	37 participants: **37 AYACs (age range: 12-20 years; mean: 16 years)**	Building relationships: demonstrating clinical competence, reliability, empathy, and showing care and concern. When clinicians demonstrated these attributes, AYACs described feelings of trust in the clinicians' ability and intent to care for them.<br>Exchanging information: providing accurate and transparent information that was adapted to AYACs' needs. These needs related to the amount, complexity, timing, and pacing of information, and balancing communication between parents and AYACs.<br>AYACs think honesty and transparency is important. However, transparency could be burdensome.<br>Exploring uncertainties and fears of the future mad AYACs feel better prepared and decreasing anxiety. There was variation between AYACs for exploring these unknowns.<br>AYACs varied in their preferences in sharing distressing information and whether healthcare professionals should remain present and or give AYACs their privacy.<br>AYACs often feel that treatment related decisions realistically only have one choice giving a sense of powerlessness. They played a greater role in decisions outside of treatment related areas.<br>While some AYACs preferred very passive or active roles most described an interdependent process of communication involving them, their parents, and their clinicians.<br>Parents often served as a conduit and buffer of communication between the AYAC and healthcare professional. Many described the integral role of parents in communication regardless of their age.	High

For peer review only - http://bmjopen.bmj.com/site/about/guidelines.xhtml

Sisk (2022)	Co-management of communication and care in adolescent and young adult oncology	USA	Qualitative Semi-structured interviews	Study aimed to learn how AYAs and parents shared and delegated roles in communication and care during and after treatment for cancer	<u>37 Participants:</u><br>**37 AYAC aged 12-24**<br>**Mean age 16 years**	There are 6 roles that AYAC co-manage with parents; managing information, managing social and emotional needs, managing health, advocacy and empowerment, making decisions and managing logistics.<br>Five factors that influence AYAC roles in communication were:<br>AYAC agency<br>Clinical encouragement<br>Emotional and physical well-being<br>Personality, preferences and values<br>Insights and skills<br><br>There are multiple benefits of engagement of the adolescent.	Medium
Son (2023)	Family Communication About Cancer in Korea: A Dyadic Analysis of Parent-Adolescent Conversation	Korea	Qualitative descriptive study	The aim was to understand communication experiences of Korean AYAC and their parents in the context of young adult cancer.	<u>14 participants:</u><br>**7 AYAC (ages 14-19 years)** and 7 parent pairs	The main theme was "experience the same thing but see it differently" along with three subthemes.<br>Different expectations for parent-adolescent communication, different views on communication challenges and limited sharing and progress in the conversation.<br>This study offers insights into different communication expectations and preferences between Korean adolescents and parents, and reasons for communication challenges, while emphasizing the individualized assessment of parent-adolescent communication between them.	Medium

For peer review only - http://bmjopen.bmj.com/site/about/guidelines.xhtml

Viola (2018)	Problem-solving skills, parent–adolescent communication, dyadic functioning, and distress among adolescents with cancer	USA	Mixed methods study	The aim was to describe and assess how intrapersonal (i.e., problem-solving ability) and social–ecological factors (i.e., cancer- related communication with parents and parent–adolescent dyadic relationship quality) are associated with adolescent adjustment (i.e., distress).	78 participants:<br>39 AYAC and 39 parent pairs<br>**AYAC 14-20 mean age 16.1**<br>39 Parents - 79.5 % mothers	Better adolescent problem solving skills and better parent problem solving skills were associated with lower adolescent distress.<br>Parents and adolescents reported similar moderate levels of cancer related communication problems.<br>The most commonly endorsed cancer-related problem was "not talking about what to do if the AYAC got significantly worse'.<br>Parents reported better problem solving ability and better dyadic functioning than their adolescent.	Low
Weaver (2016)	"Being a Good Patient" During Times of Illness as Defined by Adolescent Patients With Cancer	USA	Qualitative - semantic content analysis<br><br>Semi-structured interviews		40 participants<br>**AYAC ages 12-19**<br>Mean age of 15.5 years	The concepts of adherence and compliance were the primary phrases used to describe the good patient role, but always within the context of a relationship. Of note: A total of 23 adolescents requested to be interviewed alone with the interviewer (57.5%)	Medium
Weaver (2015)	Adolescents' Preferences for Treatment Decisional Involvement During Their Cancer	USA Multicentre	Qualitative – semantic content analysis<br><br>*Semi-structured interviews.*	Investigate AYACs' decision-making preferences and how supports and healthcare professionals can support involvement.	40 participants:<br>**40 AYACs (age range at interview: 12-18.9 years; 0.5-6 months from diagnosis/relapse)**<br><br>*NB: 34 AYACs primary diagnosis, 6 AYACs relapse.*	AYACs indicated a spectrum of preferred decisional roles, with the most common being an actively involved role (65%), although a shared decision-making approach was still valued.<br>AYACs recognized that situational and social contexts might shift their preferred level of involvement in medical decisions.<br>Although adolescents wanted to be involved in decisions, they also expressed an appreciation of family insight, parental presence, and clinician guidance.<br>AYACs can retrospectively identify their preferences for inclusion in medical decision-making, and even when preferring involvement, they value the input of trusted others.	Medium

For peer review only - http://bmjopen.bmj.com/site/about/guidelines.xhtml

Wu (2021)	Decisional conflicts, anxiety, and perceptions of shared decision-making in cancer treatment trajectory among adolescents with cancer: A longitudinal study	Taiwan	Qualitative. An explanatory mixed method was used, incorporating questionnaires and individual interviews.	To describe the perception on levels of decision-making during cancer treatment for adolescents with cancer and examine the trajectory of their decisional conflict	44 participants:<br>**22 AYAC** 11 male and 11 female<br>mean age 15.39<br>**22 Supporters**:<br>father n=1<br>mothers n=12<br>both n=6<br>other n=3	Different levels of participation in shared decision making (SDM) during the treatment trajectory were found.<br>Participants experienced the highest decisional conflict during diagnosis.<br>Roles in healthcare communication varied from direct participation to indirect involvement.<br>Overall, participants reported that doctors and parents decided their level of involvement, communication and or decision making.	Medium
Zarnegar et al (2018)	Recall of Fertility Discussion by Adolescent Female Cancer Patients: A Survey-Based Pilot Study	USA	Qualitative	To assess: recall of a fertility discussion, satisfaction with fertility knowledge, and identify factors that may influence recall.	19 participants:<br>**19 AYAC aged** 13-18 years and a mean age of 15.6	42% and 52% of AYAC did not recall discussion regarding treatment related infertility or fertility preservation during initial treatment planning.<br>63% of AYAC reported that parents made all or most of the decisions whereas 30.8% reported making decisions with parents.<br>Key Finding - A greater percentage of AYAC who reported making a joint decision with parents recalled 71% of fertility discussions than those who reported parents made most or all of medical decisions.	Medium

[¥]Age range at diagnosis is given except where this was not provided in the article in which alternative metrics are presented and this is highlighted.
AYA(C) = adolescent and young adult (with cancer); NOS = not otherwise specified; RM-ANOVA = repeated measures analysis of variance.

For peer review only - http://bmjopen.bmj.com/site/about/guidelines.xhtml

Update – December 2023

Medline

Ovid MEDLINE(R) and Epub Ahead of Print, In-Process, In-Data-Review & Other Non-Indexed Citations, Daily and Versions <1946 to December 19, 2023>

1 (Parent* or guardian* or mother* or father* or partner or wife or wives or husband* or boyfriend* or girlfriend* or sibling* or friend* or carer* or "third person" or caregiver* or "care-giver*" or spouse* or supporter* or support network*).ti,ab. or parents/ or fathers/ or mothers/ or spouses/ or caregivers/ or siblings/ or friends/ or legal guardians/ 1148168

2 (TYA cancer or TYA oncology or AYA cancer or AYA oncology or (young adult adj3 (cancer or oncology or leuk?em* or lymphom* or h?ematol*)) or ("teenage and young adult" adj3 cancer) or ("teenage and young adult" adj3 oncology) or (teenage* adj3 cancer) or (teenage* adj3 oncology) or (adolescen* adj3 cancer) or (adolescen* adj3 oncology) or (young people adj3 cancer) or (young people adj3 oncology) or ("teenage and young adult" adj3 leuk?emia*) or (teenage* adj3 leuk?emia*) or (adolescen* adj3 leuk?emia*) or (young people adj3 leuk?emia*) or (young adult adj3 leuk?emia*) or ("teenage and young adult" adj3 h?ematol*) or (teenage* adj3 h?ematol*) or (adolescen* adj3 h?ematol*) or (young people adj3 h?ematol*) or (young adult adj3 h?ematol*) or ("teenage and young adult" adj3 lymphom*) or (teenage* adj3 lymphom*) or (adolescen* adj3 lymphom*) or (young people adj3 lymphom*) or (young adult adj3 lymphom*)).ti,ab. or ((exp adolescent/ or exp young adult/) and exp neoplasms/) 337284

3 (Communicat* or Disclos* or inform* or Interact* or relationship* or Conversation* or Dialogue* or triad* or Interview* or consult* or decision making).ti,ab. or exp communication/ or exp disclosure/ or exp information dissemination/ or exp physician-patient relations/ 6147070

4 (affect* or effect* or influenc* or resultant or impact* or perception* or perspective* or encounter* or preference or opinion or involvement or occurance* or feel or "go through" or experienc*).ti,ab. 13278879

5 1 and 2 and 3 and 4 3519

6 limit 5 to english language 3399

7 (202211* or 202212* or 2023* or 2024*).dp. or (202211* or 202212* or 2023* or 2024*).ez. or (202211* or 202212* or 2023* or 2024*).ed. or (202211* or 202212* or 2023* or 2024*).ep. 1958643

8 6 and 7 163

Embase

Embase <1974 to 2023 December 19>

1 (TYA cancer or TYA oncology or AYA cancer or AYA oncology or (young adult adj3 (cancer or oncology or leuk?em* or lymphom* or h?ematol*)) or ("teenage and young adult" adj3 cancer) or ("teenage and young adult" adj3 oncology) or (teenage* adj3 cancer) or (teenage* adj3 oncology) or (adolescen* adj3 cancer) or (adolescen* adj3 oncology) or (young people adj3 cancer) or (young people adj3 oncology) or ("teenage and young adult" adj3 leuk?emia*) or (teenage* adj3 leuk?emia*) or (adolescen* adj3 leuk?emia*) or (young people adj3 leuk?emia*) or (young adult adj3 leuk?emia*) or ("teenage and young adult" adj3 h?ematol*) or (teenage* adj3 h?ematol*) or (adolescen* adj3 h?ematol*) or (young people adj3 h?ematol*) or (young adult adj3 h?ematol*) or ("teenage and young adult" adj3 lymphom*) or (teenage* adj3 lymphom*) or (adolescen* adj3 lymphom*) or (young people adj3 lymphom*) or (young adult adj3 lymphom*)).ti,ab. or ((exp *adolescent/ or exp *young adult/) and exp *neoplasm/) 10359

2 (Communicat* or Disclos* or inform* or Interact* or relationship* or Conversation* or Dialogue* or triad* or Interview* or consult* or decision making).ti,ab. or exp *interpersonal communication/ or exp *professional-patient relationship/ or exp *information dissemination/ or exp *conversation/ 7565603

3 (Parent* or guardian* or mother* or father* or partner or wife or wives or husband* or boyfriend* or girlfriend* or sibling* or friend* or carer* or "third person" or caregiver* or "care-giver*" or spouse* or supporter* or support network*).ti,ab. or *parent/ or *father/ or *mother/ or *spouse/ or *caregiver/ or *social worker/ or *sibling/ or *friend/ or *legal guardian/ 1440315

4 (affect* or effect* or influenc* or resultant or impact* or perception* or perspective* or encounter* or preference or opinion or involvement or occurance* or feel or "go through" or experienc*).ti,ab. 16593794

5 1 and 2 and 3 and 4 1032

6 limit 5 to english language 1010

7 (202211* or 202212* or 2023* or 2024*).dc. or (202211* or 202212* or 2023* or 2024*).dd. or (202211* or 202212* or 2023* or 2024*).dp. 2647560

8 6 and 7 107

PsycINFO (via Ebsco)

#	Query	Limiters/Expanders	Last Run Via	Results
S11	S1 AND S2 AND S7 AND S8	Limiters - Publication Date: 20221101-20241231 Expanders - Apply equivalent subjects Search modes - Boolean/Phrase	Interface - EBSCOhost Research Databases Search Screen - Basic Search Database - APA PsycInfo	238
S10	S1 AND S2 AND S7 AND S8	Expanders - Apply equivalent subjects Narrow by	Interface - EBSCOhost Research	6,315

For peer review only

		Language: - english Search modes - Boolean/Phrase	Databases Search Screen - Basic Search Database - APA PsycInfo	
S9	S1 AND S2 AND S7 AND S8	Expanders - Apply equivalent subjects Search modes - Boolean/Phrase	Interface - EBSCOhost Research Databases Search Screen - Basic Search Database - APA PsycInfo	6,549
S8	(affect* or effect* or influenc* or resultant or impact* or perception* or perspective* or encounter* or preference or opinion or involvement or occurance* or feel or "go through" or experienc*)	Expanders - Apply equivalent subjects Search modes - Boolean/Phrase	Interface - EBSCOhost Research Databases Search Screen - Basic Search Database - APA PsycInfo	3,524,144
S7	(S5) or (S3)	Expanders - Apply equivalent subjects Search modes - Boolean/Phrase	Interface - EBSCOhost Research Databases Search Screen - Basic Search Database - APA PsycInfo	62,557
S6	S4 AND S5	Expanders - Apply equivalent subjects Search modes - Boolean/Phrase	Interface - EBSCOhost Research Databases Search Screen - Basic Search Database - APA PsycInfo	62,384

For peer review only

S5	((DE “neoplasms” OR DE "Benign Neoplasms" OR DE "Breast Neoplasms" OR DE "Endocrine Neoplasms" OR DE "Leukemias" OR DE "Melanoma" OR DE "Metastasis" OR DE "Nervous System Neoplasms" OR DE "Terminal Cancer"))	Expanders - Apply equivalent subjects Search modes - Boolean/Phrase	Interface - EBSCOhost Research Databases Search Screen - Basic Search Database - APA PsycInfo	62,384
S4	((DE “neoplasms” OR DE "Benign Neoplasms" OR DE "Breast Neoplasms" OR DE "Endocrine Neoplasms" OR DE "Leukemias" OR DE "Melanoma" OR DE "Metastasis" OR DE "Nervous System Neoplasms" OR DE "Terminal Cancer"))	Expanders - Apply equivalent subjects Search modes - Boolean/Phrase	Interface - EBSCOhost Research Databases Search Screen - Basic Search Database - APA PsycInfo	62,384
S3	("TYA cancer" or "TYA oncology" or "AYA cancer" or "AYA oncology" or ("young adult" n3 (cancer or oncology or leuk?em* or lymphom* or h?ematol*)) or ("teenage and young adult" n3 cancer) or ("teenage and young adult" n3 oncology) or (teenage* n3 cancer) or (teenage* n3 oncology) or (adolescen* n3 cancer) or (adolescen* n3 oncology) or ("young people" n3 cancer) or ("young people" n3 oncology) or ("teenage and young adult" n3 leuk?emia*) or (teenage* n3 leuk?emia*) or (adolescen* n3 leuk?emia*) or ("young people" n3 leuk?emia*) or ("young adult" n3 leuk?emia*) or ("teenage and young adult" n3 h?ematol*) or (teenage* n3 h?ematol*) or (adolescen* n3 h?ematol*) or ("young people" n3 h?ematol*) or ("young adult" n3 h?ematol*) or ("teenage and young adult" n3 lymphom*) or (teenage* n3 lymphom*) or (adolescen* n3 lymphom*) or ("young people" n3 lymphom*) or ("young adult" n3 lymphom*))	Expanders - Apply equivalent subjects Search modes - Boolean/Phrase	Interface - EBSCOhost Research Databases Search Screen - Basic Search Database - APA PsycInfo	2,072
S2	(Communicat* or Disclos* or inform* or Interact* or relationship* or Conversation* or Dialogue* or triad* or Interview* or consult* or "decision making") or DE “communication” OR DE “information dissemination” OR DE “conversation”	Expanders - Apply equivalent subjects Search modes - Boolean/Phrase	Interface - EBSCOhost Research Databases Search Screen - Basic Search Database - APA PsycInfo	2,545,968

For peer review only

S1	(Parent* or guardian* or mother* or father* or partner or wife or wives or husband* or boyfriend* or girlfriend* or sibling* or friend* or teacher* or social worker* or carer* or "third person" or caregiver* or "care-giver*" or spouse* or chaperone*) OR DE “parents” OR DE “mothers” OR DE “fathers” OR DE “spouses” OR DE “wives” OR DE “husbands” OR DE “siblings” OR DE “significant others” OR DE “social workers” OR DE “guardianship” OR DE “caregivers”	Expanders - Apply equivalent subjects Search modes - Boolean/Phrase	Interface - EBSCOhost Research Databases Search Screen - Basic Search Database - APA PsycInfo	937,154

CINAHL (via Ebsco)

Wednesday, December 20, 2023 4:07:56 PM

#	Query	Limiters/Expanders	Last Run Via	Results
S7	S1 AND S2 AND S3 AND S4	Limiters - Publication Date: 20221101-20241231 Expanders - Apply equivalent subjects Search modes - Boolean/Phrase	Interface - EBSCOhost Research Databases Search Screen - Advanced Search Database - CINAHL	99
S6	S1 AND S2 AND S3 AND S4	Expanders - Apply equivalent subjects Narrow by Language: - english Search modes - Boolean/Phrase	Interface - EBSCOhost Research Databases Search Screen - Advanced Search Database - CINAHL	2,152
S5	S1 AND S2 AND S3 AND S4	Expanders - Apply equivalent subjects Search modes - Boolean/Phrase	Interface - EBSCOhost Research Databases Search Screen - Advanced Search Database - CINAHL	2,189

For peer review only

S4	(affect* or effect* or influenc* or resultant or impact* or perception* or perspective* or encounter* or preference or opinion or involvement or occurance* or feel or "go through" or experienc*)	Expanders - Apply equivalent subjects Search modes - Boolean/Phrase	Interface - EBSCOhost Research Databases Search Screen - Advanced Search Database - CINAHL	3,149,468
S3	("TYA cancer" or "TYA oncology" or "AYA cancer" or "AYA oncology" or ("young adult" n3 (cancer or oncology or leuk?em* or lymphom* or h?ematol*)) or ("teenage and young adult" n3 cancer) or ("teenage and young adult" n3 oncology) or (teenage* n3 cancer) or (teenage* n3 oncology) or (adolescen* n3 cancer) or (adolescen* n3 oncology) or ("young people" n3 cancer) or ("young people" n3 oncology) or ("teenage and young adult" n3 leuk?emia*) or (teenage* n3 leuk?emia*) or (adolescen* n3 leuk?emia*) or ("young people" n3 leuk?emia*) or ("young adult" n3 leuk?emia*) or ("teenage and young adult" n3 h?ematol*) or (teenage* n3 h?ematol*) or (adolescen* n3 h?ematol*) or ("young people" n3 h?ematol*) or ("young adult" n3 h?ematol*) or ("teenage and young adult" n3 lymphom*) or (teenage* n3 lymphom*) or (adolescen* n3 lymphom*) or ("young people" n3 lymphom*) or ("young adult" n3 lymphom*)) OR ((MH "adolescence+" OR MH "young adult+") AND (MH "neoplasms+"))	Expanders - Apply equivalent subjects Search modes - Boolean/Phrase	Interface - EBSCOhost Research Databases Search Screen - Advanced Search Database - CINAHL	61,263
S2	(Communicat* or Disclos* or inform* or Interact* or relationship* or Conversation* or Dialogue* or triad* or Interview* or consult* or "decision making") or MH "communication+" OR MH "discussion" OR MH "conversation"	Expanders - Apply equivalent subjects Search modes - Boolean/Phrase	Interface - EBSCOhost Research Databases Search Screen - Advanced	2,080,061

	OR (MH "Professional-Patient Relations+")		Search Database - CINAHL	
S1	(Parent* or guardian* or mother* or father* or partner or wife or wives or husband* or boyfriend* or girlfriend* or sibling* or friend* or teacher* or social worker* or carer* or "third person" or caregiver* or "care-giver*" or spouse* or chaperone*) OR MH “parents” OR MH “mothers” OR MH “fathers” OR MH “spouses” OR MH “siblings” OR MH “teachers” OR MH “social workers” OR MH “caregivers”	Expanders - Apply equivalent subjects Search modes - Boolean/Phrase	Interface - EBSCOhost Research Databases Search Screen - Advanced Search Database - CINAHL	567,768

Web of Science Core Collection

Web of Science Search Strategy (v0.1)

Database: Web of Science Core Collection

Entitlements:

- WOS.IC: 1993 to 2023
- WOS.CCR: 1985 to 2023
- WOS.SCI: 1900 to 2023
- WOS.AHCI: 1975 to 2023
- WOS.BHCI: 2008 to 2023
- WOS.BSCI: 2008 to 2023
- WOS.ESCI: 2018 to 2023
- WOS.ISTP: 1990 to 2023
- WOS.SSCI: 1956 to 2023
- WOS.ISSHP: 1990 to 2023

Searches:

1: TS=(Parent* or guardian* or mother* or father* or partner or wife or wives or husband* or boyfriend* or girlfriend* or sibling* or friend* or teacher* or social worker* or carer* or "third person" or caregiver* or "care-giver*" or spouse* or chaperone*)

Date Run: Wed Dec 20 2023 16:03:59 GMT+0000 (Greenwich Mean Time)

Results: 2283955

2: TS=("TYA cancer" or "TYA oncology" or "AYA cancer" or "AYA oncology" or ("young adult" near/3 (cancer or oncology or leuk?em* or lymphom* or h?ematol*)) or ("teenage and young adult" near/3 cancer) or ("teenage and young adult" near/3 oncology) or (teenage*

For peer review only

near/3 cancer) or (teenage* near/3 oncology) or (adolescen* near/3 cancer) or (adolescen* near/3 oncology) or ("young people" near/3 cancer) or ("young people" near/3 oncology) or ("teenage and young adult" near/3 leuk?emia*) or (teenage* near/3 leuk?emia*) or (adolescen* near/3 leuk?emia*) or ("young people" near/3 leuk?emia*) or ("young adult" near/3 leuk?emia*) or ("teenage and young adult" near/3 h?ematol*) or (teenage* near/3 h?ematol*) or (adolescen* near/3 h?ematol*) or ("young people" near/3 h?ematol*) or ("young adult" near/3 h?ematol*) or ("teenage and young adult" near/3 lymphom*) or (teenage* near/3 lymphom*) or (adolescen* near/3 lymphom*) or ("young people" near/3 lymphom*) or ("young adult" near/3 lymphom*)) Date Run: Wed Dec 20 2023 16:04:06 GMT+0000 (Greenwich Mean Time) Results: 8540

3: TS=(Communicat* or Disclos* or inform* or Interact* or relationship* or Conversation* or Dialogue* or triad* or Interview* or consult* or "decision making")
Date Run: Wed Dec 20 2023 16:04:13 GMT+0000 (Greenwich Mean Time)
Results: 12748181

4: TS= (affect* or effect* or influenc* or resultant or impact* or perception* or perspective* or encounter* or preference or opinion or involvement or occurance* or feel or "go through" or experienc*) Date Run: Wed Dec 20 2023 16:04:19 GMT+0000 (Greenwich Mean Time) Results: 26006930

5: #4 AND #3 AND #2 AND #1 Date Run: Wed Dec 20 2023 16:04:26 GMT+0000 (Greenwich Mean Time) Results: 764

6: #4 AND #3 AND #2 AND #1 and English (Languages) Date Run: Wed Dec 20 2023 16:04:35 GMT+0000 (Greenwich Mean Time) Results: 737

7: #4 AND #3 AND #2 AND #1 and English (Languages) and 2022 or 2023 (Publication Years) Date Run: Wed Dec 20 2023 16:04:39 GMT+0000 (Greenwich Mean Time) Results: 132

AMED via Ovid

AMED (Allied and Complementary Medicine) <1985 to October 2023>

1 (Parent* or guardian* or mother* or father* or partner or wife or wives or husband* or boyfriend* or girlfriend* or sibling* or friend* or carer* or "third person" or caregiver* or "care-giver*" or spouse* or supporter* or support network*).ti,ab. 14291
2 (TYA cancer or TYA oncology or AYA cancer or AYA oncology or (young adult adj3 (cancer or oncology or leuk?em* or lymphom* or h?ematol*)) or ("teenage and young adult" adj3 cancer) or ("teenage and young adult" adj3 oncology) or (teenage* adj3 cancer) or (teenage* adj3 oncology) or (adolescen* adj3 cancer) or (adolescen* adj3 oncology) or (young people adj3 cancer) or (young people adj3 oncology) or ("teenage and young adult" adj3 leuk?emia*) or (teenage* adj3 leuk?emia*) or (adolescen* adj3 leuk?emia*) or (young people adj3 leuk?emia*) or (young adult adj3 leuk?emia*) or ("teenage and young adult" adj3 h?ematol*) or (teenage* adj3 h?ematol*) or (adolescen* adj3 h?ematol*) or (young people adj3 h?ematol*) or (young adult adj3 h?ematol*) or ("teenage and young adult" adj3

For peer review only

lymphom*) or (teenage* adj3 lymphom*) or (adolescen* adj3 lymphom*) or (young people adj3 lymphom*) or (young adult adj3 lymphom*)).ti,ab. 120
3 (Communicat* or Disclos* or inform* or Interact* or relationship* or Conversation* or Dialogue* or triad* or Interview* or consult* or decision making).ti,ab. 60609
4 (affect* or effect* or influenc* or resultant or impact* or perception* or perspective* or encounter* or preference or opinion or involvement or occurance* or feel or "go through" or experienc*).ti,ab. 143225
5 1 and 2 and 3 and 4 19
6 limit 5 to yr="2005 -Current" 14

For peer review only

First run – November 2022

Medline (via Ovid)

Ovid MEDLINE(R) and Epub Ahead of Print, In-Process, In-Data-Review & Other Non-Indexed Citations, Daily and Versions <1946 to November 23, 2022>

1 (Parent* or guardian* or mother* or father* or partner or wife or wives or husband* or boyfriend* or girlfriend* or sibling* or friend* or carer* or "third person" or caregiver* or "care-giver*" or spouse* or supporter* or support network*).ti,ab. or parents/ or fathers/ or mothers/ or spouses/ or caregivers/ or siblings/ or friends/ or legal guardians/ 1074121

2 (TYA cancer or TYA oncology or AYA cancer or AYA oncology or (young adult adj3 (cancer or oncology or leuk?em* or lymphom* or h?ematol*)) or ("teenage and young adult" adj3 cancer) or ("teenage and young adult" adj3 oncology) or (teenage* adj3 cancer) or (teenage* adj3 oncology) or (adolescen* adj3 cancer) or (adolescen* adj3 oncology) or (young people adj3 cancer) or (young people adj3 oncology) or ("teenage and young adult" adj3 leuk?emia*) or (teenage* adj3 leuk?emia*) or (adolescen* adj3 leuk?emia*) or (young people adj3 leuk?emia*) or (young adult adj3 leuk?emia*) or ("teenage and young adult" adj3 h?ematol*) or (teenage* adj3 h?ematol*) or (adolescen* adj3 h?ematol*) or (young people adj3 h?ematol*) or (young adult adj3 h?ematol*) or ("teenage and young adult" adj3 lymphom*) or (teenage* adj3 lymphom*) or (adolescen* adj3 lymphom*) or (young people adj3 lymphom*) or (young adult adj3 lymphom*)).ti,ab. or ((exp adolescent/ or exp young adult/) and exp neoplasms/) 333070

3 (Communicat* or Disclos* or inform* or Interact* or relationship* or Conversation* or Dialogue* or triad* or Interview* or consult* or decision making).ti,ab. or exp communication/ or exp disclosure/ or exp information dissemination/ or exp physician-patient relations/ 5715959

4 (affect* or effect* or influenc* or resultant or impact* or perception* or perspective* or encounter* or preference or opinion or involvement or occurance* or feel or "go through" or experienc*).ti,ab. 12406352

5 1 and 2 and 3 and 4 3380

6 limit 5 to (english language and yr="2005 -Current") 2715

Embase (via Ovid)

Embase <1974 to 2022 November 23>

For peer review only

1 (TYA cancer or TYA oncology or AYA cancer or AYA oncology or (young adult adj3 (cancer or oncology or leuk?em* or lymphom* or h?ematol*)) or ("teenage and young adult" adj3 cancer) or ("teenage and young adult" adj3 oncology) or (teenage* adj3 cancer) or (teenage* adj3 oncology) or (adolescen* adj3 cancer) or (adolescen* adj3 oncology) or (young people adj3 cancer) or (young people adj3 oncology) or ("teenage and young adult" adj3 leuk?emia*) or (teenage* adj3 leuk?emia*) or (adolescen* adj3 leuk?emia*) or (young people adj3 leuk?emia*) or (young adult adj3 leuk?emia*) or ("teenage and young adult" adj3 h?ematol*) or (teenage* adj3 h?ematol*) or (adolescen* adj3 h?ematol*) or (young people adj3 h?ematol*) or (young adult adj3 h?ematol*) or ("teenage and young adult" adj3 lymphom*) or (teenage* adj3 lymphom*) or (adolescen* adj3 lymphom*) or (young people adj3 lymphom*) or (young adult adj3 lymphom*)).ti,ab. or ((exp *adolescent/ or exp *young adult/) and exp *neoplasm/) 9638

2 (Communicat* or Disclos* or inform* or Interact* or relationship* or Conversation* or Dialogue* or triad* or Interview* or consult* or decision making).ti,ab. or exp *interpersonal communication/ or exp *professional-patient relationship/ or exp *information dissemination/ or exp *conversation/ 6997005

3 (Parent* or guardian* or mother* or father* or partner or wife or wives or husband* or boyfriend* or girlfriend* or sibling* or friend* or carer* or "third person" or caregiver* or "care-giver*" or spouse* or supporter* or support network*).ti,ab. or *parent/ or *father/ or *mother/ or *spouse/ or *caregiver/ or *social worker/ or *sibling/ or *friend/ or *legal guardian/ 1339977

4 (affect* or effect* or influenc* or resultant or impact* or perception* or perspective* or encounter* or preference or opinion or involvement or occurance* or feel or "go through" or experienc*).ti,ab. 15453173

5 1 and 2 and 3 and 4 939

6 limit 5 to (english language and yr="2005 -Current") 873

PsycInfo (via Ebscohost)

#	Query	Limiters/Expanders	Last Run Via	Results
S11	S1 AND S2 AND S7 AND S8	Limiters - Publication Year: 2005-2022 Expanders - Apply equivalent subjects Narrow by Language: - english Search modes - Boolean/Phrase	Interface - EBSCOhost Research Databases Search Screen - Basic Search Database - APA PsycInfo	1,683
S10	S1 AND S2 AND S7 AND S8	Expanders - Apply equivalent subjects Narrow by Language: - english	Interface - EBSCOhost Research Databases Search Screen - Basic Search	1,981

For peer review only

		Search modes - Boolean/Phrase	Database - APA PsycInfo	
S9	S1 AND S2 AND S7 AND S8	Expanders - Apply equivalent subjects<br>Search modes - Boolean/Phrase	Interface - EBSCOhost Research Databases<br>Search Screen - Basic Search<br>Database - APA PsycInfo	2,017
S8	(affect* or effect* or influenc* or resultant or impact* or perception* or perspective* or encounter* or preference or opinion or involvement or occurance* or feel or "go through" or experienc*)	Expanders - Apply equivalent subjects<br>Search modes - Boolean/Phrase	Interface - EBSCOhost Research Databases<br>Search Screen - Basic Search<br>Database - APA PsycInfo	3,366,619
S7	(S5) or (S3)	Expanders - Apply equivalent subjects<br>Search modes - Boolean/Phrase	Interface - EBSCOhost Research Databases<br>Search Screen - Basic Search<br>Database - APA PsycInfo	13,719
S6	S4 AND S5	Expanders - Apply equivalent subjects<br>Search modes - Boolean/Phrase	Interface - EBSCOhost Research Databases<br>Search Screen - Basic Search<br>Database - APA PsycInfo	13,275
S5	((DE “neoplasms” OR DE "Benign Neoplasms" OR DE "Breast Neoplasms" OR DE "Endocrine Neoplasms" OR DE "Leukemias" OR DE "Melanoma" OR DE "Metastasis" OR DE "Nervous System Neoplasms" OR DE "Terminal Cancer"))	Expanders - Apply equivalent subjects<br>Narrow by SubjectAge: - adolescence (13-17 yrs)<br>Narrow by SubjectAge: - young adulthood (18-29 yrs)<br>Search modes - Boolean/Phrase	Interface - EBSCOhost Research Databases<br>Search Screen - Basic Search<br>Database - APA PsycInfo	13,275
S4	((DE “neoplasms” OR DE "Benign Neoplasms" OR DE "Breast Neoplasms" OR DE "Endocrine Neoplasms" OR DE "Leukemias" OR DE "Melanoma" OR DE "Metastasis" OR DE "Nervous System Neoplasms" OR DE "Terminal Cancer"))	Expanders - Apply equivalent subjects<br>Search modes - Boolean/Phrase	Interface - EBSCOhost Research Databases<br>Search Screen - Basic Search<br>Database - APA PsycInfo	58,767
S3	("TYA cancer" or "TYA oncology" or "AYA cancer" or "AYA oncology" or ("young adult" n3 (cancer or oncology or leuk?em* or lymphom* or h?ematol*)) or ("teenage and young adult" n3 cancer) or ("teenage and young adult" n3 oncology) or (teenage* n3 cancer) or (teenage* n3 oncology) or (adolescen* n3 cancer) or	Expanders - Apply equivalent subjects<br>Search modes - Boolean/Phrase	Interface - EBSCOhost Research Databases<br>Search Screen - Basic Search<br>Database - APA PsycInfo	1,864

For peer review only

	(adolescen* n3 oncology) or ("young people" n3 cancer) or ("young people" n3 oncology) or ("teenage and young adult" n3 leuk?emia*) or (teenage* n3 leuk?emia*) or (adolescen* n3 leuk?emia*) or ("young people" n3 leuk?emia*) or ("young adult" n3 leuk?emia*) or ("teenage and young adult" n3 h?ematol*) or (teenage* n3 h?ematol*) or (adolescen* n3 h?ematol*) or ("young people" n3 h?ematol*) or ("young adult" n3 h?ematol*) or ("teenage and young adult" n3 lymphom*) or (teenage* n3 lymphom*) or (adolescen* n3 lymphom*) or ("young people" n3 lymphom*) or ("young adult" n3 lymphom*))			
S2	(Communicat* or Disclos* or inform* or Interact* or relationship* or Conversation* or Dialogue* or triad* or Interview* or consult* or "decision making") or DE "communication" OR DE "information dissemination" OR DE "conversation"	Expanders - Apply equivalent subjects<br>Search modes - Boolean/Phrase	Interface - EBSCOhost Research Databases<br>Search Screen - Basic Search<br>Database - APA PsycInfo	2,423,980
S1	(Parent* or guardian* or mother* or father* or partner or wife or wives or husband* or boyfriend* or girlfriend* or sibling* or friend* or teacher* or social worker* or carer* or "third person" or caregiver* or "care-giver*" or spouse* or chaperone*) OR DE "parents" OR DE "mothers" OR DE "fathers" OR DE "spouses" OR DE "wives" OR DE "husbands" OR DE "siblings" OR DE "significant others" OR DE "social workers" OR DE "guardianship" OR DE "caregivers"	Expanders - Apply equivalent subjects<br>Search modes - Boolean/Phrase	Interface - EBSCOhost Research Databases<br>Search Screen - Basic Search<br>Database - APA PsycInfo	894,375

CINAHL (via Ebscohost)

[Accessibility Information and Tips]

Print Search History

Thursday, November 24, 2022 6:21:27 PM

#	Query	Limiters/Expanders	Last Run Via	Results
S7	S1 AND S2 AND S3 AND S4	Limiters - Published Date: 20050101-20221231<br>Expanders - Apply equivalent subjects<br>Narrow by Language: - english<br>Search modes - Boolean/Phrase	Interface - EBSCOhost Research Databases<br>Search Screen - Advanced Search<br>Database - CINAHL	1,837

For peer review only

S6	S1 AND S2 AND S3 AND S4	Limiters - Published Date: 20050101-20221231<br>Expanders - Apply equivalent subjects<br>Search modes - Boolean/Phrase	Interface - EBSCOhost Research Databases<br>Search Screen - Advanced Search<br>Database - CINAHL	1,866
S5	S1 AND S2 AND S3 AND S4	Expanders - Apply equivalent subjects<br>Search modes - Boolean/Phrase	Interface - EBSCOhost Research Databases<br>Search Screen - Advanced Search<br>Database - CINAHL	2,106
S4	(affect* or effect* or influenc* or resultant or impact* or perception* or perspective* or encounter* or preference or opinion or involvement or occurance* or feel or "go through" or experienc*)	Expanders - Apply equivalent subjects<br>Search modes - Boolean/Phrase	Interface - EBSCOhost Research Databases<br>Search Screen - Advanced Search<br>Database - CINAHL	3,016,184
S3	("TYA cancer" or "TYA oncology" or "AYA cancer" or "AYA oncology" or ("young adult" n3 (cancer or oncology or leuk?em* or lymphom* or h?ematol*)) or ("teenage and young adult" n3 cancer) or ("teenage and young adult" n3 oncology) or (teenage* n3 cancer) or (teenage* n3 oncology) or (adolescen* n3 cancer) or (adolescen* n3 oncology) or ("young people" n3 cancer) or ("young people" n3 oncology) or ("teenage and young adult" n3 leuk?emia*) or (teenage* n3 leuk?emia*) or (adolescen* n3 leuk?emia*) or ("young people" n3	Expanders - Apply equivalent subjects<br>Search modes - Boolean/Phrase	Interface - EBSCOhost Research Databases<br>Search Screen - Advanced Search<br>Database - CINAHL	59,927

For peer review only

	leuk?emia*) or ("young adult" n3 leuk?emia*) or ("teenage and young adult" n3 h?ematol*) or (teenage* n3 h?ematol*) or (adolescen* n3 h?ematol*) or ("young people" n3 h?ematol*) or ("young adult" n3 h?ematol*) or ("teenage and young adult" n3 lymphom*) or (teenage* n3 lymphom*) or (adolescen* n3 lymphom*) or ("young people" n3 lymphom*) or ("young adult" n3 lymphom*)) OR ((MH "adolescence+" OR MH "young adult+") AND (MH "neoplasms+"))			
S2	(Communicat* or Disclos* or inform* or Interact* or relationship* or Conversation* or Dialogue* or triad* or Interview* or consult* or "decision making") or MH "communication+" OR MH "discussion" OR MH "conversation" OR (MH "Professional-Patient Relations+")	Expanders - Apply equivalent subjects Search modes - Boolean/Phrase	Interface - EBSCOhost Research Databases Search Screen - Advanced Search Database - CINAHL	2,016,086
S1	(Parent* or guardian* or mother* or father* or partner or wife or wives or husband* or boyfriend* or girlfriend* or sibling* or friend* or teacher* or social worker* or carer* or "third person" or caregiver* or "care-giver*" or spouse* or chaperone*) OR MH "parents" OR MH "mothers" OR MH "fathers" OR MH "spouses" OR MH "siblings" OR MH "teachers" OR MH "social workers" OR MH "caregivers"	Expanders - Apply equivalent subjects Search modes - Boolean/Phrase	Interface - EBSCOhost Research Databases Search Screen - Advanced Search Database - CINAHL	544,991

Web of Science Core Collection

Web of Science Search Strategy (v0.1)

Database: Web of Science Core Collection

Entitlements:

- WOS.IC: 1993 to 2022
- WOS.CCR: 1985 to 2022

For peer review only

- WOS.SCI: 1900 to 2022
- WOS.AHCI: 1975 to 2022
- WOS.BHCI: 2008 to 2022
- WOS.BSCI: 2008 to 2022
- WOS.ESCI: 2017 to 2022
- WOS.ISTP: 1990 to 2022
- WOS.SSCI: 1956 to 2022
- WOS.ISSHP: 1990 to 2022

Searches:

1: TS=(Parent* or guardian* or mother* or father* or partner or wife or wives or husband* or boyfriend* or girlfriend* or sibling* or friend* or teacher* or social worker* or carer* or "third person" or caregiver* or "care-giver*" or spouse* or chaperone*)
Results: 2129759

2: TS=("TYA cancer" or "TYA oncology" or "AYA cancer" or "AYA oncology" or ("young adult" near/3 (cancer or oncology or leuk?em* or lymphom* or h?ematol*)) or ("teenage and young adult" near/3 cancer) or ("teenage and young adult" near/3 oncology) or (teenage* near/3 cancer) or (teenage* near/3 oncology) or (adolescen* near/3 cancer) or (adolescen* near/3 oncology) or ("young people" near/3 cancer) or ("young people" near/3 oncology) or ("teenage and young adult" near/3 leuk?emia*) or (teenage* near/3 leuk?emia*) or (adolescen* near/3 leuk?emia*) or ("young people" near/3 leuk?emia*) or ("young adult" near/3 leuk?emia*) or ("teenage and young adult" near/3 h?ematol*) or (teenage* near/3 h?ematol*) or (adolescen* near/3 h?ematol*) or ("young people" near/3 h?ematol*) or ("young adult" near/3 h?ematol*) or ("teenage and young adult" near/3 lymphom*) or (teenage* near/3 lymphom*) or (adolescen* near/3 lymphom*) or ("young people" near/3 lymphom*) or ("young adult" near/3 lymphom*)) Results: 7793

3: TS=(Communicat* or Disclos* or inform* or Interact* or relationship* or Conversation* or Dialogue* or triad* or Interview* or consult* or "decision making")
Results: 11889093

4: TS= (affect* or effect* or influenc* or resultant or impact* or perception* or perspective* or encounter* or preference or opinion or involvement or occurance* or feel or "go through" or experienc*) Results: 24306121

5: #4 AND #3 AND #2 AND #1 Results: 684

6: #4 AND #3 AND #2 AND #1 Results: 684

7: #4 AND #3 AND #2 AND #1 and 2005 or 2006 or 2007 or 2008 or 2009 or 2010 or 2011 or 2012 or 2013 or 2014 or 2015 or 2016 or 2017 or 2018 or 2019 or 2020 or 2021 or 2022 (Publication Years) Results: 644

For peer review only

8: #4 AND #3 AND #2 AND #1 and 2005 or 2006 or 2007 or 2008 or 2009 or 2010 or 2011 or 2012 or 2013 or 2014 or 2015 or 2016 or 2017 or 2018 or 2019 or 2020 or 2021 or 2022 (Publication Years) and English (Languages) Results: 619

For peer review only

PRISMA 2020 Checklist

Section and Topic	Item #	Checklist item	Location where item is reported
**TITLE**			
Title	1	Identify the report as a systematic review.	Page 1
**ABSTRACT**			
Abstract	2	See the PRISMA 2020 for Abstracts checklist.	Page 2
**INTRODUCTION**			
Rationale	3	Describe the rationale for the review in the context of existing knowledge.	Pages 3 and 4
Objectives	4	Provide an explicit statement of the objective(s) or question(s) the review addresses.	Page 4
**METHODS**			
Eligibility criteria	5	Specify the inclusion and exclusion criteria for the review and how studies were grouped for the syntheses.	Page 5
Information sources	6	Specify all databases, registers, websites, organisations, reference lists and other sources searched or consulted to identify studies. Specify the date when each source was last searched or consulted.	Page 4 and supplemental file
Search strategy	7	Present the full search strategies for all databases, registers and websites, including any filters and limits used.	Page 4, Table 1 and supplemental file 1
Selection process	8	Specify the methods used to decide whether a study met the inclusion criteria of the review, including how many reviewers screened each record and each report retrieved, whether they worked independently, and if applicable, details of automation tools used in the process.	Page 5
Data collection process	9	Specify the methods used to collect data from reports, including how many reviewers collected data from each report, whether they worked independently, any processes for obtaining or confirming data from study investigators, and if applicable, details of automation tools used in the process.	Pages 4 and 5
Data items	10a	List and define all outcomes for which data were sought. Specify whether all results that were compatible with each outcome domain in each study were sought (e.g. for all measures, time points, analyses), and if not, the methods used to decide which results to collect.	Page 5
	10b	List and define all other variables for which data were sought (e.g. participant and intervention characteristics, funding sources). Describe any assumptions made about any missing or unclear information.	Page 5
Study risk of bias assessment	11	Specify the methods used to assess risk of bias in the included studies, including details of the tool(s) used, how many reviewers assessed each study and whether they worked independently, and if applicable, details of automation tools used in the process.	Page 5
Effect measures	12	Specify for each outcome the effect measure(s) (e.g. risk ratio, mean difference) used in the synthesis or presentation of results.	Page 5
Synthesis methods	13a	Describe the processes used to decide which studies were eligible for each synthesis (e.g. tabulating the study intervention characteristics and comparing against the planned groups for each synthesis (item #5)).	Page 5
	13b	Describe any methods required to prepare the data for presentation or synthesis, such as handling of missing summary statistics, or data conversions.	Page 5
	13c	Describe any methods used to tabulate or visually display results of individual studies and syntheses.	Page 5
	13d	Describe any methods used to synthesize results and provide a rationale for the choice(s). If meta-analysis was performed, describe the model(s), method(s) to identify the presence and extent of statistical heterogeneity, and software package(s) used.	Page 5

For peer review only - http://bmjopen.bmj.com/site/about/guidelines.xhtml

PRISMA 2020 Checklist

Section and Topic	Item #	Checklist item	Location where item is reported
	13e	Describe any methods used to explore possible causes of heterogeneity among study results (e.g. subgroup analysis, meta-regression).	Pages 5 and 6
	13f	Describe any sensitivity analyses conducted to assess robustness of the synthesized results.	Not applicable
Reporting bias assessment	14	Describe any methods used to assess risk of bias due to missing results in a synthesis (arising from reporting biases).	Not applicable
Certainty assessment	15	Describe any methods used to assess certainty (or confidence) in the body of evidence for an outcome.	Not applicable
**RESULTS**			
Study selection	16a	Describe the results of the search and selection process, from the number of records identified in the search to the number of studies included in the review, ideally using a flow diagram.	Figure 1 page 6
	16b	Cite studies that might appear to meet the inclusion criteria, but which were excluded, and explain why they were excluded.	Figure 1 page 6
Study characteristics	17	Cite each included study and present its characteristics.	Table 2 – summary of articles pages
Risk of bias in studies	18	Present assessments of risk of bias for each included study.	Not reported
Results of individual studies	19	For all outcomes, present, for each study: (a) summary statistics for each group (where appropriate) and (b) an effect estimate and its precision (e.g. confidence/credible interval), ideally using structured tables or plots.	Not applicable
Results of syntheses	20a	For each synthesis, briefly summarise the characteristics and risk of bias among contributing studies.	Not applicable
	20b	Present results of all statistical syntheses conducted. If meta-analysis was done, present for each the summary estimate and its precision (e.g. confidence/credible interval) and measures of statistical heterogeneity. If comparing groups, describe the direction of the effect.	Not applicable
	20c	Present results of all investigations of possible causes of heterogeneity among study results.	Not applicable
	20d	Present results of all sensitivity analyses conducted to assess the robustness of the synthesized results.	Not applicable
Reporting biases	21	Present assessments of risk of bias due to missing results (arising from reporting biases) for each synthesis assessed.	Not applicable
Certainty of evidence	22	Present assessments of certainty (or confidence) in the body of evidence for each outcome assessed.	Not applicable
**DISCUSSION**			
Discussion	23a	Provide a general interpretation of the results in the context of other evidence.	Pages 10 and 11
	23b	Discuss any limitations of the evidence included in the review.	Pages 11 and 12

For peer review only

PRISMA 2020 Checklist

Section and Topic	Item #	Checklist item	Location where item is reported
	23c	Discuss any limitations of the review processes used.	Pages 11 and 12
	23d	Discuss implications of the results for practice, policy, and future research.	Page 12
**OTHER INFORMATION**			
Registration and protocol	24a	Provide registration information for the review, including register name and registration number, or state that the review was not registered.	Page 3
	24b	Indicate where the review protocol can be accessed, or state that a protocol was not prepared.	Supplemental file
	24c	Describe and explain any amendments to information provided at registration or in the protocol.	Not appliable
Support	25	Describe sources of financial or non-financial support for the review, and the role of the funders or sponsors in the review.	Page 13
Competing interests	26	Declare any competing interests of review authors.	Page 13
Availability of data, code and other materials	27	Report which of the following are publicly available and where they can be found: template data collection forms; data extracted from included studies; data used for all analyses; analytic code; any other materials used in the review.	Page 13

From: Page MJ, McKenzie JE, Bossuyt PM, Boutron I, Hoffmann TC, Mulrow CD, et al. The PRISMA 2020 statement: an updated guideline for reporting systematic reviews. BMJ 2021;372:n71. doi: 10.1136/bmj.n71

For more information, visit: http://www.prisma-statement.org/

For peer review only
